# Exploring Three Avenues: Chemo- and Regioselective Transformations of 1,2,4-Triketone Analogs into Pyrazoles and Pyridazinones

**DOI:** 10.3390/ijms241814234

**Published:** 2023-09-18

**Authors:** Yulia O. Edilova, Ekaterina A. Osipova, Pavel A. Slepukhin, Victor I. Saloutin, Denis N. Bazhin

**Affiliations:** 1 Postovsky Institute of Organic Synthesis, Ural Branch of the Russian Academy of Sciences, 620108 Yekaterinburg, Russiasaloutin@ios.uran.ru (V.I.S.); 2 Department of Organic and Biomolecular Chemistry, Ural Federal University Named after the First President of Russia B.N. Eltsin, 620002 Yekaterinburg, Russia

**Keywords:** 1,2,4-triketone analogs, pyrazole, pyridazine, regioselectivity, chemoselectivity, building blocks

## Abstract

A convenient approach to substituted pyrazoles and pyridazinones based on 1,2,4-triketones is presented. Chemo- and regiocontrol in condensations of *t*-Bu, Ph-, 2-thienyl-, and CO_2_Et-substituted 1,2,4-triketone analogs with hydrazines are described. The direction of preferential nucleophilic attack was shown to be switched depending on the substituent nature in triketone as well as the reaction conditions. The acid and temperature effects on the selectivity of condensations were revealed. Regiochemistry of heterocyclic core formation was confirmed by NMR and XRD studies. The facile construction of heterocyclic motifs bearing acetyl and (or) carbethoxy groups suggests them as promising mono- or bifunctional building blocks for subsequent transformations.

## 1. Introduction

**Figure 1:**
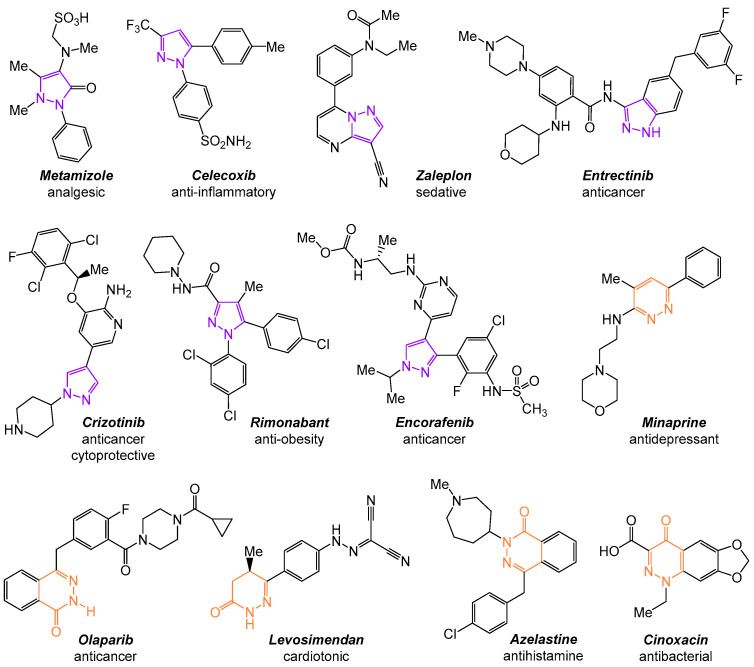
Representative examples of drugs containing pyrazole and pyridazine moieties.

Among the biologically important diazoles and diazines, one of the most privileged heterocycles are pyrazoles [1,2,3,4,5,6,7,8,9] and pyridazines [10,11,12,13,14], which are rarely found in nature but have been discovered as versatile pharmacophores. These heterocycles offer the ability to engage in diverse types of molecular interactions and demonstrate favorable physicochemical properties such as lipophilicity and water solubility [15,16,17]. Pyrazoles are of great interest as structural elements of medicinal drugs, exhibiting anti-inflammatory (celecoxib [18]), anticancer (ruxolitinib [19,20], entrectinib [21]), analgesic (metamizole [22]), anti-obesity (rimonabant [23]), cytoprotective (crizotinib [24]), and sedative (zaleplon [25]) effects (Figure 1). At the same time, pyridazine derivatives have attracted extensive attention due to their broad spectrum of biological activity, which has found use in therapeutic agents for the treatment of vasoconstriction (levosimendan [26]), allergies (azelastine [27]), cancer (olaparib [28]), and depression (minaprine [29]). In particular, pyridazinone scaffold is considered crucial for the discovery of drugs [11,14,30,31,32,33,34,35].

**Figure 2:**
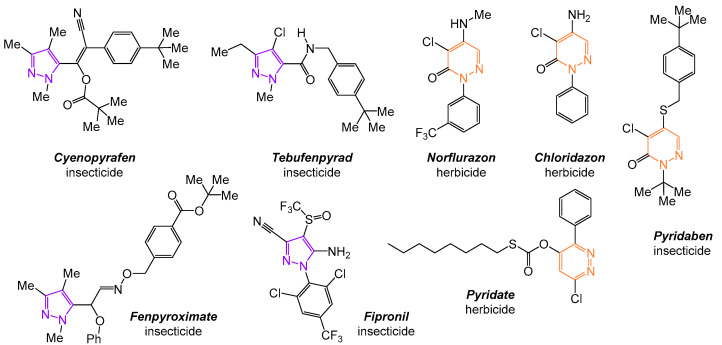
Pyridazine- and pyrazole-based agrochemicals.

The ability of these heterocycles to act as effective agrochemicals also highlights their potential for the selective targeting of biological systems [36,37]. For crop growth control photosynthesis inhibitors norflurazon [38], chloridazon [39], and pyridate [37] are used as pyridazine-based herbicides along with insecticides (pyridaben [40]). The pesticidal activity of the 1,2-diazole core can be illustrated by a range of *N*-phenylpyrazoles [41], among which fipronil [42] is the most popular insecticide, and also by methyl-substituted analogs such as cyenopyrafen [43], tebufenpyrad, and fenpyroximate [2,44] (Figure 2).

Since the structural features have extremely significant effects on the physical and drug-like properties of pyrazole and pyridazine derivatives, the starting materials for the synthesis of promising pharmaceuticals and agrochemicals based on these motifs should have a flexible structure for the fine tuning of the desired characteristics. In this context, the building block strategy has been proven to be a convenient approach to modified heterocycles with two adjacent nitrogen atoms. The classical method for creating pyrazoles is based on the condensation reaction of 1,3-dicarbonyl compounds (β-diketones and 3-ketoesters [45,46,47,48]) or α,β-unsaturated ketones [49,50] with hydrazines. One further route affording the pyrazole core formation involves [3+2] cycloaddition reactions of diazocompounds, nitrilimines with alkenes or alkynes [51,52,53,54,55]. For the synthesis of pyridazine ring systems, condensation reactions of 1,4-bifunctional reagents (4-oxocarboxylic acids or furan-2(5*H*)-ones [56,57,58,59], cyclic anhydrides [60,61]) with hydrazines commonly used as well as the Diels–Alder cycloaddition approach [62,63].

However, there are some limitations associated with reactions of unsymmetrical diketones and their synthetic equivalents with substituted hydrazines. The major difficulty is that they can sometimes suffer from regioselectivity issues, leading to the formation of multiple isomeric products [64,65,66,67,68]. In some cases, using specific catalysts along with varying the reaction parameters allow for the precise manipulation of the reactivity of different carbonyl groups.

Nevertheless, β-diketones are overwhelmingly used as easily available and highly reactive compounds, offering a straightforward and versatile approach to accessing a wide range of five- and six-membered nitrogen containing heterocycles [69,70]. One important benefit comes from the modification of 1,3-diketones with various substituents including functional groups, which makes it possible to directly introduce them into the desired positions of heterocycles when it is problematic to do otherwise. This opens up new possibilities for the construction of more complex molecules as well as fused heterocycles with improved biological activities [8,71].

**Figure 3:**
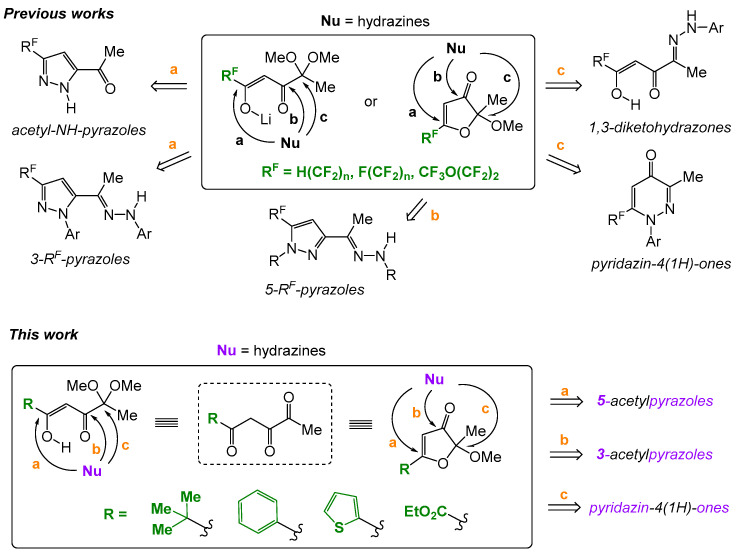
Different chemo- and regioselectivity in the reactions of fluorinated and non-fluorinated 1,2,4-triketones with hydrazines.

The synthetic potential of β-diketones can be further expanded by incorporating an additional keto group and thereby turning to a 1,2,4-triketone scaffold. The literature provides scarce data on the synthesis and transformations of such triketones, in contrast to closely related class of 2,4-diketoesters [72,73]. Substantially more attention has been paid to the 1,2,4-triketone analogs, mainly fluorine-containing [74,75,76,77,78]. These compounds combine the reactivity of α-, β-, and γ-dicarbonyl systems, opening the route to a wider range of possible products via interaction with binucleophiles [79]. Previously fluorinated acetal-containing lithium β-diketonates and their cyclic derivatives—furan-3(2*H*)-ones—were found to be the polyfunctional building blocks for the preparation of 3-R^F^ and 5-R^F^ pyrazoles, pyridazine-4(1*H*)-ones, and β-diketohydrazones during condensations with arylhydrazines [80,81,82] (Figure 3). In this case, the solvent-dependent regiocontrol strategy turned out to be effective.

Overall, the nature of a fluoroalkyl substituent in 1,2,4-triketones was responsible for the distinctive reaction pattern, leading to three different routes [79]. In this regard, similar transformations of non-fluorinated derivatives must be considered for in-depth study and the achievement of regio- and chemocontrol in reactions of tricarbonyl compounds with hydrazines. Moreover, revealing the electronic effects of the substituents and evaluating steric control provide access to specific isomers.

With this aim in mind, we designed a series of novel 1,2,4-triketone analogs bearing alkyl, aryl, heteroaryl, and functional groups and explored how they affect the direction of condensations with hydrazines compared to fluorinated substituents (Figure 3). Here, we discuss the ability to control their influence to access either pyridazines or pyrazoles of isomeric structure with high selectivity. This work will ensure the use of 1,2,4-triketones as polyfunctional starting materials for the synthesis of multiple heterocyclic scaffolds that can be subsequently functionalized.

## 2. Results and Discussion

**Scheme 1:**
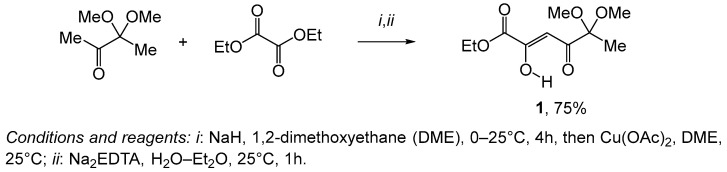
Synthetic route to acetal-functionalized 2,4-diketoester **1**.

The base-promoted Claisen condensation was chosen as the main approach to novel 1,2,4-triketone analogs. Acetal- and ester-functionalized β-diketone **1** was obtained by the reaction of 3,3-dimethoxybutan-2-one with ethyl oxalate in the presence of sodium hydride (Scheme 1). Since sodium β-diketonates are highly soluble in organic solvents and water, we used a convenient method to isolate compound **1**. This procedure includes the formation of the copper(II) complex by adding Cu(OAc)_2_ to the rection mixture and the further treatment of the Cu(II) chelate with Na_2_EDTA (disodium ethylenediamine tetraacetate) [75].

**Scheme 2:**
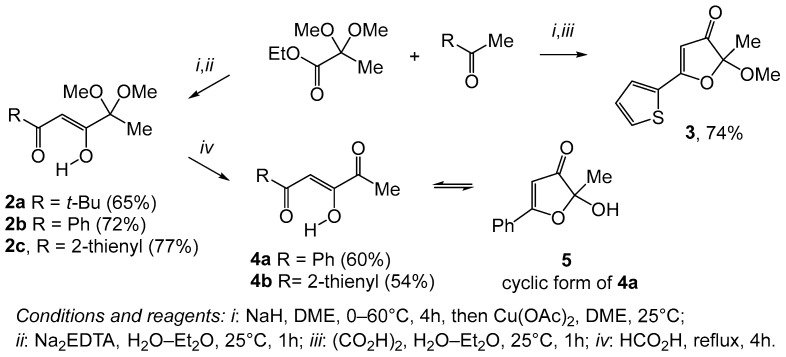
Design of novel 1,2,4-triketones **4a**,**b** and their synthetic equivalents **2a**–**c**, **3**.

The same access has been successfully applied to the synthesis of tricarbonyl derivatives containing alkyl, aryl, and heteroaryl fragments. Ethyl 2,2-dimethoxypropanoate was reacted with a series of methyl ketones giving 1,2,4-triketone analogs **2a–c** bearing *tert*-butyl, phenyl, and 2-thienyl substituents (Scheme 2). It was found that using oxalic acid instead of Na_2_EDTA for the decomposition of the Cu(II) complex based on 2-thienyl-substituted diketone led to the formation of cyclic product **3**. Moreover, the partial crystallization of 1,2,4-triketone **2b** to 2-hydroxy-2-methyl-5-phenylfuran-3(2*H*)-one **5** occurred during storage in air, affording a few crystals suitable for XRD studies. In this regard, the acid-catalyzed intramolecular cyclization of compounds **1**, **2a**–**c** was attempted. However, only aryl-substituted triketones **4a**,**b**, existing in enolized form, were isolated and characterized (Scheme 2). In the case of the *tert*-butyl **2a** and ester **1** substituents, small amounts of 1,2,4-triketones and furanones were observed in a mixture with the decomposition products according to GC-MS analysis. It should be noted that although 2-hydroxyfuran-3(2*H*)-one **5** is stable in a solid state, it is mainly observed in the open-chain form when dissolved in a nonpolar solvent (CDCl_3_). β-Diketones **2c** and **4a**,**b** and furan-3(2*H*)-ones **3** and **5** are also stable as powders, while the other 1,2,4-tricarbonyl analogs exist as oils at normal conditions.

**Scheme 3:**
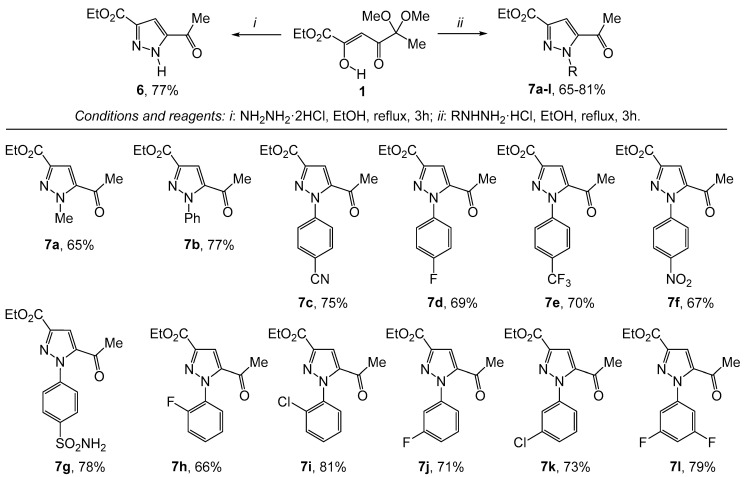
Reactions of CO_2_Et-functionalized 1,2,4-triketone analog **1** providing 3,5-bifunctional pyrazoles **6** and **7a**–**l**.

As previously mentioned, hydrazine dihydrochloride is a convenient reagent providing for the formation of heterocyclic products in the absence of acid catalysis [80]. The transformations of acetal-containing 2,4-diketoester **1** were considered starting from reaction with this binucleophile. As a result of reflux in EtOH, the *NH*-pyrazole **6** was formed (Scheme 3, see Section 3.2. *Method A*). Turning to the methyl- and phenylhydrazines, 5-acetylpyrazoles **7a**,**b** as the sole products were obtained. Likewise, reactions between 1,2,4-triketone **1** and the substituted arylhydrazines proceeded in a regiospecific manner. A large series of *N*-aryl-5-acetyl-1*H*-pyrazole-3-carboxylates **7c**–**l** was synthesized (Scheme 3). The pyrazoles **7c**–**l** were easily isolated with the high yields as the precipitates from reaction mixtures.

Accordingly, the direction of the initial nucleophilic attack is strongly determined by influence of the ethoxycarbonyl group, despite the hydrazine structure. It provides a wide range of bifunctional pyrazole derivatives, which are of interest for further modification via the acetyl and ester fragments.

In order to compare the electron-withdrawing and -donating effects of the substituents in 1,2,4-triketones, the chemical properties of *tert*-butyl, phenyl-, and 2-thienyl-substituted analogs were also examined in reactions with different binucleophiles. Herewith, unsubstituted hydrazine, methyl-, and phenylhydrazines were used as the most illustrative examples.

**Scheme 4:**
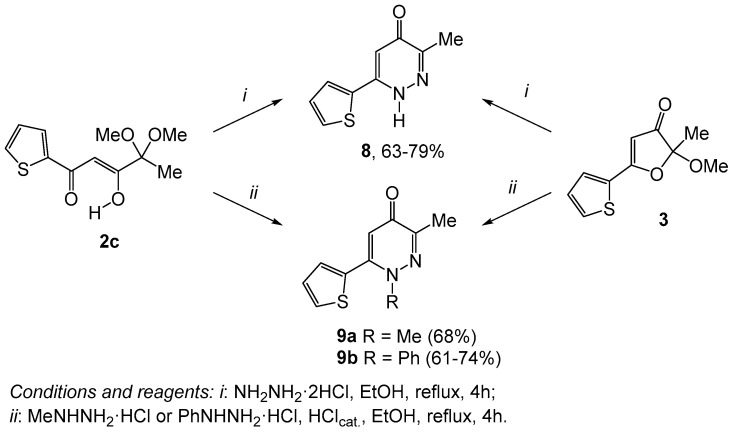
Chemoselective transformations of 2-thienyl-substituted 1,2,4-triketone analogs **2c** and **3** into pyridazine-4(1*H*)-ones **8** and **9a**,**b**.

It was found that both β-diketone **2c** and the furan-3(2*H*)-one **3** bearing the 2-thienyl fragment give pyridazine-4(1*H*)-ones **8** and **9** during reflux with binucleophiles in EtOH (Scheme 4). To improve the conversion in reactions with substituted hydrazines, hydrochloric acid was used as a catalyst. In these cases, binucleophiles attack the acetal carbon atom, leading to cyclization or recyclization of compounds **2c** and **3** to six-membered products **8** and **9**. Similarly to CO_2_Et-functionalized β-diketone **1**, the reaction pathway is affected by the nature of the substituent near the 1,3-dicarbonyl fragment and does not depend upon the hydrazine structure.

**Scheme 5:**
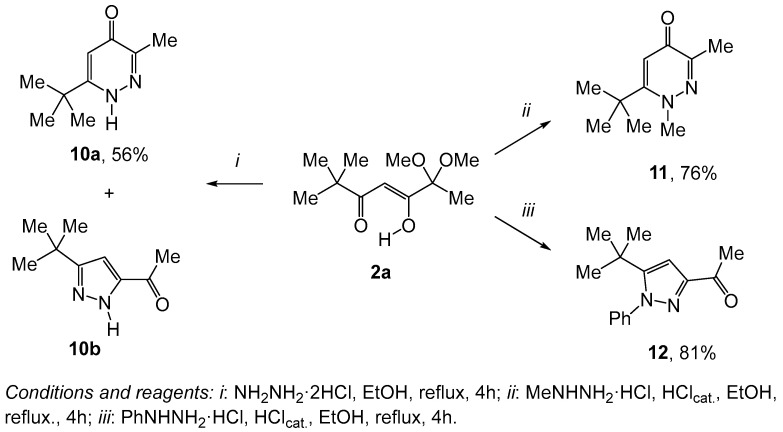
Acid-catalyzed condensations of *t*-Bu-substituted triketone **2a** with hydrazines.

When *t*-Bu-substituted 1,2,4-triketone **2a** was refluxed with hydrazine dihydrochloride, both five- and six-membered products were obtained. According to ^1^H NMR data, pyridazin-4(1*H*)-one **10a** and *NH*-pyrazole **10b** were formed in the ratio 3:2 (Scheme 5). It is worth noting that attempts to separate the product mixture by recrystallization were unsuccessful, thus column chromatography was required. The high chemoselectivity of the process was achieved during the reflux of *t*-Bu-containing β-diketone **2a** with substituted hydrazines. Being the strongest nucleophile, MeNHNH_2_ transforms compound **2a** into pyridazin-4(1*H*)-one **11** through interaction with the carbon atom of the acetal group (Scheme 5). At the same time, phenylhydrazine provides the formation of 3-regioisomeric acetylpyrazole **12** in accordance with the spectral and XRD studies.

**Scheme 6:**
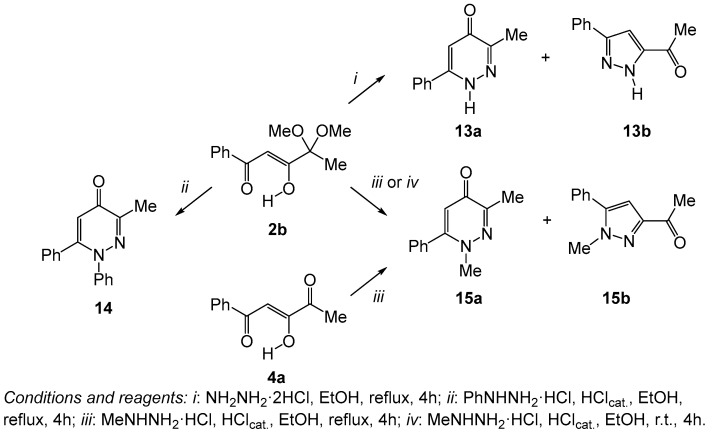
Multiple products obtained via the heterocyclization of Ph-containing building blocks **2b** and **4**.

Turning to Ph-containing 1,2,4-triketone **2b**, its cyclization with hydrazine was accompanied by the formation of two nitrogen heterocycles **13a** and **13b**, as in the case of *tert*-butyl analog **2a**. In addition, only one product was obtained by using PhNHNH_2_∙HCl, which was not pyrazole but pyridazinone **14** (Scheme 6). Although the reaction between diketone **2b** and methylhydrazine proceeded mainly via the 1,4-addition pathway under reflux, 3-acetylpyrazole **15b** was isolated along with pyridazinone **15a** in the ratio of 1:3, respectively. The yields of the products are presented in Table 1.

Since methylhydrazine is a strong nucleophile, we decreased the reaction temperature to enhance the selectivity of its initial attack. Nevertheless, similar transformations were observed with the predominant formation of a 1,3-addition product. Moreover, we turned to 1,2,4-triketone **4a** to evaluate the effect of the acetal group on the reaction pathway. During refluxing **4a** with MeNHNH_2_, the mixture of heterocycles **15a** and **15b** was observed in the same ratio as in the case of acetal-functionalized analog **2b** (Table 1).

One might conclude that hydrazine can attack either one or several positions of the 1,2,4-triketone concurrently. It depends only on the substituent nature in the building block and the nucleophilicity of hydrazine, and is not affected by the presence of a protecting group. Besides, the temperature has been found to be a significant and potent tool for directing the nucleophilic attack.

**Scheme 7:**
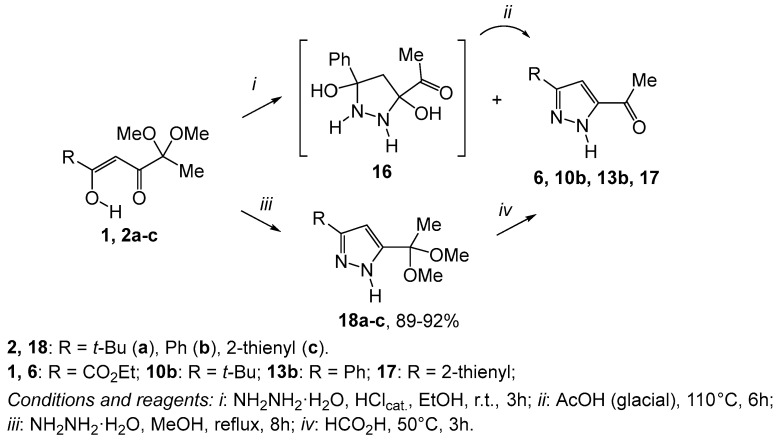
Effective approaches to the synthesis of acetylpyrazoles **6**, **10b**, **13b**, and **17**.

Based on these results, attempts have been made to gain control over the direction of the preferential nucleophile attack by varying the reaction conditions. Firstly, the influence of temperature was analyzed in condensations of 1,2,4-triketone analogs **1** and **2a**–**c** with hydrazine hydrate in the presence of HCl as a catalyst. This choice was made in light of the low solubility of RNHNH_2_∙HCl in ethanol at room temperature. Herewith, the corresponding *NH*-pyrazoles **6**, **10b**, **13b**, and **17** were obtained (Scheme 7, see Section 3.2. *Method B*). Only in the case of Ph-substituted β-diketone was the intermediate pyrazolidine **16** precipitated from the reaction mixture, preventing its further dehydration. According to the ^1^H NMR spectrum of compound **16**, protons of two NH- and two OH-groups appeared as multiplets in the ranges of 7.15–7.25 ppm and 13.45–13.55 ppm, respectively. In addition, signals of the acetyl group as well as CH_2_ fragment were observed at δ_H_ = 2.33–2.39 ppm. It was found that compound **16** could be converted to acetylpyrazole **13b** upon reflux in the excess of glacial acetic acid.

Next, the heterocyclization of compounds **2a**–**c** under acid-free conditions was investigated (Scheme 7). Recently, it has been demonstrated that fluorine-containing 1,2,4-triketone analogs can be easily cyclized to *NH*-pyrazoles in MeOH under the action of aqueous hydrazine while retaining the acetal group [83]. The behavior of *t*-Bu, Ph, and 2-thienyl-substituted β-diketones was not an exception, whereby heterocyclic compounds **18a**–**c** were obtained. It should be noted that the products **18a**–**c** open an alternative route to acetylpyrazoles **10b**, **13b**, and **17** via acid-catalyzed hydrolysis of the acetal fragment (see Section 3.2. *Method C*).

**Scheme 8:**
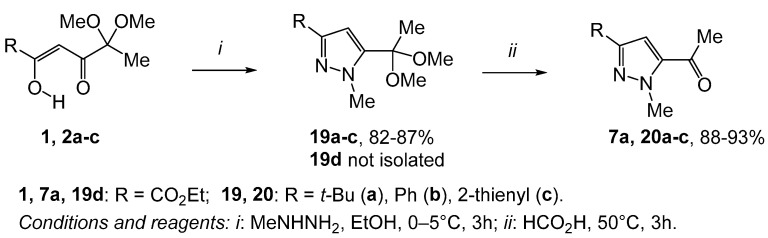
Two-step method for the preparation of 5-acetyl-*N*-methylpyrazoles **7a** and **20a**–**c**.

Following this method, the regiocontrolled transformations of 1,2,4-triketones **2a**–**c** to 5-acetyl-*N*-methylpyrazoles **20a**–**c** were performed without catalysis (Scheme 8). The selectivity of the methylhydrazine attack was found to increase when the reaction mixture was cooled, providing the formation of the acetal-containing pyrazoles **19a**–**c**. Further hydrolysis of compounds **19a**–**c** carried out in formic acid led to the target products. For triketone **1**, regiospecific condensation with methylhydrazine afforded 5-acetylpyrazole-3-carboxylate **7a** as well as the reaction with the salt form of binucleophile.

Accordingly, there was no interaction of hydrazine with the carbon atom of the acetal group under mild conditions, since the formation of six-membered products was not detected. Furthermore, different regioisomeric pyrazoles are derived during condensation reactions with substituted hydrazines depending on whether the acid is used. This confirms that chemo- and regioselectivity of conversions is dictated not only by the structure of the reagents but can be definitely ruled by the temperature as well as by the catalysts.

**Scheme 9:**
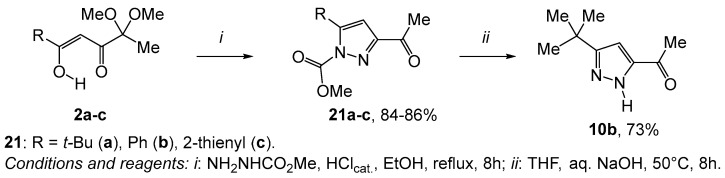
Regioselective synthesis of pyrazole-1-carboxylates **21a**–**c**.

Finally, to ensure that the substituent effect on the preferred reaction pathway is mainly of an electronic nature, a series of condensations was carried out involving hydrazine functionalized with the CO_2_Me group. While the reflux of triketones **2a**–**c** with methyl carbazate in the presence of HCl, 3-acetylpyrazole-1-carboxylates **21a**–**c** were formed, not-withstanding the substituent in the building block (Scheme 9). The compounds **21a**–**c** can also be considered as precursors for the preparation of *NH*-pyrazoles via base-induced ester group hydrolysis (see Section 3.2. *Method D*).

**Scheme 10:**
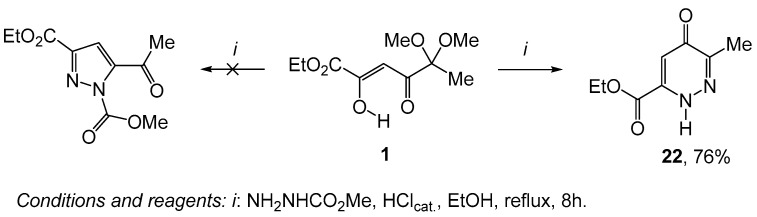
Unexpected formation of six-membered product **22** during the reaction between acetal-functionalized 2,4-diketoester **1** and methyl carbazate.

In contrast, acetal-containing 2,4-diketoester **1** did not yield pyrazole during the reaction with NH_2_NHCO_2_Me. The formation of pyridazinone **22** was observed as a result of the initial nucleophilic attack on the carbon atom of the acetal group, followed by step-by-step acid hydrolysis of the CO_2_Me fragment, decarboxylation, and intramolecular cyclization through the NH_2_ group interaction with the electrophilic center adjacent to the ester substituent (Scheme 10). 

**Scheme 11:**
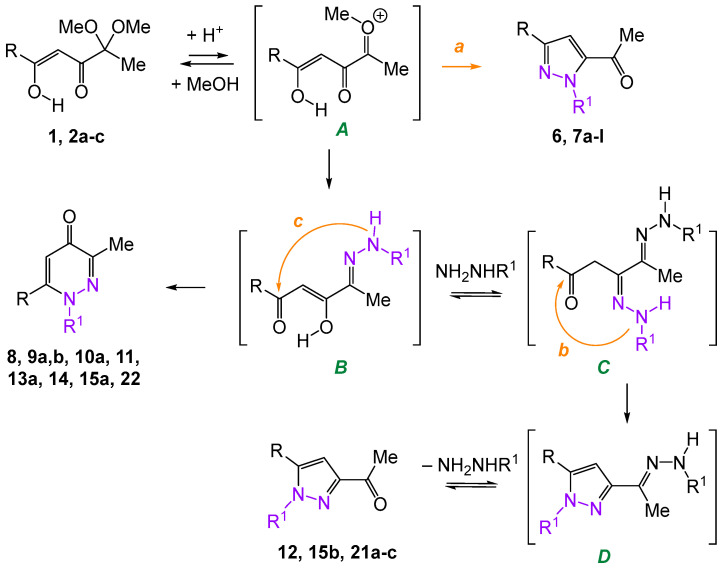
Proposed mechanism of acid-catalyzed heterocyclization of 1,2,4-triketone analogs **1** and **2a**–**c** into regioisomeric 3- and 5-acetylpyrazoles and pyridazinones.

The acid-catalyzed reactions between 1,2,4-triketone analogs **2a**–**c** and **3** and hydrazines can be described by the general mechanism proposed in Scheme 11. It includes three possible directions leading to the formation of regiosomeric 3- and 5-acetylpyrazoles (paths **a** and **b**) or pyridazinones (path **c**). The acid cleavage of the acetal fragment provides intermediate **A**. Herewith, two enolic forms of diketone **A** can exist, one of which becomes dominant depending on the nature of the substituent. Path **a**, yielding 5-acetylpyrazoles **6** and **7a**–**l** becomes possible in the presence of the CO_2_Et substituent. As a strong acceptor, it increases the reactivity of the adjacent enolized keto group, thereby the cyclocondensations proceed in a regiospecific manner. Regarding the reactions of *t*-Bu, Ph, thienyl-containing analogs, paths **b** and **c** are mostly competing, which makes the nature of the substituent in 1,2,4-triketone crucial for the structure of the products.

The 2-thienyl-triketone was found to exhibit a high chemoselectivity of transformations, which decreased when passing to the Ph and *t*-Bu-substituted analogs. During all reactions, hydrazines preferentially attacked the charged keto group of intermediate **A** (path **c**), providing pyridazinones **8**, **9a**,**b**, **10a**, **11**, **13a**,**14**, and **15a**, apart from the condensation of *t*-Bu-triketone with PhNHNH_2_. This exception can be induced by steric hindrances when the bulky *t*-Bu and Ph substituents approach one another as well as by the weak nucleophilicity of the -NHPh fragment, which prevents rapid dikethohydrazone **B** cyclization. Therefore, the second phenylhydrazine molecule can be attached via path **b** to form intermediate **C**, followed by the pyrazole ring closure and the cleavage of the first hydrazine molecule from pyrazolyl hydrazone **D**. This mechanism of the formation of 3-acetylpyrazoles **12** and **15b** corresponds to the conversions of fluorinated 1,2,4-triketones to 5-R^F^-pyrazoles by reactions with substituted arylhydrazines under similar conditions [81]. In other cases, the reaction temperature appeared to be the main decisive factor. It was found that 1,4-addition of binucleophiles (path **c**) proceeds by kinetic control, whereas path **b** is favored at lower temperatures, leading to pyrazoles as more thermodynamically stable products.

The features of the reactions involving NH_2_NHCO_2_Me should be considered separately. Methyl carbazate is a weak nucleophile and a harder base than other hydrazines, so it primarily attacks the positively charged keto group (the harder acid) of intermediate **A**. However, the formed dikethohydrazone **B** undergoes intramolecular cyclization to pyridazinone **22** only in the case of the strong acceptor CO_2_Et substituent. This confirms that the increased donor effects of the substituents and the reduced nucleophilicity of the NH group prevent the cyclization of both fluorinated [80] and non-fluorinated diketohydrazones. Thus, compounds **2a**–**c** give 3-acetylpyrazoles **21a**–**c** via a series of transformations, similar to those described above for the reaction between *t*-Bu-triketone and PhNHNH_2_.

It seems that the chemical behavior of non-fluorinated 1,2,4-triketones corresponds to β-diketones upon removing the acid from the reaction sphere. The nature of the substituent did not define the main route of these reactions, which was path **a**. Nevertheless, the selectivity of 5-acetylpyrazole formation from the *t*-Bu-, Ph-, and 2-thienyl-triketones was influenced by the temperature.

Taking this into account, one can highlight the key features. In contrast to the fluorine-containing 1,2,4-triketones, it does not matter in which form the non-fluorinated analogs react with hydrazines: acetal-functionalized β-diketones, 1,2,4-triketones, or furan-3(2*H*)-ones give identical products. Furthermore, these conversions tend to proceed non-selectively, although the reaction conditions are the same as for R^F^-triketones. Nevertheless, chemo- and regioselectivity have been demonstrated to be achievable.

**Figure 4:**
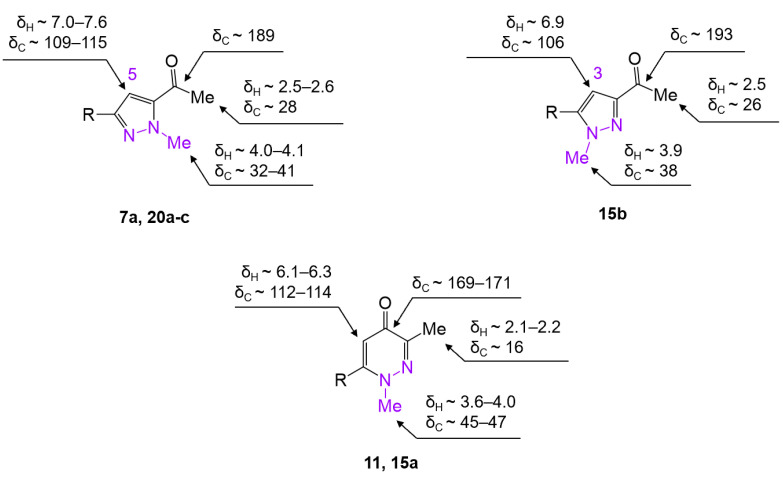
Characteristic ^1^H and ^13^C NMR signals (DMSO-*d*_6_, *δ*, ppm) for regioisomeric *N*-methylpyrazoles **7a**, **15b**, and **20a**–**c** and pyridazinones **11** and **15a**.

Analyzing the NMR spectroscopy data, the structure of the isomeric heterocyclic products can be proven. In ^1^H spectra registered in DMSO-*d*_6_ solution, the chemical shifts of the methyl groups of pyridazinones **8**, **10a**, **11**, **13a**, **14**, **15a**, **22** and acetylpyrazoles **7a**, **10b**, **12**, **13b**, **15b**, **20a**–**c**, and **21a** were observed in the ranges of 2.1–2.4 ppm and 2.4–2.6 ppm, respectively. The singlets of methine protons related to pyrazoles also appeared in a weaker field (δ_H_ = 6.4–7.6 ppm) than in the case of six-membered products (δ_H_ = 6.1–7.2 ppm). According to the ^13^C NMR data, for pyridazinones, the signals of the CH_3_ (15–17 ppm) and C=O groups (164–171 ppm) were upfield in comparison with the corresponding ranges detected for pyrazoles of δ_Me_ = 26–28 ppm and δ_C=O_ = 189–194 ppm. Likewise, these characteristic signals were used to correlate the regioisomeric structure of 3- and 5-acetylpyrazoles **7a**, **15b**, and **20a**–**c**, obtained as *N*-methyl derivatives (Figure 4). One can see clearly how δ_C_ values of the carbon atoms in the acetyl fragment depend upon its position in a pyrazole ring, along with the signals of the CH-group in both the ^1^H and ^13^C NMR spectra. 

To additionally confirm the structural features among the 1,2,4-triketone analogs and heterocyclic products, XRD experiments were performed for compounds **2c**, **3**, **5**, pyrazoles **7a**, **7l**, **12**, **15b**, **21b**, and pyridazinones **9a**, **9b**, and **14** (Figure 5 and Figures S99–S120, Tables S1–S6). For instance, the crystal packing differences were determined by H-contacts between the oxygen and nitrogen atoms of the functional groups, heterocyclic rings with hydrogen atoms of methyl groups and C–H fragments in (het)aromatic systems (Figures S110–S120, Table S6).

The enolic form of compound **2c** adopted a mostly planar conformation. The pseudo hexagonal cycle of the β-dicarbonyl fragment is characterized by the presence of the methine proton and intramolecular hydrogen bond between the oxygen atom and hydroxy group. Herewith, the values of the bond lengths and angles between carbon atoms of the enolic form **2c** corresponded to sp^2^ hybridization (Table S1).

The difference between methoxy- and hydroxy-substituted furanones **3** and **5** was found. In the case of compound **3**, the carbon atom bearing methoxy and methyl groups extended ~0.4 Å beyond the plane of the furan ring. In contrast, the furan ring of product **5** was in the same plane as the aryl substituent. The formation of intermolecular hydrogen bonds between the hydroxy and keto groups of compound **5** provided zigzag chains in which there were no π–π interactions between the aromatic fragments (Figure S112).

For pyrazoles **7a**, **7l**, **12**, **15b**, and **21b**, the crystal structure data provides an accurate determination of the substituent position at the nitrogen atom of the heterocyclic system. In contrast to 3-acetylpyrazoles **12**, **15b**, and **21b**, the carbonyl group in 5-substituted pyrazoles **7a** and **7l** was oriented toward the methyl or aryl substituent at the nitrogen atom.

In the case of heterocycles **7a**, **7l**, **9a**, **9b**, **12**, **14**, **15b**, and **21b**, the deviation of aromatic substituents from either pyrazole or pyridazine plane was observed (Table S3), which affects their crystal packing features (Figures S102–S109). Almost all compounds exhibited the formation of stacks due to the orientation of aryl or heterocyclic rings. However, not all of them corresponded to π–π stacking. It should be noted that π–π interactions with characteristic values of ~3.4 Å were observed for only one aromatic fragment among the products. As an exception, compound **7l** was found to realize π–π-stacking with a minimum value for the difluorophenyl rings while the interplanar distances between the pyrazole rings were increased to 3.6 Å.

## 3. Experimental

### 3.1. Materials and Methods

The solvents and reagents except for dimethoxybutan-2-one and ethyl 2,2-dimethoxypropanoate are commercially available (Alfa Aesar, Sigma-Aldrich, VEKTON) and were used without purification. Dimethoxybutan-2-one and ethyl 2,2-dimethoxypropanoate were synthesized according to the previously reported procedures [84]. The NMR spectra of the synthesized compounds (see Supplementary Materials) were recorded on Bruker DRX-400 and Bruker AVANCE^III^ 500 spectrometers (^1^H, 400.13 (DRX400) and 500.13 (AV500) MHz, ^13^C, 125.76 MHz, Me_4_Si as an internal standard; ^19^F, 376.44 (DRX400) and 470.52 (AV500) MHz, C_6_F_6_ as an internal standard). The microanalyses (C, H, N) were carried out on a PerkinElmer PE 2400 series II (PerkinElmer, Waltham, MA, USA) elemental analyzer. High-resolution mass spectrometry (HRMS) was performed using a Bruker Daltonics MaXis Impact HD mass spectrometer (Bruker, Karlsruhe, Germany) with positive electrospray ionization. Melting points were measured in open capillaries on a Stuart SMP30 melting point apparatus (Bibby Scientific Limited, Staffordshire, UK). The column chromatography was performed on silica gel 60 (0.062–0.2 mm) (Macherey-Nagel GmbH & Co KG, Duren, Germany).

### 3.2. General Procedures

Synthesis of compound **1**: Sodium hydride (4 g, 100 mmol; 60% in mineral oil) was slowly added at 0–5 °C to a solution of 3,3-dimethoxybutan-2-one (13.2 g, 0.1 mol) and ethyl oxalate (14.6 g, 0.1 mol) in 100 mL of 1,2-dimethoxyethane. The suspension was stirred at room temperature (25 °C) for 4 h, then Cu(OAc)_2_ (0.05 mol) and 120 mL of water were added. The precipitate was filtered off, dried, and added to the Et_2_O–H_2_O mixture (80 mL/80 mL). The copper(II) chelate was decomposed by stirring with Na_2_EDTA (0.05 mol) at r.t. (25 °C) for 1 h. Then, the organic layer was separated, dried over sodium sulfate, and distilled. 

Synthesis of compounds **2a**–**c** and **3**: Sodium hydride (100 mmol; 60% in mineral oil) was slowly added at 0–5 °C to a solution of ethyl 2,2-dimethoxypropanoate (0.1 mol) and corresponding methyl ketone (0.1 mol) in 100 mL of 1,2-dimethoxyethane. The suspension was stirred at room temperature (25 °C) for 1 h and then at 60 °C for 3 h. The products were isolated similarly to β-diketone **1** via the formation of Cu(II) chelates, decomposed by stirring with Na_2_EDTA or oxalic acid dihydrate (0.05 mol) at r.t. (25 °C) for 1 h. In the case of compounds **2c**, **3** the solvent was removed by evaporation.

Synthesis of compounds **4a**,**b**: 1,2,4-Triketone analogs **2b**,**c** (10 mmol) were refluxed in an excess of formic acid for 4 h. Then, water was added, the precipitate formed was filtered off, dried, and recrystallized from hexane.

Synthesis of compound **6** (*method A*): A mixture of 1,2,4-triketone analog **1** (3 mmol) and hydrazine dihydrochloride (3 mmol) was refluxed in 10 mL of EtOH for 3 h. Then, water was added, the mixture was extracted by Et_2_O (2 × 5 mL), the organic layer was separated, dried over magnesium sulfate, and the solvent was evaporated.

Synthesis of compounds **7a**–**l**: A mixture of 1,2,4-triketone analog **1** (3 mmol) and the corresponding hydrazine hydrochloride (3 mmol) was refluxed in 10 mL of EtOH for 3 h. The precipitate formed was filtered off, washed with NaHCO_3_ aqueous solution, and dried to give products **7b**–**l**. In the case of **7a**, the water was added to the reaction mixture, the precipitate formed was filtered off, and recrystallized from hexane.

Synthesis of compounds **8** and **9a**,**b**: A mixture of 2-thienyl-substituted 1,2,4-triketone analog **2c** (3 mmol) or furan-3(2*H*)-one **3** (3 mmol) and the corresponding hydrazine dihydrochloride (3 mmol) was refluxed in 10 mL of EtOH for 4 h. The solvent was evaporated and the residue was recrystallized from an appropriate solvent. 

Synthesis of compounds **10a** and **13a**: A mixture of 1,2,4-triketone analogs **2a**,**b** (3 mmol) and hydrazine dihydrochloride (3 mmol) was refluxed in 10 mL of EtOH for 4 h. The solvent was evaporated, the residue was washed by EtOAc, immobilized on silica gel, and purified by column chromatography (eluent for **10a**: CHCl_3_–Et_2_O/4:1, CHCl_3_–Et_2_O/1:1; eluent for **13a**: CHCl_3_–Et_2_O/1:1, EtOAc).

Synthesis of compounds **11**, **12**, and **14**: A mixture of 1,2,4-triketone analogs **2a**,**b** (3 mmol) and the substituted hydrazine (3 mmol) was refluxed in 10 mL of EtOH for 4 h. The solvent was evaporated, and the residue was recrystallized from hexane or Et_2_O.

Synthesis of compounds **15a**,**b**: A mixture of phenyl-substituted 1,2,4-triketone analog **2b** (3 mmol) and methyl hydrazine hydrochloride (3 mmol) was refluxed in 10 mL of EtOH for 4 h, then treated with NaHCO_3_ and filtered off. The filtrate was evaporated and the residue was purified by column chromatography (eluent: CHCl_3_–hexane/1:1, CHCl_3_–EtOAc/1:1).

Synthesis of compounds **6**, **10b**, **13b**, and **17** (*method B*): A mixture of 1,2,4-triketone analogs **1** and **2a**–**c** (3 mmol) and hydrazine monohydrate (3 mmol) was stirred in 10 mL of EtOH and 0.5 mL of HCl at room temperature (25 °C) for 8 h. The solvent was evaporated and the residue was recrystallized from an appropriate solvent. In the case of Ph-substituted products, the precipitate formed in the reaction mixture was filtered off to give pyrazolidine **16**, then the filtrate was evaporated, and the residue was washed by hexane and recrystallized from EtOAc to give **13b**.

Synthesis of compounds **18a**–**c**: A mixture of 1,2,4-triketone analogs **2a**–**c** (5 mmol) and hydrazine hydrate (5 mmol) was refluxed in 12 mL of MeOH for 3 h. The solvent was evaporated and the residue was recrystallized from hexane.

Synthesis of compounds **10b**, **13b**, and **17** (*method C*): An appropriate pyrazole bearing the acetal group **18a**–**c** (3 mmol) was heated at 50 °C with stirring in an excess of formic acid for 3 h. Then, water was added, and the precipitate formed was filtered off and dried without further purification.

Synthesis of compounds **19a**–**c**: To a solution of 1,2,4-triketone analogs **2a**–**c** (5 mmol) in 15 mL of EtOH methyl hydrazine was added dropwise at 0–5 °C. The mixture was stirred for 3 h, then the solvent was evaporated, and the residue was recrystallized from hexane.

Synthesis of compounds **20a**–**c**: An appropriate pyrazole bearing the acetal group **19a**–**c** (3 mmol) was heated at 50 °C with stirring in an excess of formic acid for 3 h. Then, water was added, the mixture was extracted by CHCl_3_ (2 × 7 mL), the organic layer was separated, dried over sodium sulfate, and the solvent was evaporated.

Synthesis of compounds **21a**–**c** and **22**: A mixture of 1,2,4-triketone analogs **1** and **2a**–**c** (5 mmol) and methyl carbazate (5 mmol) was refluxed in 15 mL of EtOH and 1 mL of HCl for 8 h. Then, the solvent was evaporated, and the residue was recrystallized from an appropriate solvent to give **21a** and **22**. In the case of **21b**,**c** the precipitate formed was filtered off, dried, and recrystallized from Et_2_O.

Synthesis of compound **10b** (*method D*): Pyrazole bearing the methyl ester group **21a** (3 mmol) was dissolved in 5 mL of THF, then an aqueous solution of NaOH (5 mmol) was added and the mixture was heated at 50 °C with stirring for 8 h. The solution was neutralized with 0.1 M HCl and extracted with THF, the organic layer was separated, dried over sodium sulfate, and the solvent was evaporated.

### 3.3. Spectral and Elemental Analysis Data of Synthesized Compounds

*Ethyl 4-hydroxy-5,5-dimethoxy-2-oxohex-3-enoate* (**1**). Yield 17.42 g (75%); yellow oil; bp 157–159 °C (10 torr). ^1^H NMR (400 MHz, CDCl_3_) δ 1.39 (t, *J* = 7.1 Hz, 3H, OCH_2_CH_3_), 1.46 (s, 3H, Me), 3.27 (s, 6H, 2MeO), 4.37 (q, *J* = 7.1 Hz, 2H, OCH_2_CH_3_), 6.81 (s, 1H, CH), 14.11 (br. s, 1H, OH); ^13^C NMR (125 MHz, CDCl_3_) δ 14.0 (CH_3_^ester^), 20.7 (Me), 49.7 (MeO), 62.6 (CH_2_^ester^), 99.3 (CH), 101.1 (C^acetal^), 161.8 (C=O^ester^), 167.3 (C^enol^), 200.7 (C=O). IR ν 3300–2890 (C–H, O–H), 1763 (C=O_ester_), 1675 (C=O), 1530–1439 (C=C), 1138, 1124 (C-O). HRMS *m/z* 233.1023 (calcd. for C_10_H_17_O_6_ [M + H]^+^ 233.1020).

*5-Hydroxy-2,2-dimethoxy-6,6-dimethylhept-4-en-3-one* (**2a**). Yield 14.05 g (65%); colorless oil; ^1^H NMR (400 MHz, CDCl_3_) δ 1.20 (s, 9H, *t*-Bu), 1.47 (s, 3H, Me), 3.25 (s, 6H, 2MeO), 6.08 (s, 1H, CH); ^13^C NMR (125 MHz, CDCl_3_) δ 21.3 (Me), 27.2 (CH_3_*^t^*^-Bu^), 39.5 (C*^t^*^-Bu^), 49.4 (MeO), 93.2 (CH), 100.5 (C^acetal^), 191.2 (C^enol^), 202.4 (C=O). IR ν 3500–2870 (C–H, O–H), 1683 (C=O), 1552–1460 (C=C), 1144, 1123 (C–O). HRMS *m/z* 217.1427 (calcd. for C_11_H_21_O_4_ [M + H]^+^ 217.1434).

*1-Hydroxy-4,4-dimethoxy-1-phenylpent-1-en-3-one* (**2b**). Yield 17.01 g (72%); brown oil; ^1^H NMR (400 MHz, CDCl_3_) δ 1.52 (s, 3H, Me), 3.30 (s, 6H, 2MeO), 6.68 (s, 1H, CH), 7.45– 7.49 (m, 2H, 2CH_Ar_), 7.53–7.57 (m, 1H, CH_Ar_), 7.94–7.97 (m, 2H, 2CH_Ar_), 16.01 (c, 1H, OH); ^13^C NMR (125 MHz, CDCl_3_) δ 21.3 (Me), 49.6 (MeO), 94.1 (CH), 100.7 (C^acetal^), 127.3 (C^Ph^), 128.6 (C^Ph^), 132.7 (C^Ph^), 134.7 (C^Ph^), 184.5 (C^enol^), 193.3 (C=O). IR ν 3181(O–H), 3090–2850 (C–H), 1670 (C=O), 1565–1454 (C=C), 1144, 1121 (C–O). HRMS *m/z* 237.1120 (calcd. for C_13_H_17_O_4_ [M + H]^+^ 237.1121).

*1-Hydroxy-4,4-dimethoxy-1-(thiophen-2-yl)pent-1-en-3-one* (**2c**). Yield 18.66 g (77%); green powder; mp 75–77 °C; ^1^H NMR (400 MHz, CDCl_3_) δ 1.53 (s, 3H, Me), 3.28 (s, 6H, 2MeO), 6.51 (s, 1H, CH), 7.15 (dd, *J* = 4.7, 3.8 Hz, 1H, CH_Ar_), 7.64 (dd, *J* = 5.0, 1.2 Hz, 1H, CH_Ar_), 7.79 (dd, *J* = 3.7, 1.2 Hz, 1H, CH_Ar_); ^13^C NMR (125 MHz, CDCl_3_) δ 21.4 (Me), 49.4 (MeO), 94.7 (CH), 100.1 (C^acetal^), 128.3 (C^Ar^), 131.0 (C^Ar^), 133.1 (C^Ar^), 141.6 (C^Ar^), 182.7 (C^enol^), 186.2 (C=O). IR ν 3115–2833 (C–H, O–H), 1604 (C=O), 1573, 1515, 1403 (C=C), 1148, 1122 (C–O). HRMS *m/z* 265.0506 (calcd. for C_11_H_14_NaO_4_S [M + Na]^+^ 265.0505).

*2-Methyl-2-methoxy-5-(thiophen-2-yl)furan-3(2H)-one* (**3**). Yield 15.56 g (74%); brown powder; mp 109–111 °C; ^1^H NMR (500 MHz, CDCl_3_) δ 1.60 (s, 3H, Me), 3.33 (s, 3H, MeO), 5.88 (s, 1H, CH), 7.22 (dd, *J* = 5.1, 3.8 Hz, 1H, CH_Ar_), 7.70 (dd, *J* = 5.1, 1.2 Hz, 1H, CH_Ar_), 7.76 (dd, *J* = 3.8, 1.2 Hz, 1H, CH_Ar_); ^13^C NMR (125 MHz, CDCl_3_) δ 21.4 (Me), 52.3 (MeO), 98.4 (CH), 108.6 (C^acetal^), 128.7 (C^Ar^), 131.4 (C^Ar^), 131.6 (C^Ar^), 132.7 (C^Ar^), 178.3 (C=), 199.7 (C=O); Anal. calcd. for C_10_H_10_O_3_S. C, 57.13; H, 4.79. Found: C, 57.06; H, 4.80. IR 3055–2899 (C–H), 1697 (C=O), 1602, 1589, 1567, 1478 (C=C), 1152, 1132 (C–O).

*3-Hydroxy-1-phenylpent-2-ene-1,4-dione* (**4a**). Yield 1.141 g (60%); white powder; mp 99–100 °C (litr. 95–96 °C [85]); ^1^H NMR (400 MHz, CDCl_3_) triketone **4a**/furanone **5** = 9/1. **Triketone 4a** ^1^H NMR δ 2.50 (s, 3H, Me), 6.95 (s, 1H, CH), 7.48–7.53 (m, 2H, 2CH_Ar_), 7.58–7.63 (m, 1H, CH_Ar_), 7.98–8.01 (m, 2H, 2CH_Ar_); ^13^C NMR (125 MHz, CDCl_3_) δ 25.1 (Me), 93.8 (CH), 127.8 (C^Ph^), 128.9 (C^Ph^), 133.6 (C^Ph^), 135.2 (C^Ph^), 173.8 (C^enol^), 190.7 (C=O), 197.6 (C=O). **Furanone 5** ^1^H NMR δ 1.68 (s, 3H, Me), 5.98 (s, 1H, CH), 7.48–7.53 (m, 2H, 2CH_Ar_), 7.58–7.63 (m, 1H, CH_Ar_), 7.98–8.01 (m, 2H, 2CH_Ar_); ^13^C NMR (125 MHz, CDCl_3_) δ 22.2 (Me), 97.6 (CH), 104.2 (C–OH), 127.5 (C^Ph^), 128.9 (C^Ph^), 133.3 (C^Ph^), 133.1 (C^Ph^), 183.8 (C=), 201.4 (C=O). Anal. calcd. for C_11_H_10_O_3_. C, 69.46; H, 5.30. Found: C, 69.51; H, 5.29. IR ν 3313–2854 (C–H, O–H), 1675, 1588 (C=O), 1546–1449 (C=C). HRMS *m/z* 191.0702 (calcd. for C_11_H_11_O_3_ [M + H]^+^ 191.0703).

*3-Hydroxy-1-(thiophen-2-yl)pent-2-ene-1,4-dione* (**4b**). Yield 1.060 g (54%); beige powder; mp 84–86 °C; ^1^H NMR (500 MHz, CDCl_3_) δ 2.48 (s, 3H, Me), 6.77 (s, 1H, CH), 7.19 (dd, 1H, CH_Ar_, *J* = 4.9, 3.8 Hz), 7.73 (dd, *J* = 4.9, 1.1 Hz, 1H, CH_Ar_), 7.84 (dd, *J* = 3.8, 1.2 Hz, 1H, CH_Ar_), 14.70 (br. s, 1H, OH); ^13^C NMR (125 MHz, CDCl_3_) δ 25.4 (Me), 95.3 (CH), 128.7 (C^Ar^), 132.4 (C^Ar^), 134.9 (C^Ar^), 142.5 (C^Ar^), 168.7 (C^enol^), 186.4 (C=O), 197.0 (C=O); Anal. calcd. for C_9_H_8_O_3_S. C, 55.09; H, 4.11. Found: C, 55.16; H, 4.12. IR ν 3137–2877 (C–H, O–H), 1658, 1552 (C=O), 1505–1411 (C=C). HRMS *m/z* 197.0267 (calcd. for C_9_H_9_O_3_S [M + H]^+^ 197.0267).

*Ethyl 3(5)-acetyl-1H-pyrazole-5(3)-carboxylate* (**6**). Yield 0.421 g (77%, *method A*), 0.459 g (84%, *method B*); beige powder; mp 102 °C (hexane, litr. 92–94 °C [86]); ^1^H NMR (500 MHz, DMSO-*d*_6_) δ 1.31 (t, *J* = 7.1 Hz, 3H, OCH_2_CH_3_), 2.52 (s, 3H, Me), 4.32 (q, *J* = 7.1 Hz, 2H, OCH_2_CH_3_); (**A**) 7.16 (s, 1H, CH), 14.44 (s, 1H, NH), (**B**) 7.46 (s, 1H, CH), 14.60 (s, 1H, NH); ^13^C NMR (125 MHz, DMSO-*d*_6_) δ 14.1 (CH_3_^ester^), 26.7 (Me), 60.8 (CH_2_^ester^), (**A**) 108.4 (CH), 135.3 (C^pyr^), 143.6 (C^pyr^), 158.7 (C=O^ester^), 188.6 (C=O), (**B**) 111.5 (CH), 142.2 (C^pyr^), 151.5 (C^pyr^), 161.1 (C=O^ester^), 192.8 (C=O); Anal. calcd. for C_8_H_10_N_2_O_3_. C, 52.74; H, 5.53; N, 15.38. Found: C, 52.76; H, 5.56; N, 15.35. IR ν 3266 (N–H), 3130–2929 (C–H), 1707 (C=O_ester_), 1686 (C=O), 1560–1418 (C=C, C=N).

*Ethyl 5-acetyl-1-methyl-1H-pyrazole-3-carboxylate* (**7a**). Yield 0.383 g (65%); white powder; mp 99 °C; ^1^H NMR (500 MHz, CDCl_3_) δ 1.35 (t, *J* = 7.1 Hz, 3H, OCH_2_CH_3_), 2.53 (s, 3H, CH_3_), 4.12 (s, 3H, NMe), 4.29 (q, *J* = 7.1 Hz, 2H, OCH_2_CH_3_), 7.49 (s, 1H, CH); ^13^C NMR (125 MHz, CDCl_3_) δ 14.1 (CH_3_^ester^), 28.4 (Me), 40.6 (NMe), 60.5 (CH_2_^ester^), 114.6 (CH), 139.8 (C^pyr^), 140.7 (C^pyr^), 161.0 (C=O^ester^), 189.2 (C=O); Anal. calcd. for C_9_H_12_N_2_O_3_. C, 55.09; H, 6.16; N, 14.28. Found: C, 54.87; H, 6.18; N, 14.16. IR ν 3345–2900 (C–H), 1712 (C=O_ester_), 1679 (C=O), 1522–1411 (C=C, C=N). HRMS *m/z* 197.0912 (calcd. for C_9_H_13_N_2_O_3_ [M + H]^+^ 197.0921).

*Ethyl 5-acetyl-1-phenyl-1H-pyrazole-3-carboxylate* (**7b**). Yield 0.597 g (77%); yellow powder; mp 105 °C (litr. 112 °C [87]); ^1^H NMR (400 MHz, CDCl_3_) δ 1.42 (t, *J* = 7.1 Hz, 3H, OCH_2_CH_3_), 2.53 (s, 3H, CH_3_), 4.45 (q, *J* = 7.1 Hz, 2H, OCH_2_CH_3_), 7.37–7.40 (m, 2H, 2CH_Ar_), 7.45–7.46 (m, 3H, 3CH_Ar_), 7.50 (s, 1H, CH); ^13^C NMR (125 MHz, CDCl_3_) δ 14.3 (CH_3_^ester^), 28.7 (Me), 61.5 (CH_2_^ester^), 114.8 (CH), 126.0 (C^Ph^), 128.7 (C^Ph^), 129.3 (C^Ph^), 140.0 (C^Ph^), 140.8 (C^pyr^), 143.7 (C^pyr^), 161.5 (C=O^ester^), 187.1 (C=O); Anal. calcd. for C_14_H_14_N_2_O_3_. C, 65.11; H, 5.46; N, 10.85. Found: C, 65.02; H, 5.40; N, 10.89. IR ν 3350–2910 (C–H), 1712 (C=O_ester_), 1682 (C=O), 1560–1454 (C=C, C=N). HRMS *m/z* 259.1080 (calcd. for C_14_H_15_N_2_O_3_ [M + H]^+^ 259.1077).

*Ethyl 5-acetyl-1-(4-cyanophenyl)-1H-pyrazole-3-carboxylate* (**7c**). Yield 0.637 g (75%); white powder; mp 219 °C; ^1^H NMR (400 MHz, CDCl_3_) δ 1.43 (t, *J* = 7.1 Hz, 3H, OCH_2_CH_3_), 2.59 (s, 3H, Me), 4.46 (q, *J* = 7.1 Hz, 2H, OCH_2_CH_3_), 7.53–7.56 (m, 3H, CH and 2CH_Ar_), 7.75–7.78 (m, 2H, 2CH_Ar_); ^13^C NMR (125 MHz, CDCl_3_) δ 14.3 (CH_3_^ester^), 28.6 (Me), 61.8 (CH_2_^ester^), 113.0 (CCN), 115.6 (CH), 117.9 (CN), 126.9 (C^Ar^), 132.6 (C^Ar^), 140.7 (C^pyr^), 143.3 (C^Ar^), 144.7 (C^pyr^), 161.1 (C=O^ester^), 187.1 (C=O); Anal. calcd. for C_15_H_13_N_3_O_3_. C, 63.60; H, 4.63; N, 14.83. Found: C, 63.19; H, 4.57; N, 14.82. IR ν 3356, 3136, 3110–2950 (C–H), 2227 (C≡N), 1714 (C=O_ester_), 1684 (C=O), 1605–1426 (C=C, C=N). HRMS *m/z* 284.1032 (calcd. for C_15_H_14_N_3_O_3_ [M + H]^+^ 284.1030).

*Ethyl 5-acetyl-1-(4-fluorophenyl)-1H-pyrazole-3-carboxylate* (**7d**). Yield 0.572 g (69%); white powder; mp 148 °C; ^1^H NMR (400 MHz, CDCl_3_) δ 1.42 (t, *J* = 7.1 Hz, 3H, OCH_2_CH_3_), 2.54 (s, 3H, Me), 4.46 (q, *J* = 7.1 Hz, 2H, OCH_2_CH_3_), 7.11–7.17 (m, 2H, 2CH_Ar_), 7.34–7.40 (m, 2H, 2CH_Ar_), 7.50 (s, 1H, CH); ^13^C NMR (125 MHz, CDCl_3_) δ 14.3 (CH_3_^ester^), 28.6 (Me), 61.5 (CH_2_^ester^), 114.9 (CH), 115.7 (d, *J* = 23.3 Hz, C^ArF^), 128.0 (d, *J* = 8.8 Hz, C^ArF^), 136.2 (d, *J* = 3.3 Hz, C^ArF^), 140.7 (C^pyr^), 143.8 (C^pyr^), 161.4 (C=O^ester^), 162.8 (d, *J* = 249.5 Hz, CF), 187.1 (C=O); ^19^F NMR (376 MHz, CDCl_3_) δ 50.31 (tt, *J* = 8.2, 4.7 Hz, CF); Anal. calcd. for C_14_H_13_FN_2_O_3_. C, 60.87; H, 4.74; N, 10.14. Found: C, 60.85; H, 4.73; N, 10.13. IR ν 3354–2890 (C–H), 1715 (C=O_ester_), 1682 (C=O), 1608–1443 (C=C, C=N), 1158–1074 (C–F). HRMS *m/z* 277.0986 (calcd. for C_14_H_14_FN_2_O_3_ [M + H]^+^ 277.0983).

*Ethyl 5-acetyl-1-[4-(trifluoromethyl)phenyl]-1H-pyrazole-3-carboxylate* (**7e**). Yield 0.685 g (70%); white powder; mp 137 °C; ^1^H NMR (400 MHz, CDCl_3_) δ 1.43 (t, *J* = 7.1 Hz, 3H, OCH_2_CH_3_), 2.58 (s, 3H, Me), 4.46 (q, *J* = 7.1 Hz, 2H, OCH_2_CH_3_), 7.52–7.55 (m, 3H, CH and 2CH_Ar_), 7.73 (d, *J* = 8.3 Hz, 2H, 2CH_Ar_); ^13^C NMR (125 MHz, CDCl_3_) δ 14.3 (CH_3_^ester^), 28.6 (Me), 61.6 (CH_2_^ester^), 115.3 (CH), 123.6 (q, *J* = 272.3 Hz, CF_3_), 125.9 (q, *J* = 3.8 Hz, C^ArF^), 126.6 (C^ArF^); 131.3 (q, *J* = 32.9 Hz, CCF_3_), 140.7 (C^pyr^), 142.8 (C^ArF^), 144.4 (C^pyr^), 161.2 (C=O^ester^), 187.1 (C=O); ^19^F NMR (376 MHz, CDCl_3_) δ 99.08 (s, CF_3_); Anal. calcd. for C_15_H_13_F_3_N_2_O_3_. C, 55.22; H, 4.02; N, 8.59. Found: C, 55.05; H, 3.84; N, 8.55. IR ν 3347–2970 (C–H), 1705 (C=O_ester_), 1684 (C=O), 1589–1462 (C=C, C=N), 1163–1102 (C–F). HRMS *m/z* 327.0957 (calcd. for C_15_H_14_F_3_N_2_O_3_ [M + H]^+^ 327.0951).

*Ethyl 5-acetyl-1-(4-nitrophenyl)-1H-pyrazole-3-carboxylate* (**7f**). Yield 0.610 g (67%); beige powder; mp 193 °C; ^1^H NMR (400 MHz, CDCl_3_) δ 1.43 (t, *J* = 7.1 Hz, 3H, OCH_2_CH_3_), 2.60 (s, 3H, Me), 4.47 (q, *J* = 7.1 Hz, 2H, OCH_2_CH_3_), 7.56 (s, 1H, CH), 7.59–7.62 (m, 2H, 2CH_Ar_), 8.31–8.35 (m, 2H, 2CH_Ar_); ^13^C NMR (125 MHz, CDCl_3_) δ 14.3 (CH_3_^ester^), 28.6 (Me), 61.8 (CH_2_^ester^), 115.7 (CH), 124.1 (C^Ar^), 127.0 (C^Ar^), 140.8 (C^pyr^), 144.7 (C^pyr^), 144.8 (C^Ar^), 147.7 (CNO_2_), 161.0 (C=O^ester^), 187.1 (C=O); Anal. calcd. for C_14_H_13_N_3_O_5_. C, 55.45; H, 4.32; N, 13.86. Found: C, 55.38; H, 4.24; N, 13.82. IR ν 3359, 3133, 3089–2862 (C–H), 1714 (C=O_ester_), 1683 (C=O), 1597–1427 (C=C, C=N), 1527 (NO_2_). HRMS *m/z* 304.0927 (calcd. for C_14_H_14_N_3_O_5_ [M + H]^+^ 304.0928).

*Ethyl 5-acetyl-1-(4-sulfamoylphenyl)-1H-pyrazole-3-carboxylate* (**7g**). Yield 0.790 g (78%); beige powder; mp 199 °C; ^1^H NMR (500 MHz, DMSO-*d*_6_) δ 1.32 (t, *J* = 7.1 Hz, 3H, OCH_2_CH_3_), 2.60 (s, 3H, Me), 4.35 (q, *J* = 7.1 Hz, 2H, OCH_2_CH_3_), 7.55 (s, 2H, 2CH_Ar_), 7.68–7.71 (m, 2H, 2CH_Ar_), 7.85 (s, 1H, CH), 7.91–7.94 (m, 2H, NH_2_); ^13^C NMR (125 MHz, DMSO-*d*_6_) δ 14.2 (CH_3_^ester^), 28.7 (Me), 61.0 (CH_2_^ester^), 115.5 (CH), 126.2 (C^Ar^), 126.4 (C^Ar^), 141.2 (C^pyr^), 142.3 (C^Ar^), 143.5 (C^Ar^), 144.3 (C^pyr^), 160.8 (C=O^ester^), 188.2 (C=O); Anal. calcd. for C_14_H_15_N_3_O_5_S. C, 49.85; H, 4.48; N, 12.46. Found: C, 49.88; H, 4.54; N, 12.34. IR ν 3322–2873 (C–H, N–H), 1714 (C=O_ester_), 1683 (C=O), 1591–1409 (C=C, C=N). HRMS *m/z* 338.0803 (calcd. for C_14_H_16_N_3_O_5_S [M + H]^+^ 338.0805).

*Ethyl 5-acetyl-1-(2-fluorophenyl)-1H-pyrazole-3-carboxylate* (**7h**). Yield 0.547 g (66%); white powder; mp 95 °C; ^1^H NMR (400 MHz, CDCl_3_) δ 1.43 (t, *J* = 7.1 Hz, 3H, OCH_2_CH_3_), 2.55 (s, 3H, Me), 4.46 (q, *J* = 7.1 Hz, 2H, OCH_2_CH_3_), 7.16–7.29 (m, 2H, 2CH_Ar_), 7.42–7.49 (m, 1H, CH_Ar_), 7.49 (s, 1H, CH); ^13^C NMR (125 MHz, CDCl_3_) δ 14.3 (CH_3_^ester^), 28.2 (Me), 61.6 (CH_2_^ester^), 114.0 (CH), 116.0 (d, *J* = 19.5 Hz, C^ArF^), 124.4 (d, *J* = 3.8 Hz, C^ArF^), 128.1 (C^ArF^), 128.5 (d, *J* = 12.7 Hz, C^ArF^), 131.0 (d, *J* = 7.8 Hz, C^ArF^), 141.6 (C^pyr^), 144.5 (C^pyr^), 156.7 (d, *J* = 252.0 Hz, CF), 161.4 (C=O^ester^), 187.1 (C=O); ^19^F NMR (376 MHz, CDCl_3_) δ 39.09–39.15 (m, CF); Anal. calcd. for C_14_H_13_FN_2_O_3_. C, 60.87; H, 4.74; N, 10.14. Found: C, 60.86; H, 4.79; N, 10.08. IR ν 3343–2910 (C–H), 1713 (C=O_ester_), 1685 (C=O), 1598–1450 (C=C, C=N), 1152–1096 (C–F). HRMS *m/z* 277.0986 (calcd. for C_14_H_14_FN_2_O_3_ [M + H]^+^ 277.0983).

*Ethyl 5-acetyl-1-(2-chlorophenyl)-1H-pyrazole-3-carboxylate* (**7i**). Yield 0.711 g (81%); yellow powder; mp 94 °C; ^1^H NMR (500 MHz, CDCl_3_) δ 1.43 (t, *J* = 7.1 Hz, 3H, OCH_2_CH_3_), 2.53 (s, 3H, Me), 4.46 (q, *J* = 7.1 Hz, 2H, OCH_2_CH_3_), 7.36–7.49 (m, 4H, 4CH_Ar_), 7.50 (s, 1H, CH); ^13^C NMR (125 MHz, CDCl_3_) δ 14.3 (CH_3_^ester^), 28.0 (Me), 61.6 (CH_2_^ester^), 113.7 (CH), 127.4 (C^Ar^), 128.6 (C^Ar^), 129.8 (C^Ar^), 130.7 (C^Ar^), 131.5 (C^Ar^), 138.3 (C^Ar^), 141.8 (C^pyr^), 144.3 (C^pyr^), 161.4 (C=O^ester^), 187.0 (C=O); Anal. calcd. for C_14_H_13_ClN_2_O_3_. C, 57.45; H, 4.48; N, 9.57. Found: C, 57.37; H, 4.33; N, 9.69. IR ν 3368–2932 (C–H), 1715 (C=O_ester_), 1690 (C=O), 1593–1439 (C=C, C=N). HRMS *m/z* 293.0691 (calcd. for C_14_H_14_ClN_2_O_3_ [M + H]^+^ 293.0687).

*Ethyl 5-acetyl-1-(3-fluorophenyl)-1H-pyrazole-3-carboxylate* (**7j**). Yield 0.588 g (71%); white powder; mp 136 °C; ^1^H NMR (400 MHz, CDCl_3_) δ 1.42 (t, *J* = 7.1 Hz, 3H, OCH_2_CH_3_), 2.56 (s, 3H, Me), 4.46 (q, *J* = 7.1 Hz, 2H, OCH_2_CH_3_), 7.02–7.12 (m, 3H, 3CH_Ar_), 7.39–7.45 (m, 1H, CH_Ar_), 7.50 (s, 1H, CH); ^13^C NMR (125 MHz, CDCl_3_) δ 14.3 (CH_3_^ester^), 28.6 (Me), 61.6 (CH_2_^ester^), 114.0 (d, *J* = 25.2 Hz, C^ArF^), 115.0 (CH), 116.4 (d, *J* = 21.0 Hz, C^ArF^), 122.0 (d, *J* = 3.3 Hz, C^ArF^), 129.9 (d, *J* = 8.6 Hz, C^ArF^), 140.7 (C^pyr^), 141.1 (d, *J* = 10.0 Hz, C^ArF^), 144.0 (C^pyr^), 161.3 (C=O^ester^), 162.2 (d, *J* = 248.2 Hz, CF), 187.0 (C=O); ^19^F NMR (376 MHz, CDCl_3_) δ 50.14–50.20 (m, CF); Anal. calcd. for C_14_H_13_FN_2_O_3_. C, 60.87; H, 4.74; N, 10.14. Found: C, 60.74; H, 4.89; N, 9.93. IR ν 3230–2920 (C–H), 1714 (C=O_ester_), 1688 (C=O), 1590–1440 (C=C, C=N), 1161–1103 (C–F). HRMS *m/z* 277.0982 (calcd. for C_14_H_14_FN_2_O_3_ [M + H]^+^ 277.0983).

*Ethyl 5-acetyl-1-(3-chlorophenyl)-1H-pyrazole-3-carboxylate* (**7k**). Yield 0.641 g (73%), yellow powder; mp 131 °C; ^1^H NMR (500 MHz, CDCl_3_) δ 1.42 (t, *J* = 7.1 Hz, 3H, OCH_2_CH_3_), 2.56 (s, 3H, Me), 4.46 (q, *J* = 7.1 Hz, 2H, OCH_2_CH_3_), 7.27–7.29 (m, 1H, CH_Ar_), 7.39 (t, *J* = 7.9 Hz, 1H, CH_Ar_), 7.42–7.47 (m, 2H, 2CH_Ar_), 7.50 (s, 1H, CH); ^13^C NMR (125 MHz, CDCl_3_) δ 14.3 (CH_3_^ester^), 28.6 (Me), 61.6 (CH_2_^ester^), 115.0 (CH), 124.4 (C^Ar^), 126.5 (C^Ar^), 129.5 (C^Ar^), 129.6 (C^Ar^), 134.4 (C^Ar^), 140.7 (C^pyr^), 140.9 (C^Ar^), 144.1 (C^pyr^), 161.3 (C=O^ester^), 187.0 (C=O); Anal. calcd. for C_14_H_13_ClN_2_O_3_. C, 57.45; H, 4.48; N, 9.57. Found: C, 57.24; H, 4.38; N, 9.43. IR ν 3132, 3080–2938 (C–H), 1716 (C=O_ester_), 1689 (C=O), 1595–1418 (C=C, C=N). HRMS *m/z* 293.0690 (calcd. for C_14_H_14_ClN_2_O_3_ [M + H]^+^ 293.0687).

*Ethyl 5-acetyl-1-(3,5-difluorophenyl)-1H-pyrazole-3-carboxylate* (**7l**). Yield 0.697 g (79%); white powder; mp 173 °C; ^1^H NMR (400 MHz, CDCl_3_) δ 1.43 (t, *J* = 7.1 Hz, 3H, OCH_2_CH_3_), 2.58 (s, 3H, Me), 4.46 (q, *J* = 7.1 Hz, 2H, OCH_2_CH_3_), 6.90–7.02 (m, 3H, 3CH_Ar_), 7.50 (s, 1H, CH); ^13^C NMR (125 MHz, CDCl_3_) δ 14.3 (CH_3_^ester^), 28.6 (Me), 61.7 (CH_2_^ester^), 105.0 (t, *J* = 25.2 Hz, C^ArF^), 110.2 (d, *J* = 21.5 Hz, C^ArF^), 110.3 (d, *J* = 21.5 Hz, C^ArF^), 115.2 (CH), 140.7 (C^pyr^), 141.6 (t, *J* = 12.6 Hz, C^ArF^), 144.4 (C^pyr^), 161.1 (C=O^ester^), 162.3 (d, *J* = 250.0 Hz, CF), 162.4 (d, *J* = 250.0 Hz, CF), 186.9 (C=O); ^19^F NMR (376 MHz, CDCl_3_) δ 53.31–53.42 (m, 2CF); Anal. calcd. for C_14_H_12_F_2_N_2_O_3_. C, 57.15; H, 4.11; N, 9.52. Found: C, 57.15; H, 4.10; N, 9.48. IR ν 3250–2948 (C–H), 1717 (C=O_ester_), 1690 (C=O), 1593–1410 (C=C, C=N), 1162–1078 (C–F). HRMS *m/z* 295.0893 (calcd. for C_14_H_13_F_2_N_2_O_3_ [M + H]^+^ 295.0889).

*3-Methyl-6-(thiophen-2-yl)pyridazin-4(1H)-one* (**8**). Yield 0.456 g (79%, *obtained from* **2c**), 0.363 g (63%, *obtained from* **3**); brown powder; mp 208–210 °C (CHCl_3_); ^1^H NMR (400 MHz, DMSO-*d*_6_) δ 2.39 (s, 3H, Me), 7.23 (s, 1H, CH), 7.31 (dd, *J* = 5.0, 3.8 Hz, 1H, CH_Ar_), 7.95–7.98 (m, 2H, 2CH_Ar_); ^13^C NMR (125 MHz, DMSO-*d*_6_) δ 15.5 (Me), 110.0 (CH), 129.2 (C^Ar^), 130.2 (C^Ar^), 132.2 (C^Ar^), 133.4 (C^Ar^), 150.3 (C^pyr^), 153.4 (C^pyr^), 164.0 (C=O); Anal. calcd. for C_9_H_8_N_2_OS. C, 56.23; H, 4.19; N, 14.57. Found: C, 56.17; H, 4.25; N, 14.66. IR ν 3213–2700, 2554–2454 (C–H, N–H), 1607 (C=O), 1575–1421 (C=C, C=N). HRMS *m/z* 193.0432 (calcd. for C_9_H_9_N_2_OS [M + H]^+^ 193.0430).

*1,3-Dimethyl-6-(thiophen-2-yl)pyridazin-4(1H)-one* (**9a**). Yield 0.421 g (68%, *obtained from* **2c**); white powder; mp 170–172 °C (CHCl_3_–Et_2_O/1:1); ^1^H NMR (400 MHz, CDCl_3_) δ 2.36 (s, 3H, Me), 3.85 (s, 3H, NMe), 6.48 (s, 1H, CH), 7.16 (dd, *J* = 5.1, 3.6 Hz, 1H, CH_Ar_), 7.24 (dd, *J* = 3.6, 1.2 Hz, 1H, CH_Ar_), 7.55 (dd, *J* = 5.1, 1.2 Hz, 1H, CH_Ar_); ^13^C NMR (125 MHz, CDCl_3_) δ 16.9 (Me), 45.2 (NMe), 116.7 (CH), 127.7 (C^Ar^), 128.9 (C^Ar^), 129.6 (C^Ar^), 133.2 (C^Ar^), 147.5 (C^pyr^), 157.9 (C^pyr^), 170.6 (C=O); Anal. calcd. for C_10_H_10_N_2_OS. C, 58.23; H, 4.89; N, 13.58. Found: C, 57.96; H, 4.79; N, 13.43. IR ν 3221–2900 (C–H), 1613 (C=O), 1568–1429 (C=C, C=N). HRMS *m/z* 207.0589 (calcd. for C_10_H_11_N_2_OS [M + H]^+^ 207.0587).

*3-Methyl-1-phenyl-6-(thiophen-2-yl)pyridazin-4(1H)-one* (**9b**). Yield 0.596 g (74%, *obtained from* **2c**), 0.491 g (61%, *obtained from* **3**); brown powder; mp 190 °C (CHCl_3_); ^1^H NMR (500 MHz, CDCl_3_) δ 2.41 (s, 3H, Me), 6.66 (s, 1H, CH), 6.85 (dd, *J* = 3.7, 1.2 Hz, 1H, CH_Ar_), 6.91 (dd, *J* = 5.1, 3.7 Hz, 1H, CH_Ar_), 7.28 (dd, *J* = 7.0, 3.4 Hz, 2H, 2CH_Ar_), 7.36 (dd, *J* = 5.0, 1.2 Hz, 1H, CH_Ar_), 7.39 (dd, *J* = 7.0, 3.4 Hz, 3H, 3CH_Ar_); ^13^C NMR (125 MHz, DMSO-*d*_6_) δ 16.5 (Me), 114.4 (CH), 127.0 (C^Ph^), 127.4 (C^Ar^), 129.0 (C^Ar^), 129.1 (C^Ph^), 130.8 (C^Ar^), 130.8 (C^Ph^), 133.4 (C^Ar^), 143.0 (C^Ph^), 147.0 (C^pyr^), 156.2 (C^pyr^), 169.6 (C=O); Anal. calcd. for C_15_H_12_N_2_OS. C, 67.14; H, 4.51; N, 10.44. Found: C, 66.81; H, 4.39; N, 10.19. IR ν 3228–2930 (C–H), 1618 (C=O), 1590–1426 (C=C, C=N). HRMS *m/z* 269.0745 (calcd. for C_15_H_13_N_2_OS [M + H]^+^ 269.0743).

*6-(Tert-butyl)-3-methylpyridazin-4(1H)-one* (**10a**). Yield 0.279 g (56%); beige powder; mp 239–241 °C; ^1^H NMR (500 MHz, DMSO-*d*_6_) δ 1.27 (s, 9H, *t*-Bu), 2.11 (s, 3H, Me), 6.11 (s, 1H, CH), 12.60 (s, 1H, NH); ^13^C NMR (125 MHz, DMSO-*d*_6_) δ 16.4 (Me), 28.3 (CH_3_*^t^*^-Bu^), 33.8 (C*^t^*^-Bu^), 108.3 (CH), 154.8 (C^pyr^), 160.9 (C^pyr^), 170.9 (C=O); Anal. calcd. for C_9_H_14_N_2_O. C, 65.03; H, 8.49; N, 16.85. Found: C, 65.08; H, 8.52; N, 16.92. IR ν 3432–2760 (C–H, N–H), 1606 (C=O), 1579–1442 (C=C, C=N). HRMS *m/z* 167.1180 (calcd. for C_9_H_15_N_2_O [M + H]^+^ 167.1179).

*1-(3(5)-(Tert-butyl)-1H-pyrazol-5(3)-yl)ethan-1-one* (**10b**). Yield 0.409 g (82%, *method B*), 0.464 g (93%, *method C*), 0.374 g (75%, *method D*); white powder; mp 123 °C (hexane); ^1^H NMR (500 MHz, DMSO-*d*_6_) δ 1.30 (s, 9H, *t*-Bu), 2.43 (s, 3H, Me), 6.34 (s, 1H, CH); ^13^C NMR (125 MHz, DMSO-*d*_6_) δ 26.1 (Me), 29.9 (CH_3_*^t^*^-Bu^), 30.6 (C*^t^*^-Bu^), 100.6 (CH), 150.8 (C^pyr^), 154.4 (C^pyr^), 193.6 (C=O); Anal. calcd. for C_9_H_14_N_2_O. C, 65.03; H, 8.49; N, 16.85. Found: C, 65.12; H, 8.61; N, 16.72. IR ν 3480–2876 (C–H, N–H), 1671 (C=O), 1563–1420 (C=C, C=N). HRMS *m/z* 167.1179 (calcd. for C_9_H_15_N_2_O [M + H]^+^ 167.1179).

*6-(Tert-butyl)-1,3-dimethylpyridazin-4(1H)-one* (**11**). Yield 0.411 g (76%); white powder; mp 210 °C; ^1^H NMR (400 MHz, DMSO-*d*_6_) δ 1.38 (s, 9H, *t*-Bu), 2.10 (s, 3H, Me), 3.97 (s, 3H, NMe), 6.25 (s, 1H, CH); ^13^C NMR (125 MHz, DMSO-*d*_6_) δ 16.5 (Me), 29.5 (CH_3_*^t^*^-Bu^), 35.4 (C*^t^*^-Bu^), 47.4 (NMe), 112.0 (CH), 155.0 (C^pyr^), 161.5 (C^pyr^), 170.5 (C=O); Anal. calcd. for C_10_H_16_N_2_O. C, 66.63; H, 8.95; N, 15.54. Found: C, 66.65; H, 8.92; N, 15.50. IR ν 3417–3072, 2971–2915 (C–H), 1606 (C=O), 1570–1500 (C=C, C=N). HRMS *m/z* 181.1335 (calcd. for C_10_H_17_N_2_O [M + H]^+^ 181.1335).

*1-(5-(Tert-butyl)-1-phenyl-1H-pyrazol-3-yl)ethan-1-one* (**12**). Yield 0.589 g (81%); white powder; mp 102–103 °C; ^1^H NMR (400 MHz, DMSO-*d*_6_) δ 1.17 (s, 9H, *t*-Bu), 2.42 (s, 3H, Me), 6.59 (s, 1H, CH), 7.38–7.45 (m, 2H, 2CH_Ar_), 7.49–7.62 (m, 3H, 3CH_Ar_); ^13^C NMR (125 MHz, DMSO-*d*_6_) δ 26.1 (Me), 30.2 (CH_3_*^t^*^-Bu^), 31.7 (C*^t^*^-Bu^), 103.6 (CH), 128.4 (C^Ph^), 128.9 (C^Ph^), 129.8 (C^Ph^), 141.2 (C^Ph^), 149.5 (C^pyr^), 155.0 (C^pyr^), 193.3 (C=O); Anal. calcd. for C_15_H_18_N_2_O. C, 74.35; H, 7.49; N, 11.56. Found: C, 74.50; H, 7.60; N, 11.44. IR ν 3524–2986 (C–H), 1688 (C=O), 1540–1408 (C=C, C=N). HRMS *m/z* 243.1488 (calcd. for C_15_H_19_N_2_O [M + H]^+^ 243.1492).

*3-Methyl-6-phenylpyridazin-4(1H)-one* (**13a**). Yield 0.330 g (59%); beige powder; mp 283–284 °C; ^1^H NMR (400 MHz, DMSO-*d*_6_) δ 2.19 (s, 3H, Me), 6.51 (s, 1H, CH), 7.48–7.62 (m, 3H, 3CH_Ar_), 7.73–7.76 (m, 2H, 2CH_Ar_), 3.98 (br. s, 1H, NH); ^13^C NMR (125 MHz, DMSO-*d*_6_) δ 16.6 (Me), 110.2 (CH), 127.1 (C^Ph^), 129.1 (C^Ph^), 130.8 (C^Ph^), 131.3 (C^Ph^), 151.0 (C^pyr^), 156.2 (C^pyr^), 170.7 (C=O); Anal. calcd. for C_11_H_10_N_2_O. C, 70.95; H, 5.41; N, 15.04. Found: C, 70.83; H, 5.31; N, 14.95. IR ν 3120–2732 (C–H, N–H), 1610 (C=O), 1584–1433 (C=C, C=N). HRMS *m/z* 187.0866 (calcd. for C_11_H_11_N_2_O [M + H]^+^ 187.0866).

*1-(3(5)-Phenyl-1H-pyrazol-5(3)-yl)ethan-1-one* (**13b**). Yield 0.296 g (53%, *method B*), 0.536 g (96%, *method C*); beige powder; mp 155 °C (EtOAc); ^1^H NMR (500 MHz, DMSO-*d*_6_) δ 2.53 (s, 3H, Me), 7.02 (s, 1H, CH), 7.38–7.41 (m, 1H, CH_Ar_), 7.44–7.50 (m, 2H, 2CH_Ar_), 7.81–7.87 (m, 2H, 2CH_Ar_), 13.89 (br. s, 1H, NH); ^13^C NMR (125 MHz, DMSO-*d*_6_) δ (**A**) 26.2 (Me), 102.2 (CH), 125.4 (C^Ph^), 129.0 (C^Ph^), 128.5 (C^Ph^), 128.5 (C^Ph^), 143.9 (C^pyr^), 152.1 (C^pyr^), 193.4 (C=O); (**B**) 27.2 (Me), 106.2 (CH), 125.1 (C^Ph^), 128.7 (C^Ph^), 127.8 (C^Ph^), 132.8 (C^Ph^), 142.3 (C^pyr^), 151.1 (C^pyr^), 188.5 (C=O); Anal. calcd. for C_11_H_10_N_2_O. C, 70.95; H, 5.41; N, 15.04. Found: C, 70.87; H, 5.52; N, 15.24. IR ν 3227 (N–H), 3177–2851 (C–H), 1657 (C=O), 1611–1427 (C=C, C=N). HRMS *m/z* 187.0878 (calcd. for C_11_H_11_N_2_O [M + H]^+^ 187.0866).

*3-Methyl-1,6-diphenylpyridazin-4(1H)-one* (**14**). Yield 0.653 g (83%); beige powder; mp 224 °C; ^1^H NMR (400 MHz, DMSO-*d*_6_) δ 2.26 (s, 3H, Me), 6.30 (s, 1H, CH), 7.18–7.23 (m, 4H, 4CH_Ar_), 7.25–7.33 (m, 6H, 6CH_Ar_); ^13^C NMR (125 MHz, DMSO-*d*_6_) δ 16.5 (Me), 114.7 (CH), 126.8 (C^Ph^), 128.2 (C^Ph^), 128.3 (C^Ph^), 128.8 (C^Ph^), 128.8 (C^Ph^), 129.4 (C^Ph^), 133.5 (C^Ph^), 143.1 (C^Ph^), 153.3 (C^pyr^), 156.4 (C^pyr^), 169.8 (C=O); Anal. calcd. for C_17_H_14_N_2_O. C, 77.84; H, 5.38; N, 10.68. Found: C, 77.85; H, 5.40; N, 10.63. IR ν 3064–2921 (C–H), 1622 (C=O), 1591–1421 (C=C, C=N). HRMS *m/z* 263.1179 (calcd. for C_17_H_15_N_2_O [M + H]^+^ 263.1179).

*1,3-Dimethyl-6-phenylpyridazin-4(1H)-one* (**15a**). Yield 0.366 g (61%); beige powder; mp 125 °C; ^1^H NMR (400 MHz, DMSO-*d*_6_) δ 2.20 (s, 3H, Me), 3.62 (s, 3H, NMe), 6.13 (s, 1H, CH), 7.52–7.57 (m, 5H, Ph); ^13^C NMR (125 MHz, DMSO-*d*_6_) δ 16.5 (Me), 44.7 (NMe), 114.3 (CH), 128.3 (C^Ph^), 128.8 (C^Ph^), 129.9 (C^Ph^), 133.3 (C^Ph^), 153.8 (C^pyr^), 156.6 (C^pyr^), 169.3 (C=O); Anal. calcd. for C_12_H_12_N_2_O. C, 71.98; H, 6.04; N, 13.99. Found: C, 71.92; H, 6.00; N, 14.10. IR ν 3100–2918 (C–H), 1619 (C=O), 1573–1411 (C=C, C=N). HRMS *m/z* 201.1024 (calcd. for C_12_H_13_N_2_O [M + H]^+^ 201.1022).

*1-(1-Methyl-5-phenyl-1H-pyrazol-3-yl)ethan-1-one* (**15b**). Yield 0.144 g (24%); white powder; mp 88 °C; ^1^H NMR (400 MHz, DMSO-*d*_6_) δ 2.49 (s, 3H, Me), 3.94 (s, 3H, NMe), 6.85 (s, 1H, CH), 7.46–7.62 (m, 5H, Ph); ^13^C NMR (125 MHz, DMSO-*d*_6_) δ 26.2 (Me), 38.3 (NMe), 105.9 (CH), 128.6 (C^Ph^), 128.9 (C^Ph^), 128.9 (C^Ph^), 129.2 (C^Ph^), 145.0 (C^pyr^), 149.4 (C^pyr^), 193.0 (C=O); Anal. calcd. for C_12_H_12_N_2_O. C, 71.98; H, 6.04; N, 13.99. Found: C, 72.02; H, 6.05; N, 13.95. IR ν 3265–2940 (C–H), 1673 (C=O), 1593–1417 (C=C, C=N). HRMS *m/z* 201.1021 (calcd. for C_12_H_13_N_2_O [M + H]^+^ 201.1022).

*1-(3,5-Dihydroxy-5-phenylpyrazolidin-3-yl)ethan-1-one* (**16**). Yellow powder; mp 255–257 °C; ^1^H NMR (400 MHz, DMSO-*d*_6_) δ 2.33 (s, 3H, Me), 2.38 (s, 1H, CH_2_), 2.39 (s, 1H, CH_2_), 7.15–7.16 (m, 1H, NH), 7.22–7.26 (m, 1H, NH), 7.31–7.40 (m, 2H, H-A^Ph^, H-B^Ph^), 7.42–7.50 (m, 4H, 2H-A^Ph^, 2H-B^Ph^), 7.82–7.89 (m, 4H, 2H-A^Ph^, 2H-B^Ph^), 13.45–13.55 (m, 2H, 2OH). IR ν 3359–2889, (C–H, N–H, O–H), 1613 (C=O).

*1-(5(3))-(Thiophen-2-yl)-1H-pyrazol-3(5)-yl)ethan-1-one* (**17**). Yield 0.398 g (69%, *method B*), 0.542 g (94%, *method C*); beige powder; mp 118 °C (EtOAc); ^1^H NMR (500 MHz, CDCl_3_) δ 2.60 (s, 3H, Me), 6.98 (s, 1H, CH), 7.10 (dd, *J* = 5.1, 3.6 Hz, 1H, CH_Ar_), 7.33 (dd, *J* = 5.1, 1.2 Hz, 1H, CH_Ar_), 7.38 (dd, *J* = 3.6, 1.1 Hz, 1H, CH_Ar_), 11.16 (br. s, 1H, NH); ^13^C NMR (125 MHz, CDCl_3_) δ (**A**) 27.2 (Me), 105.8 (CH), 124.3 (C^Ar^), 125.3 (C^Ar^), 127.8 (C^Ar^), 135.8 (C^Ar^), 142.3 (C^pyr^), 146.8 (C^pyr^), 188.5 (C=O); (**B**) 26.2 (Me), 102.2 (CH), 125.5 (C^Ar^), 126.8 (C^Ar^), 128.2 (C^Ar^), 130.3 (C^Ar^), 138.4 (C^pyr^), 152.0 (C^pyr^), 193.3 (C=O); Anal. calcd. for C_9_H_8_N_2_OS. C, 56.23; H, 4.19; N, 14.57. Found: C, 56.07; H, 4.00; N, 14.44. IR ν 3190–2914 (C–H, N–H), 1668 (C=O), 1587–1428 (C=C, C=N). HRMS *m/z* 193.0431 (calcd. for C_9_H_9_N_2_OS [M + H]^+^ 193.0430).

*3-(Tert-butyl)-5-(1,1-dimethoxyethyl)-1H-pyrazole* (**18a**). Yield 0.945 g (89%); white powder; mp 87–88 °C; ^1^H NMR (400 MHz, DMSO-*d*_6_) δ 1.26 (s, 9H, *t*-Bu), 1.52 (s, 3H, Me), 3.07 (s, 6H, 2MeO), 5.92 (s, 1H, CH), 12.24 (br. s, 1H, NH); ^13^C NMR (125 MHz, DMSO-*d*_6_) δ 23.8 (Me), 30.2 (CH_3_*^t^*^-Bu^), 31.6 (C*^t^*^-Bu^), 48.2 (MeO), 98.0 (CH), 99.3 (C^acetal^), 152.0 (C^pyr^), 160.0 (C^pyr^); Anal. calcd. for C_11_H_20_N_2_O_2_. C, 62.24; H, 9.50; N, 13.20. Found: C, 62.20; H, 9.58; N, 13.21. IR ν 3470–2889 (C–H, N–H), 1682–1436 (C=C, C=N), 1141, 1111 (C–O). HRMS *m/z* 213.1593 (calcd. for C_11_H_21_N_2_O_2_ [M + H]^+^ 213.1598).

*5-(1,1-Dimethoxyethyl)-3-phenyl-1H-pyrazole* (**18b**). Yield 1.068 g (92%); white powder; mp 100–101 °C; ^1^H NMR (400 MHz, CDCl_3_) δ 1.68 (s, 3H, Me), 3.24 (s, 6H, 2MeO), 6.55 (s, 1H, CH), 7.30–7.35 (m, 1H, CH_Ar_), 7.39–7.47 (m, 2H, 2CH_Ar_), 7.79 (d, *J* = 7.6 Hz, 2H, 2CH_Ar_), 10.41 (br. s, 1H, NH); ^13^C NMR (125 MHz, CDCl_3_) δ 24.7 (Me), 49.1 (MeO), 98.6 (CH), 100.1 (C^acetal^), 125.6 (C^Ph^), 127.9 (C^Ph^), 128.7 (C^Ph^), 133.0 (C^Ph^), 146.2 (C^pyr^), 152.4 (C^pyr^); Anal. calcd. for C_13_H_16_N_2_O_2_. C, 67.20; H, 6.94; N, 12.06. Found: C, 67.24; H, 7.16; N, 12.03. IR ν 3010–2870 (C–H, N–H), 1680–1387 (C=C, C=N), 1144, 1112 (C–O). HRMS *m/z* 255.1106 (calcd. for C_13_H_16_N_2_O_2_Na [M + Na]^+^ 255.1104).

*3-(1,1-Dimethoxyethyl)-5-(thiophen-2-yl)-1H-pyrazole* (**18c**). Yield 1.072 g (90%); white powder; mp 96–98 °C; ^1^H NMR (500 MHz, CDCl_3_) δ 1.65 (s, 3H, Me), 3.23 (s, 6H, 2MeO), 6.44 (s, 1H, CH), 7.06 (dd, *J* = 5.1, 3.6 Hz, 1H, CH_Ar_), 7.25 (dd, *J* = 5.1, 1.1 Hz, 1H, CH_Ar_), 7.32 (dd, *J* = 3.6, 1.1 Hz, 1H, CH_Ar_), 10.21 (br. s, 1H, NH); ^13^C NMR (125 MHz, CDCl_3_) δ 24.7 (Me), 49.1 (MeO), 98.4 (CH), 100.1 (C^acetal^), 123.8 (C^Ar^), 124.6 (C^Ar^), 127.4 (C^Ar^), 136.4 (C^Ar^), 146.1 (C^pyr^), 1C was not observed; Anal. calcd. for C_11_H_14_N_2_O_2_S. C, 55.44; H, 5.92; N, 11.76. Found: C, 54.48; H, 5.45; N, 11.86. IR ν 3218 (N–H), 3111–2939 (C–H), 1682, 1598–1435 (C=C, C=N), 1147, 1109 (C–O). HRMS *m/z* 261.0671 (calcd. for C_11_H_14_N_2_O_2_SNa [M + Na]^+^ 261.0668).

*3-(Tert-butyl)-5-(1,1-dimethoxyethyl)-1-methyl-1H-pyrazole* (**19a**). Yield 0.928 g (82%); white crystals; mp 104–105 °C; ^1^H NMR (500 MHz, CDCl_3_) δ 1.28 (s, 9H, *t*-Bu), 1.59 (s, 3H, Me), 3.20 (s, 6H, 2MeO), 3.87 (s, 3H, NMe), 6.12 (s, 1H, CH); ^13^C NMR (125 MHz, CDCl_3_) δ 23.6 (Me), 30.6 (CH_3_*^t^*^-Bu^), 31.9 (C*^t^*^-Bu^), 37.4 (NMe), 48.9 (MeO), 99.0 (CH), 102.6 (C^acetal^), 143.2 (C^pyr^), 160.3 (C^pyr^); Anal. calcd. for C_12_H_22_N_2_O_2_. C, 63.69; H, 9.80; N, 12.38. Found: C, 63.71; H, 9.77; N, 13.44. IR ν 3431–2890 (C–H), 1568–1416 (C=C, C=N), 1146, 1110 (C–O). HRMS *m/z* 227.1755 (calcd. for C_12_H_23_N_2_O_2_ [M + H]^+^ 227.1754).

*5-(1,1-Dimethoxyethyl)-1-methyl-3-phenyl-1H-pyrazole* (**19b**). Yield 1.071 g (87%); yellow crystals; mp 114–115 °C; ^1^H NMR (500 MHz, CDCl_3_) δ 1.64 (s, 3H, Me), 3.24 (s, 6H, 2MeO), 3.99 (s, 3H, NMe), 6.61 (s, 1H, CH), 7.27–7.29 (m, 1H, CH_Ar_), 7.36–7.39 (m, 2H, 2CH_Ar_), 7.77–7.79 (m, 2H, 2CH_Ar_); ^13^C NMR (125 MHz, CDCl_3_) δ 23.5 (Me), 37.9 (NMe), 48.9 (MeO), 98.9 (CH), 103.6 (C^acetal^), 125.3 (C^Ph^), 127.4 (C^Ph^), 128.5 (C^Ph^), 133.4 (C^Ph^), 144.7 (C^pyr^), 149.6 (C^pyr^); Anal. calcd. for C_14_H_18_N_2_O_2_. C, 68.27; H, 7.37; N, 11.37. Found: C, 68.26; H, 7.36; N, 11.34. IR ν 2956–2834 (C–H), 1541–1430 (C=C, C=N), 1145, 1108 (C–O). HRMS *m/z* 247.1442 (calcd. for C_14_H_19_N_2_O_2_ [M + H]^+^ 247.1441).

*5-(1,1-Dimethoxyethyl)-1-methyl-3-(thiophen-2-yl)-1H-pyrazole* (**19c**). Yield 1.072 g (85%); white powder; mp 101–102 °C; ^1^H NMR (400 MHz, DMSO-*d*_6_) δ 1.57 (s, 3H, Me), 3.14 (s, 6H, 2MeO), 3.85 (s, 3H, NMe), 6.58 (s, 1H, CH), 7.05 (dd, *J* = 5.1, 3.5 Hz, 1H, CH_Ar_), 7.39 (dd, *J* = 3.7, 1.4 Hz, 1H, CH), 7.42 (dd, *J* = 5.1, 1.2 Hz, 1H, CH_Ar_); ^13^C NMR (125 MHz, DMSO-*d*_6_) δ 23.1 (Me), 37.6 (NMe), 48.5 (MeO), 98.4 (CH), 103.0 (C^acetal^), 123.7 (C^Ar^), 124.6 (C^Ar^), 127.6 (C^Ar^), 136.3 (C^Ar^), 144.0 (C^pyr^), 144.5 (C^pyr^); Anal. calcd. for C_12_H_16_N_2_O_2_S. C, 57.12; H, 6.39; N, 11.10. Found: C, 57.02; H, 6.47; N, 11.21. IR ν 3117–2911 (C–H), 1581–1435 (C=C, C=N), 1142, 1109 (C–O). HRMS *m/z* 253.1005 (calcd. for C_12_H_17_N_2_O_2_S [M + H]^+^ 253.1005).

*1-(3-(Tert-butyl)-1-methyl-1H-pyrazol-5-yl)ethan-1-one* (**20a**). Yield 0.481 g (89%); white powder; mp 59–60 °C; ^1^H NMR (400 MHz, DMSO-*d*_6_) δ 1.26 (s, 9H, *t*-Bu), 2.47 (s, 3H, Me), 3.97 (s, 3H, NMe), 6.98 (s, 1H, CH); ^13^C NMR (125 MHz, DMSO-*d*_6_) δ 28.3 (Me), 30.3 (CH_3_*^t^*^-Bu^), 31.6 (C*^t^*^-Bu^), 108.6 (CH), 138.8 (C^pyr^), 159.1 (C^pyr^), 189.2 (C=O), 1C was not observed; Anal. calcd. for C_10_H_16_N_2_O. C, 66.63; H, 8.95; N, 15.54. Found: C, 66.60; H, 8.92; N, 15.61. IR ν 3460–2920 (C–H), 1681 (C=O), 1564–1417 (C=C, C=N). HRMS *m/z* 181.1335 (calcd. for C_10_H_17_N_2_O [M + H]^+^ 181.1335).

*1-(1-Methyl-3-phenyl-1H-pyrazol-5-yl)ethan-1-one* (**20b**). Yield 0.559 g (93%); white powder; mp 59–60 °C; ^1^H NMR (400 MHz, DMSO-*d*_6_) δ 2.56 (s, 3H, Me), 4.09 (s, 3H, NMe), 7.32–7.36 (m, 1H, CH_Ar_), 7.42–7.46 (m, 2H, 2CH_Ar_), 7.64 (s, 1H, CH), 7.84–7.86 (m, 2H, 2CH_Ar_); ^13^C NMR (125 MHz, DMSO-*d*_6_) δ 28.4 (Me), 39.8 (NMe), 109.3 (CH), 125.0 (C^Ph^), 127.9 (C^Ph^), 128.8 (C^Ph^), 132.2 (C^Ph^), 139.9 (C^pyr^), 148.4 (C^pyr^), 189.2 (C=O); Anal. calcd. for C_12_H_12_N_2_O. C, 71.98; H, 6.04; N, 13.99. Found: C, 71.88; H, 6.10; N, 13.76. IR ν 3333–2947 (C–H), 1674 (C=O), 1605–1409 (C=C, C=N). HRMS *m/z* 201.1024 (calcd. for C_12_H_13_N_2_O [M + H]^+^ 201.1022).

*1-(1-Methyl-3-(thiophen-2-yl)-1H-pyrazol-5-yl)ethan-1-one* (**20c**). Yield 0.545 g (88%); white powder; mp 136–137 °C; ^1^H NMR (400 MHz, DMSO-*d*_6_) δ 2.54 (s, 3H, Me), 4.05 (s, 3H, NMe), 7.12 (dd, *J* = 5.1, 3.5 Hz, 1H, CH_Ar_), 7.47 (dd, *J* = 3.6, 1.2 Hz, 1H, CH_Ar_), 7.50 (s, 1H, CH), 7.51 (dd, *J* = 5.1, 1.2 Hz, 1H, CH_Ar_); ^13^C NMR (125 MHz, DMSO-*d*_6_) δ 28.4 (Me), 39.6 (NMe), 108.8 (CH), 124.3 (C^Ar^), 125.4 (C^Ar^), 127.8 (C^Ar^), 135.1 (C^Ar^), 139.8 (C^pyr^), 144.2 (C^pyr^), 189.2 (C=O); Anal. calcd. for C_10_H_10_N_2_OS. C, 58.23; H, 4.89; N, 13.58. Found: C, 58.29; H, 5.02; N, 13.43. IR ν 3200–2940 (C–H), 1673 (C=O), 1595–1426 (C=C, C=N). HRMS *m/z* 207.0588 (calcd. for C_10_H_11_N_2_OS [M + H]^+^ 207.0587).

*Methyl 3-acetyl-5-(tert-butyl)-1H-pyrazole-1-carboxylate* (**21a**). Yield 0.942 g (84%); white powder; mp 95–96 °C (hexane); ^1^H NMR (500 MHz, DMSO-*d*_6_) δ 1.44 (s, 9H, *t*-Bu), 2.52 (s, 3H, Me), 4.07 (s, 3H, OMe), 6.62 (s, 1H, CH); ^13^C NMR (125 MHz, DMSO-*d*_6_) δ 26.2 (Me), 29.0 (CH_3_*^t^*^-Bu^), 32.8 (C*^t^*^-Bu^), 55.5 (MeO), 106.6 (CH), 150.7 (C^pyr^), 151.1 (C^pyr^), 158.1 (C=O^ester^), 193.3 (C=O); Anal. calcd. for C_11_H_16_N_2_O_3_. C, 58.91; H, 7.19; N, 12.49. Found: C, 58.98; H, 7.16; N, 12.36. IR ν 3516–2875 (C–H), 1770 (C=O_ester_), 1689 (C=O), 1549–1423 (C=C, C=N). HRMS *m/z* 225.1239 (calcd. for C_10_H_17_N_2_O_3_ [M + H]^+^ 225.1234).

*Methyl 3-acetyl-5-phenyl-1H-pyrazole-1-carboxylate* (**21b**). Yield 1.050 g (86%); white powder; mp 149–150 °C; ^1^H NMR (400 MHz, CDCl_3_) δ 2.69 (s, 3H, Me), 3.98 (s, 3H, OMe), 6.84 (s, 1H, CH), 7.38–7.40 (m, 2H, 2CH_Ar_), 7.43–7.45 (m, 3H, 3CH_Ar_); ^13^C NMR (125 MHz, CDCl_3_) δ 26.5 (Me), 55.2 (MeO), 110.1 (CH), 128.1 (C^Ph^), 129.0 (C^Ph^), 129.3 (C^Ph^), 130.0 (C^Ph^), 148.7 (C^pyr^), 150.0 (C^pyr^), 153.1 (C=O^ester^), 194.0 (C=O); Anal. calcd. for C_13_H_12_N_2_O_3_. C, 63.93; H, 4.95; N, 11.47. Found: C, 63.43; H, 4.78; N, 11.21. IR ν 3244–2860 (C–H), 1767 (C=O_ester_), 1684 (C=O), 1510–1402 (C=C, C=N). HRMS *m/z* 245.0920 (calcd. for C_13_H_13_N_2_O_3_ [M + H]^+^ 245.0921).

*Methyl 3-acetyl-5-(thiophen-2-yl)-1H-pyrazole-1-carboxylate* (**21c**). Yield 1.051 g (84%); white powder; mp 139–140 °C; ^1^H NMR (500 MHz, CDCl_3_) δ 2.67 (s, 3H, Me), 4.05 (s, 3H, OMe), 6.95 (s, 1H, CH), 7.10 (dd, *J* = 5.1, 3.6 Hz, 1H, CH_Ar_), 7.34 (dd, *J* = 3.7, 1.2 Hz, 1H, CH_Ar_), 7.47 (dd, *J* = 5.1, 1.2 Hz, 1H, CH_Ar_); ^13^C NMR (125 MHz, CDCl_3_) δ 26.4 (Me), 55.3 (MeO), 111.1 (CH), 127.1 (C^Ar^), 128.2 (C^Ar^), 129.3 (C^Ar^), 129.9 (C^Ar^), 141.8 (C^pyr^), 150.0 (C^pyr^), 152.8 (C=O^ester^), 193.7 (C=O); Anal. calcd. for C_11_H_10_N_2_O_3_S. C, 52.79; H, 4.03; N, 11.19. Found: C, 52.46; H, 3.81; N, 11.21. IR ν 3212–2910 (C–H), 1769 (C=O_ester_), 1687 (C=O), 1570–1430 (C=C, C=N). HRMS *m/z* 251.0484 (calcd. for C_11_H_11_N_2_O_3_S [M + H]^+^ 251.0485).

*Ethyl 6-methyl-5-oxo-2,5-dihydropyridazine-3-carboxylate* (**22**). Yield 0.692 g (76%); beige powder; mp 163–165 °C (EtOAc); ^1^H NMR (500 MHz, DMSO-*d*_6_) δ 1.38 (t, *J* = 7.0 Hz, 3H, OCH_2_CH_3_), 2.19 (s, 3H, Me), 4.39 (q, *J* = 7.1 Hz, 2H, OCH_2_CH_3_), 6.62 (s, 1H, CH), 13.44 (s, 1H, NH); ^13^C NMR (125 MHz, DMSO-*d*_6_) δ 13.8 (CH_3_^ester^), 16.8 (Me), 62.7 (CH_2_^ester^), 112.6 (CH), 140.0 (C^pyr^), 158.4 (C^pyr^), 160.5 (C=O^ester^), 170.9 (C=O); Anal. calcd. for C_8_H_10_N_2_O_3_. C, 52.74; H, 5.53; N, 15.38. Found: C, 53.00; H, 5.78; N, 15.53. IR ν 3457–3200, 3073–2696 (C–H, N–H), 1737 (C=O_ester_), 1707 (C=O), 1576–1435 (C=C, C=N). HRMS *m/z* 183.0765 (calcd. for C_8_H_11_N_2_O_3_ [M + H]^+^ 183.0764).

### 3.4. XRD Experiments

The X-ray diffraction data of **2c**, **3**, **7a**, **7l**, **9a**, **9b**, **12**, **14**, **15b**, and **21b** were collected on an Xcalibur 3 CCDC diffractometer (Mo-K_α_, λ = 0.71073 Å, graphite monochromator, ω/2θ-scanning technique) [88]. The structures were solved by direct methods and refined by the full-matrix least squares in the anisotropic approximation for non-hydrogen atoms. The calculations were carried out by the SHELX-2014/2018 program package [89] using Olex2 1.2/1.3 [90]. The X-ray CIF files have been deposited at the Cambridge Crystallographic Data Center, allocated with the deposition numbers CCDC 2271046 (**2c**), CCDC 2271047 (**3**), CCDC 2271048 (**5**), CCDC 2271049 (**7a**), CCDC 2271050 (**7l**), CCDC 2271051 (**9a**), CCDC 2271052 (**9b**), CCDC 2271053 (**12**), CCDC 2271054 (**14**), CCDC 2271055 (**15b**), and CCDC 2271056 (**21b**). These data can be obtained free of charge from The Cambridge Crystallographic Data Center via www.ccdc.cam.ac.uk/data_request/cif, accessed on 1 August 2023.

*Crystal data for* **2c**: C_11_H_14_O_4_S (M = 242.28): tetragonal, space group P4_3_2_1_2, a = b = 11.1930(6) Å, c = 19.785(3) Å, V = 2478.8(4) Å^3^, Z = 8, μ = 0.257 mm^–1^, D_calc_ = 1.298 g/cm^3^, 6623 reflections measured to (7.17° ≤ 2Θ ≤ 56.53°), 3046 unique (R_int_ = 0.0538, R_sigma_ = 0.0705), which were used in all calculations. The final R_1_ was 0.0770 (I > 2σ(I)) and wR_2_ was 0.2588 (all data).

*Crystal data for* **3**: C_10_H_10_O_3_S (M = 210.24): monoclinic, space group P2_1_/c, a = 11.1721(12) Å, b = 9.9883(8) Å, c = 18.6591(16) Å, β = 91.751(8)^o^, V = 2081.2(3) Å^3^, Z = 8, μ = 0.289 mm^–1^, D_calc_ = 1.342 g/cm^3^, 11,131 reflections measured to (7.72° ≤ 2Θ ≤ 56.56°), 5033 unique (R_int_ = 0.0609, R_sigma_ = 0.0707), which were used in all calculations. The final R_1_ was 0.0635 (I > 2σ(I)) and wR_2_ was 0.2244 (all data).

*Crystal data for* **5**: C_11_H_10_O_3_S (M = 190.19): triclinic, space group P-1, a = 10.4213(6) Å, b = 15.8394(9) Å, c = 18.2990(12) Å, α = 85.814(5)°, β = 84.801(5)°, γ = 78.314(5)°, V = 2941.2(3) Å^3^, Z = 12, μ = 0.094 mm^–1^, D_calc_ = 1.289 g/cm^3^, 27,630 reflections measured to (6.97° ≤ 2Θ ≤ 56.56°), 14,387 unique (R_int_ = 0.0636, R_sigma_ = 0.0881), which were used in all calculations. The final R_1_ was 0.0814 (I > 2σ(I)) and wR_2_ was 0.2907 (all data).

*Crystal data for* **7a**: C_9_H_12_N_2_O_3_, (M = 196.21): triclinic, space group P-1, a = 4.3116(4) Å, b = 9.7366(10) Å, c = 12.3141(12) Å, α = 94.006(8)°, β = 94.579(8) ^o^, γ = 98.086(9)°, V = 508.45(9) Å^3^, Z = 2, μ = 0.097 mm^–1^, D_calc_ = 1.282 g/cm^3^, 3785 reflections measured to (7.60° ≤ 2Θ ≤ 52.74°), 2068 unique (R_int_ = 0.0291, R_sigma_ = 0.0531), which were used in all calculations. The final R_1_ was 0.0553 (I > 2σ(I)) and wR_2_ was 0.1786 (all data).

*Crystal data for* **7l**: C_14_H_12_F_2_N_2_O_3_, (M = 294.26): triclinic, space group P-1, a = 4.3326(6) Å, b = 11.619(2) Å, c = 13.963(2) Å, α = 96.867(13)°, β = 95.604(12)°, γ = 93.747(13)°, V = 692.40(18) Å^3^, Z = 2, μ = 0.118 mm^–1^, D_calc_ = 1.411 g/cm^3^, 4992 reflections measured to (5.92° ≤ 2Θ ≤ 58.82°), 3154 unique (R_int_ = 0.0339), which were used in all calculations. The final R_1_ was 0.0726 (I > 2σ(I)) and wR_2_ was 0.2211 (all data).

*Crystal data for* **9a**: C_10_H_10_N_2_OS, (M = 206.26): orthorhombic, space group Pbca, a = 15.6718(14) Å, b = 6.8466(6) Å, c = 18.4840(16) Å, V = 1983.3(3) Å^3^, Z = 8, μ = 0.292 mm^–1^, D_calc_ = 1.382 g/cm^3^, 8324 reflections measured to (7.85° ≤ 2Θ ≤ 59.104°), 2652 unique (R_int_ = 0.0657, R_sigma_ = 0.0597), which were used in all calculations. The final R_1_ was 0.0799 (I > 2σ(I)) and wR_2_ was 0.2593 (all data).

*Crystal data for* **9b**: C_15_H_12_N_2_OS, (M = 268.33): orthorhombic, space group Pbca, a = 6.6692(5) Å, b = 16.0059(11) Å, c = 24.8783(16) Å, V = 2655.7(3) Å^3^, Z = 8, μ = 0.236 mm^–1^, D_calc_ = 1.342 g/cm^3^, 10,931 reflections measured to (7.39° ≤ 2Θ ≤ 62.11°), 3627 unique (R_int_ = 0.0509, R_sigma_ = 0.0488), which were used in all calculations. The final R_1_ was 0.0603 (I > 2σ(I)) and wR_2_ was 0.2018 (all data).

*Crystal data for* **12**: C_15_H_18_N_2_O (M = 242.31): monoclinic, space group Pm, a = 8.0767(16) Å, b = 6.7744(16) Å, c = 13.682(3) Å, β = 105.611(19)°, V = 721.0(3) Å^3^, Z = 2, μ = 0.071 mm^–1^, D_calc_ = 1.116 g/cm^3^, 5286 reflections measured to (7.98^o^ ≤ 2Θ ≤ 56.52^o^), 2544 unique (R_int_ = 0.0560, R_sigma_ = 0.0744), which were used in all calculations. The final R_1_ was 0.0670 (I > 2σ(I)) and wR_2_ was 0.2083 (all data).

*Crystal data for* **14**: C_17_H_14_N_2_O, (M = 262.30): orthorhombic, space group Pbca, a = 6.8084(10) Å, b = 16.270(2) Å, c = 24.839(2) Å, V = 2751.5(6) Å^3^, Z = 8, μ = 0.080 mm^–1^, D_calc_ = 1.266 g/cm^3^, 10,377 reflections measured to (7.27° ≤ 2Θ ≤ 62.38°), 3788 unique (R_int_ = 0.0643, R_sigma_ = 0.0732), which were used in all calculations. The final R_1_ was 0.0715 (I > 2σ(I)) and wR_2_ was 0.2466 (all data).

*Crystal data for* **15b**: C_12_H_12_N_2_O, (M = 200.24): orthorhombic, space group Pbca, a = 17.093(2) Å, b = 6.9475(10) Å, c = 18.529(3) Å, V = 2200.4(5) Å^3^, Z = 8, μ = 0.079 mm^–1^, D_calc_ = 1.209 g/cm^3^, 7770 reflections measured to (7.56° ≤ 2Θ ≤ 60.98°), 3012 unique (R_int_ = 0.0703, R_sigma_ = 0.1195), which were used in all calculations. The final R_1_ was 0.0662 (I > 2σ(I)) and wR_2_ was 0.2241 (all data).

*Crystal data for* **21b**: C_13_H_12_N_2_O_3_, (M = 244.25): monoclinic, space group P2_1_/c, a = 7.8231(16) Å, b = 18.346(3) Å, c = 9.1104(19) Å, β = 110.58(2)°, V = 1224.1(4) Å^3^, Z = 4, μ = 0.096 mm^–1^, D_calc_ = 1.325 g/cm^3^, 6754 reflections measured to (7.40° ≤ 2Θ ≤ 58.26°), 3126 unique (R_int_ = 0.0577, R_sigma_ = 0.0901), which were used in all calculations. The final R_1_ was 0.0802 (I > 2σ(I)) and wR_2_ was 0.2579 (all data).

## 4. Conclusions

In this research, 1,2,4-triketone analogs were shown to serve as convenient building blocks for the synthesis of both pyrazoles and pyridazines via reactions with hydrazines. The chemo- and regioselectivity of cyclocondensations is ruled by several factors, among which are the triketone structure and reaction conditions (temperature, acidity). It was demonstrated how the electronic and steric effects of the substituents near the β-diketone fragment can direct the hydrazine attack toward a specific carbonyl group, controlling the regiochemistry of the heterocyclic core formation. In comparison with the reactions of fluorinated analogs in similar conditions, a variety of routes was revealed.

All of the reported 1,2,4-tricarbonyl compounds provided mono- or bifunctional pyrazoles. Diverse synthetic strategies to the 3- and 5-acetylpyrazoles were developed. In particular, the two-step method offering acetal derivatives as intermediates was found to be the most effective. The incorporation of ester and/or acetyl substituents makes these azoles promising precursors for subsequent transformations into specific products with tailored properties and biological activities such as pyrazole carboxamides, pyrazolyl hydrazones, *bis*-pyrazoles, and fused heterocycles.

Overall, by designing the structure of 1,2,4-triketone analogs and optimizing the reaction conditions, the selectivity of condensation reactions with hydrazines can be switched, ensuring the formation of the desired products.

## Figures and Tables

**Figure 5:**
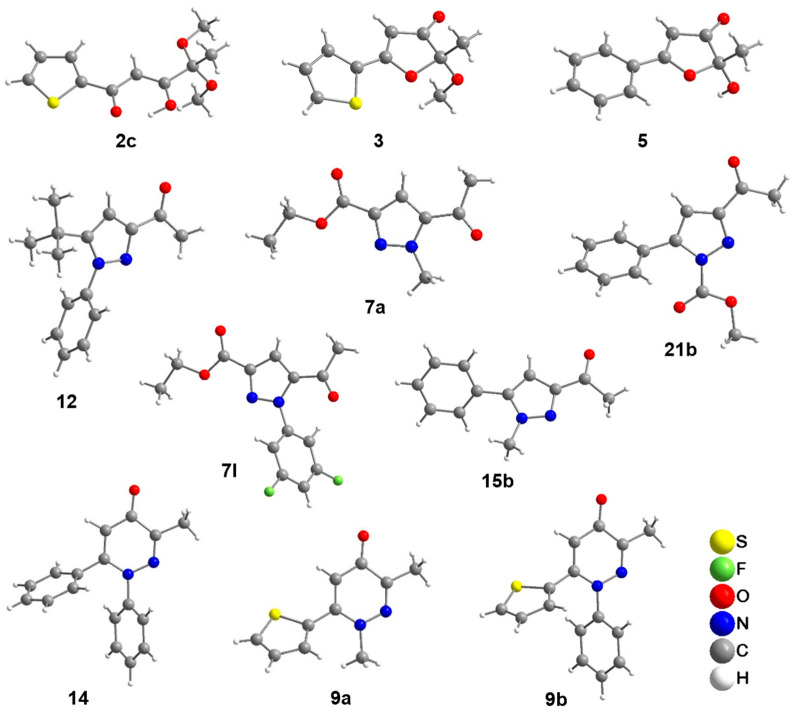
Molecular structure of 1,2,4-triketone analogs **2c**, **3**, **5**, pyrazoles **7a**, **7l**, **12**, **15b**, **21b**, and pyridazinones **9a**, **9b**, and **14** according to the XRD data.

**Table 1:** Summary of the data of the reactions between compounds **2b**/**4a** and the hydrazines.

Compound	Conditions	Products (Ratio)	Products (Yields)
**2b**	i	**13a**:**13b** (4:1)	**13a** (59%)
**2b**	ii	**14**	**14** (83%)
**2b**	iii	**15a**:**15b** (3:1)	**15a** (61%), **15b** (24%)
**2b**	iv	**15a**:**15b** (1:3)	–
**4a**	iii	**15a**:**15b** (3:1)	–

## Data Availability

Not applicable.

## Supplementary Materials

The following supporting information can be downloaded at: https://www.mdpi.com/article/10.3390/ijms241814234/s1.
