# Supplementary material for: Exploring Three Avenues: Chemo- and Regioselective Transformations of 1,2,4-Triketone Analogs into Pyrazoles and Pyridazinones
Source: Int J Mol Sci. 2023 Sep 18;24(18):14234. doi: 10.3390/ijms241814234 (PMC10531707; doi:10.3390/ijms241814234)

checkCIF/PLATON report

Structure factors have been supplied for datablock(s) exp_215

THIS REPORT IS FOR GUIDANCE ONLY. IF USED AS PART OF A REVIEW PROCEDURE FOR PUBLICATION, IT SHOULD NOT REPLACE THE EXPERTISE OF AN EXPERIENCED CRYSTALLOGRAPHIC REFEREE.

No syntax errors found. CIF dictionary Interpreting this report

Datablock: exp_215

```
Bond precision:   C-C = 0.0110 A          Wavelength=0.71073

Cell:             a=8.0767(16)     b=6.7744(16)         c=13.682(3)
                  alpha=90         beta=105.611(19)     gamma=90
Temperature:      295 K

                 Calculated                    Reported
Volume           721.0(3)                      721.0(3)
Space group      P m                           P 1 m 1
Hall group       P -2y                         P -2y
Moiety formula   C15 H18 N2 O                  2(C7.5 H9 N O0.5)
Sum formula      C15 H18 N2 O                  C15 H18 N2 O
Mr               242.31                        242.31
Dx,g cm-3        1.116                         1.116
Z                2                             2
Mu (mm-1)        0.071                         0.071
F000             260.0                         260.0
F000'            260.10
h,k,lmax         10,9,18                       10,9,18
Nref             3857[ 1933]                   2544
Tmin,Tmax        0.974,0.987                   0.225,1.000
Tmin'            0.969

Correction method= # Reported T Limits: Tmin=0.225 Tmax=1.000
AbsCorr = MULTI-SCAN

Data completeness= 1.32/0.66       Theta(max)= 28.259

R(reflections)= 0.0670( 1373)                          wR2(reflections)=
                                                       0.2083( 2544)
S = 1.006                    Npar= 204
```

The following ALERTS were generated. Each ALERT has the format
test-name_ALERT_alert-type_alert-level.
Click on the hyperlinks for more details of the test.

Alert level B

```
PLAT242_ALERT_2_B Low    'MainMol' Ueq as Compared to Neighbors of         C6 Check
PLAT242_ALERT_2_B Low    'MainMol' Ueq as Compared to Neighbors of        C6A Check
PLAT340_ALERT_3_B Low Bond Precision on  C-C Bonds ...............    0.01096 Ang.
```

Alert level C

```
STRVA01_ALERT_4_C           Flack parameter is too small
          From the CIF: _refine_ls_abs_structure_Flack   -4.000
          From the CIF: _refine_ls_abs_structure_Flack_su    1.000
PLAT042_ALERT_1_C Calc. and Reported MoietyFormula Strings  Differ     Please Check
PLAT220_ALERT_2_C NonSolvent   Resd 1  C   Ueq(max)/Ueq(min) Range        3.8 Ratio
PLAT220_ALERT_2_C NonSolvent   Resd 2  C   Ueq(max)/Ueq(min) Range        3.7 Ratio
PLAT234_ALERT_4_C Large Hirshfeld Difference C6A      --C8A      .       0.16 Ang.
PLAT790_ALERT_4_C Centre of Gravity not Within Unit Cell: Resd.  #          1 Note
             C15 H18 N2 O
PLAT910_ALERT_3_C Missing # of FCF Reflection(s) Below Theta(Min).          8 Note
PLAT911_ALERT_3_C Missing FCF Refl Between Thmin & STh/L=    0.600          8 Report
```

Alert level G

```
FORMU01_ALERT_2_G  There is a discrepancy between the atom counts in the
           _chemical_formula_sum and the formula from the _atom_site* data.
           Atom count from _chemical_formula_sum:C15 H18 N2 O1
           Atom count from the _atom_site data:  C15 H17.75 N2 O1
CELLZ01_ALERT_1_G Difference between formula and atom_site contents detected.
CELLZ01_ALERT_1_G WARNING: H atoms missing from atom site list. Is this intentional?
          From the CIF: _cell_formula_units_Z    2
          From the CIF: _chemical_formula_sum  C15 H18 N2 O
          TEST: Compare cell contents of formula and atom_site data

          atom    Z*formula  cif sites diff
          C         30.00     30.00    0.00
          H         36.00     35.50    0.50
          N          4.00      4.00    0.00
          O          2.00      2.00    0.00
PLAT012_ALERT_1_G No       _shelx_res_checksum Found in CIF ......     Please Check
PLAT032_ALERT_4_G Std. Uncertainty on Flack Parameter Value High .      1.000 Report
PLAT300_ALERT_4_G Atom Site Occupancy of H1A       Constrained at        0.5 Check
PLAT300_ALERT_4_G Atom Site Occupancy of H1B       Constrained at        0.5 Check
PLAT300_ALERT_4_G Atom Site Occupancy of H1C       Constrained at        0.5 Check
PLAT300_ALERT_4_G Atom Site Occupancy of H7A       Constrained at        0.5 Check
PLAT300_ALERT_4_G Atom Site Occupancy of H7B       Constrained at        0.5 Check
PLAT300_ALERT_4_G Atom Site Occupancy of H7C       Constrained at        0.5 Check
PLAT300_ALERT_4_G Atom Site Occupancy of H1AA      Constrained at        0.5 Check
PLAT300_ALERT_4_G Atom Site Occupancy of H1AB      Constrained at        0.5 Check
PLAT300_ALERT_4_G Atom Site Occupancy of H1AC      Constrained at        0.5 Check
PLAT300_ALERT_4_G Atom Site Occupancy of H8AA      Constrained at        0.5 Check
PLAT300_ALERT_4_G Atom Site Occupancy of H8AB      Constrained at        0.5 Check
PLAT300_ALERT_4_G Atom Site Occupancy of H8AC      Constrained at        0.5 Check
```

```
PLAT367_ALERT_2_G Long?  C(sp?)-C(sp?) Bond  C6       - C7       .       1.51 Ang.
PLAT367_ALERT_2_G Long?  C(sp?)-C(sp?) Bond  C6A      - C8A      .       1.50 Ang.
PLAT720_ALERT_4_G Number of Unusual/Non-Standard Labels .........           9 Note
PLAT789_ALERT_4_G Atoms with Negative _atom_site_disorder_group  #         12 Check
PLAT790_ALERT_4_G Centre of Gravity not Within Unit Cell: Resd.  #          2 Note
              C15 H18 N2 O
PLAT822_ALERT_4_G CIF-embedded .res Contains Negative PART Numbers          4 Check
PLAT912_ALERT_4_G Missing # of FCF Reflections Above STh/L=  0.600         17 Note
PLAT915_ALERT_3_G No Flack x Check Done: Low Friedel Pair Coverage         33 %
PLAT916_ALERT_2_G Hooft y and Flack x Parameter Values Differ by .       3.50 Check
PLAT933_ALERT_2_G Number of HKL-OMIT Records in Embedded .res File          3 Note
PLAT978_ALERT_2_G Number C-C Bonds with Positive Residual Density.          0 Info
```

```
  0 ALERT level A = Most likely a serious problem - resolve or explain
  3 ALERT level B = A potentially serious problem, consider carefully
  8 ALERT level C = Check. Ensure it is not caused by an omission or oversight
 28 ALERT level G = General information/check it is not something unexpected

  4 ALERT type 1 CIF construction/syntax error, inconsistent or missing data
 10 ALERT type 2 Indicator that the structure model may be wrong or deficient
  4 ALERT type 3 Indicator that the structure quality may be low
 21 ALERT type 4 Improvement, methodology, query or suggestion
  0 ALERT type 5 Informative message, check
```

It is advisable to attempt to resolve as many as possible of the alerts in all categories. Often the minor alerts point to easily fixed oversights, errors and omissions in your CIF or refinement strategy, so attention to these fine details can be worthwhile. In order to resolve some of the more serious problems it may be necessary to carry out additional measurements or structure refinements. However, the purpose of your study may justify the reported deviations and the more serious of these should normally be commented upon in the discussion or experimental section of a paper or in the "special_details" fields of the CIF. checkCIF was carefully designed to identify outliers and unusual parameters, but every test has its limitations and alerts that are not important in a particular case may appear. Conversely, the absence of alerts does not guarantee there are no aspects of the results needing attention. It is up to the individual to critically assess their own results and, if necessary, seek expert advice.

Publication of your CIF in IUCr journals

A basic structural check has been run on your CIF. These basic checks will be run on all CIFs submitted for publication in IUCr journals (*Acta Crystallographica*, *Journal of Applied Crystallography*, *Journal of Synchrotron Radiation*); however, if you intend to submit to *Acta Crystallographica Section C* or *E* or *IUCrData*, you should make sure that full publication checks are run on the final version of your CIF prior to submission.

Publication of your CIF in other journals

Please refer to the *Notes for Authors* of the relevant journal for any special instructions relating to CIF submission.

PLATON version of 06/07/2023; check.def file version of 30/06/2023

Datablock exp_215 - ellipsoid plot

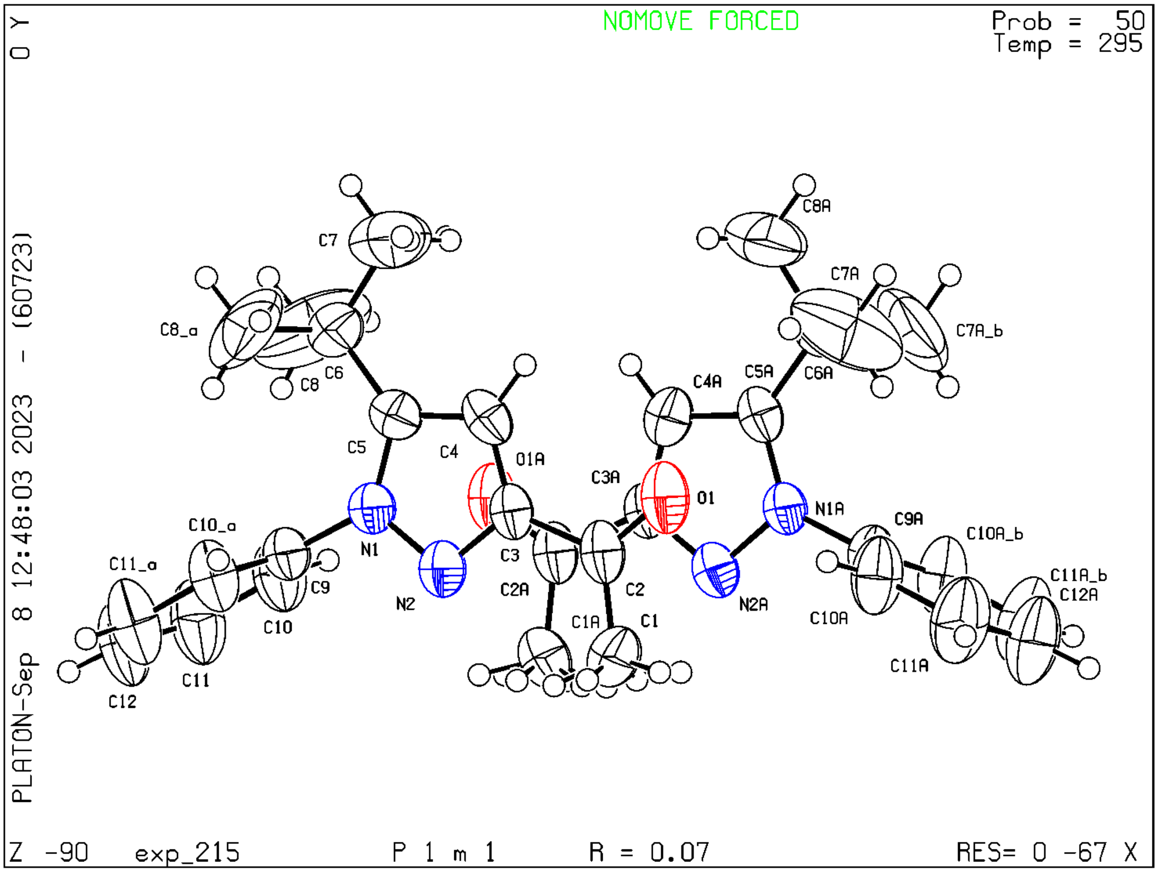

Supplement: Supplementary file 1 [file ijms-24-14234-s001.zip › ijms-2578992-supplementary/SI_ijms-2578992/checkcif 12.pdf]
