# Supplementary material for: Exploring Three Avenues: Chemo- and Regioselective Transformations of 1,2,4-Triketone Analogs into Pyrazoles and Pyridazinones
Source: Int J Mol Sci. 2023 Sep 18;24(18):14234. doi: 10.3390/ijms241814234 (PMC10531707; doi:10.3390/ijms241814234)

checkCIF/PLATON report

Structure factors have been supplied for datablock(s) exp_307

THIS REPORT IS FOR GUIDANCE ONLY. IF USED AS PART OF A REVIEW PROCEDURE FOR PUBLICATION, IT SHOULD NOT REPLACE THE EXPERTISE OF AN EXPERIENCED CRYSTALLOGRAPHIC REFEREE.

No syntax errors found. CIF dictionary Interpreting this report

Datablock: exp_307

```
Bond precision:   C-C = 0.0045 A           Wavelength=0.71073

Cell:             a=7.8231(16)     b=18.346(3)        c=9.1104(19)
                  alpha=90         beta=110.58(2)     gamma=90
Temperature:      295 K

                 Calculated                  Reported
Volume           1224.1(4)                   1224.1(4)
Space group      P 21/c                      P 1 21/c 1
Hall group       -P 2ybc                     -P 2ybc
Moiety formula   C13 H12 N2 O3               C13 H12 N2 O3
Sum formula      C13 H12 N2 O3               C13 H12 N2 O3
Mr               244.25                      244.25
Dx,g cm-3        1.325                       1.325
Z                4                           4
Mu (mm-1)        0.096                       0.096
F000             512.0                       512.0
F000'            512.25
h,k,lmax         10,25,12                    10,25,12
Nref             3304                        3126
Tmin,Tmax        0.959,0.983                 0.244,1.000
Tmin'            0.954

Correction method= # Reported T Limits: Tmin=0.244 Tmax=1.000
AbsCorr = MULTI-SCAN

Data completeness= 0.946           Theta(max)= 29.130

R(reflections)= 0.0802( 1555)                        wR2(reflections)=
                                                     0.2579( 3126)
S = 1.054                    Npar= 170
```

The following ALERTS were generated. Each ALERT has the format
test-name_ALERT_alert-type_alert-level.
Click on the hyperlinks for more details of the test.

Alert level C

```
PLAT029_ALERT_3_C _diffrn_measured_fraction_theta_full value Low .      0.974 Why?
PLAT031_ALERT_4_C Refined Extinction Parameter Within Range of ...      3.143 Sigma
PLAT084_ALERT_3_C High wR2 Value (i.e. > 0.25) ..................        0.26 Report
PLAT340_ALERT_3_C Low Bond Precision on  C-C Bonds ..............     0.00445 Ang.
PLAT905_ALERT_3_C Negative K value in the Analysis of Variance ...     -0.536 Report
PLAT910_ALERT_3_C Missing # of FCF Reflection(s) Below Theta(Min).          7 Note
PLAT911_ALERT_3_C Missing FCF Refl Between Thmin & STh/L=    0.600         51 Report
```

Alert level G

```
PLAT012_ALERT_1_G No       _shelx_res_checksum Found in CIF ......     Please Check
PLAT072_ALERT_2_G SHELXL First  Parameter in WGHT  Unusually Large       0.11 Report
PLAT912_ALERT_4_G Missing # of FCF Reflections Above STh/L=  0.600        120 Note
PLAT933_ALERT_2_G Number of HKL-OMIT Records in Embedded .res File         10 Note
PLAT978_ALERT_2_G Number C-C Bonds with Positive Residual Density.          0 Info
```

0 **ALERT level A** = Most likely a serious problem - resolve or explain
0 **ALERT level B** = A potentially serious problem, consider carefully
7 **ALERT level C** = Check. Ensure it is not caused by an omission or oversight
5 **ALERT level G** = General information/check it is not something unexpected

1 ALERT type 1 CIF construction/syntax error, inconsistent or missing data
3 ALERT type 2 Indicator that the structure model may be wrong or deficient
6 ALERT type 3 Indicator that the structure quality may be low
2 ALERT type 4 Improvement, methodology, query or suggestion
0 ALERT type 5 Informative message, check

It is advisable to attempt to resolve as many as possible of the alerts in all categories. Often the minor alerts point to easily fixed oversights, errors and omissions in your CIF or refinement strategy, so attention to these fine details can be worthwhile. In order to resolve some of the more serious problems it may be necessary to carry out additional measurements or structure refinements. However, the purpose of your study may justify the reported deviations and the more serious of these should normally be commented upon in the discussion or experimental section of a paper or in the "special_details" fields of the CIF. checkCIF was carefully designed to identify outliers and unusual parameters, but every test has its limitations and alerts that are not important in a particular case may appear. Conversely, the absence of alerts does not guarantee there are no aspects of the results needing attention. It is up to the individual to critically assess their own results and, if necessary, seek expert advice.

Publication of your CIF in IUCr journals

A basic structural check has been run on your CIF. These basic checks will be run on all CIFs submitted for publication in IUCr journals (*Acta Crystallographica*, *Journal of Applied Crystallography*, *Journal of Synchrotron Radiation*); however, if you intend to submit to *Acta Crystallographica Section C* or *E* or *IUCrData*, you should make sure that full publication checks are run on the final version of your CIF prior to submission.

Publication of your CIF in other journals

Please refer to the *Notes for Authors* of the relevant journal for any special instructions relating to CIF submission.

PLATON version of 06/07/2023; check.def file version of 30/06/2023

Datablock exp_307 - ellipsoid plot

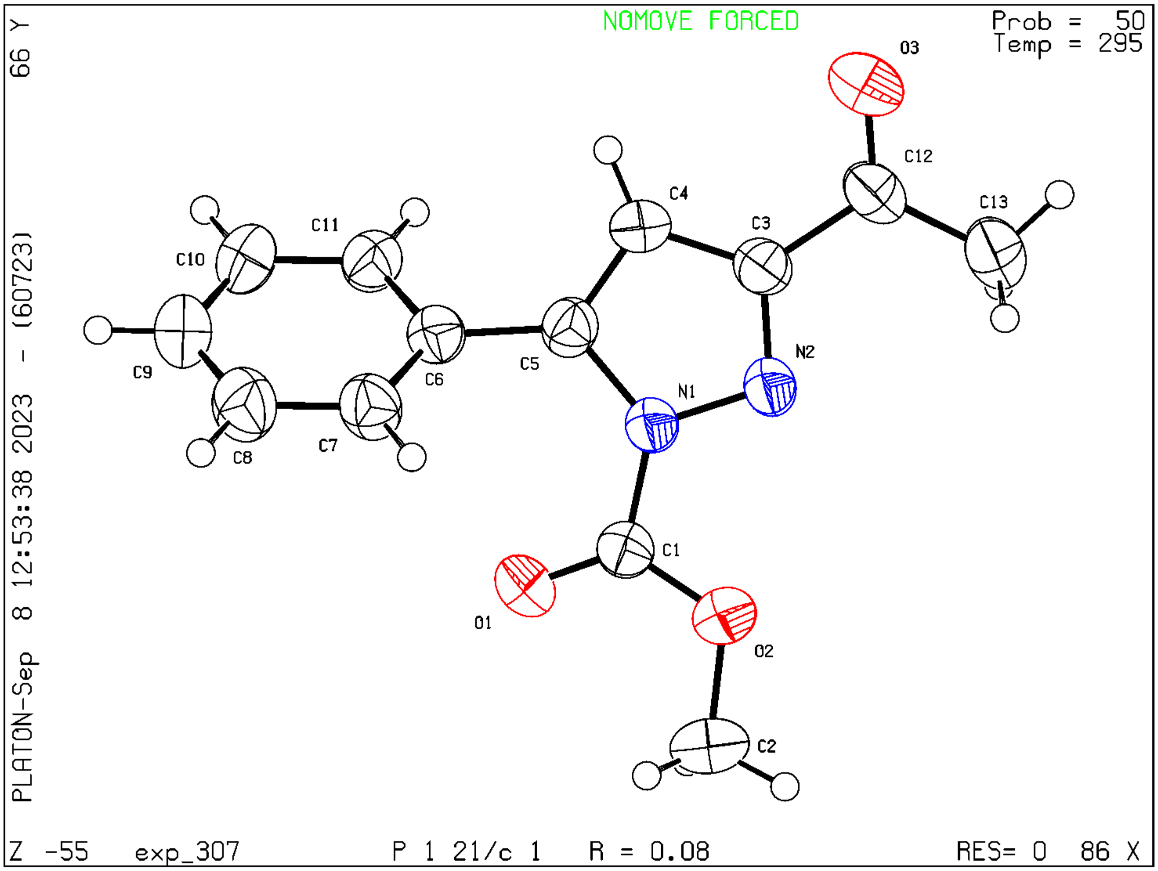

Supplement: Supplementary file 1 [file ijms-24-14234-s001.zip › ijms-2578992-supplementary/SI_ijms-2578992/checkcif 21b.pdf]
