# Supplementary material for: Exploring Three Avenues: Chemo- and Regioselective Transformations of 1,2,4-Triketone Analogs into Pyrazoles and Pyridazinones
Source: Int J Mol Sci. 2023 Sep 18;24(18):14234. doi: 10.3390/ijms241814234 (PMC10531707; doi:10.3390/ijms241814234)

checkCIF/PLATON report

Structure factors have been supplied for datablock(s) exp_306

THIS REPORT IS FOR GUIDANCE ONLY. IF USED AS PART OF A REVIEW PROCEDURE FOR PUBLICATION, IT SHOULD NOT REPLACE THE EXPERTISE OF AN EXPERIENCED CRYSTALLOGRAPHIC REFEREE.

No syntax errors found. CIF dictionary Interpreting this report

Datablock: exp_306

Bond precision: C-C = 0.0107 A Wavelength=0.71073

Cell:	a=11.1930(6)	b=11.1930(6)	c=19.785(3)
	alpha=90	beta=90	gamma=90
Temperature:	295 K		

	Calculated	Reported
Volume	2478.7(5)	2478.8(4)
Space group	P 43 21 2	P 43 21 2
Hall group	P 4nw 2abw	P 4nw 2abw
Moiety formula	C11 H14 O4 S	C11 H14 O4 S
Sum formula	C11 H14 O4 S	C11 H14 O4 S
Mr	242.28	242.28
Dx,g cm-3	1.298	1.298
Z	8	8
Mu (mm-1)	0.257	0.257
F000	1024.0	1024.0
F000'	1025.53	
h,k,lmax	14,14,26	12,14,26
Nref	3071[1829]	3046
Tmin,Tmax	0.892,0.940	0.006,1.000
Tmin'	0.882	

Correction method= # Reported T Limits: Tmin=0.006 Tmax=1.000
AbsCorr = MULTI-SCAN

Data completeness= 1.67/0.99 Theta(max)= 28.266

R(reflections)= 0.0770(1451) wR2(reflections)= 0.2588(3046)

S = 1.033 Npar= 162

The following ALERTS were generated. Each ALERT has the format
test-name_ALERT_alert-type_alert-level.
Click on the hyperlinks for more details of the test.

Alert level B

```
PLAT230_ALERT_2_B Hirshfeld Test Diff for    O1       --C11      .       10.1 s.u.
PLAT340_ALERT_3_B Low Bond Precision on  C-C Bonds ...............    0.01067 Ang.
```

Alert level C

```
PLAT026_ALERT_3_C Ratio Observed / Unique Reflections (too) Low ..        48% Check
PLAT084_ALERT_3_C High wR2 Value (i.e. > 0.25) ...................       0.26 Report
PLAT230_ALERT_2_C Hirshfeld Test Diff for    C1       --C2       .        5.2 s.u.
PLAT234_ALERT_4_C Large Hirshfeld Difference S1A      --C6       .       0.16 Ang.
PLAT234_ALERT_4_C Large Hirshfeld Difference C6       --C7       .       0.16 Ang.
PLAT241_ALERT_2_C High   'MainMol' Ueq as Compared to Neighbors of         O1 Check
PLAT242_ALERT_2_C Low    'MainMol' Ueq as Compared to Neighbors of         O2 Check
PLAT245_ALERT_2_C U(iso) H3        Smaller than U(eq) O3        by      0.027 Ang**2
PLAT355_ALERT_3_C Long   O-H (X0.82,N0.98A)  O3       - H3       .       1.03 Ang.
PLAT906_ALERT_3_C Large K Value in the Analysis of Variance ......      2.268 Check
PLAT910_ALERT_3_C Missing # of FCF Reflection(s) Below Theta(Min).          5 Note
PLAT911_ALERT_3_C Missing FCF Refl Between Thmin & STh/L=    0.600          9 Report
```

Alert level G

```
PLAT002_ALERT_2_G Number of Distance or Angle Restraints on AtSite          5 Note
PLAT012_ALERT_1_G No       _shelx_res_checksum Found in CIF ......     Please Check
PLAT072_ALERT_2_G SHELXL First  Parameter in WGHT  Unusually Large       0.11 Report
PLAT171_ALERT_4_G The CIF-Embedded .res File Contains EADP Records          2 Report
PLAT172_ALERT_4_G The CIF-Embedded .res File Contains DFIX Records          5 Report
PLAT300_ALERT_4_G Atom Site Occupancy of S1         Constrained at       0.75 Check
PLAT300_ALERT_4_G Atom Site Occupancy of S1A        Constrained at       0.25 Check
PLAT300_ALERT_4_G Atom Site Occupancy of C7         Constrained at       0.75 Check
PLAT300_ALERT_4_G Atom Site Occupancy of C7A        Constrained at       0.25 Check
PLAT300_ALERT_4_G Atom Site Occupancy of H7         Constrained at       0.75 Check
PLAT300_ALERT_4_G Atom Site Occupancy of H8A        Constrained at       0.75 Check
PLAT300_ALERT_4_G Atom Site Occupancy of H9A        Constrained at       0.75 Check
PLAT300_ALERT_4_G Atom Site Occupancy of H7A        Constrained at       0.25 Check
PLAT300_ALERT_4_G Atom Site Occupancy of H8B        Constrained at       0.25 Check
PLAT300_ALERT_4_G Atom Site Occupancy of H9B        Constrained at       0.25 Check
PLAT301_ALERT_3_G Main Residue  Disorder ..............(Resd  1  )        13% Note
PLAT860_ALERT_3_G Number of Least-Squares Restraints .............          5 Note
PLAT912_ALERT_4_G Missing # of FCF Reflections Above STh/L=  0.600          1 Note
PLAT933_ALERT_2_G Number of HKL-OMIT Records in Embedded .res File          3 Note
PLAT950_ALERT_5_G Calculated (ThMax) and CIF-Reported Hmax Differ           2 Units
PLAT978_ALERT_2_G Number C-C Bonds with Positive Residual Density.          0 Info
```

0 **ALERT level A** = Most likely a serious problem - resolve or explain
2 **ALERT level B** = A potentially serious problem, consider carefully
12 **ALERT level C** = Check. Ensure it is not caused by an omission or oversight
21 **ALERT level G** = General information/check it is not something unexpected

1 ALERT type 1 CIF construction/syntax error, inconsistent or missing data

```
  9 ALERT type 2 Indicator that the structure model may be wrong or deficient
  9 ALERT type 3 Indicator that the structure quality may be low
 15 ALERT type 4 Improvement, methodology, query or suggestion
  1 ALERT type 5 Informative message, check
```

It is advisable to attempt to resolve as many as possible of the alerts in all categories. Often the minor alerts point to easily fixed oversights, errors and omissions in your CIF or refinement strategy, so attention to these fine details can be worthwhile. In order to resolve some of the more serious problems it may be necessary to carry out additional measurements or structure refinements. However, the purpose of your study may justify the reported deviations and the more serious of these should normally be commented upon in the discussion or experimental section of a paper or in the "special_details" fields of the CIF. checkCIF was carefully designed to identify outliers and unusual parameters, but every test has its limitations and alerts that are not important in a particular case may appear. Conversely, the absence of alerts does not guarantee there are no aspects of the results needing attention. It is up to the individual to critically assess their own results and, if necessary, seek expert advice.

Publication of your CIF in IUCr journals

A basic structural check has been run on your CIF. These basic checks will be run on all CIFs submitted for publication in IUCr journals (*Acta Crystallographica*, *Journal of Applied Crystallography*, *Journal of Synchrotron Radiation*); however, if you intend to submit to *Acta Crystallographica Section C* or *E* or *IUCrData*, you should make sure that full publication checks are run on the final version of your CIF prior to submission.

Publication of your CIF in other journals

Please refer to the *Notes for Authors* of the relevant journal for any special instructions relating to CIF submission.

PLATON version of 06/07/2023; check.def file version of 30/06/2023

Datablock exp_306 - ellipsoid plot

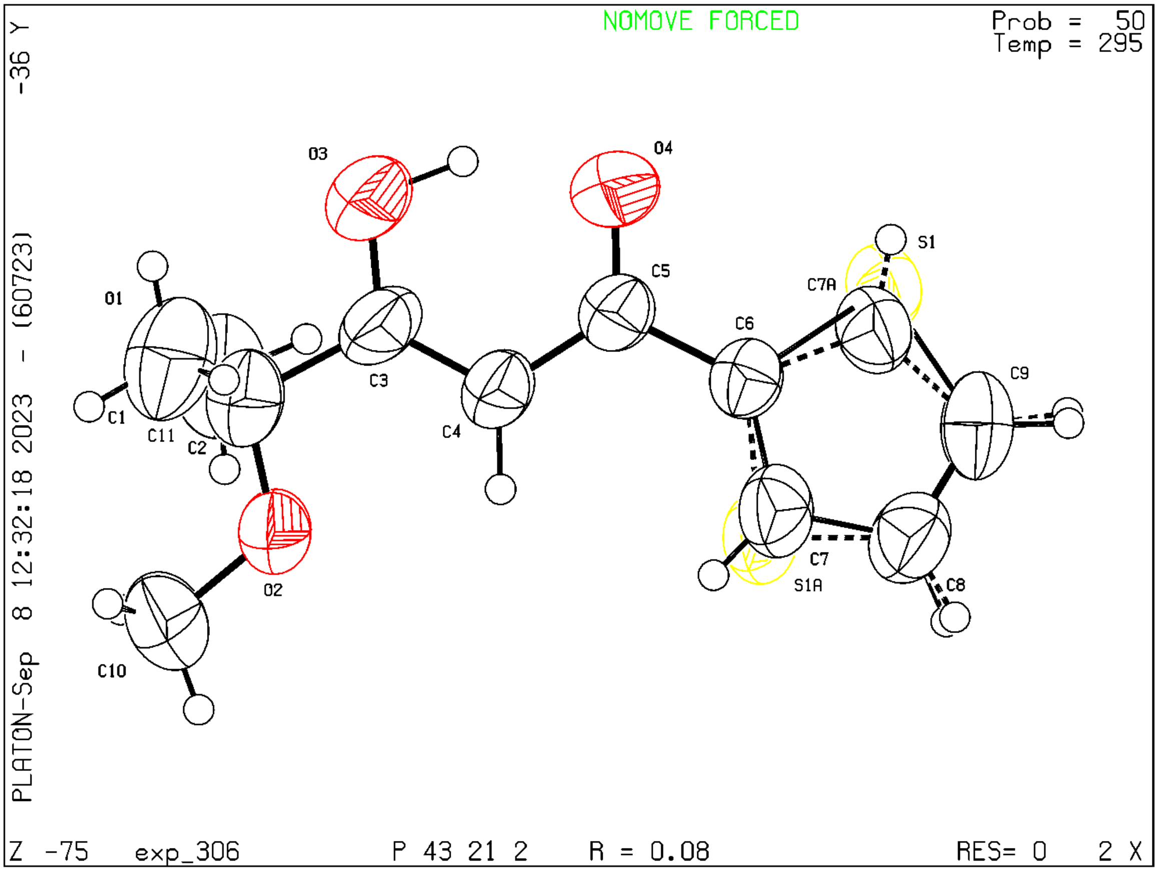

Supplement: Supplementary file 1 [file ijms-24-14234-s001.zip › ijms-2578992-supplementary/SI_ijms-2578992/checkcif 2c.pdf]
