# Supplementary material for: Exploring Three Avenues: Chemo- and Regioselective Transformations of 1,2,4-Triketone Analogs into Pyrazoles and Pyridazinones
Source: Int J Mol Sci. 2023 Sep 18;24(18):14234. doi: 10.3390/ijms241814234 (PMC10531707; doi:10.3390/ijms241814234)

checkCIF/PLATON report

Structure factors have been supplied for datablock(s) exp_521

THIS REPORT IS FOR GUIDANCE ONLY. IF USED AS PART OF A REVIEW PROCEDURE FOR PUBLICATION, IT SHOULD NOT REPLACE THE EXPERTISE OF AN EXPERIENCED CRYSTALLOGRAPHIC REFEREE.

No syntax errors found. CIF dictionary Interpreting this report

Datablock: exp_521

```
Bond precision:   C-C = 0.0045 A          Wavelength=0.71073

Cell:             a=11.1721(12)     b=9.9883(8)        c=18.6591(16)
                  alpha=90          beta=91.751(8)     gamma=90
Temperature:      295 K
```

	Calculated	Reported
Volume	2081.2(3)	2081.2(3)
Space group	P 21/c	P2(1)/c
Hall group	-P 2ybc	?
Moiety formula	C10 H10 O3 S	C10 H10 O3 S
Sum formula	C10 H10 O3 S	C10 H10 O3 S
Mr	210.24	210.24
Dx,g cm-3	1.342	1.342
Z	8	8
Mu (mm-1)	0.289	0.289
F000	880.0	880.0
F000'	881.43	
h,k,lmax	14,13,24	14,13,24
Nref	5169	5033
Tmin,Tmax	0.914,0.949	0.595,1.000
Tmin'	0.896	

```
Correction method= # Reported T Limits: Tmin=0.595 Tmax=1.000
AbsCorr = MULTI-SCAN

Data completeness= 0.974          Theta(max)= 28.280

R(reflections)= 0.0635( 2440)     wR2(reflections)= 0.2244( 5033)

S = 1.003                Npar= 328
```

The following ALERTS were generated. Each ALERT has the format
test-name_ALERT_alert-type_alert-level.
Click on the hyperlinks for more details of the test.

Alert level B

```
PLAT241_ALERT_2_B High   'MainMol' Ueq as Compared to Neighbors of        O1A Check
PLAT910_ALERT_3_B Missing # of FCF Reflection(s) Below Theta(Min).         12 Note
```

Alert level C

```
PLAT026_ALERT_3_C Ratio Observed / Unique Reflections (too) Low ..        48% Check
PLAT241_ALERT_2_C High   'MainMol' Ueq as Compared to Neighbors of         O1 Check
PLAT241_ALERT_2_C High   'MainMol' Ueq as Compared to Neighbors of         S2 Check
PLAT242_ALERT_2_C Low    'MainMol' Ueq as Compared to Neighbors of         C6 Check
PLAT242_ALERT_2_C Low    'MainMol' Ueq as Compared to Neighbors of        C5A Check
PLAT340_ALERT_3_C Low Bond Precision on  C-C Bonds ...............     0.0045 Ang.
PLAT905_ALERT_3_C Negative K value in the Analysis of Variance ...     -2.412 Report
PLAT911_ALERT_3_C Missing FCF Refl Between Thmin & STh/L=    0.600         30 Report
```

Alert level G

```
PLAT002_ALERT_2_G Number of Distance or Angle Restraints on AtSite          9 Note
PLAT003_ALERT_2_G Number of Uiso or Uij Restrained non-H Atoms ...          6 Report
PLAT005_ALERT_5_G No Embedded Refinement Details Found  in the CIF     Please Do !
PLAT300_ALERT_4_G Atom Site Occupancy of S1          Constrained at       0.8 Check
PLAT300_ALERT_4_G Atom Site Occupancy of S1C         Constrained at       0.2 Check
PLAT300_ALERT_4_G Atom Site Occupancy of O3          Constrained at       0.5 Check
PLAT300_ALERT_4_G Atom Site Occupancy of O3C         Constrained at       0.5 Check
PLAT300_ALERT_4_G Atom Site Occupancy of C1          Constrained at       0.5 Check
PLAT300_ALERT_4_G Atom Site Occupancy of C1C         Constrained at       0.5 Check
PLAT300_ALERT_4_G Atom Site Occupancy of C3          Constrained at       0.5 Check
PLAT300_ALERT_4_G Atom Site Occupancy of C3C         Constrained at       0.5 Check
PLAT300_ALERT_4_G Atom Site Occupancy of C7          Constrained at       0.8 Check
PLAT300_ALERT_4_G Atom Site Occupancy of C10         Constrained at       0.5 Check
PLAT300_ALERT_4_G Atom Site Occupancy of C10C        Constrained at       0.5 Check
PLAT300_ALERT_4_G Atom Site Occupancy of C7C         Constrained at       0.2 Check
PLAT300_ALERT_4_G Atom Site Occupancy of H1A         Constrained at       0.5 Check
PLAT300_ALERT_4_G Atom Site Occupancy of H1B         Constrained at       0.5 Check
PLAT300_ALERT_4_G Atom Site Occupancy of H1C         Constrained at       0.5 Check
PLAT300_ALERT_4_G Atom Site Occupancy of H1CA        Constrained at       0.5 Check
PLAT300_ALERT_4_G Atom Site Occupancy of H7A         Constrained at       0.8 Check
PLAT300_ALERT_4_G Atom Site Occupancy of H1CB        Constrained at       0.5 Check
PLAT300_ALERT_4_G Atom Site Occupancy of H1CC        Constrained at       0.5 Check
PLAT300_ALERT_4_G Atom Site Occupancy of H10A        Constrained at       0.5 Check
PLAT300_ALERT_4_G Atom Site Occupancy of H10B        Constrained at       0.5 Check
PLAT300_ALERT_4_G Atom Site Occupancy of H10C        Constrained at       0.5 Check
PLAT300_ALERT_4_G Atom Site Occupancy of H10G        Constrained at       0.5 Check
PLAT300_ALERT_4_G Atom Site Occupancy of H10H        Constrained at       0.5 Check
PLAT300_ALERT_4_G Atom Site Occupancy of H10I        Constrained at       0.5 Check
PLAT300_ALERT_4_G Atom Site Occupancy of H7CA        Constrained at       0.2 Check
PLAT300_ALERT_4_G Atom Site Occupancy of O3A         Constrained at       0.5 Check
PLAT300_ALERT_4_G Atom Site Occupancy of O3B         Constrained at       0.5 Check
PLAT300_ALERT_4_G Atom Site Occupancy of C1A         Constrained at       0.5 Check
PLAT300_ALERT_4_G Atom Site Occupancy of C1B         Constrained at       0.5 Check
```

```
PLAT300_ALERT_4_G Atom Site Occupancy of C3A       Constrained at       0.5 Check
PLAT300_ALERT_4_G Atom Site Occupancy of C3B       Constrained at       0.5 Check
PLAT300_ALERT_4_G Atom Site Occupancy of C10A      Constrained at       0.5 Check
PLAT300_ALERT_4_G Atom Site Occupancy of C10B      Constrained at       0.5 Check
PLAT300_ALERT_4_G Atom Site Occupancy of H1AA      Constrained at       0.5 Check
PLAT300_ALERT_4_G Atom Site Occupancy of H1AB      Constrained at       0.5 Check
PLAT300_ALERT_4_G Atom Site Occupancy of H1AC      Constrained at       0.5 Check
PLAT300_ALERT_4_G Atom Site Occupancy of H1BA      Constrained at       0.5 Check
PLAT300_ALERT_4_G Atom Site Occupancy of H10D      Constrained at       0.5 Check
PLAT300_ALERT_4_G Atom Site Occupancy of H10E      Constrained at       0.5 Check
PLAT300_ALERT_4_G Atom Site Occupancy of H10F      Constrained at       0.5 Check
PLAT300_ALERT_4_G Atom Site Occupancy of H10J      Constrained at       0.5 Check
PLAT300_ALERT_4_G Atom Site Occupancy of H10K      Constrained at       0.5 Check
PLAT300_ALERT_4_G Atom Site Occupancy of H10L      Constrained at       0.5 Check
PLAT300_ALERT_4_G Atom Site Occupancy of H1BB      Constrained at       0.5 Check
PLAT300_ALERT_4_G Atom Site Occupancy of H1BC      Constrained at       0.5 Check
PLAT301_ALERT_3_G Main Residue  Disorder .............(Resd  1  )       43% Note
PLAT301_ALERT_3_G Main Residue  Disorder .............(Resd  2  )       29% Note
PLAT398_ALERT_2_G Deviating  C-O-C   Angle From 120 for O1       .      29.3 Degree
PLAT398_ALERT_2_G Deviating  C-O-C   Angle From 120 for O3C      .     106.7 Degree
PLAT398_ALERT_2_G Deviating  C-O-C   Angle From 120 for O1A      .      32.7 Degree
PLAT720_ALERT_4_G Number of Unusual/Non-Standard Labels ..........        13 Note
PLAT779_ALERT_4_G Suspect or Irrelevant (Bond) Angle(s) in CIF ...     29.40 Deg.
              C3   -O1   -C3C     1_555   1_555   1_555 ......    #    3 Check
PLAT779_ALERT_4_G Suspect or Irrelevant (Bond) Angle(s) in CIF ...     28.90 Deg.
              C3   -C2   -C3C     1_555   1_555   1_555 ......    #    9 Check
PLAT779_ALERT_4_G Suspect or Irrelevant (Bond) Angle(s) in CIF ...      7.20 Deg.
              C7   -C6   -S1C     1_555   1_555   1_555 ......    #   19 Check
PLAT779_ALERT_4_G Suspect or Irrelevant (Bond) Angle(s) in CIF ...     11.40 Deg.
              C7C  -C6   -S1      1_555   1_555   1_555 ......    #   23 Check
PLAT779_ALERT_4_G Suspect or Irrelevant (Bond) Angle(s) in CIF ...     10.90 Deg.
              C7   -C8   -S1C     1_555   1_555   1_555 ......    #   28 Check
PLAT779_ALERT_4_G Suspect or Irrelevant (Bond) Angle(s) in CIF ...     12.40 Deg.
              C7C  -C9   -S1      1_555   1_555   1_555 ......    #   34 Check
PLAT779_ALERT_4_G Suspect or Irrelevant (Bond) Angle(s) in CIF ...     31.90 Deg.
              C3A  -C2A  -C3B     1_555   1_555   1_555 ......    #   53 Check
PLAT779_ALERT_4_G Suspect or Irrelevant (Bond) Angle(s) in CIF ...     32.70 Deg.
              C3B  -O1A  -C3A     1_555   1_555   1_555 ......    #   56 Check
PLAT811_ALERT_5_G No ADDSYM Analysis: Too Many Excluded Atoms ....         ! Info
PLAT860_ALERT_3_G Number of Least-Squares Restraints .............        42 Note
PLAT899_ALERT_4_G SHELXL-97  is Deprecated and Succeeded by SHELXL     2019/3 Note
PLAT912_ALERT_4_G Missing # of FCF Reflections Above STh/L=  0.600        98 Note
PLAT978_ALERT_2_G Number C-C Bonds with Positive Residual Density.         0 Info
PLAT992_ALERT_5_G Repd & Actual _reflns_number_gt Values Differ by         2 Check
```

---

0 **ALERT level A** = Most likely a serious problem - resolve or explain
2 **ALERT level B** = A potentially serious problem, consider carefully
8 **ALERT level C** = Check. Ensure it is not caused by an omission or oversight
69 **ALERT level G** = General information/check it is not something unexpected

0 ALERT type 1 CIF construction/syntax error, inconsistent or missing data
11 ALERT type 2 Indicator that the structure model may be wrong or deficient
8 ALERT type 3 Indicator that the structure quality may be low
57 ALERT type 4 Improvement, methodology, query or suggestion
3 ALERT type 5 Informative message, check

It is advisable to attempt to resolve as many as possible of the alerts in all categories. Often the minor alerts point to easily fixed oversights, errors and omissions in your CIF or refinement strategy, so attention to these fine details can be worthwhile. In order to resolve some of the more serious problems it may be necessary to carry out additional measurements or structure refinements. However, the purpose of your study may justify the reported deviations and the more serious of these should normally be commented upon in the discussion or experimental section of a paper or in the "special_details" fields of the CIF. checkCIF was carefully designed to identify outliers and unusual parameters, but every test has its limitations and alerts that are not important in a particular case may appear. Conversely, the absence of alerts does not guarantee there are no aspects of the results needing attention. It is up to the individual to critically assess their own results and, if necessary, seek expert advice.

Publication of your CIF in IUCr journals

A basic structural check has been run on your CIF. These basic checks will be run on all CIFs submitted for publication in IUCr journals (*Acta Crystallographica*, *Journal of Applied Crystallography*, *Journal of Synchrotron Radiation*); however, if you intend to submit to *Acta Crystallographica Section C* or *E* or *IUCrData*, you should make sure that full publication checks are run on the final version of your CIF prior to submission.

Publication of your CIF in other journals

Please refer to the *Notes for Authors* of the relevant journal for any special instructions relating to CIF submission.

PLATON version of 06/07/2023; check.def file version of 30/06/2023

Datablock exp_521 - ellipsoid plot

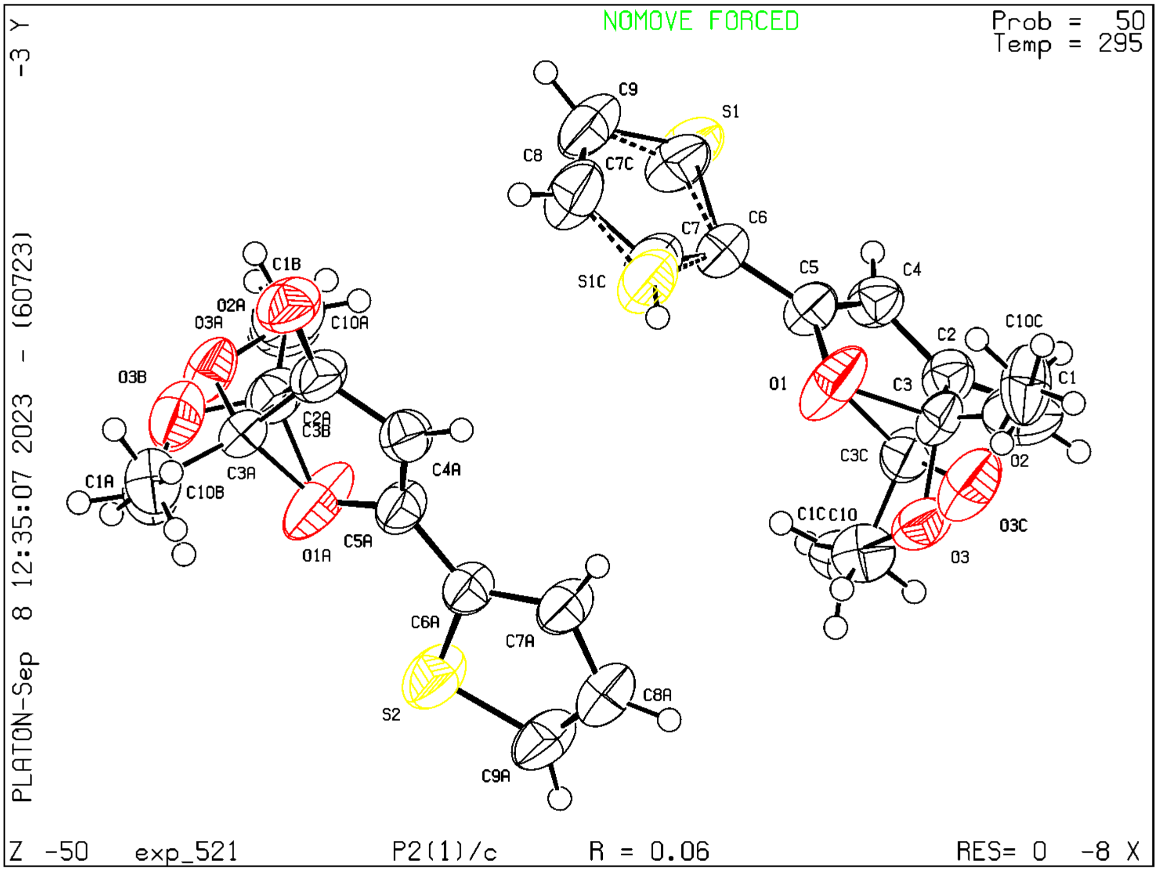

Supplement: Supplementary file 1 [file ijms-24-14234-s001.zip › ijms-2578992-supplementary/SI_ijms-2578992/checkcif 3.pdf]
