# Supplementary material for: Exploring Three Avenues: Chemo- and Regioselective Transformations of 1,2,4-Triketone Analogs into Pyrazoles and Pyridazinones
Source: Int J Mol Sci. 2023 Sep 18;24(18):14234. doi: 10.3390/ijms241814234 (PMC10531707; doi:10.3390/ijms241814234)

checkCIF/PLATON report

Structure factors have been supplied for datablock(s) exp_199

THIS REPORT IS FOR GUIDANCE ONLY. IF USED AS PART OF A REVIEW PROCEDURE FOR PUBLICATION, IT SHOULD NOT REPLACE THE EXPERTISE OF AN EXPERIENCED CRYSTALLOGRAPHIC REFEREE.

No syntax errors found. CIF dictionary Interpreting this report

Datablock: exp_199

```
Bond precision:   C-C = 0.0057 A           Wavelength=0.71073

Cell:            a=10.4213(6)      b=15.8394(9)      c=18.2990(12)
                 alpha=85.814(5)   beta=84.801(5)    gamma=78.314(5)
Temperature:     295 K

                  Calculated                  Reported
Volume            2941.2(3)                   2941.2(3)
Space group       P -1                        P -1
Hall group        -P 1                        -P 1
Moiety formula    C11 H10 O3                  C11 H10 O3
Sum formula       C11 H10 O3                  C11 H10 O3
Mr                190.19                      190.19
Dx,g cm-3         1.288                       1.289
Z                 12                          12
Mu (mm-1)         0.094                       0.094
F000              1200.0                      1200.0
F000'             1200.68
h,k,lmax          13,21,24                    13,21,24
Nref              14589                       14387
Tmin,Tmax         0.966,0.987                 0.659,1.000
Tmin'             0.959

Correction method= # Reported T Limits: Tmin=0.659 Tmax=1.000
AbsCorr = MULTI-SCAN

Data completeness= 0.986           Theta(max)= 28.281

R(reflections)= 0.0814( 6249)                     wR2(reflections)=
                                                  0.2907( 14387)
S = 1.050                  Npar= 812
```

The following ALERTS were generated. Each ALERT has the format
test-name_ALERT_alert-type_alert-level.
Click on the hyperlinks for more details of the test.

Alert level B

```
PLAT230_ALERT_2_B Hirshfeld Test Diff for    C9D      --C10D     .        7.4 s.u.
PLAT910_ALERT_3_B Missing # of FCF Reflection(s) Below Theta(Min).        29 Note
```

Alert level C

```
PLAT026_ALERT_3_C Ratio Observed / Unique Reflections (too) Low ..        43% Check
PLAT084_ALERT_3_C High wR2 Value (i.e. > 0.25) ..................       0.29 Report
PLAT340_ALERT_3_C Low Bond Precision on  C-C Bonds ..............    0.00574 Ang.
PLAT355_ALERT_3_C Long   O-H (X0.82,N0.98A)  O2       - H2       .       1.05 Ang.
PLAT355_ALERT_3_C Long   O-H (X0.82,N0.98A)  O2A      - H2A      .       1.01 Ang.
PLAT790_ALERT_4_C Centre of Gravity not Within Unit Cell: Resd.  #          1 Note
              C11 H10 O3
PLAT906_ALERT_3_C Large K Value in the Analysis of Variance ......      7.233 Check
PLAT911_ALERT_3_C Missing FCF Refl Between Thmin & STh/L=    0.600         89 Report
```

Alert level G

```
PLAT012_ALERT_1_G No       _shelx_res_checksum Found in CIF ......     Please Check
PLAT072_ALERT_2_G SHELXL First  Parameter in WGHT  Unusually Large       0.12 Report
PLAT154_ALERT_1_G The s.u.'s on the Cell Angles are Equal ..(Note)      0.005 Degree
PLAT398_ALERT_2_G Deviating  C-O-C   Angle From 120 for O1       .      107.9 Degree
PLAT398_ALERT_2_G Deviating  C-O-C   Angle From 120 for O1A      .      108.7 Degree
PLAT398_ALERT_2_G Deviating  C-O-C   Angle From 120 for O1B      .      108.6 Degree
PLAT398_ALERT_2_G Deviating  C-O-C   Angle From 120 for O1C      .      106.8 Degree
PLAT398_ALERT_2_G Deviating  C-O-C   Angle From 120 for O1D      .      108.3 Degree
PLAT398_ALERT_2_G Deviating  C-O-C   Angle From 120 for O1E      .      108.8 Degree
PLAT720_ALERT_4_G Number of Unusual/Non-Standard Labels .........         15 Note
PLAT790_ALERT_4_G Centre of Gravity not Within Unit Cell: Resd.  #          3 Note
              C11 H10 O3
PLAT912_ALERT_4_G Missing # of FCF Reflections Above STh/L=  0.600         85 Note
PLAT933_ALERT_2_G Number of HKL-OMIT Records in Embedded .res File         13 Note
PLAT978_ALERT_2_G Number C-C Bonds with Positive Residual Density.          0 Info
PLAT992_ALERT_5_G Repd & Actual _reflns_number_gt Values Differ by          4 Check
```

0 **ALERT level A** = Most likely a serious problem - resolve or explain
2 **ALERT level B** = A potentially serious problem, consider carefully
8 **ALERT level C** = Check. Ensure it is not caused by an omission or oversight
15 **ALERT level G** = General information/check it is not something unexpected

2 ALERT type 1 CIF construction/syntax error, inconsistent or missing data
10 ALERT type 2 Indicator that the structure model may be wrong or deficient
8 ALERT type 3 Indicator that the structure quality may be low
4 ALERT type 4 Improvement, methodology, query or suggestion
1 ALERT type 5 Informative message, check

It is advisable to attempt to resolve as many as possible of the alerts in all categories. Often the minor alerts point to easily fixed oversights, errors and omissions in your CIF or refinement strategy, so attention to these fine details can be worthwhile. In order to resolve some of the more serious problems it may be necessary to carry out additional measurements or structure refinements. However, the purpose of your study may justify the reported deviations and the more serious of these should normally be commented upon in the discussion or experimental section of a paper or in the "special_details" fields of the CIF. checkCIF was carefully designed to identify outliers and unusual parameters, but every test has its limitations and alerts that are not important in a particular case may appear. Conversely, the absence of alerts does not guarantee there are no aspects of the results needing attention. It is up to the individual to critically assess their own results and, if necessary, seek expert advice.

Publication of your CIF in IUCr journals

A basic structural check has been run on your CIF. These basic checks will be run on all CIFs submitted for publication in IUCr journals (*Acta Crystallographica*, *Journal of Applied Crystallography*, *Journal of Synchrotron Radiation*); however, if you intend to submit to *Acta Crystallographica Section C* or *E* or *IUCrData*, you should make sure that full publication checks are run on the final version of your CIF prior to submission.

Publication of your CIF in other journals

Please refer to the *Notes for Authors* of the relevant journal for any special instructions relating to CIF submission.

PLATON version of 06/07/2023; check.def file version of 30/06/2023

Datablock exp_199 - ellipsoid plot

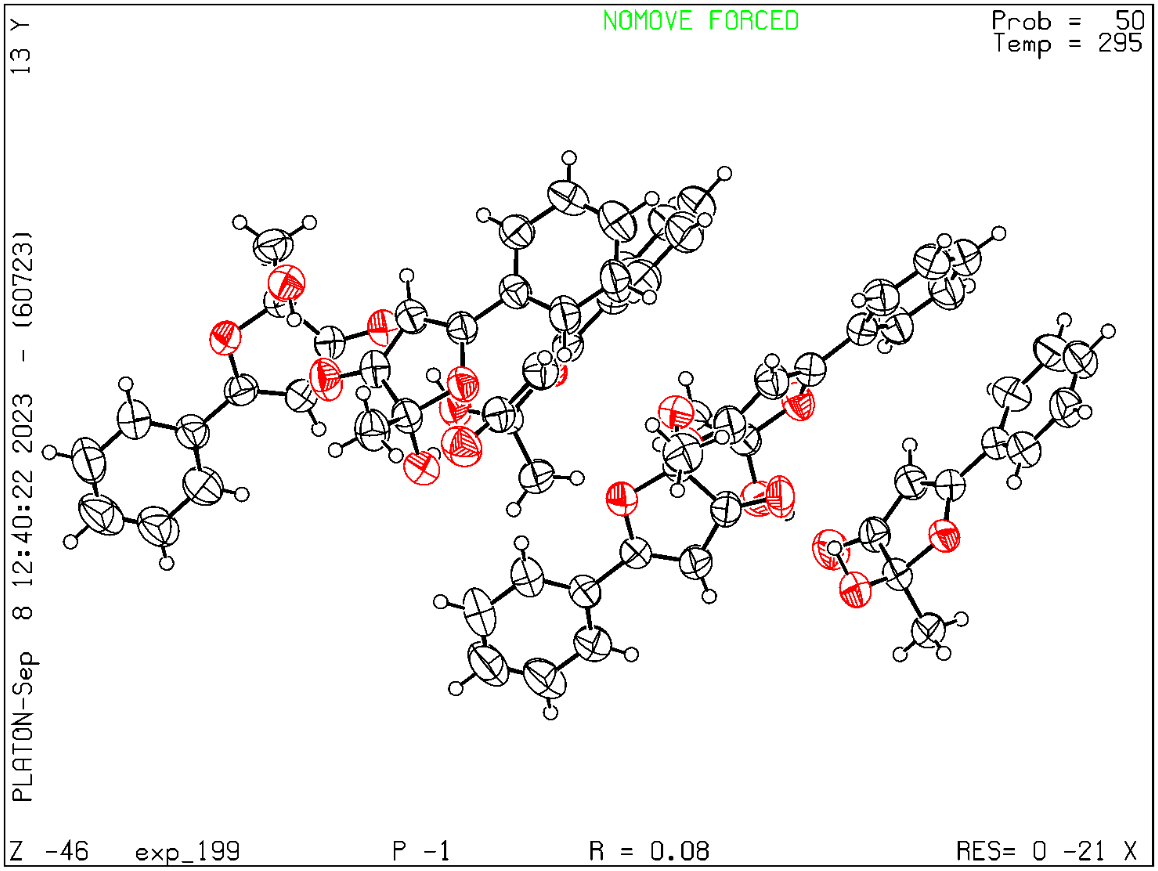

Supplement: Supplementary file 1 [file ijms-24-14234-s001.zip › ijms-2578992-supplementary/SI_ijms-2578992/checkcif 5.pdf]
