# Supplementary material for: Exploring Three Avenues: Chemo- and Regioselective Transformations of 1,2,4-Triketone Analogs into Pyrazoles and Pyridazinones
Source: Int J Mol Sci. 2023 Sep 18;24(18):14234. doi: 10.3390/ijms241814234 (PMC10531707; doi:10.3390/ijms241814234)

checkCIF/PLATON report

Structure factors have been supplied for datablock(s) exp_165

THIS REPORT IS FOR GUIDANCE ONLY. IF USED AS PART OF A REVIEW PROCEDURE FOR PUBLICATION, IT SHOULD NOT REPLACE THE EXPERTISE OF AN EXPERIENCED CRYSTALLOGRAPHIC REFEREE.

No syntax errors found. CIF dictionary Interpreting this report

Datablock: exp_165

```
Bond precision:   C-C = 0.0032 A          Wavelength=0.71073

Cell:            a=4.3116(4)        b=9.7366(10)      c=12.3141(12)
                 alpha=94.006(8)    beta=94.579(8)    gamma=98.086(9)
Temperature:     295 K

                  Calculated                  Reported
Volume            508.45(9)                   508.45(9)
Space group       P -1                        P -1
Hall group        -P 1                        -P 1
Moiety formula    C9 H12 N2 O3                C9 H12 N2 O3
Sum formula       C9 H12 N2 O3                C9 H12 N2 O3
Mr                196.21                      196.21
Dx,g cm-3         1.282                       1.282
Z                 2                           2
Mu (mm-1)         0.097                       0.097
F000              208.0                       208.0
F000'             208.11
h,k,lmax          5,12,15                     5,12,15
Nref              2086                        2068
Tmin,Tmax         0.976,0.992                 0.676,1.000
Tmin'             0.955

Correction method= # Reported T Limits: Tmin=0.676 Tmax=1.000
AbsCorr = MULTI-SCAN

Data completeness= 0.991           Theta(max)= 26.371

R(reflections)= 0.0553( 1222)                           wR2(reflections)=
                                                        0.1786( 2068)
S = 1.001                   Npar= 135
```

The following ALERTS were generated. Each ALERT has the format
test-name_ALERT_alert-type_alert-level.
Click on the hyperlinks for more details of the test.

Alert level B

```
PLAT031_ALERT_4_B Refined Extinction Parameter Within Range of ...      2.273 Sigma
```

Alert level C

```
PLAT242_ALERT_2_C Low    'MainMol' Ueq as Compared to Neighbors of         C6 Check
PLAT905_ALERT_3_C Negative K value in the Analysis of Variance ...     -3.178 Report
PLAT910_ALERT_3_C Missing # of FCF Reflection(s) Below Theta(Min).          5 Note
PLAT911_ALERT_3_C Missing FCF Refl Between Thmin & STh/L=    0.600          8 Report
```

Alert level G

```
PLAT012_ALERT_1_G No       _shelx_res_checksum Found in CIF ......     Please Check
PLAT912_ALERT_4_G Missing # of FCF Reflections Above STh/L=  0.600          5 Note
PLAT933_ALERT_2_G Number of HKL-OMIT Records in Embedded .res File          3 Note
PLAT978_ALERT_2_G Number C-C Bonds with Positive Residual Density.          0 Info
PLAT992_ALERT_5_G Repd & Actual _reflns_number_gt Values Differ by          4 Check
```

0 **ALERT level A** = Most likely a serious problem - resolve or explain
1 **ALERT level B** = A potentially serious problem, consider carefully
4 **ALERT level C** = Check. Ensure it is not caused by an omission or oversight
5 **ALERT level G** = General information/check it is not something unexpected

1 ALERT type 1 CIF construction/syntax error, inconsistent or missing data
3 ALERT type 2 Indicator that the structure model may be wrong or deficient
3 ALERT type 3 Indicator that the structure quality may be low
2 ALERT type 4 Improvement, methodology, query or suggestion
1 ALERT type 5 Informative message, check

It is advisable to attempt to resolve as many as possible of the alerts in all categories. Often the minor alerts point to easily fixed oversights, errors and omissions in your CIF or refinement strategy, so attention to these fine details can be worthwhile. In order to resolve some of the more serious problems it may be necessary to carry out additional measurements or structure refinements. However, the purpose of your study may justify the reported deviations and the more serious of these should normally be commented upon in the discussion or experimental section of a paper or in the "special_details" fields of the CIF. checkCIF was carefully designed to identify outliers and unusual parameters, but every test has its limitations and alerts that are not important in a particular case may appear. Conversely, the absence of alerts does not guarantee there are no aspects of the results needing attention. It is up to the individual to critically assess their own results and, if necessary, seek expert advice.

Publication of your CIF in IUCr journals

A basic structural check has been run on your CIF. These basic checks will be run on all CIFs submitted for publication in IUCr journals (*Acta Crystallographica*, *Journal of Applied Crystallography*, *Journal of Synchrotron Radiation*); however, if you intend to submit to *Acta Crystallographica Section C* or *E* or *IUCrData*, you should make sure that full publication checks are run on the final version of your CIF prior to submission.

Publication of your CIF in other journals

Please refer to the *Notes for Authors* of the relevant journal for any special instructions relating to CIF submission.

PLATON version of 06/07/2023; check.def file version of 30/06/2023

Datablock exp_165 - ellipsoid plot

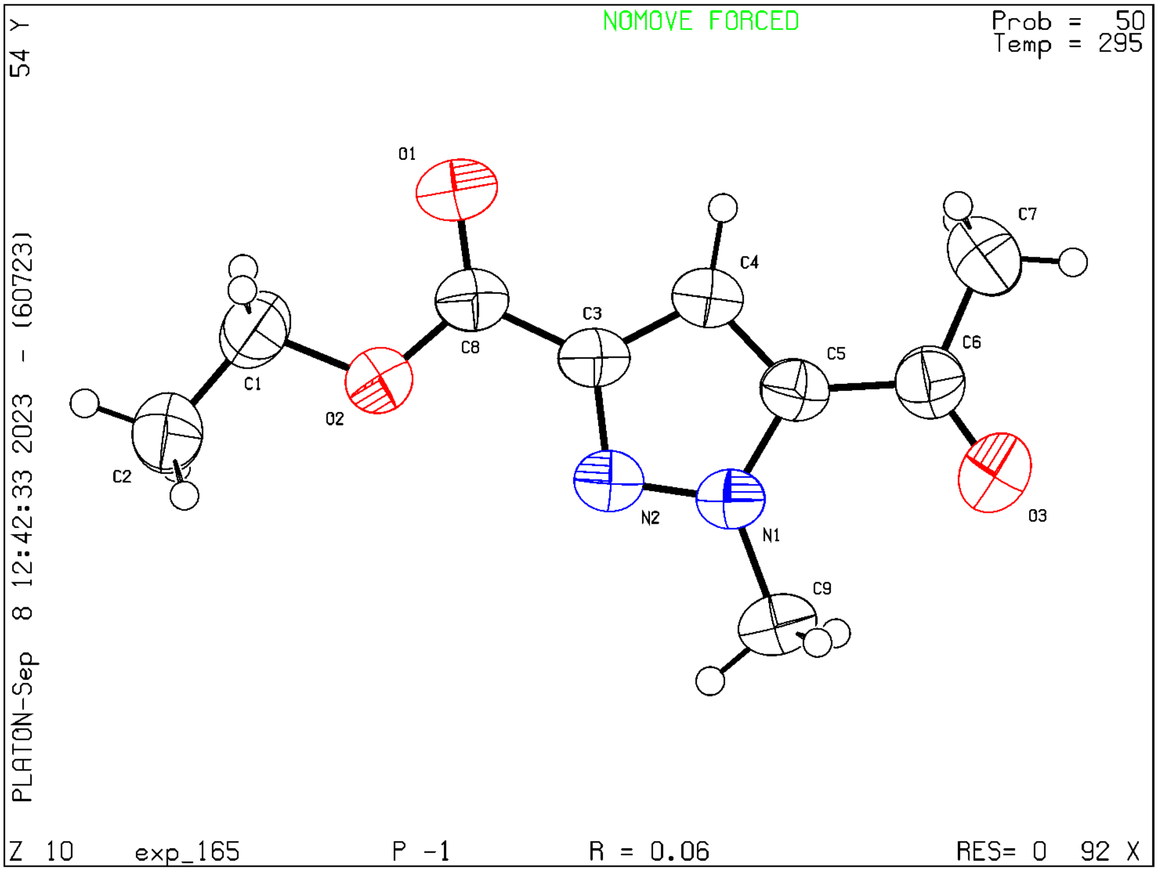

Supplement: Supplementary file 1 [file ijms-24-14234-s001.zip › ijms-2578992-supplementary/SI_ijms-2578992/checkcif 7a.pdf]
