# Supplementary material for: Exploring Three Avenues: Chemo- and Regioselective Transformations of 1,2,4-Triketone Analogs into Pyrazoles and Pyridazinones
Source: Int J Mol Sci. 2023 Sep 18;24(18):14234. doi: 10.3390/ijms241814234 (PMC10531707; doi:10.3390/ijms241814234)

checkCIF/PLATON report

Structure factors have been supplied for datablock(s) exp_1158

THIS REPORT IS FOR GUIDANCE ONLY. IF USED AS PART OF A REVIEW PROCEDURE FOR PUBLICATION, IT SHOULD NOT REPLACE THE EXPERTISE OF AN EXPERIENCED CRYSTALLOGRAPHIC REFEREE.

No syntax errors found. CIF dictionary Interpreting this report

Datablock: exp_1158

```
Bond precision:   C-C = 0.0057 A           Wavelength=0.71073

Cell:           a=4.3326(6)        b=11.619(2)       c=13.963(2)
                alpha=96.867(13)   beta=95.604(12)   gamma=93.747(13)
Temperature:    295 K

                 Calculated                   Reported
Volume           692.41(18)                   692.40(18)
Space group      P -1                         P -1
Hall group       -P 1                         -P 1
Moiety formula   C14 H12 F2 N2 O3             C14 H12 F2 N2 O3
Sum formula      C14 H12 F2 N2 O3             C14 H12 F2 N2 O3
Mr               294.26                       294.26
Dx,g cm-3        1.411                        1.411
Z                2                            2
Mu (mm-1)        0.118                        0.118
F000             304.0                        304.0
F000'            304.20
h,k,lmax         5,16,19                      5,15,18
Nref             3803                         3154
Tmin,Tmax        0.976,0.996                  0.141,1.000
Tmin'            0.945

Correction method= # Reported T Limits: Tmin=0.141 Tmax=1.000
AbsCorr = MULTI-SCAN

Data completeness= 0.829            Theta(max)= 29.410

R(reflections)= 0.0726( 1357)                        wR2(reflections)=
                                                     0.2211( 3154)
S = 0.988                  Npar= 208
```

The following ALERTS were generated. Each ALERT has the format
test-name_ALERT_alert-type_alert-level.
Click on the hyperlinks for more details of the test.

Alert level C

```
PLAT026_ALERT_3_C Ratio Observed / Unique Reflections (too) Low ..        43% Check
PLAT340_ALERT_3_C Low Bond Precision on  C-C Bonds ..............    0.00567 Ang.
PLAT350_ALERT_3_C Short  C-H (X0.96,N1.08A)  C4       - H4       .       0.83 Ang.
PLAT906_ALERT_3_C Large K Value in the Analysis of Variance ......      4.072 Check
PLAT911_ALERT_3_C Missing FCF Refl Between Thmin & STh/L=    0.600         17 Report
```

Alert level G

```
PLAT005_ALERT_5_G No Embedded Refinement Details Found  in the CIF     Please Do !
PLAT910_ALERT_3_G Missing # of FCF Reflection(s) Below Theta(Min).          4 Note
PLAT912_ALERT_4_G Missing # of FCF Reflections Above STh/L=  0.600        619 Note
PLAT978_ALERT_2_G Number C-C Bonds with Positive Residual Density.          0 Info
```

0 **ALERT level A** = Most likely a serious problem - resolve or explain
0 **ALERT level B** = A potentially serious problem, consider carefully
5 **ALERT level C** = Check. Ensure it is not caused by an omission or oversight
4 **ALERT level G** = General information/check it is not something unexpected

0 ALERT type 1 CIF construction/syntax error, inconsistent or missing data
1 ALERT type 2 Indicator that the structure model may be wrong or deficient
6 ALERT type 3 Indicator that the structure quality may be low
1 ALERT type 4 Improvement, methodology, query or suggestion
1 ALERT type 5 Informative message, check

It is advisable to attempt to resolve as many as possible of the alerts in all categories. Often the minor alerts point to easily fixed oversights, errors and omissions in your CIF or refinement strategy, so attention to these fine details can be worthwhile. In order to resolve some of the more serious problems it may be necessary to carry out additional measurements or structure refinements. However, the purpose of your study may justify the reported deviations and the more serious of these should normally be commented upon in the discussion or experimental section of a paper or in the "special_details" fields of the CIF. checkCIF was carefully designed to identify outliers and unusual parameters, but every test has its limitations and alerts that are not important in a particular case may appear. Conversely, the absence of alerts does not guarantee there are no aspects of the results needing attention. It is up to the individual to critically assess their own results and, if necessary, seek expert advice.

Publication of your CIF in IUCr journals

A basic structural check has been run on your CIF. These basic checks will be run on all CIFs submitted for publication in IUCr journals (*Acta Crystallographica*, *Journal of Applied Crystallography*, *Journal of Synchrotron Radiation*); however, if you intend to submit to *Acta Crystallographica Section C* or *E* or *IUCrData*, you should make sure that full publication checks are run on the final version of your CIF prior to submission.

Publication of your CIF in other journals

Please refer to the *Notes for Authors* of the relevant journal for any special instructions relating to CIF submission.

PLATON version of 06/07/2023; check.def file version of 30/06/2023

Datablock exp_1158 - ellipsoid plot

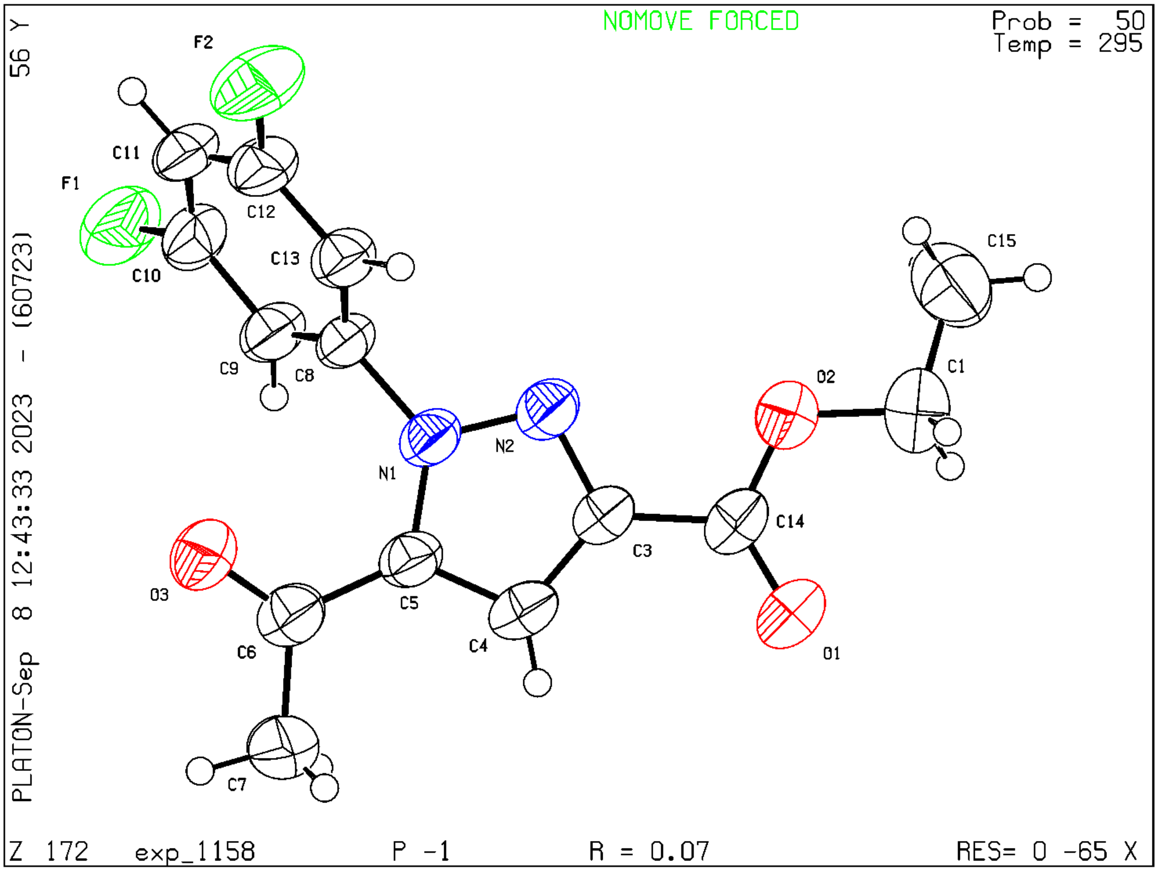

Supplement: Supplementary file 1 [file ijms-24-14234-s001.zip › ijms-2578992-supplementary/SI_ijms-2578992/checkcif 7l.pdf]
