# Supplementary material for: Exploring Three Avenues: Chemo- and Regioselective Transformations of 1,2,4-Triketone Analogs into Pyrazoles and Pyridazinones
Source: Int J Mol Sci. 2023 Sep 18;24(18):14234. doi: 10.3390/ijms241814234 (PMC10531707; doi:10.3390/ijms241814234)

checkCIF/PLATON report

Structure factors have been supplied for datablock(s) exp_206

THIS REPORT IS FOR GUIDANCE ONLY. IF USED AS PART OF A REVIEW PROCEDURE FOR PUBLICATION, IT SHOULD NOT REPLACE THE EXPERTISE OF AN EXPERIENCED CRYSTALLOGRAPHIC REFEREE.

No syntax errors found. CIF dictionary Interpreting this report

Datablock: exp_206

Bond precision:	C-C = 0.0045 A		Wavelength=0.71073
Cell:	a=15.6718(14)	b=6.8466(6)	c=18.4840(16)
	alpha=90	beta=90	gamma=90
Temperature:	295 K		

	Calculated	Reported
Volume	1983.3(3)	1983.3(3)
Space group	P b c a	P b c a
Hall group	-P 2ac 2ab	-P 2ac 2ab
Moiety formula	C10 H10 N2 O S	C10 H10 N2 O S
Sum formula	C10 H10 N2 O S	C10 H10 N2 O S
Mr	206.26	206.26
Dx,g cm-3	1.382	1.382
Z	8	8
Mu (mm-1)	0.292	0.292
F000	864.0	864.0
F000'	865.28	
h,k,lmax	21,9,25	21,9,24
Nref	2779	2652
Tmin,Tmax	0.881,0.924	0.220,1.000
Tmin'	0.869	

Correction method= # Reported T Limits: Tmin=0.220 Tmax=1.000
AbsCorr = MULTI-SCAN

Data completeness= 0.954 Theta(max)= 29.552

R(reflections)= 0.0799(1555) wR2(reflections)= 0.2593(2652)

S = 1.095 Npar= 134

The following ALERTS were generated. Each ALERT has the format
test-name_ALERT_alert-type_alert-level.
Click on the hyperlinks for more details of the test.

Alert level C

```
PLAT084_ALERT_3_C High wR2 Value (i.e. > 0.25) ...................       0.26 Report
PLAT340_ALERT_3_C Low Bond Precision on  C-C Bonds ...............     0.0045 Ang.
PLAT910_ALERT_3_C Missing # of FCF Reflection(s) Below Theta(Min).          5 Note
PLAT911_ALERT_3_C Missing FCF Refl Between Thmin & STh/L=    0.600          8 Report
```

Alert level G

```
PLAT012_ALERT_1_G No       _shelx_res_checksum Found in CIF ......     Please Check
PLAT072_ALERT_2_G SHELXL First  Parameter in WGHT  Unusually Large       0.13 Report
PLAT912_ALERT_4_G Missing # of FCF Reflections Above STh/L=  0.600        109 Note
PLAT913_ALERT_3_G Missing # of Very Strong Reflections in FCF ....          1 Note
PLAT933_ALERT_2_G Number of HKL-OMIT Records in Embedded .res File          5 Note
PLAT978_ALERT_2_G Number C-C Bonds with Positive Residual Density.          0 Info
```

0 **ALERT level A** = Most likely a serious problem - resolve or explain
0 **ALERT level B** = A potentially serious problem, consider carefully
4 **ALERT level C** = Check. Ensure it is not caused by an omission or oversight
6 **ALERT level G** = General information/check it is not something unexpected

1 ALERT type 1 CIF construction/syntax error, inconsistent or missing data
3 ALERT type 2 Indicator that the structure model may be wrong or deficient
5 ALERT type 3 Indicator that the structure quality may be low
1 ALERT type 4 Improvement, methodology, query or suggestion
0 ALERT type 5 Informative message, check

It is advisable to attempt to resolve as many as possible of the alerts in all categories. Often the minor alerts point to easily fixed oversights, errors and omissions in your CIF or refinement strategy, so attention to these fine details can be worthwhile. In order to resolve some of the more serious problems it may be necessary to carry out additional measurements or structure refinements. However, the purpose of your study may justify the reported deviations and the more serious of these should normally be commented upon in the discussion or experimental section of a paper or in the "special_details" fields of the CIF. checkCIF was carefully designed to identify outliers and unusual parameters, but every test has its limitations and alerts that are not important in a particular case may appear. Conversely, the absence of alerts does not guarantee there are no aspects of the results needing attention. It is up to the individual to critically assess their own results and, if necessary, seek expert advice.

Publication of your CIF in IUCr journals

A basic structural check has been run on your CIF. These basic checks will be run on all CIFs submitted for publication in IUCr journals (*Acta Crystallographica*, *Journal of Applied Crystallography*, *Journal of Synchrotron Radiation*); however, if you intend to submit to *Acta Crystallographica Section C* or *E* or *IUCrData*, you should make sure that full publication checks are run on the final version of your CIF prior to submission.

Publication of your CIF in other journals

Please refer to the *Notes for Authors* of the relevant journal for any special instructions relating to CIF submission.

PLATON version of 06/07/2023; check.def file version of 30/06/2023

Datablock exp_206 - ellipsoid plot

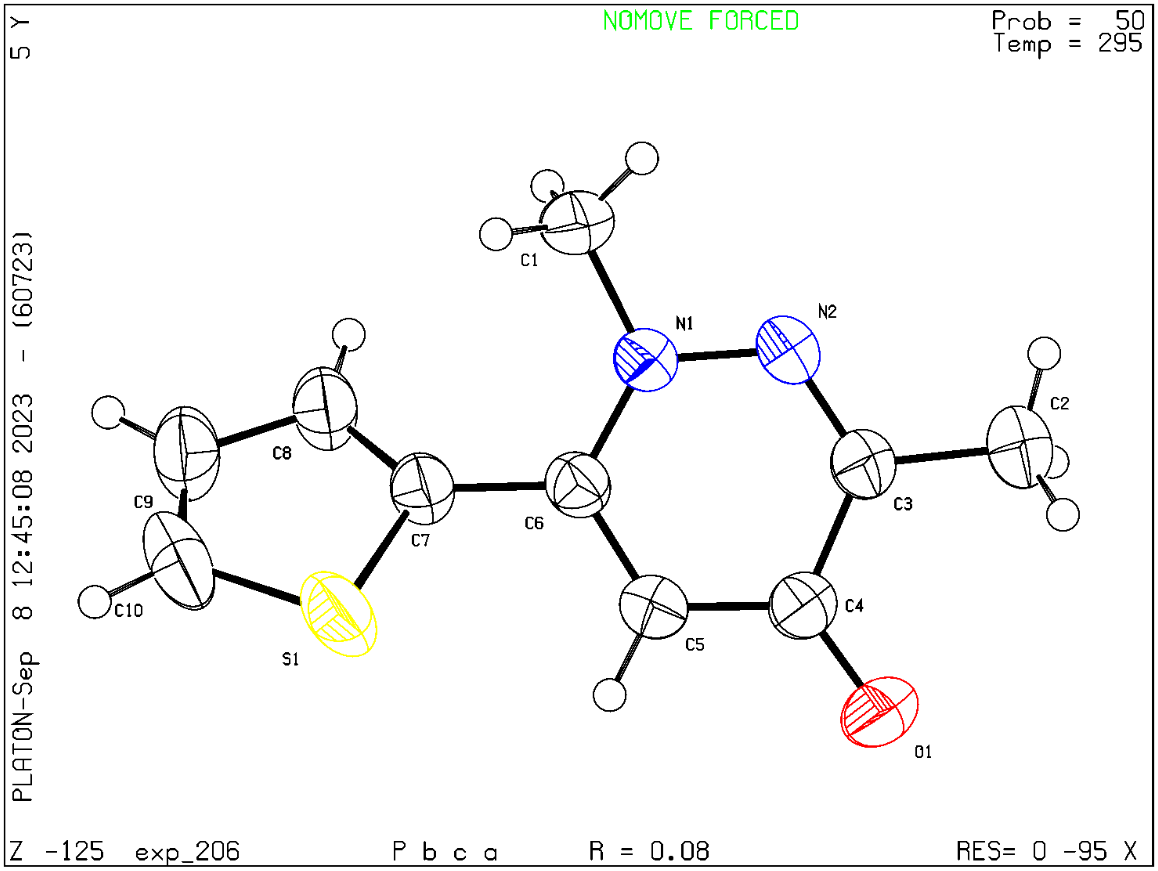

Supplement: Supplementary file 1 [file ijms-24-14234-s001.zip › ijms-2578992-supplementary/SI_ijms-2578992/checkcif 9a.pdf]
