# Supplementary material for: Exploring Three Avenues: Chemo- and Regioselective Transformations of 1,2,4-Triketone Analogs into Pyrazoles and Pyridazinones
Source: Int J Mol Sci. 2023 Sep 18;24(18):14234. doi: 10.3390/ijms241814234 (PMC10531707; doi:10.3390/ijms241814234)

checkCIF/PLATON report

Structure factors have been supplied for datablock(s) exp_184

THIS REPORT IS FOR GUIDANCE ONLY. IF USED AS PART OF A REVIEW PROCEDURE FOR PUBLICATION, IT SHOULD NOT REPLACE THE EXPERTISE OF AN EXPERIENCED CRYSTALLOGRAPHIC REFEREE.

No syntax errors found. CIF dictionary Interpreting this report

Datablock: exp_184

Bond precision:	C-C = 0.0032 A		Wavelength=0.71073
Cell:	a=6.6692(5)	b=16.0059(11)	c=24.8783(16)
	alpha=90	beta=90	gamma=90
Temperature:	295 K		

	Calculated	Reported
Volume	2655.7(3)	2655.7(3)
Space group	P b c a	P b c a
Hall group	-P 2ac 2ab	-P 2ac 2ab
Moiety formula	C15 H12 N2 O S	C15 H12 N2 O S
Sum formula	C15 H12 N2 O S	C15 H12 N2 O S
Mr	268.33	268.33
Dx,g cm-3	1.342	1.342
Z	8	8
Mu (mm-1)	0.236	0.236
F000	1120.0	1120.0
F000'	1121.38	
h,k,lmax	9,23,36	9,21,33
Nref	4254	3627
Tmin,Tmax	0.913,0.956	0.281,1.000
Tmin'	0.897	

Correction method= # Reported T Limits: Tmin=0.281 Tmax=1.000
AbsCorr = MULTI-SCAN

Data completeness= 0.853 Theta(max)= 31.056

R(reflections)= 0.0603(2188) wR2(reflections)= 0.2018(3627)

S = 1.049 Npar= 210

The following ALERTS were generated. Each ALERT has the format
test-name_ALERT_alert-type_alert-level.
Click on the hyperlinks for more details of the test.

Alert level C

```
PLAT242_ALERT_2_C Low    'MainMol' Ueq as Compared to Neighbors of        C1 Check
PLAT906_ALERT_3_C Large K Value in the Analysis of Variance ......     4.894 Check
PLAT910_ALERT_3_C Missing # of FCF Reflection(s) Below Theta(Min).         8 Note
PLAT911_ALERT_3_C Missing FCF Refl Between Thmin & STh/L=    0.600        14 Report
```

Alert level G

```
PLAT002_ALERT_2_G Number of Distance or Angle Restraints on AtSite         9 Note
PLAT003_ALERT_2_G Number of Uiso or Uij Restrained non-H Atoms ...         2 Report
PLAT012_ALERT_1_G No       _shelx_res_checksum Found in CIF ......    Please Check
PLAT171_ALERT_4_G The CIF-Embedded .res File Contains EADP Records         4 Report
PLAT172_ALERT_4_G The CIF-Embedded .res File Contains DFIX Records        14 Report
PLAT186_ALERT_4_G The CIF-Embedded .res File Contains ISOR Records         1 Report
PLAT300_ALERT_4_G Atom Site Occupancy of S1A        Constrained at       0.8 Check
PLAT300_ALERT_4_G Atom Site Occupancy of S1         Constrained at       0.2 Check
PLAT300_ALERT_4_G Atom Site Occupancy of C14A       Constrained at       0.8 Check
PLAT300_ALERT_4_G Atom Site Occupancy of C15A       Constrained at       0.8 Check
PLAT300_ALERT_4_G Atom Site Occupancy of C16A       Constrained at       0.8 Check
PLAT300_ALERT_4_G Atom Site Occupancy of C14        Constrained at       0.2 Check
PLAT300_ALERT_4_G Atom Site Occupancy of C15        Constrained at       0.2 Check
PLAT300_ALERT_4_G Atom Site Occupancy of C16        Constrained at       0.2 Check
PLAT300_ALERT_4_G Atom Site Occupancy of H14A       Constrained at       0.8 Check
PLAT300_ALERT_4_G Atom Site Occupancy of H15A       Constrained at       0.8 Check
PLAT300_ALERT_4_G Atom Site Occupancy of H16A       Constrained at       0.8 Check
PLAT300_ALERT_4_G Atom Site Occupancy of H14        Constrained at       0.2 Check
PLAT300_ALERT_4_G Atom Site Occupancy of H15        Constrained at       0.2 Check
PLAT300_ALERT_4_G Atom Site Occupancy of H16        Constrained at       0.2 Check
PLAT301_ALERT_3_G Main Residue  Disorder .............(Resd  1  )       21% Note
PLAT860_ALERT_3_G Number of Least-Squares Restraints ............         26 Note
PLAT912_ALERT_4_G Missing # of FCF Reflections Above STh/L=  0.600       533 Note
PLAT933_ALERT_2_G Number of HKL-OMIT Records in Embedded .res File        11 Note
PLAT951_ALERT_5_G Calculated (ThMax) and CIF-Reported Kmax Differ          2 Units
PLAT952_ALERT_5_G Calculated (ThMax) and CIF-Reported Lmax Differ.         3 Units
PLAT957_ALERT_1_G Calculated (ThMax) and Actual (FCF) Kmax Differ          2 Units
PLAT958_ALERT_1_G Calculated (ThMax) and Actual (FCF) Lmax Differ.         3 Units
PLAT978_ALERT_2_G Number C-C Bonds with Positive Residual Density.         1 Info
```

0 **ALERT level A** = Most likely a serious problem - resolve or explain
0 **ALERT level B** = A potentially serious problem, consider carefully
4 **ALERT level C** = Check. Ensure it is not caused by an omission or oversight
29 **ALERT level G** = General information/check it is not something unexpected

3 ALERT type 1 CIF construction/syntax error, inconsistent or missing data
5 ALERT type 2 Indicator that the structure model may be wrong or deficient
5 ALERT type 3 Indicator that the structure quality may be low
18 ALERT type 4 Improvement, methodology, query or suggestion
2 ALERT type 5 Informative message, check

It is advisable to attempt to resolve as many as possible of the alerts in all categories. Often the minor alerts point to easily fixed oversights, errors and omissions in your CIF or refinement strategy, so attention to these fine details can be worthwhile. In order to resolve some of the more serious problems it may be necessary to carry out additional measurements or structure refinements. However, the purpose of your study may justify the reported deviations and the more serious of these should normally be commented upon in the discussion or experimental section of a paper or in the "special_details" fields of the CIF. checkCIF was carefully designed to identify outliers and unusual parameters, but every test has its limitations and alerts that are not important in a particular case may appear. Conversely, the absence of alerts does not guarantee there are no aspects of the results needing attention. It is up to the individual to critically assess their own results and, if necessary, seek expert advice.

Publication of your CIF in IUCr journals

A basic structural check has been run on your CIF. These basic checks will be run on all CIFs submitted for publication in IUCr journals (*Acta Crystallographica*, *Journal of Applied Crystallography*, *Journal of Synchrotron Radiation*); however, if you intend to submit to *Acta Crystallographica Section C* or *E* or *IUCrData*, you should make sure that full publication checks are run on the final version of your CIF prior to submission.

Publication of your CIF in other journals

Please refer to the *Notes for Authors* of the relevant journal for any special instructions relating to CIF submission.

PLATON version of 06/07/2023; check.def file version of 30/06/2023

Datablock exp_184 - ellipsoid plot

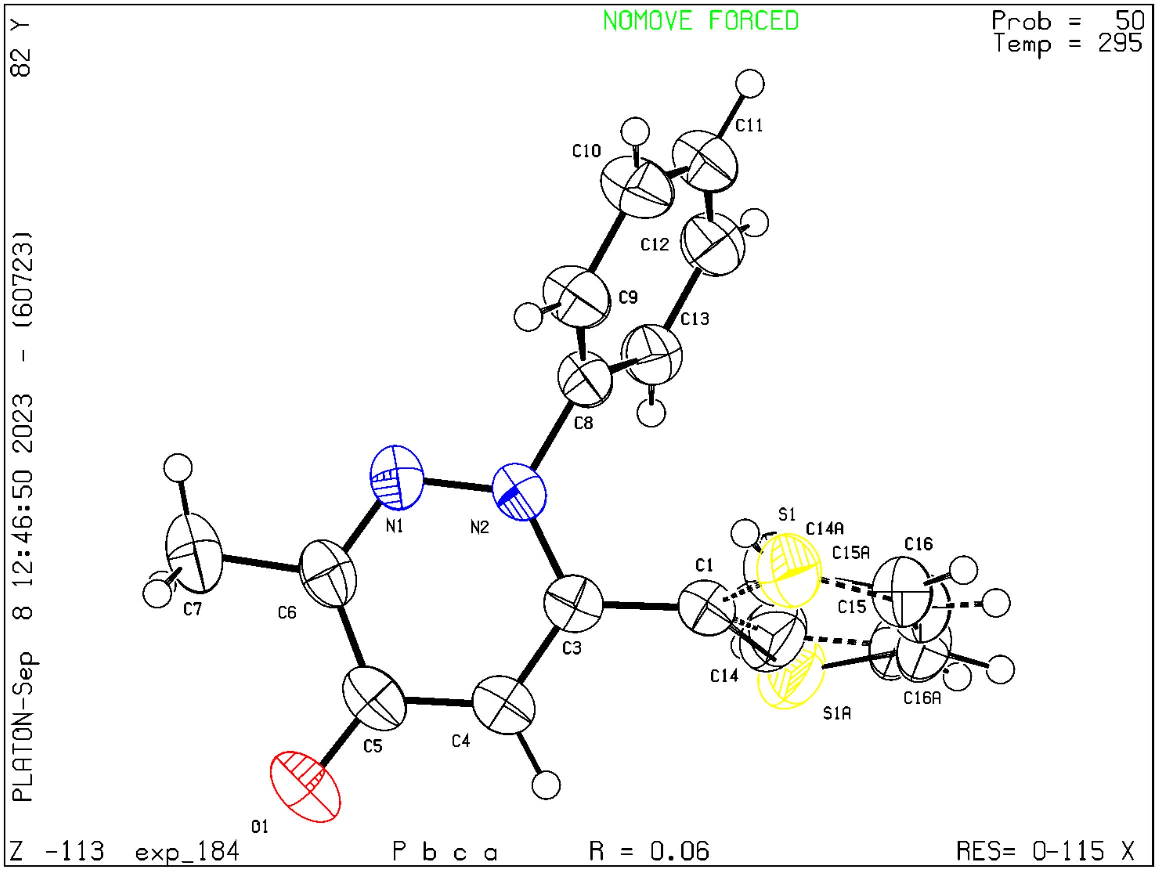

Supplement: Supplementary file 1 [file ijms-24-14234-s001.zip › ijms-2578992-supplementary/SI_ijms-2578992/checkcif 9b.pdf]
