# Supplementary material for: Exploring Three Avenues: Chemo- and Regioselective Transformations of 1,2,4-Triketone Analogs into Pyrazoles and Pyridazinones
Source: Int J Mol Sci. 2023 Sep 18;24(18):14234. doi: 10.3390/ijms241814234 (PMC10531707; doi:10.3390/ijms241814234)
Supplement: Supplementary file 1 [file ijms-24-14234-s001.zip › ijms-2578992-supplementary/SI_ijms-2578992/ijms-2578992-supplementary.pdf]

Supporting information

Exploring Three Avenues: Chemo- and Regioselective Transformations of 1,2,4-Triketone Analogs into Pyrazoles and Pyridazinones

Yulia O. Edilova [1*], Ekaterina A. Osipova [1,2], Pavel A. Slepukhin [1], Victor I. Saloutin [1] and Denis N. Bazhin [1,2*]

[1] Postovsky Institute of Organic Synthesis, Ural Branch of the Russian Academy of Sciences, 620108, Yekaterinburg, Russian Federation

[2] Department of Organic and Biomolecular Chemistry, Ural Federal University named after the First President of Russia B.N. Eltsin, 620002, Yekaterinburg, Russian Federation

*Correspondence: edilova_yuliya@mail.ru (Y.O.E.); dnbazhin@gmail.com (D.N.B.)

Table of contents

Figure S1. ^{1}H NMR spectrum of compound **1**. 5

Figure S2. ^{13}C NMR spectrum of compound **1**. 6

Figure S3. ^{1}H NMR spectrum of compound **2a**. 7

Figure S4. ^{13}C NMR spectrum of compound **2a**. 8

Figure S5. ^{1}H NMR spectrum of compound **2b**. 9

Figure S6. ^{13}C NMR spectrum of compound **2b**. 10

Figure S7. ^{1}H NMR spectrum of compound **2c**. 11

Figure S8. ^{13}C NMR spectrum of compound **2c**. 12

Figure S9. ^{1}H NMR spectrum of compound **3**. 13

Figure S10. ^{13}C NMR spectrum of compound **3**. 14

Figure S11. ^{1}H NMR spectrum of compound **4a**. 15

Figure S12. ^{13}C NMR spectrum of compound **4a**. 16

Figure S13. ^{1}H NMR spectrum of compound **4b**. 17

Figure S14. ^{13}C NMR spectrum of compound **4b**. 18

Figure S15. ^{1}H NMR spectrum of compound **6**. 19

Figure S16. ^{13}C NMR spectrum of compound **6**. 20

Figure S17. ^{1}H NMR spectrum of compound **7a**. 21

Figure S18. ^{13}C NMR spectrum of compound **7a**. 22

Figure S19. ^{1}H NMR spectrum of compound **7b**. 23

Figure S20. ^{13}C NMR spectrum of compound **7b**. 24

Figure S21. ^{1}H NMR spectrum of compound **7c**. 25
Figure S22. ^{13}C NMR spectrum of compound **7c.** 26
Figure S23. ^{1}H NMR spectrum of compound **7d**. 27
Figure S24. ^{13}C NMR spectrum of compound **7d**. 28
Figure S25. ^{19}F NMR spectrum of compound **7d**. 29
Figure S26. ^{1}H NMR spectrum of compound **7e**. 30
Figure S27. ^{13}C NMR spectrum of compound **7e**. 31
Figure S28. ^{19}F NMR spectrum of compound **7e**. 32
Figure S29. ^{1}H NMR spectrum of compound **7f**. 33
Figure S30. ^{13}C NMR spectrum of compound **7f**. 34
Figure S31. ^{1}H NMR spectrum of compound **7g**. 35
Figure S32. ^{13}C NMR spectrum of compound **7g**. 36
Figure S33. ^{1}H NMR spectrum of compound **7h**. 37
Figure S34. ^{13}C NMR spectrum of compound **7h**. 38
Figure S35. ^{19}F NMR spectrum of compound **7h**. 39
Figure S36. ^{1}H NMR spectrum of compound **7i**. 40
Figure S37. ^{13}C NMR spectrum of compound **7i**. 41
Figure S38. ^{1}H NMR spectrum of compound **7j**. 42
Figure S39. ^{13}C NMR spectrum of compound **7j**. 43
Figure S40. ^{19}F NMR spectrum of compound **7j.** 44
Figure S41. ^{1}H NMR spectrum of compound **7k**. 45
Figure S42. ^{13}C NMR spectrum of compound **7k**. 46
Figure S43. ^{1}H NMR spectrum of compound **7l**. 47
Figure S44. ^{13}C NMR spectrum of compound **7l**. 48
Figure S45. ^{19}F NMR spectrum of compound **7l**. 49
Figure S46. ^{1}H NMR spectrum of compound **8**. 50
Figure S47. ^{13}C NMR spectrum of compound **8**. 51
Figure S48. ^{1}H NMR spectrum of compound **9a**. 52
Figure S49. ^{13}C NMR spectrum of compound **9a.** 53
Figure S50. 1H NMR spectrum of compound **9b**. 54
Figure S51. ^{13}C NMR spectrum of compound **9b**. 55
Figure S52. ^{1}H NMR spectrum of compound **10a**. 56
Figure S53. ^{13}C NMR spectrum of compound **10a**. 57
Figure S54. ^{1}H NMR spectrum of compound **10b**. 58
Figure S55. ^{13}C NMR spectrum of compound **10b**. 59
Figure S56. ^{1}H NMR spectrum of compound **11**. 60

Figure S57. ^{13}C NMR spectrum of compound **11**. 61
Figure S58. ^{1}H NMR spectrum of compound **12**. 62
Figure S59. ^{13}C NMR spectrum of compound **12**. 63
Figure S60. ^{1}H NMR spectrum of compound **13a**. 64
Figure S61. ^{13}C NMR spectrum of compound **13a**. 65
Figure S62. ^{1}H NMR spectrum of compound **13b**. 66
Figure S63. ^{13}C NMR spectrum of compound **13b**. 67
Figure S64. ^{1}H NMR spectrum of compound **14**. 68
Figure S65. ^{13}C NMR spectrum of compound **14**. 69
Figure S66. ^{1}H NMR spectrum of compound **15a**. 70
Figure S67. ^{13}C NMR spectrum of compound **15a**. 71
Figure S68. ^{1}H NMR spectrum of compound **15b**. 72
Figure S69. ^{13}C NMR spectrum of compound **15b**. 73
Figure S70. ^{1}H NMR spectrum of compound **16**. 74
Figure S71. ^{1}H NMR spectrum of compound **17**. 75
Figure S72. ^{13}C NMR spectrum of compound **17**. 76
Figure S73. ^{1}H NMR spectrum of compound **18a**. 77
Figure S74. ^{13}C NMR spectrum of compound **18a**. 78
Figure S75. ^{1}H NMR spectrum of compound **18b**. 79
Figure S76. ^{13}C NMR spectrum of compound **18b**. 80
Figure S77. ^{1}H NMR spectrum of compound **18c**. 81
Figure S78. ^{13}C NMR spectrum of compound **18c**. 82
Figure S79. ^{1}H NMR spectrum of compound **19a**. 83
Figure S80. ^{13}C NMR spectrum of compound **19a**. 84
Figure S81. ^{1}H NMR spectrum of compound **19b**. 85
Figure S82. ^{13}C NMR spectrum of compound **19b**. 86
Figure S83. ^{1}H NMR spectrum of compound **19c**. 87
Figure S84. ^{13}C NMR spectrum of compound **19c**. 88
Figure S85. ^{1}H NMR spectrum of compound **20a**. 89
Figure S86 ^{13}C NMR spectrum of compound **20a**. 90
Figure S87. ^{1}H NMR spectrum of compound **20b**. 91
Figure S88. ^{13}C NMR spectrum of compound **20b**. 92
Figure S89. ^{1}H NMR spectrum of compound **20c**. 93
Figure S90. ^{13}C NMR spectrum of compound **20c**. 94
Figure S91. ^{1}H NMR spectrum of compound **21a**. 95
Figure S92. ^{13}C NMR spectrum of compound **21a**. 96

Figure S93. ¹H NMR spectrum of compound **21b**. 97
Figure S94. ¹³C NMR spectrum of compound **21b**. 98
Figure S95. ¹H NMR spectrum of compound **21c**. 99
Figure S96. ¹³C NMR spectrum of compound **21c**. 100
Figure S97. ¹H NMR spectrum of compound **22.** 101
Figure S98. ¹³C NMR spectrum of compound **22**. 102
Figure S99. Crystal packing of diketone **2c** along *a* axis. 103
Figure S100. Crystal packing of furanone **3** along *b* axis. 103
Figure S101. Crystal packing of furanone **5**. 104
Figure S102. Crystal packing of pyrazole **7a** along *a* (**a**) and *c* (**b**) axis. 105
Figure S103. Crystal packing of pyrazole **7l** along *a* (**a**) and *b* (**b**) axis. 106
Figure S104**.** Crystal packing of pyridazinone **9a** along *a* (**a**), *b* (**b**) and *c* (**c**) axis. 107
Figure S105. Crystal packing of pyridazinone **9b** along *a* axis. 108
Figure S106. Crystal packing of pyrazole **12** along *a* (**a**) and *b* (**b**) axis. 109
Figure S107. Crystal packing of pyridazinone **14** along *a* axis. 110
Figure S108. Crystal packing of pyrazole **15b** along *a* (**a**) and *b* (**b**) axis. 111
Figure S109. Crystal packing of pyrazole **21b** along *c* axis. 111
Table S1. Selected bond distances (Å) in diketone **2c**. 112
Table S2. Selected bond distances (Å) in furanones **3** and **5**. 112
Table S3. Dihedral angles between the azaheterocyclic and aryl planes in the crystals of compounds **7l**, **9a**, **9b**, **12**, **14**, **15b**, **21b**. 112
Table S4. Selected bond distances in pyrazoles **7a**, **7l**, **12**, **15b**, **21b**. 112
Table S5. Selected bond distances in pyridazinones **9a**, **9b**, **14**. 113
Figure S110. H-contacts in the crystal packing of **2c**. 114
Figure S111. H-contacts in the crystal packing of **3**. 114
Figure S112. Intermolecular hydrogen bonds in the crystal packing of **5**. 115
Figure S113. H-contacts in the crystal packing of **7a**. 115
Figure S114. H-contacts in the crystal packing of **7l**. 116
Figure S115. H-contacts in the crystal packing of **9a**. 116
Figure S116. H-contacts in the crystal packing of **9b**. 117
Figure S117. H-contacts in the crystal packing of **12**. 117
Figure S118. H-contacts in the crystal packing of **14**. 118
Figure S119. H-contacts in the crystal packing of **15b**. 118
Figure S120. H-contacts in the crystal packing of **21b**. 119
Table S6. Intermolecular hydrogen bonds and hydrogen-contacts geometry (distances in Å, angles in degrees) in **2c, 3, 5, 7l, 9a, 9b, 12, 14, 15b, 21b**. 119

Figure S1. ^{1}H NMR spectrum of compound **1**.

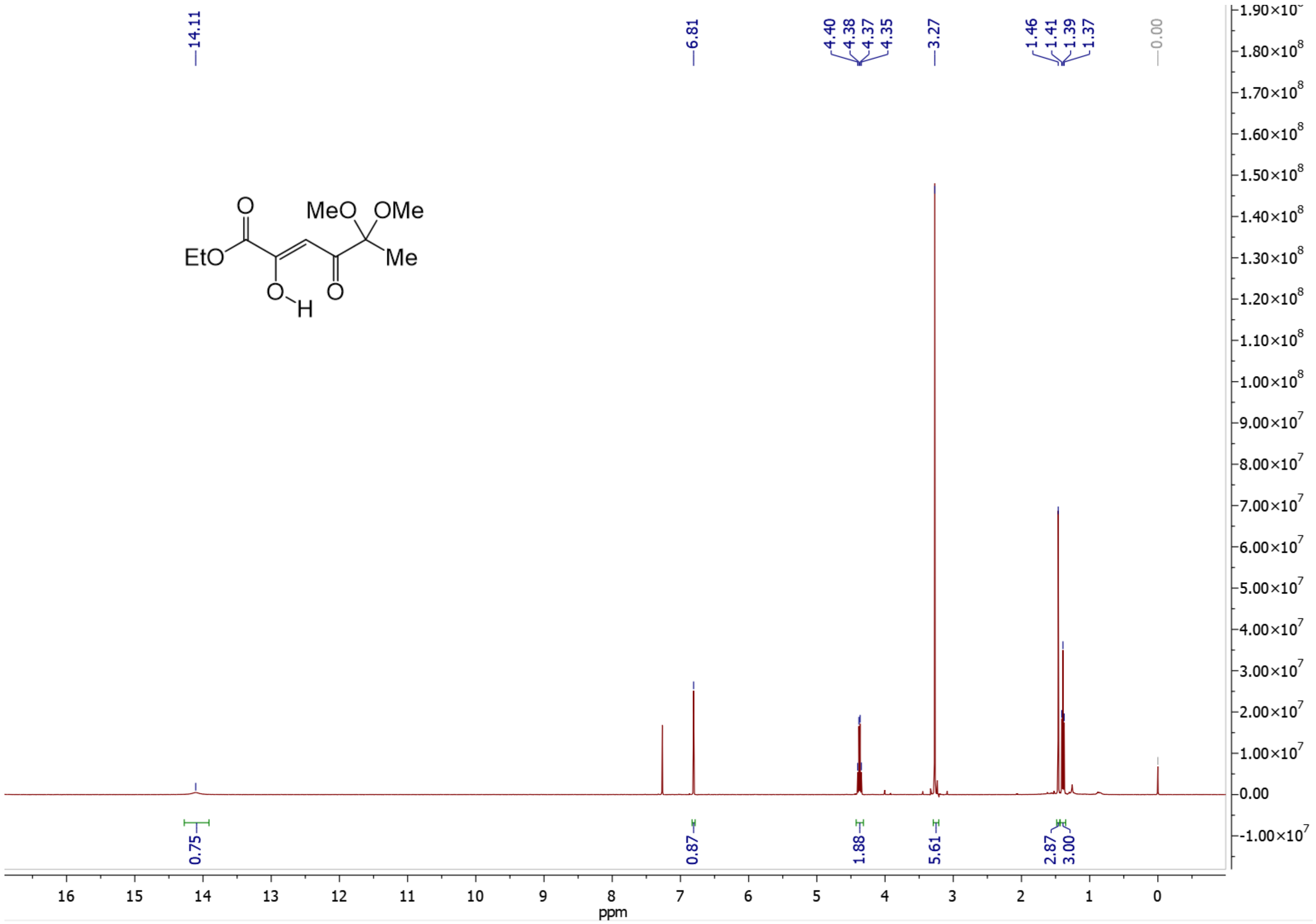

Figure S2. ^{13}C NMR spectrum of compound **1**.

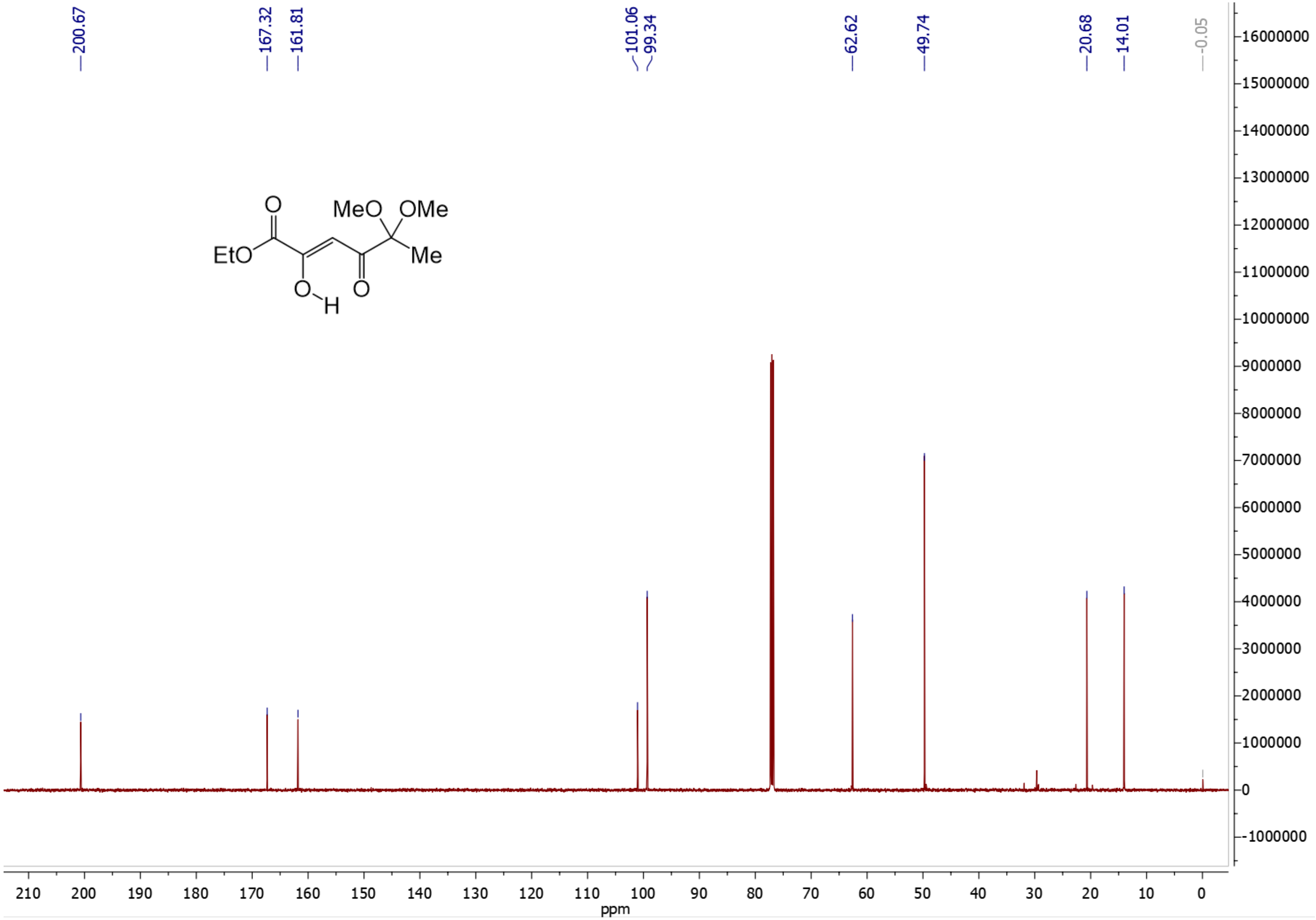

Figure S3. ^{1}H NMR spectrum of compound **2a**.

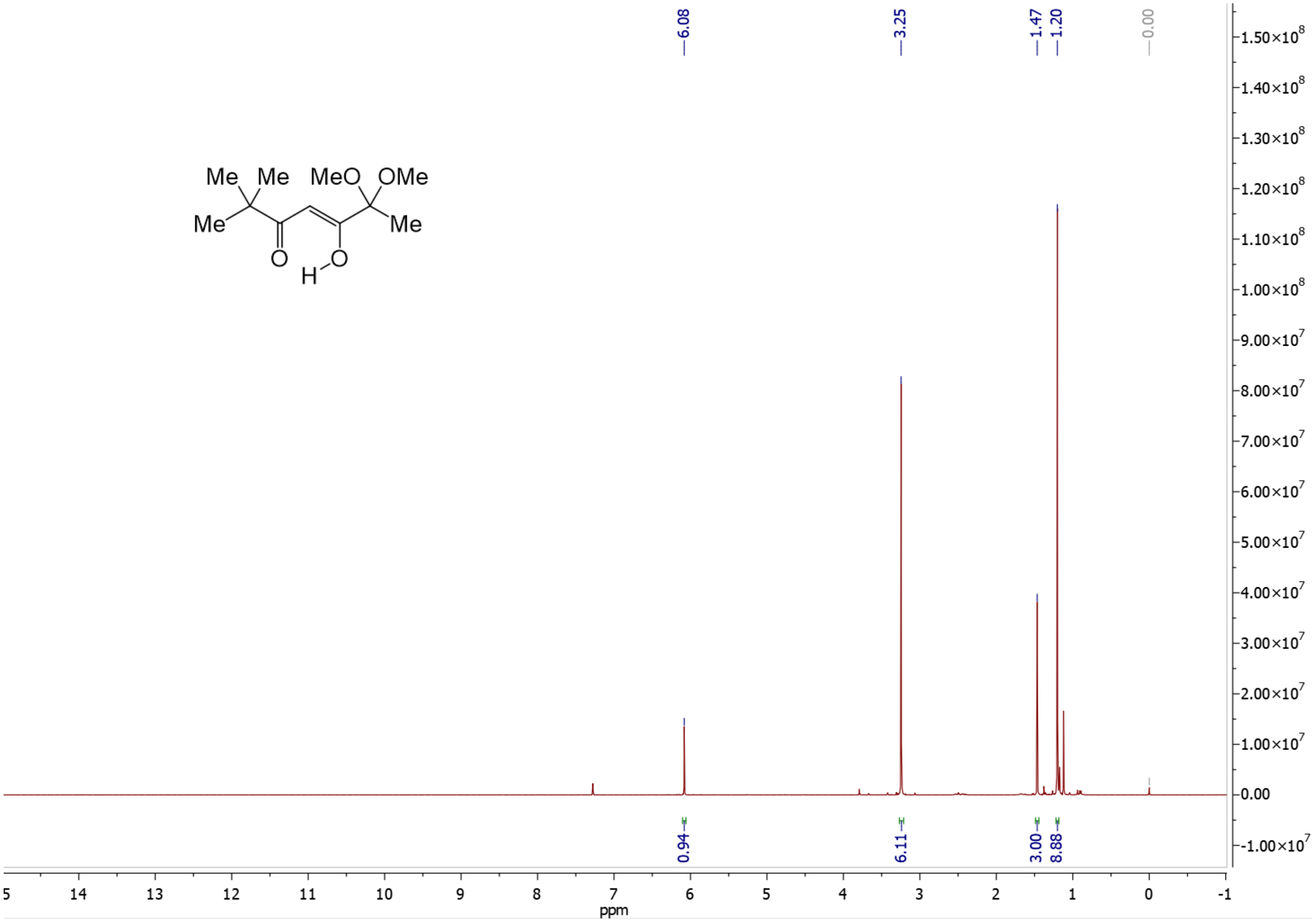

Figure S4. ^{13}C NMR spectrum of compound **2a**.

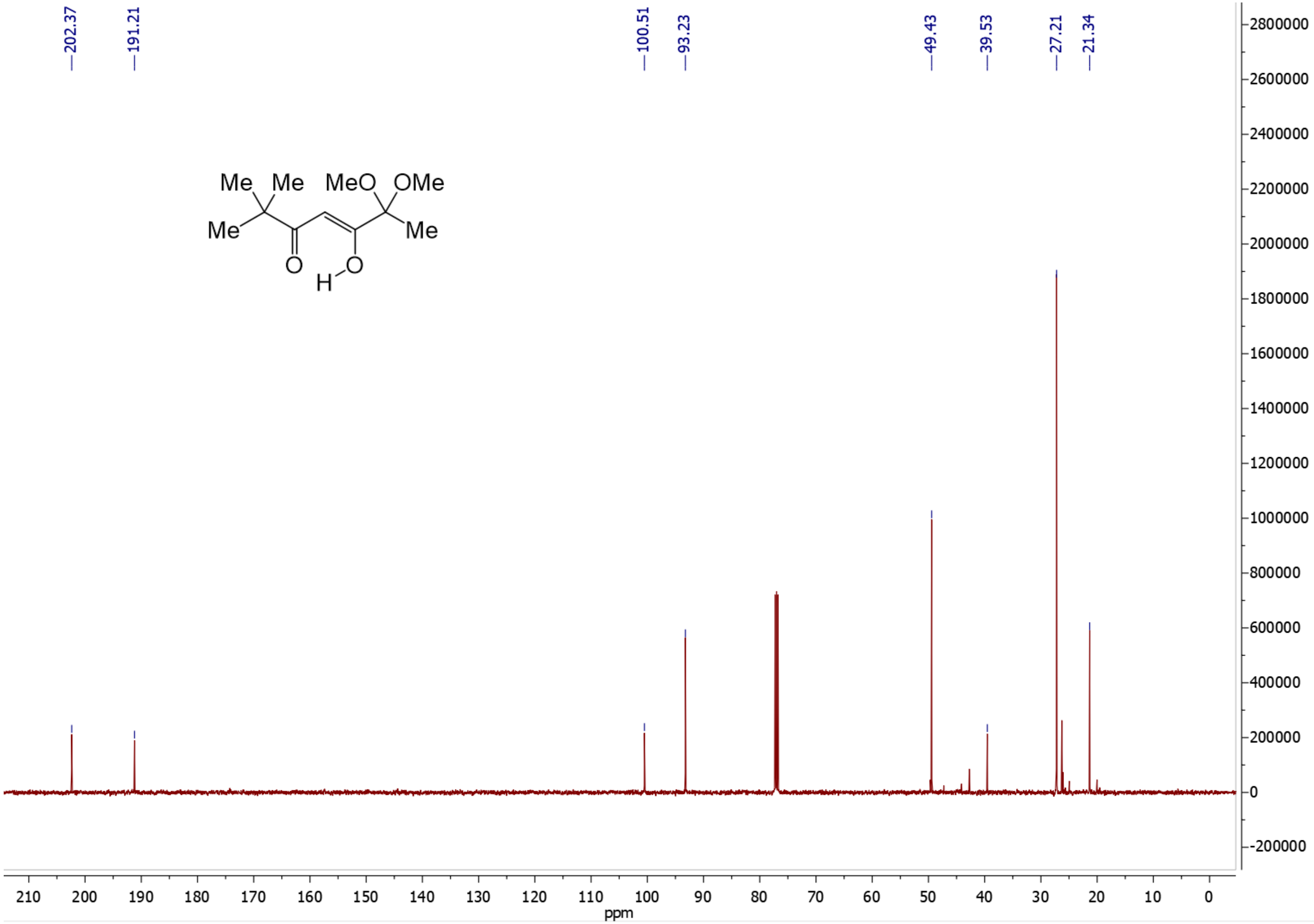

Figure S5. ^{1}H NMR spectrum of compound **2b**.

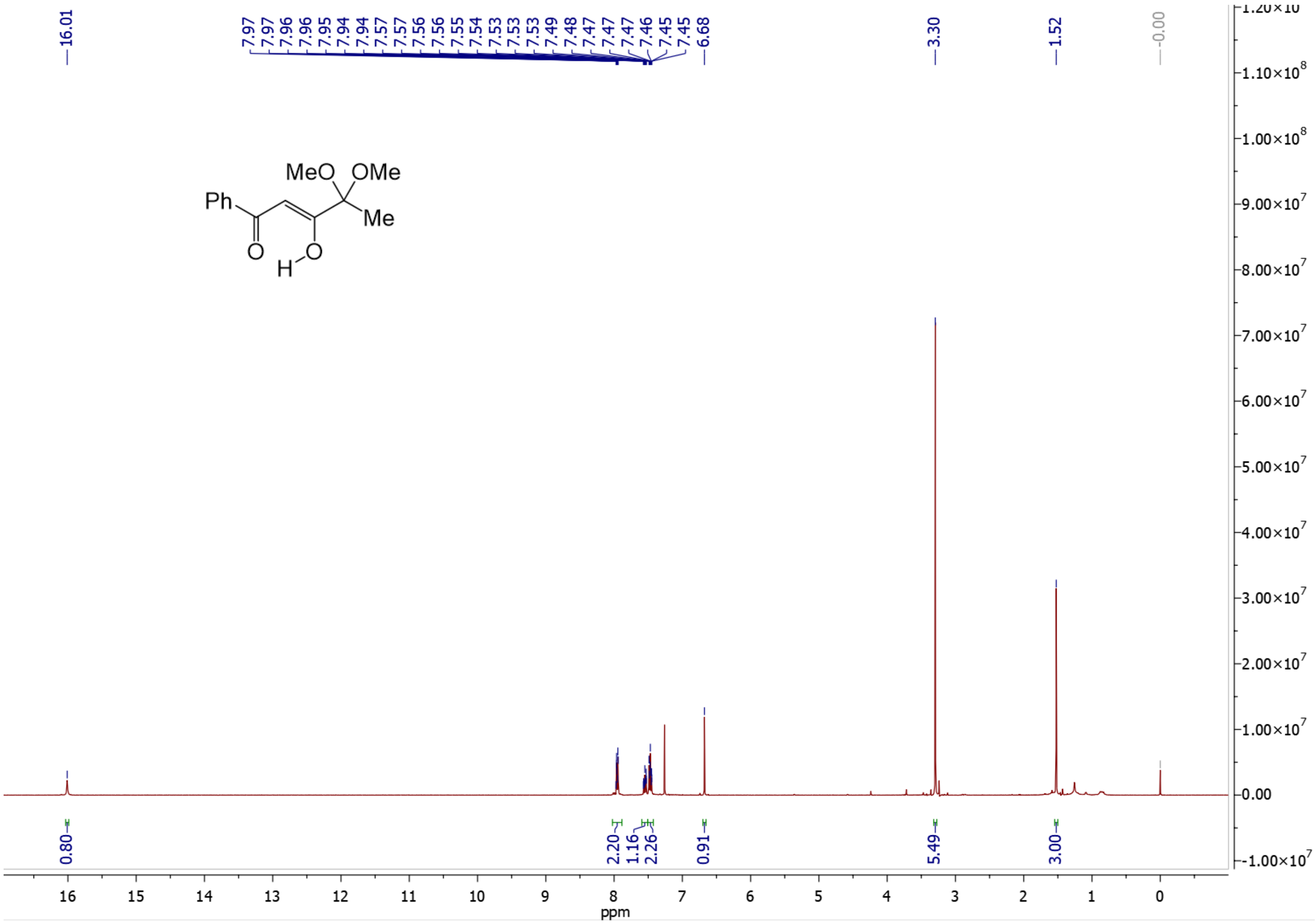

Figure S6. ^{13}C NMR spectrum of compound **2b**.

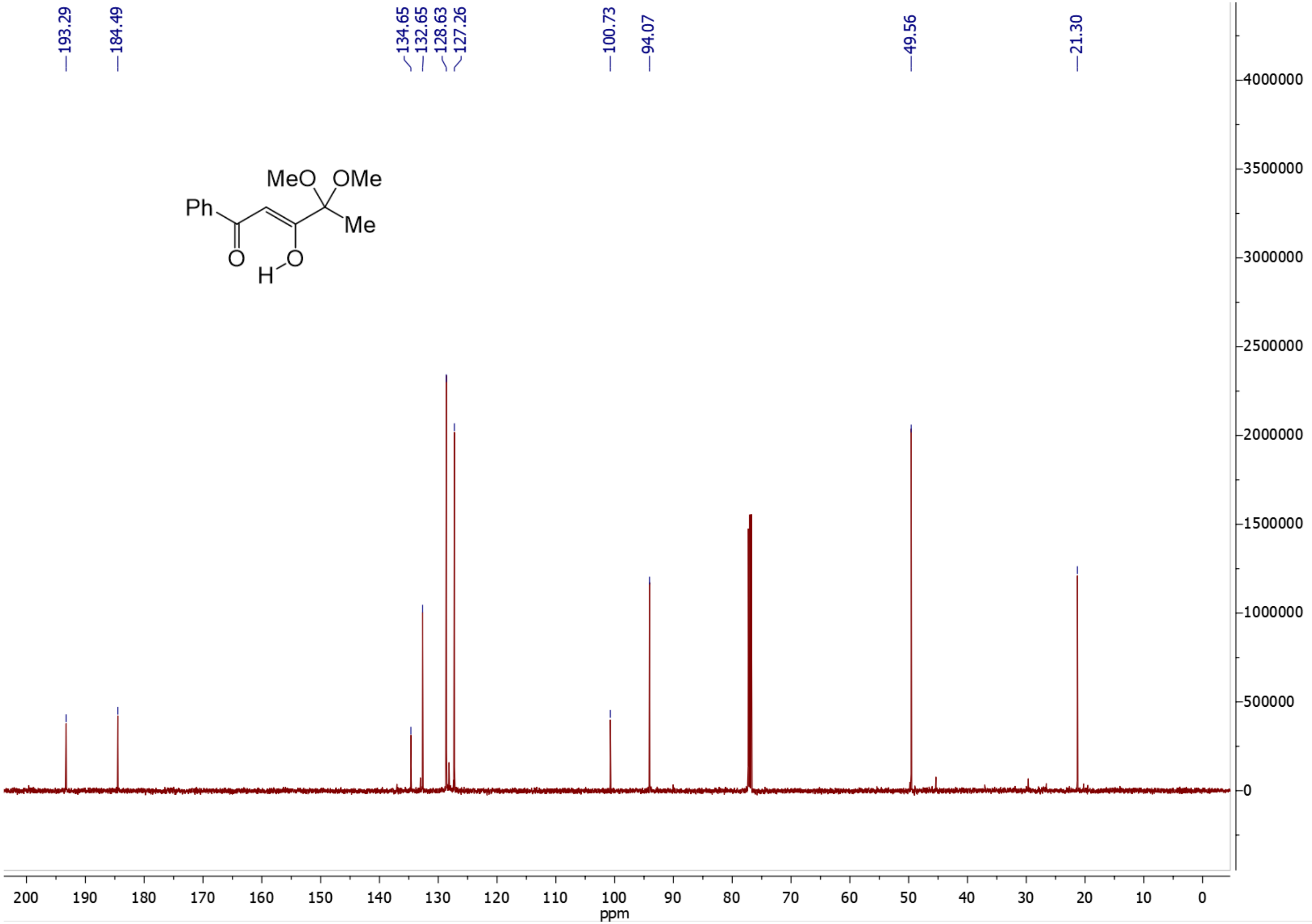

Figure S7. ^{1}H NMR spectrum of compound **2c**.

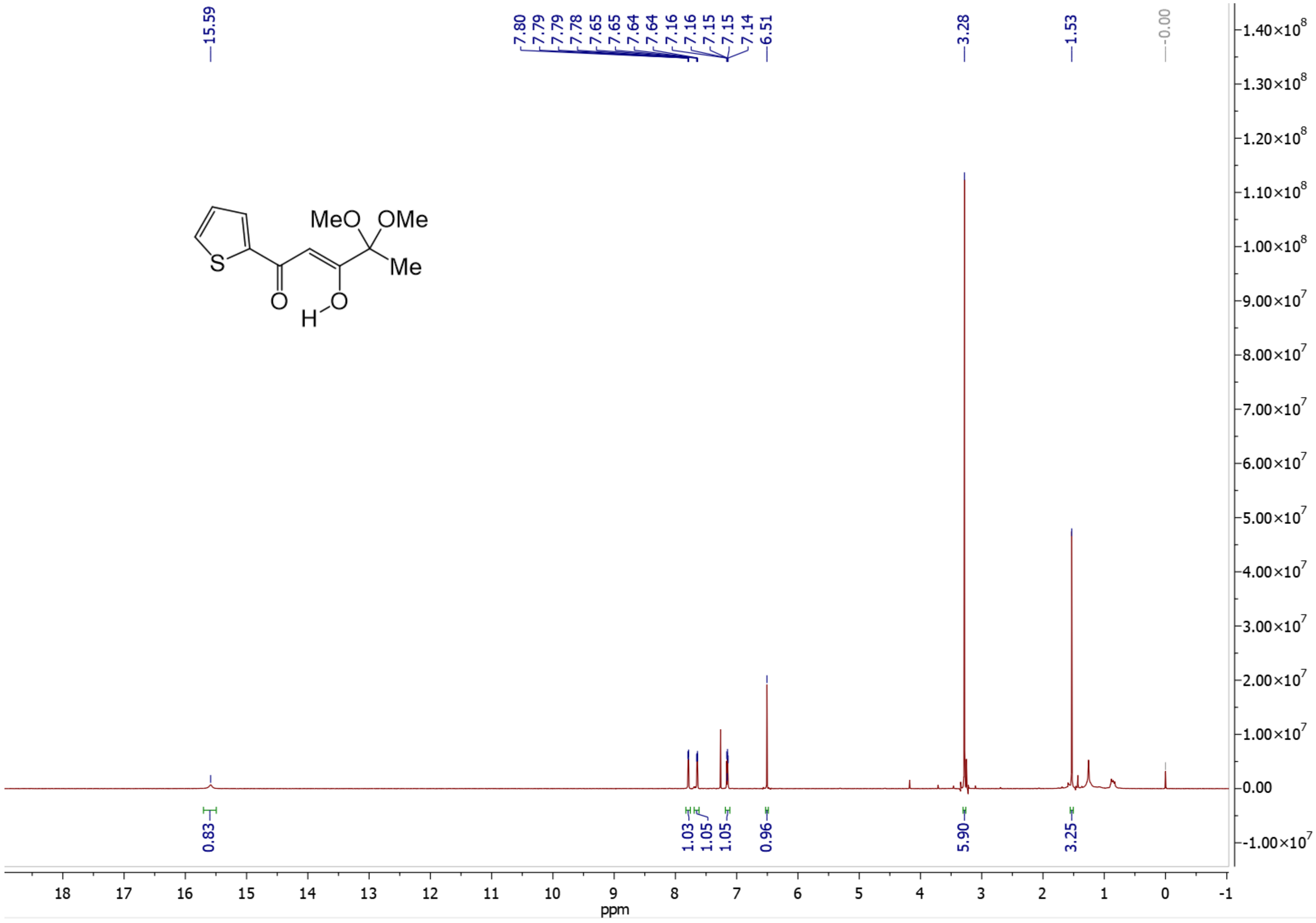

Figure S8. ^{13}C NMR spectrum of compound **2c**.

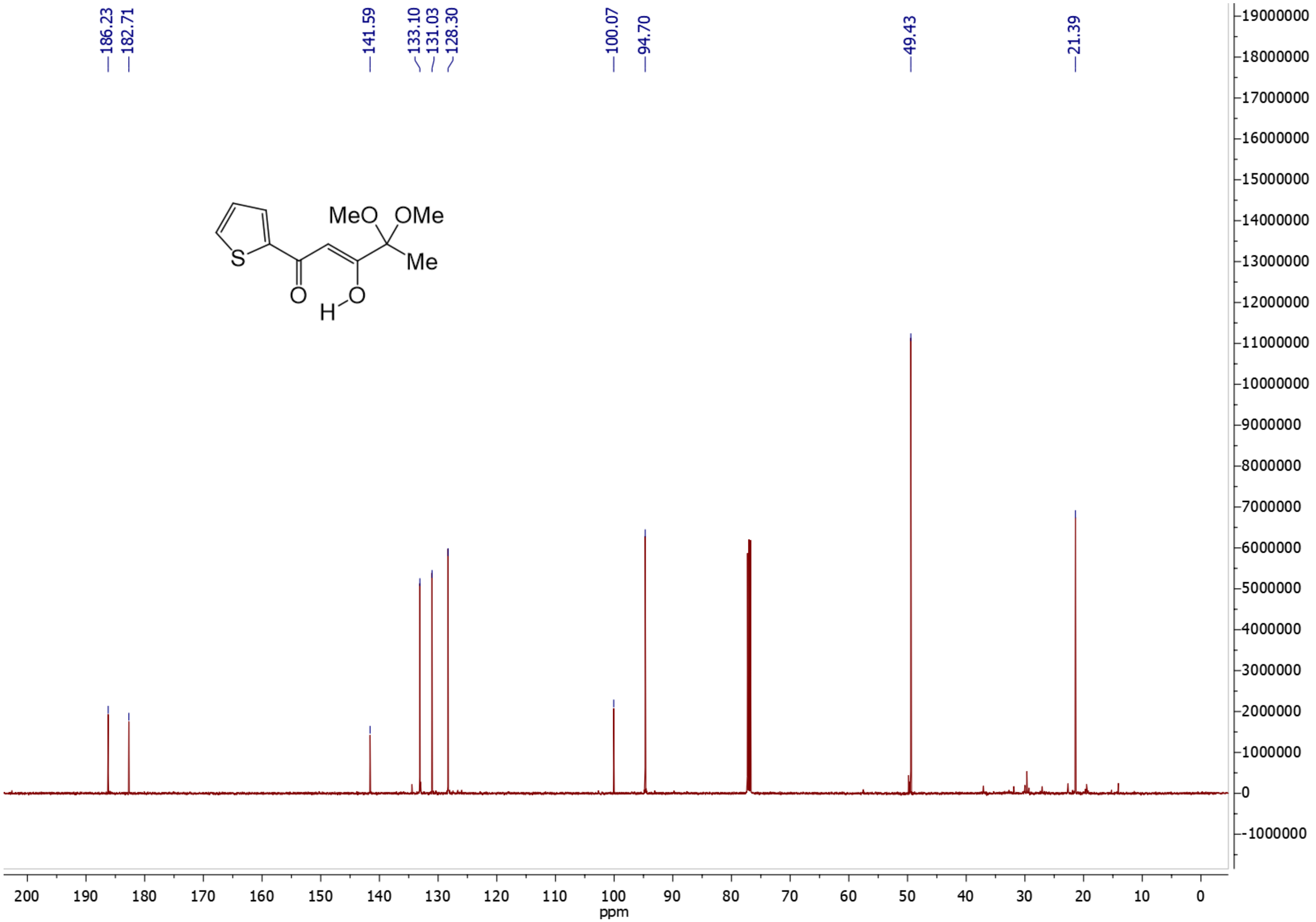

Figure S9. ^{1}H NMR spectrum of compound **3**.

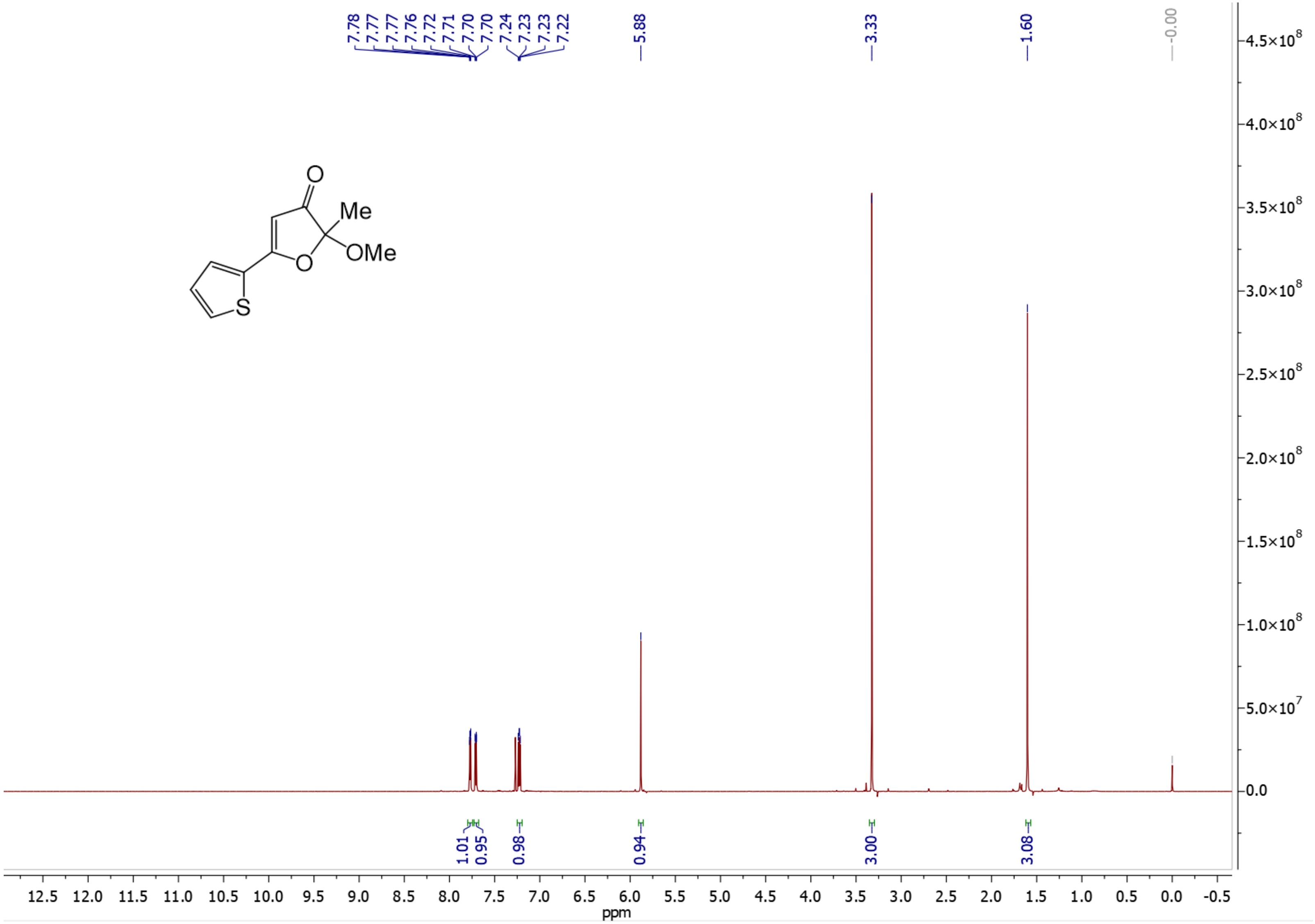

Figure S10. ^{13}C NMR spectrum of compound **3**.

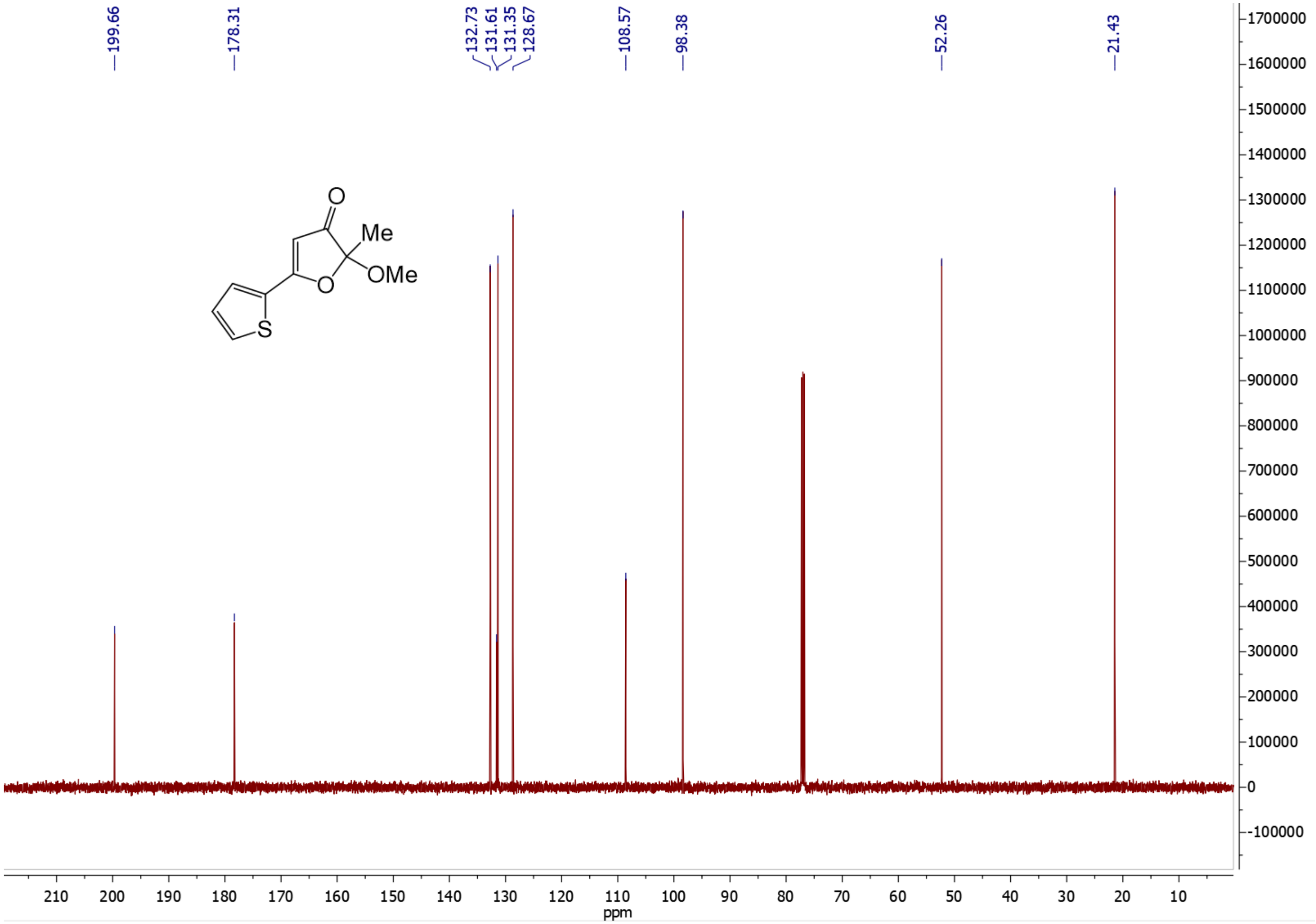

Figure S11. ^{1}H NMR spectrum of compound **4a**.

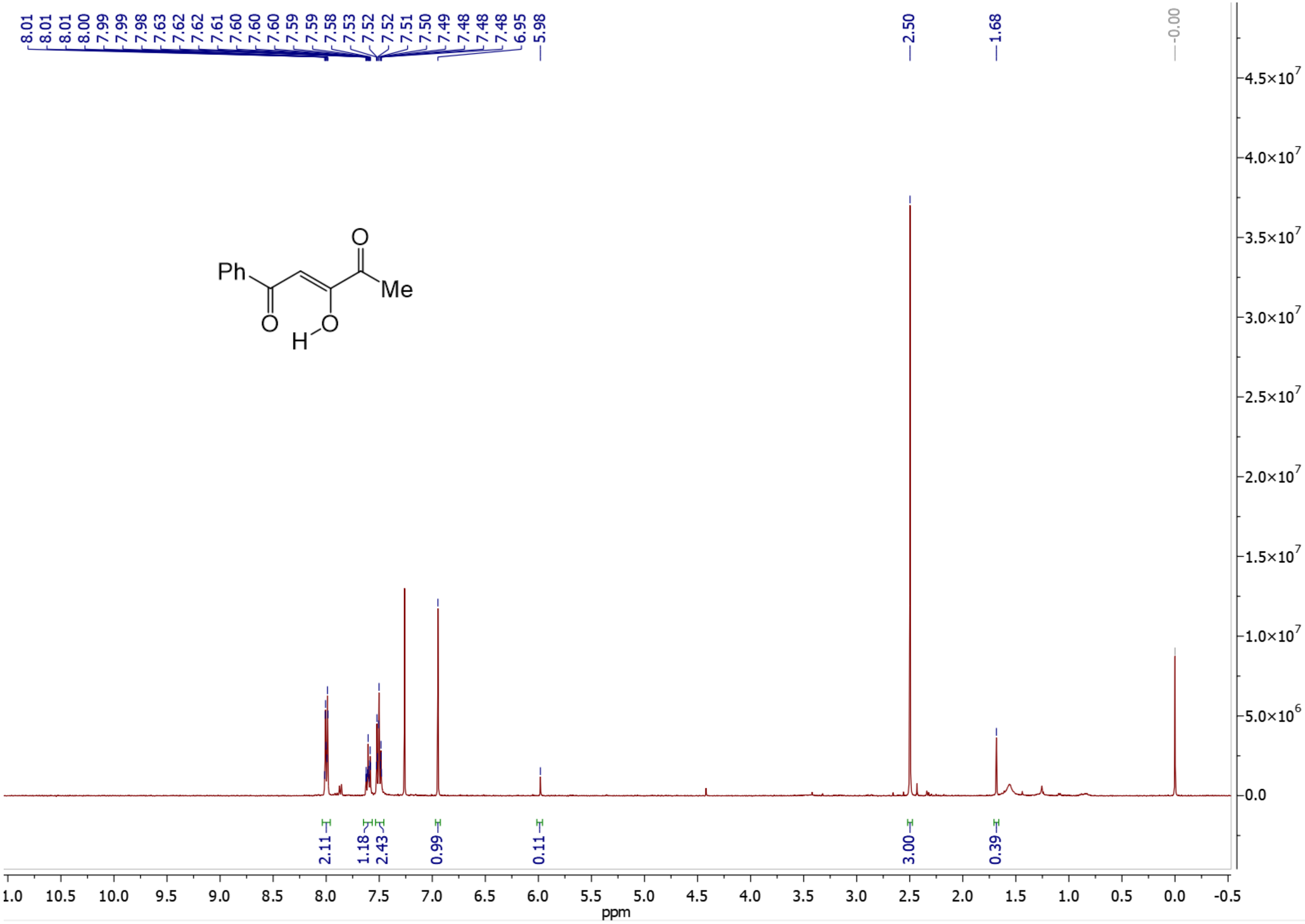

Figure S12. ^{13}C NMR spectrum of compound **4a**.

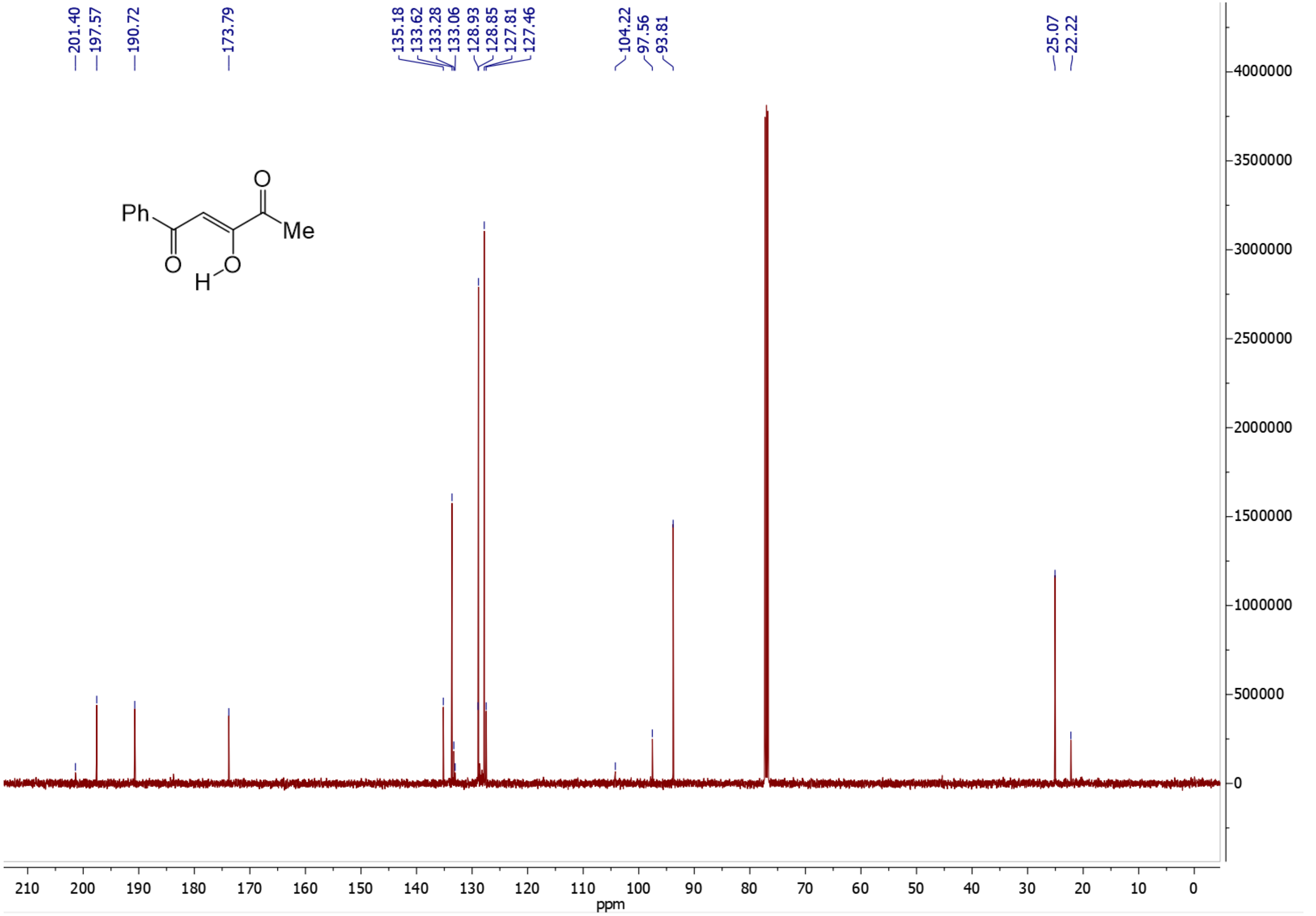

Figure S13. ^{1}H NMR spectrum of compound **4b**.

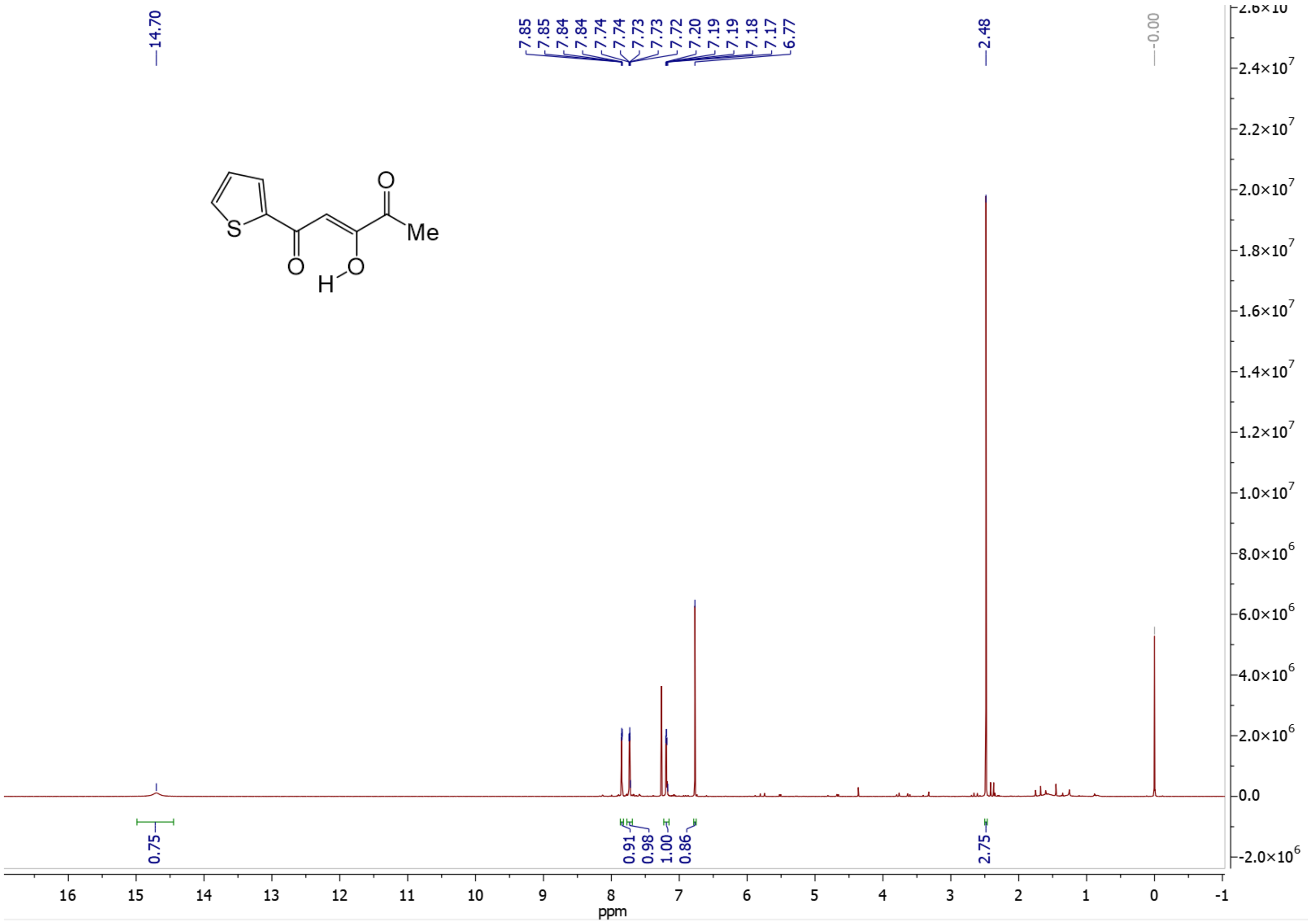

Figure S14. ^{13}C NMR spectrum of compound **4b**.

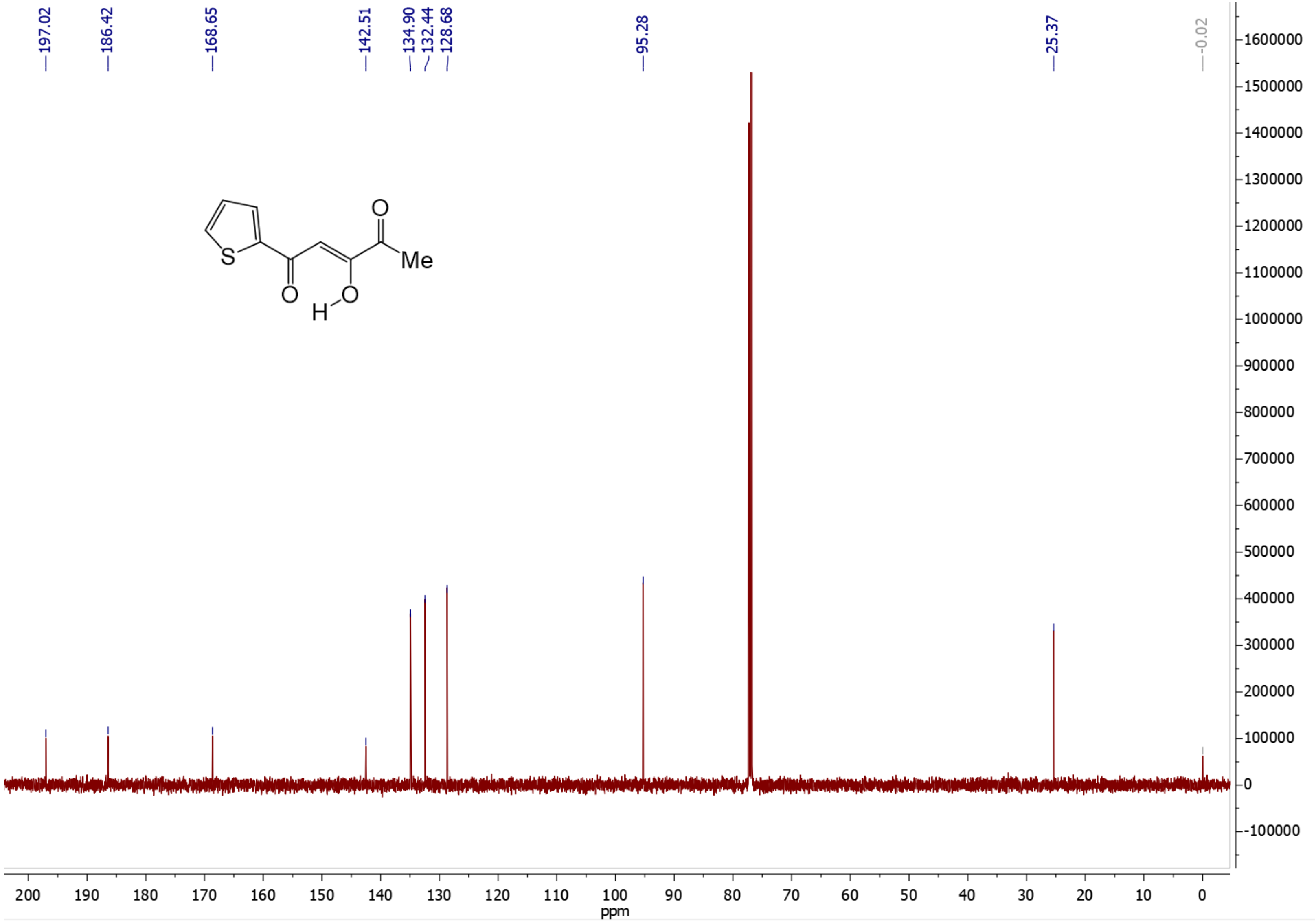

Figure S15. ^{1}H NMR spectrum of compound **6**.

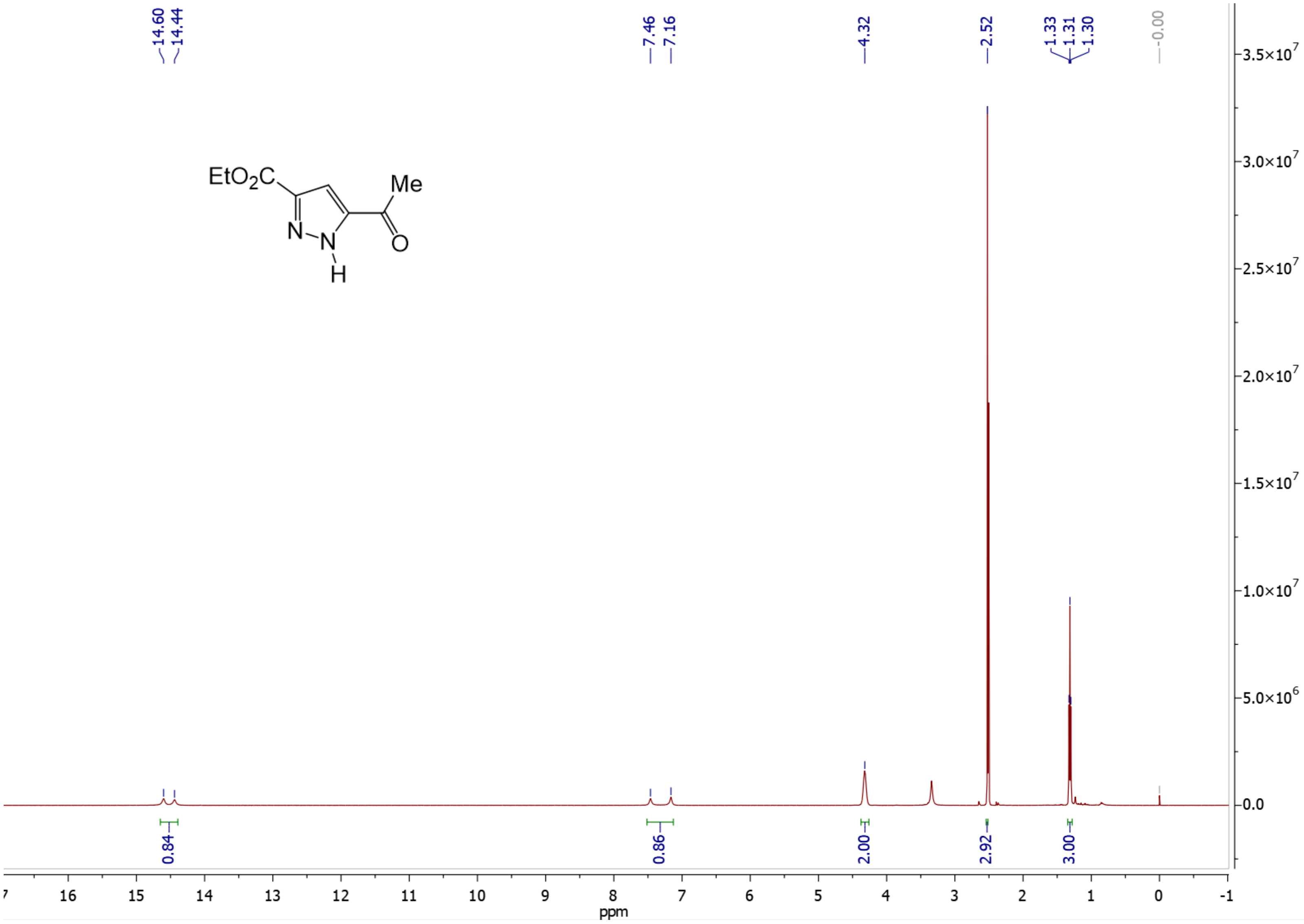

Figure S16. ^{13}C NMR spectrum of compound **6**.

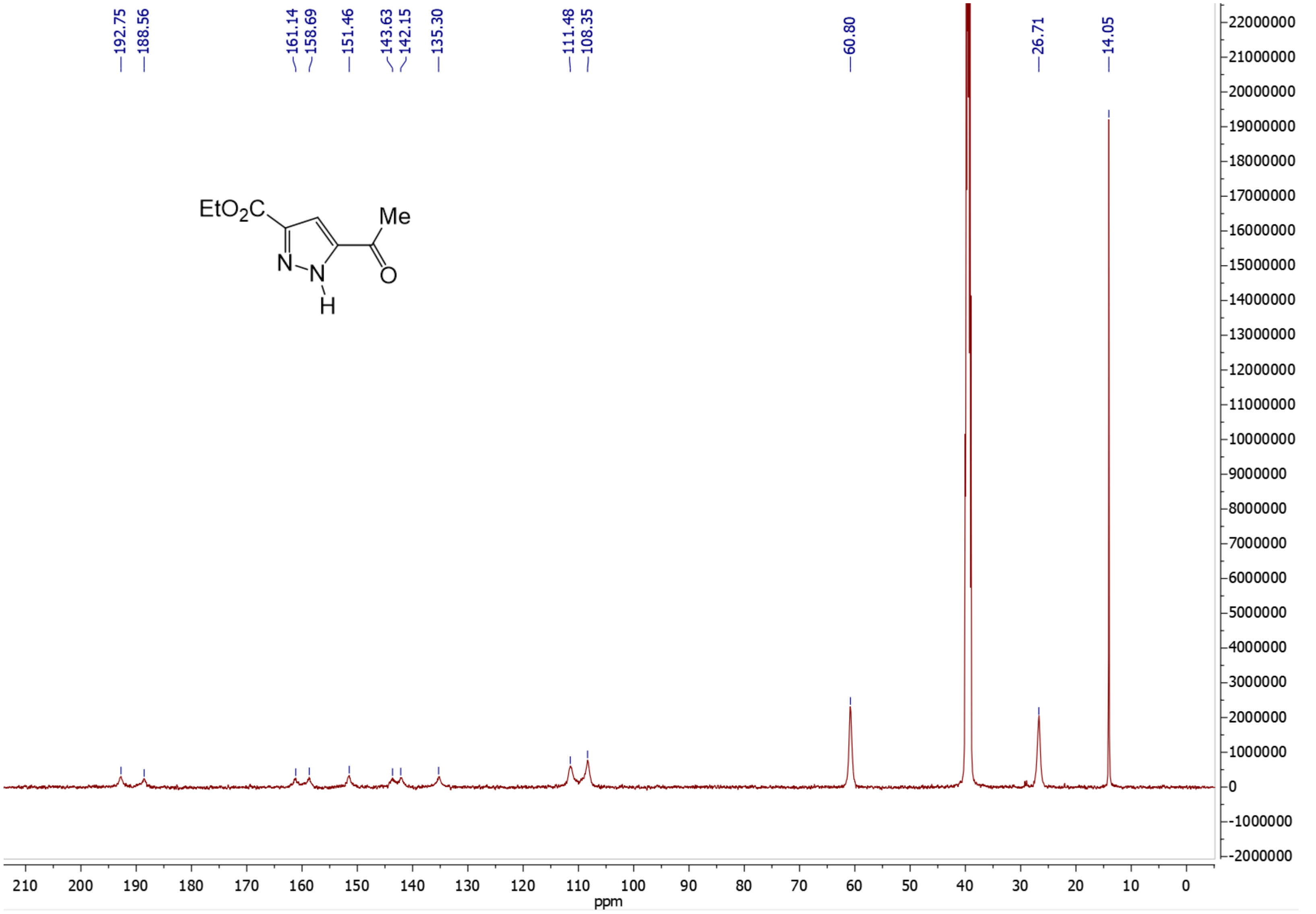

Figure S17. ^{1}H NMR spectrum of compound **7a**.

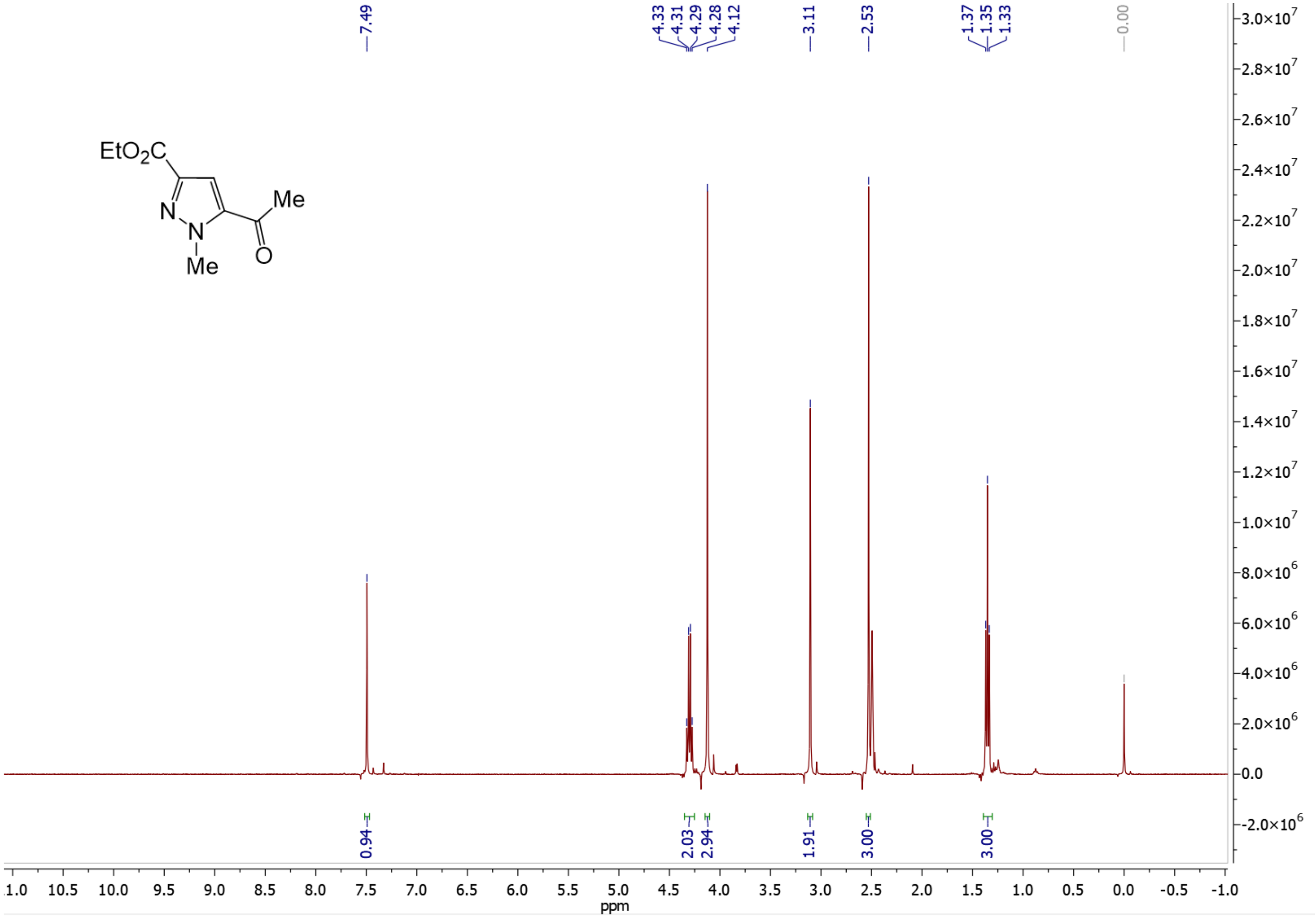

Figure S18. ^{13}C NMR spectrum of compound **7a**.

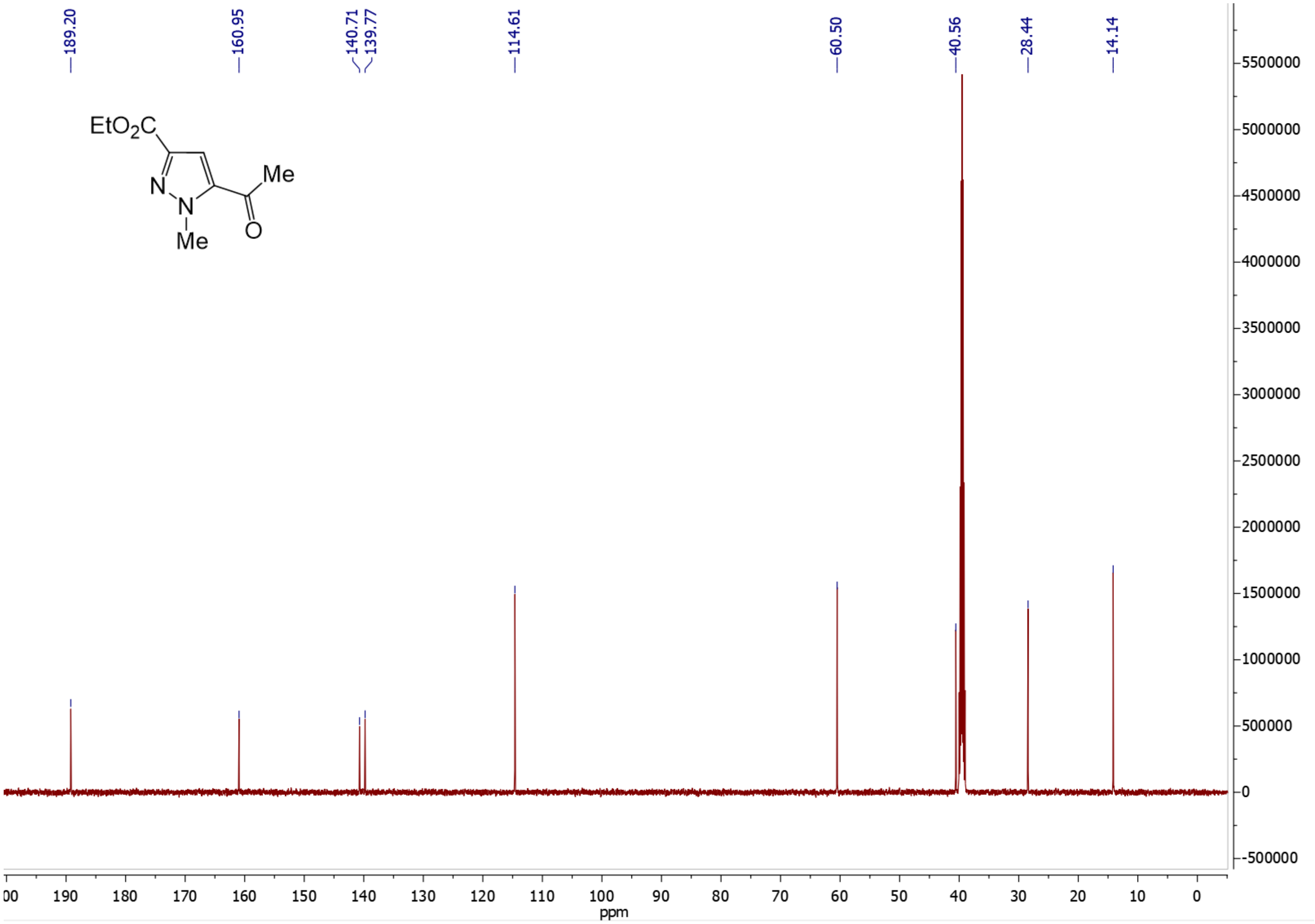

Figure S19. ^{1}H NMR spectrum of compound **7b**.

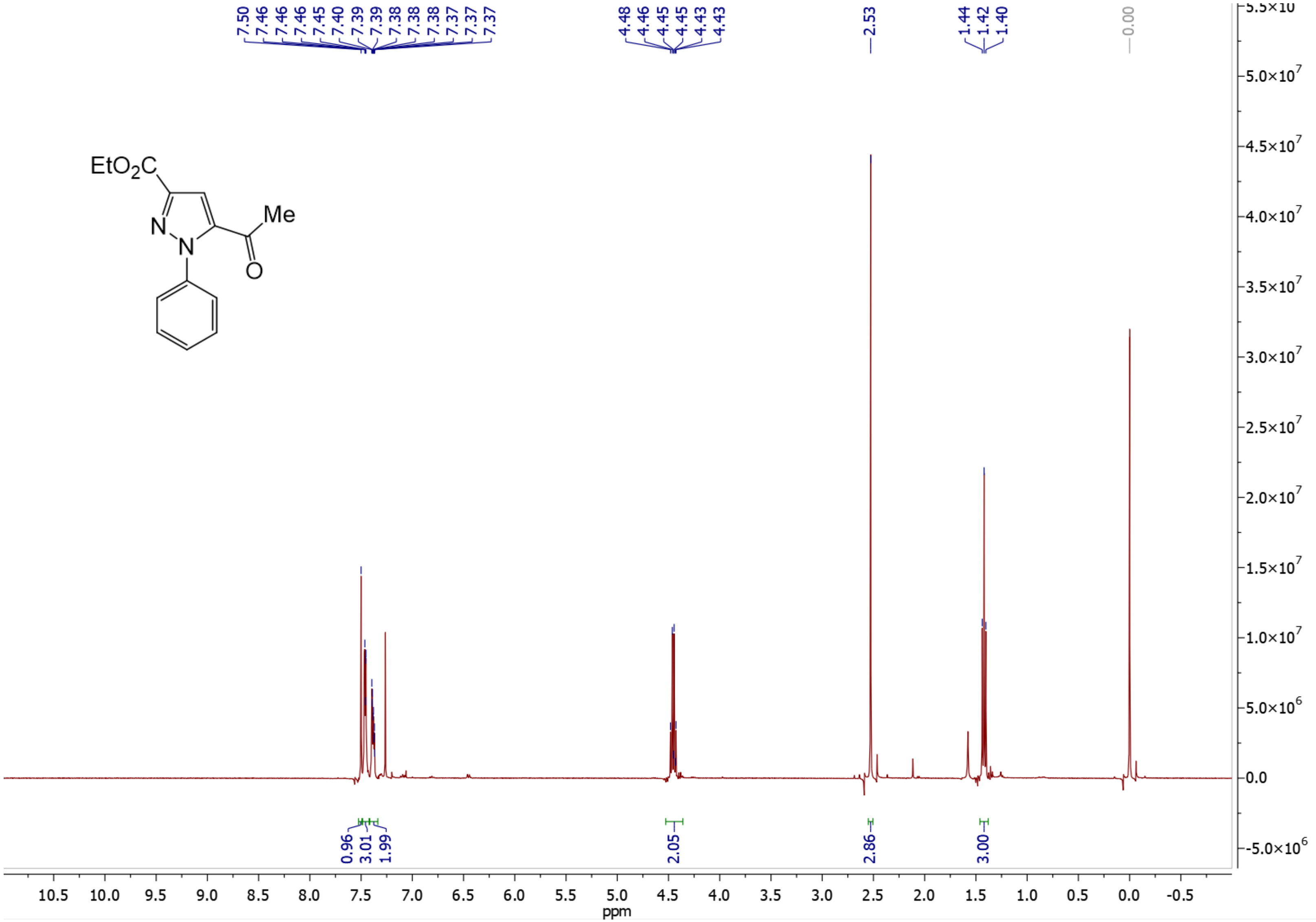

Figure S20. ^{13}C NMR spectrum of compound **7b**.

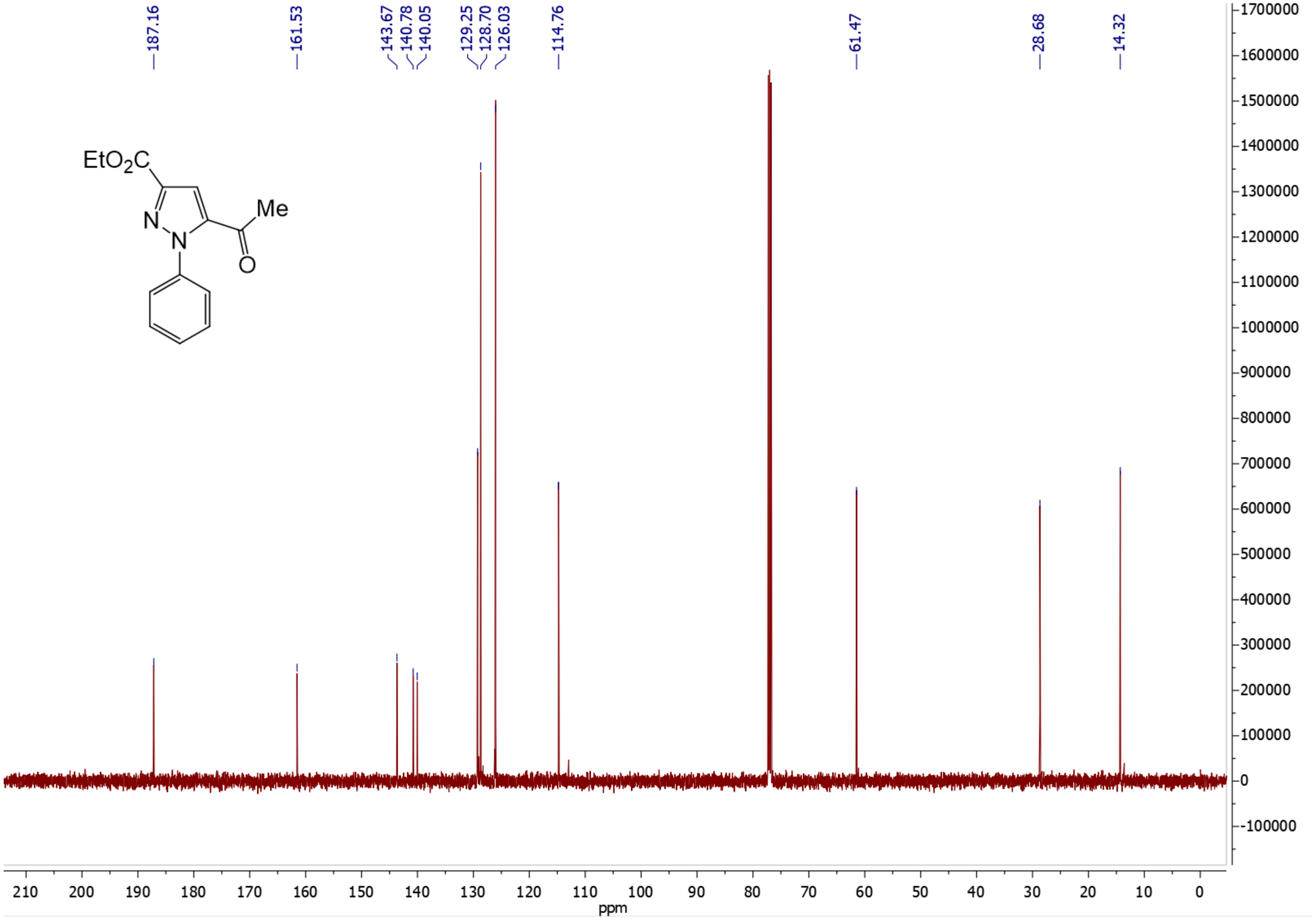

Figure S21. ¹H NMR spectrum of compound **7c**.

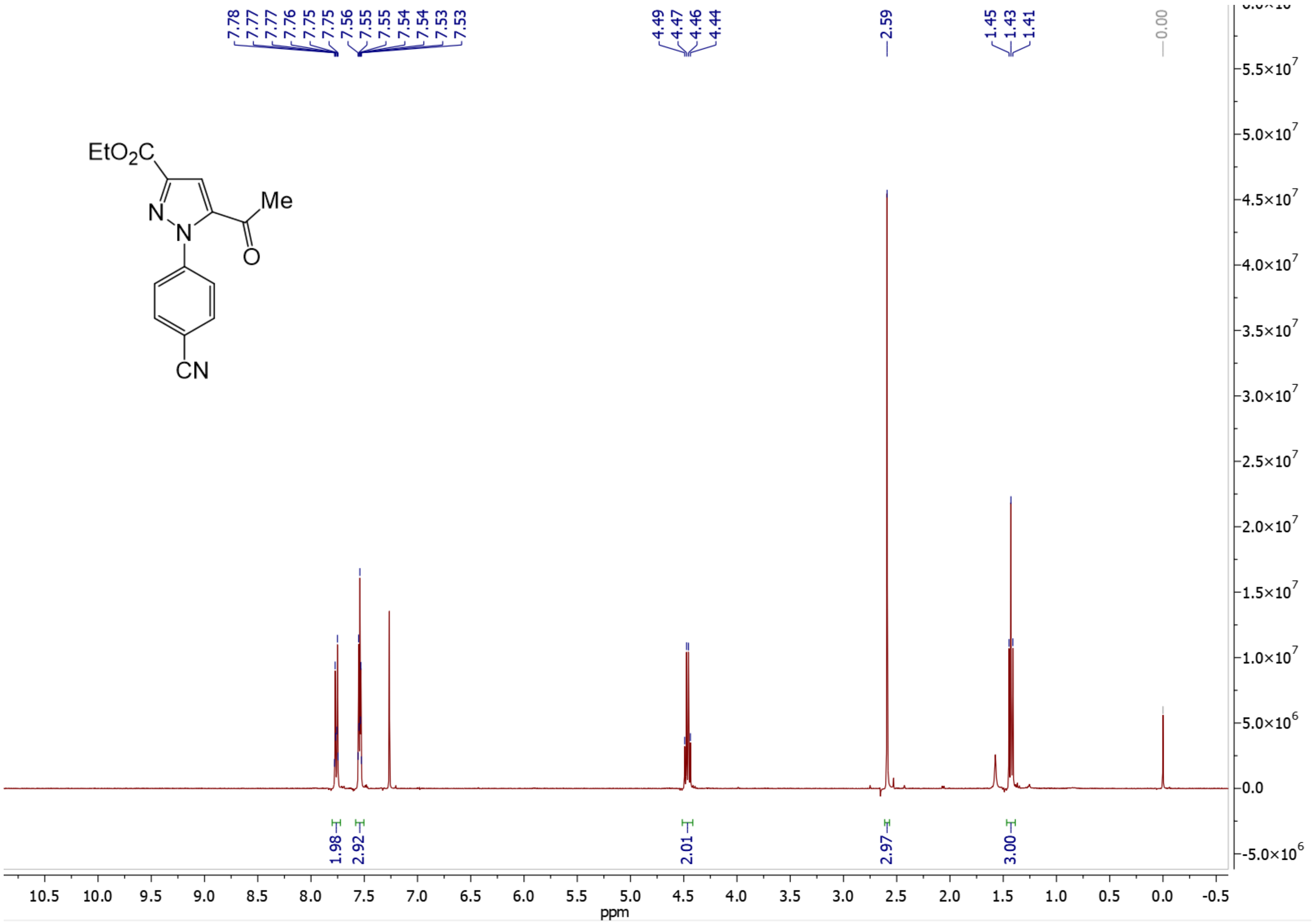

Figure S22. ^{13}C NMR spectrum of compound **7c.**

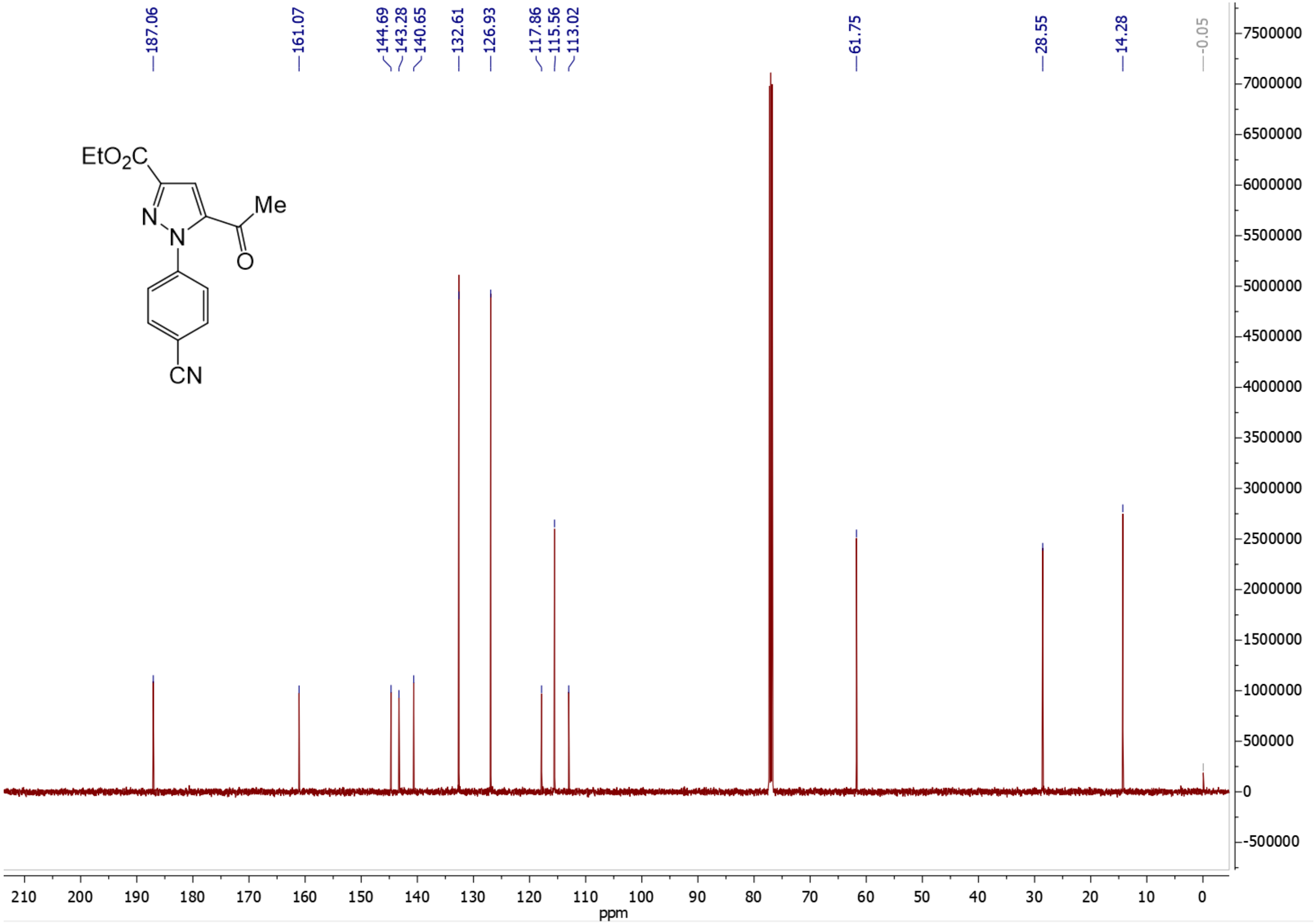

Figure S23. ^{1}H NMR spectrum of compound **7d**.

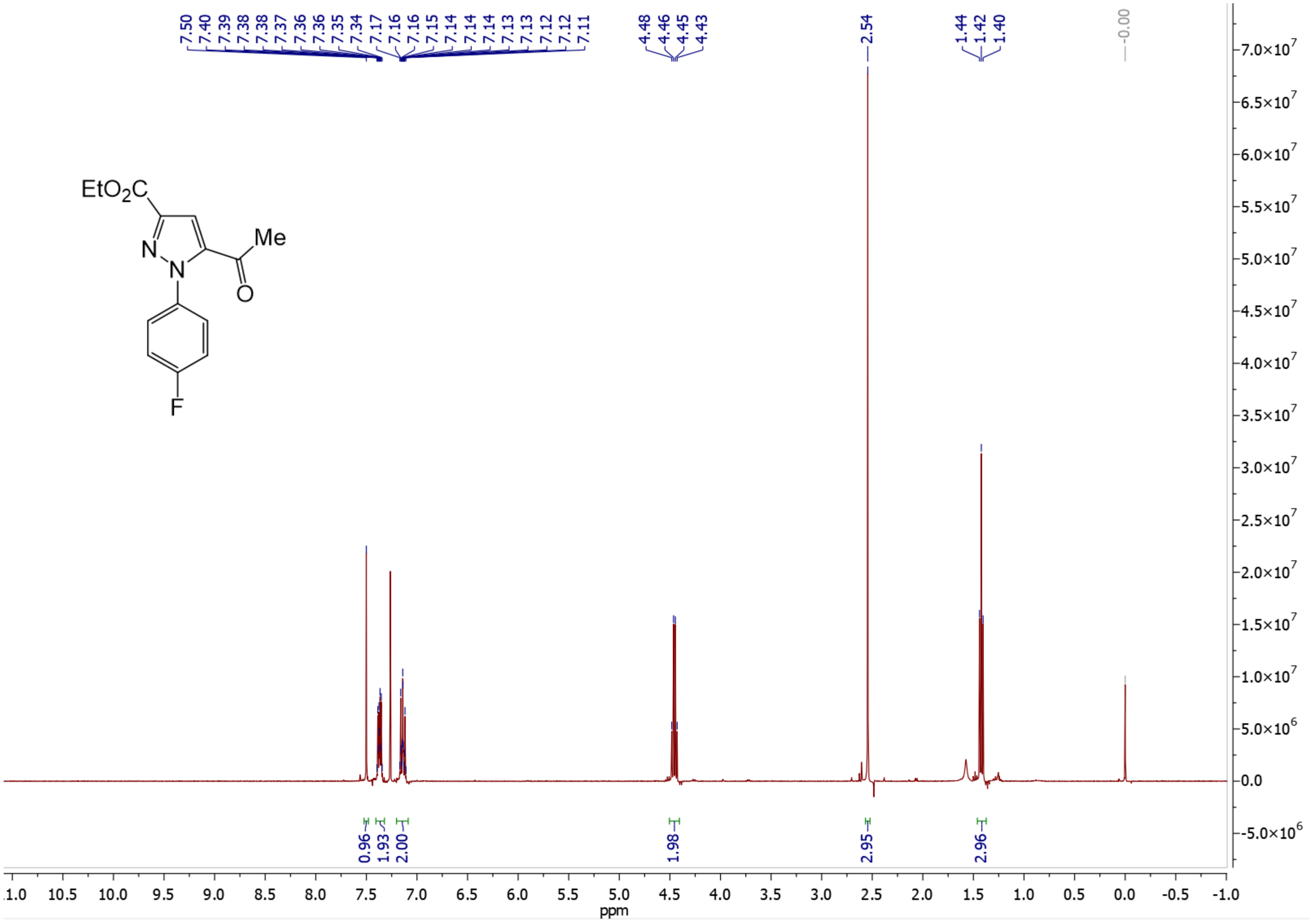

Figure S24. ^{13}C NMR spectrum of compound **7d**.

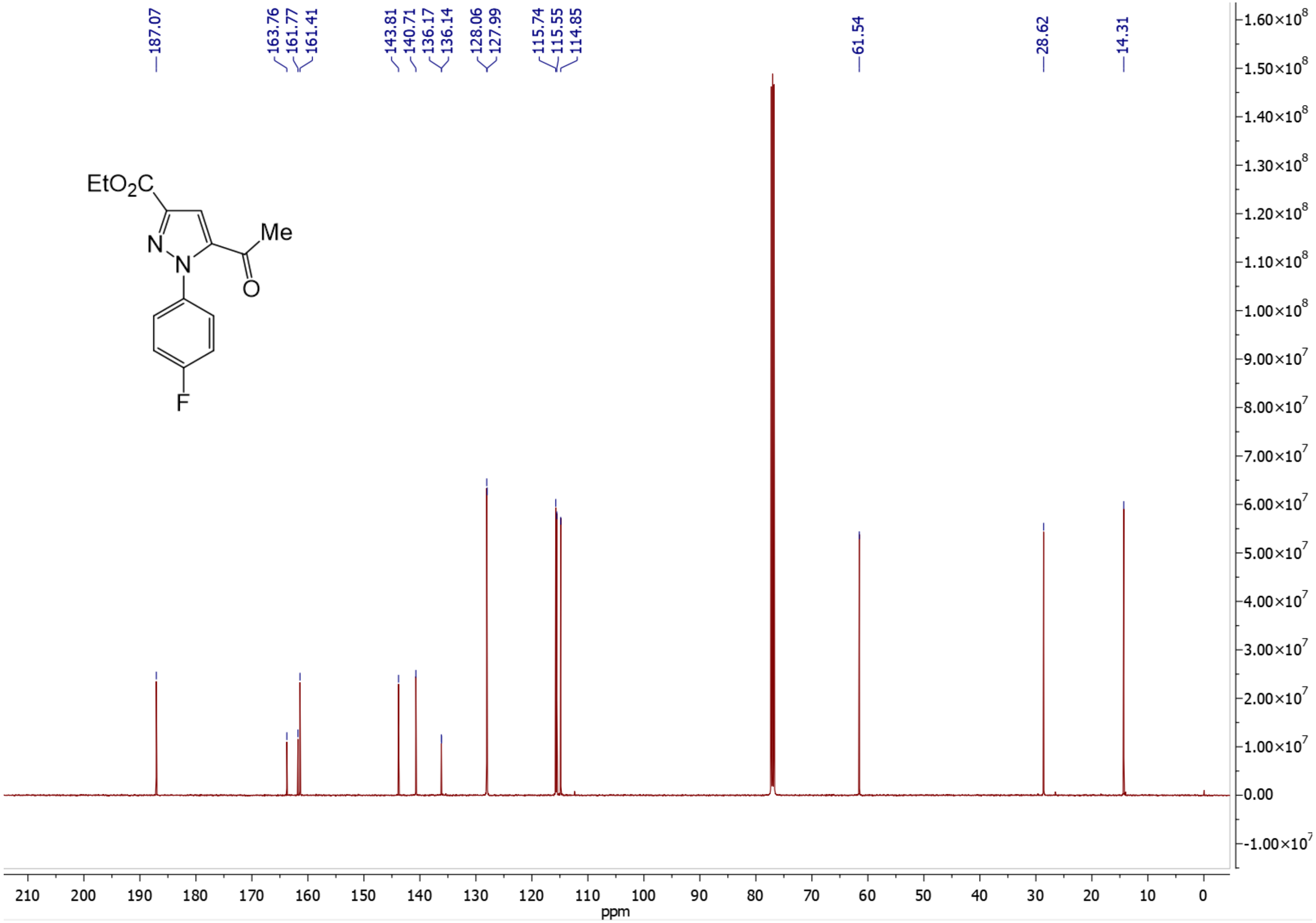

Figure S25. ^{19}F NMR spectrum of compound **7d**.

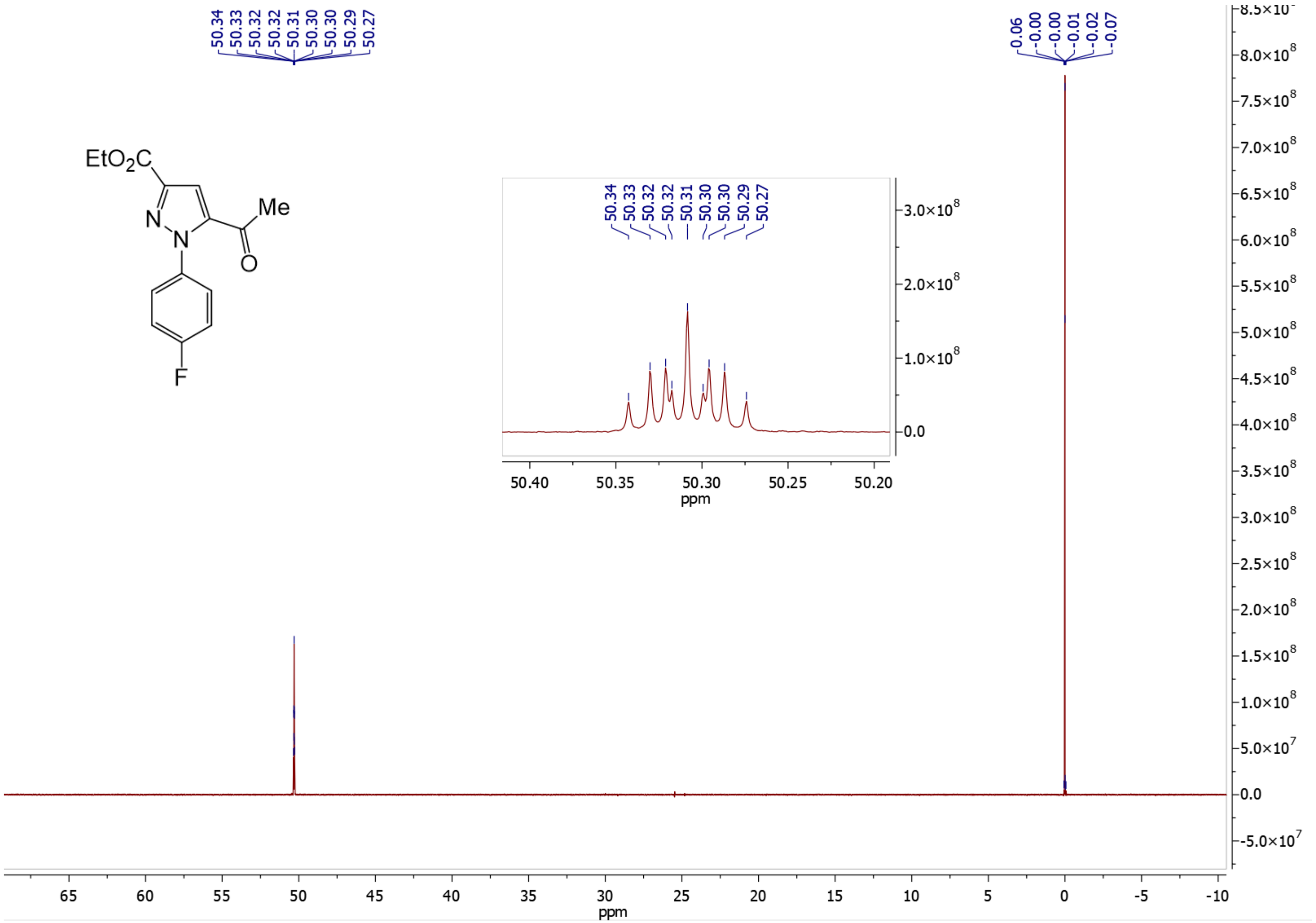

Figure S26. ^{1}H NMR spectrum of compound **7e**.

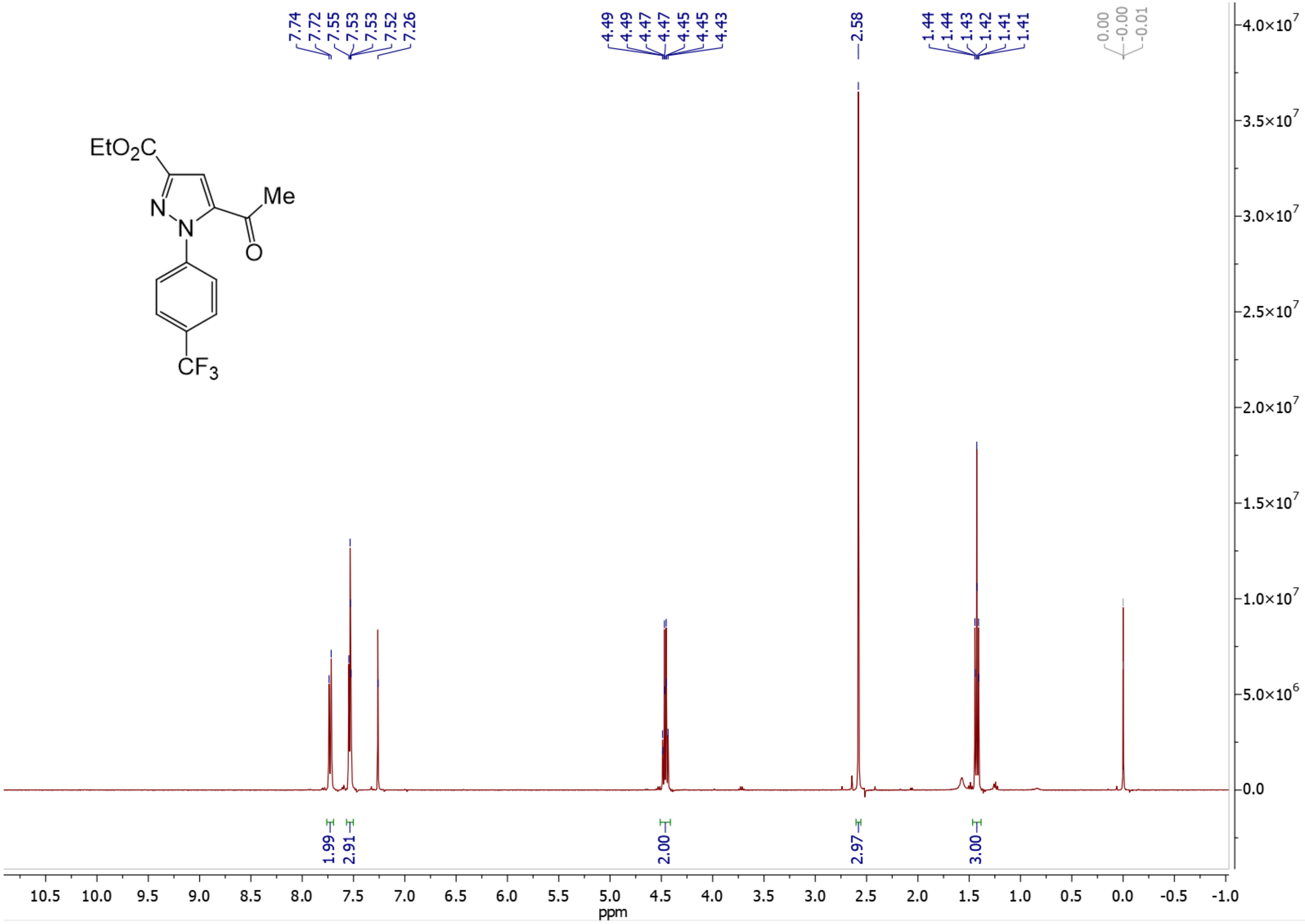

Figure S27. ^{13}C NMR spectrum of compound **7e**.

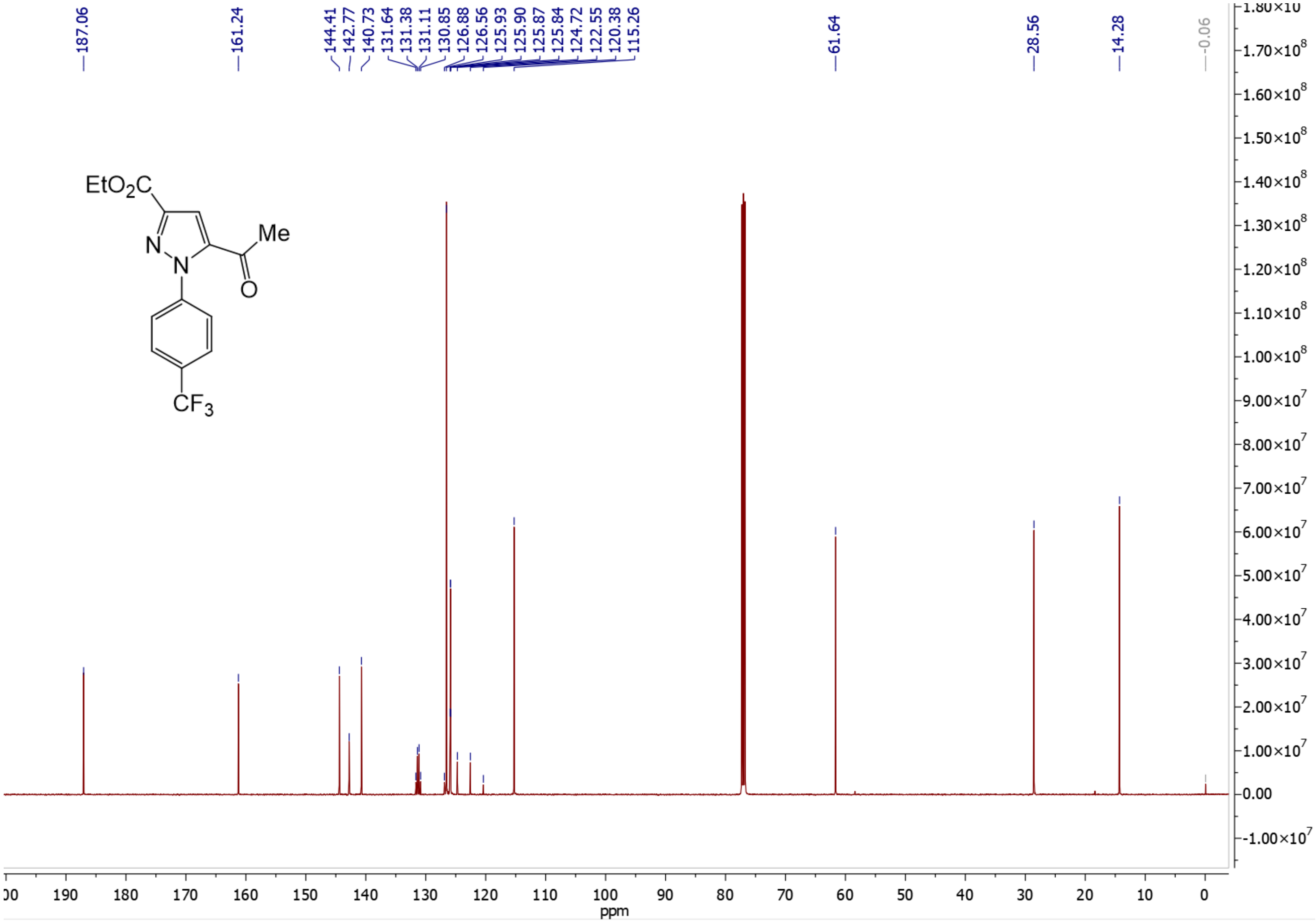

Figure S28. ^{19}F NMR spectrum of compound **7e**.

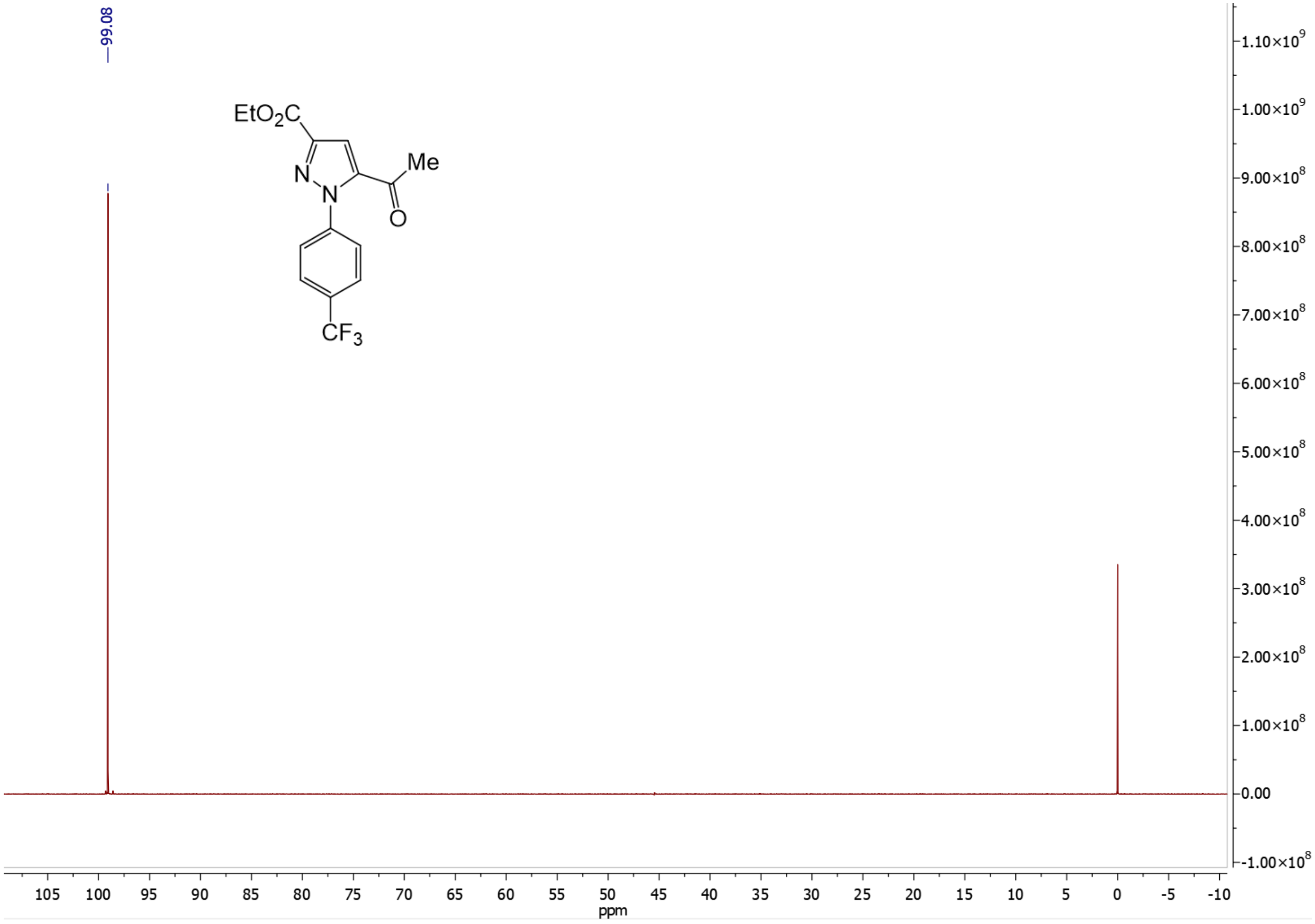

Figure S29. ^{1}H NMR spectrum of compound **7f**.

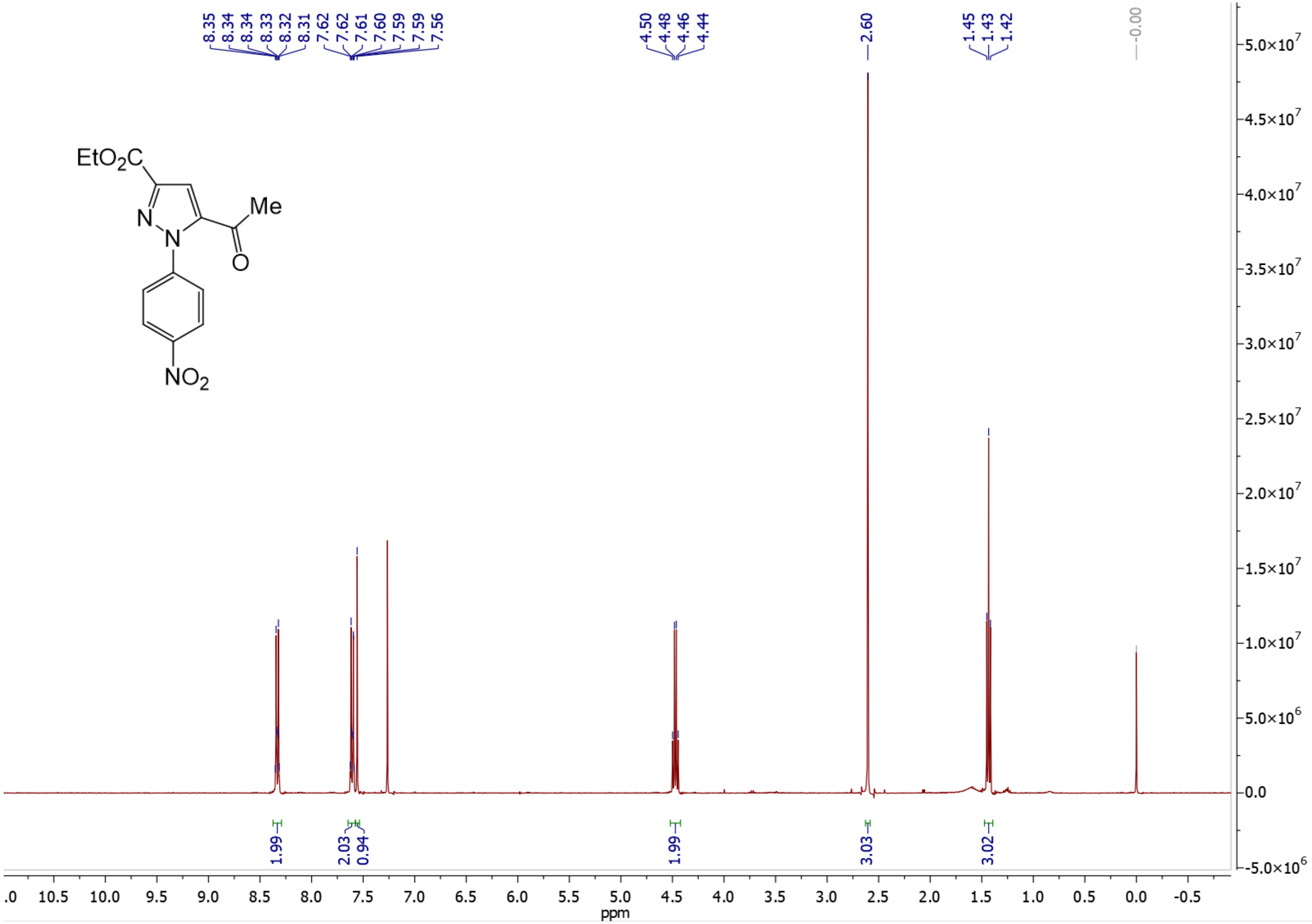

Figure S30. ^{13}C NMR spectrum of compound **7f**.

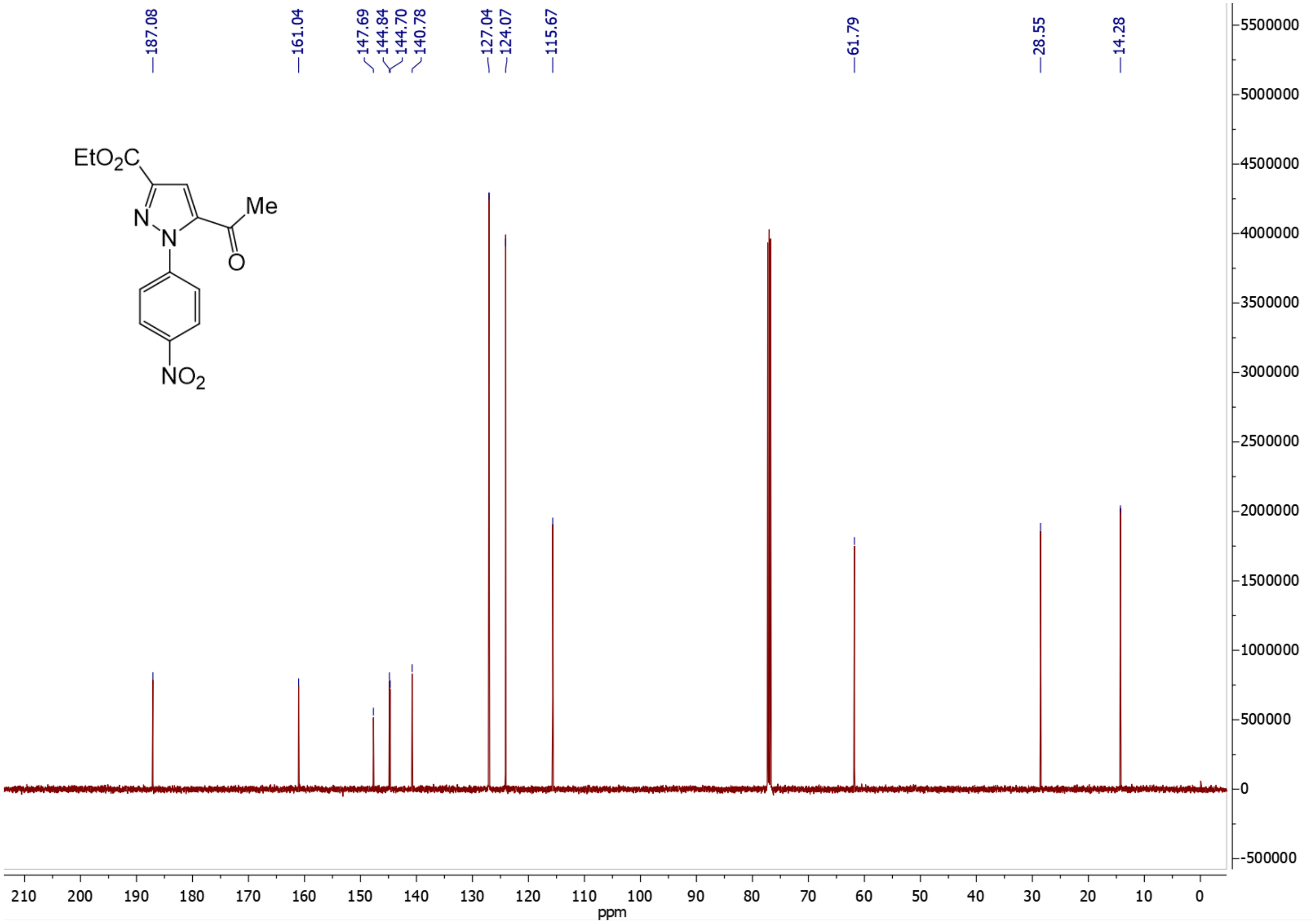

Figure S31. ^{1}H NMR spectrum of compound **7g**.

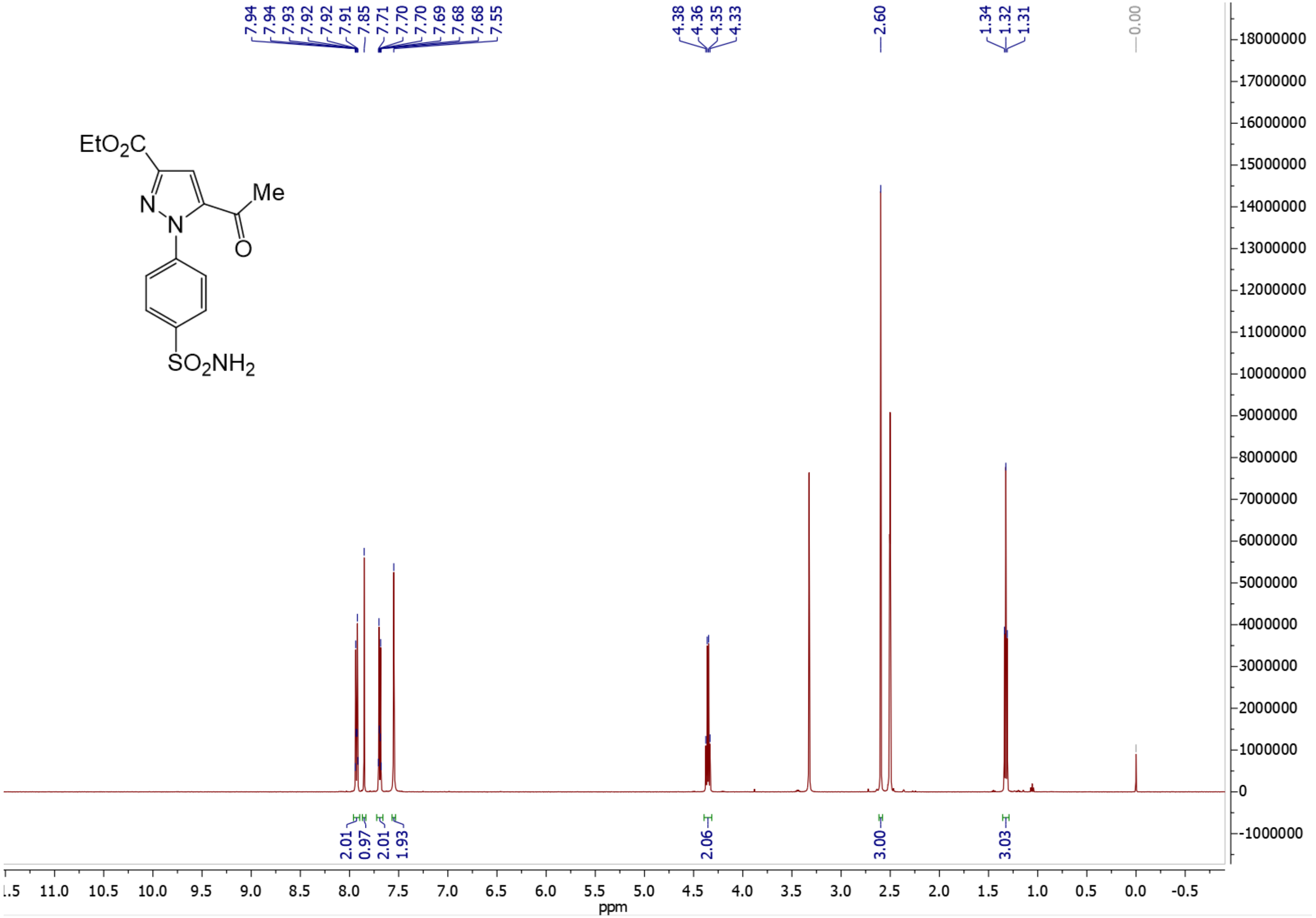

Figure S32. ^{13}C NMR spectrum of compound **7g**.

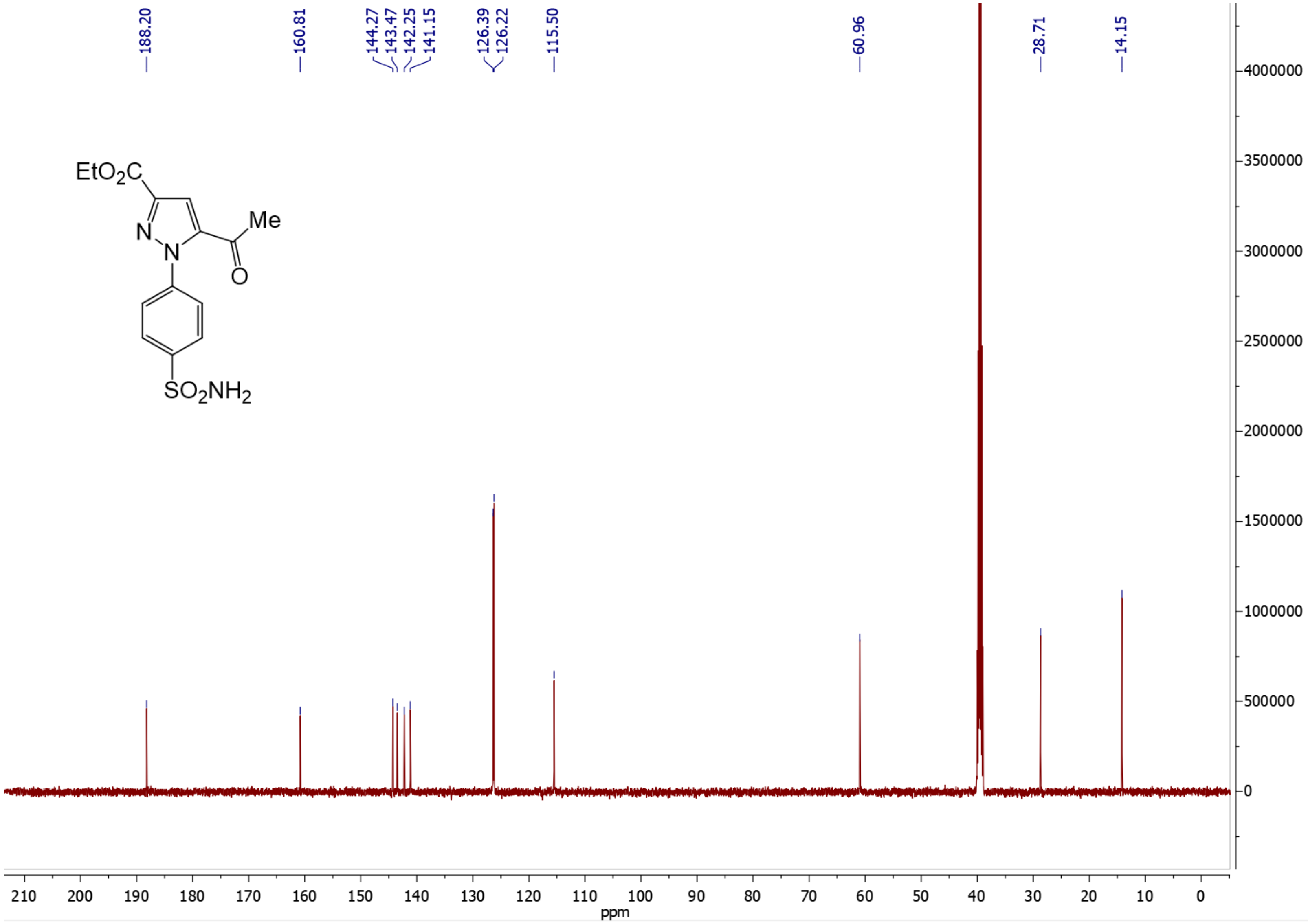

Figure S33. ^{1}H NMR spectrum of compound **7h**.

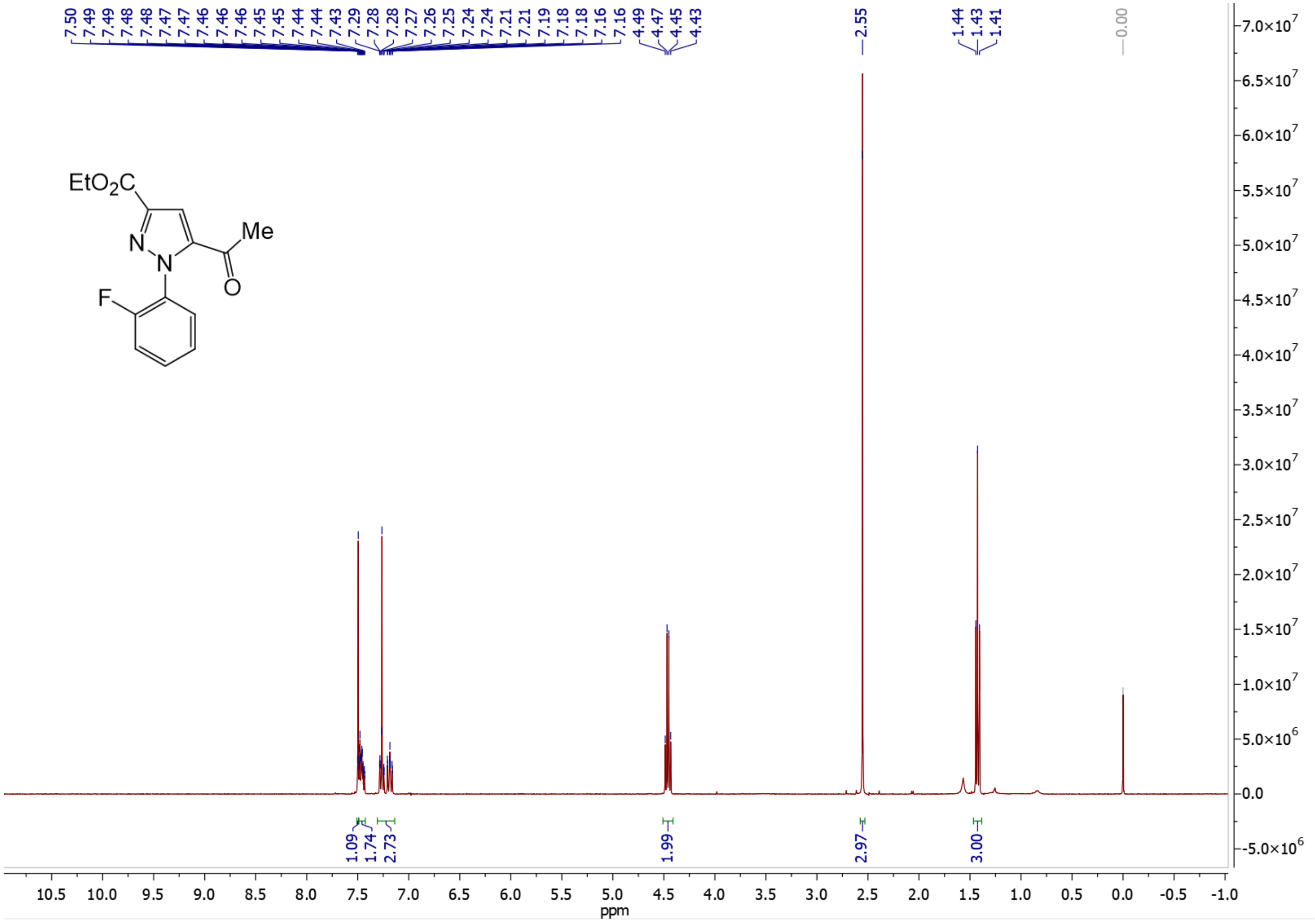

Figure S34. ^{13}C NMR spectrum of compound **7h**.

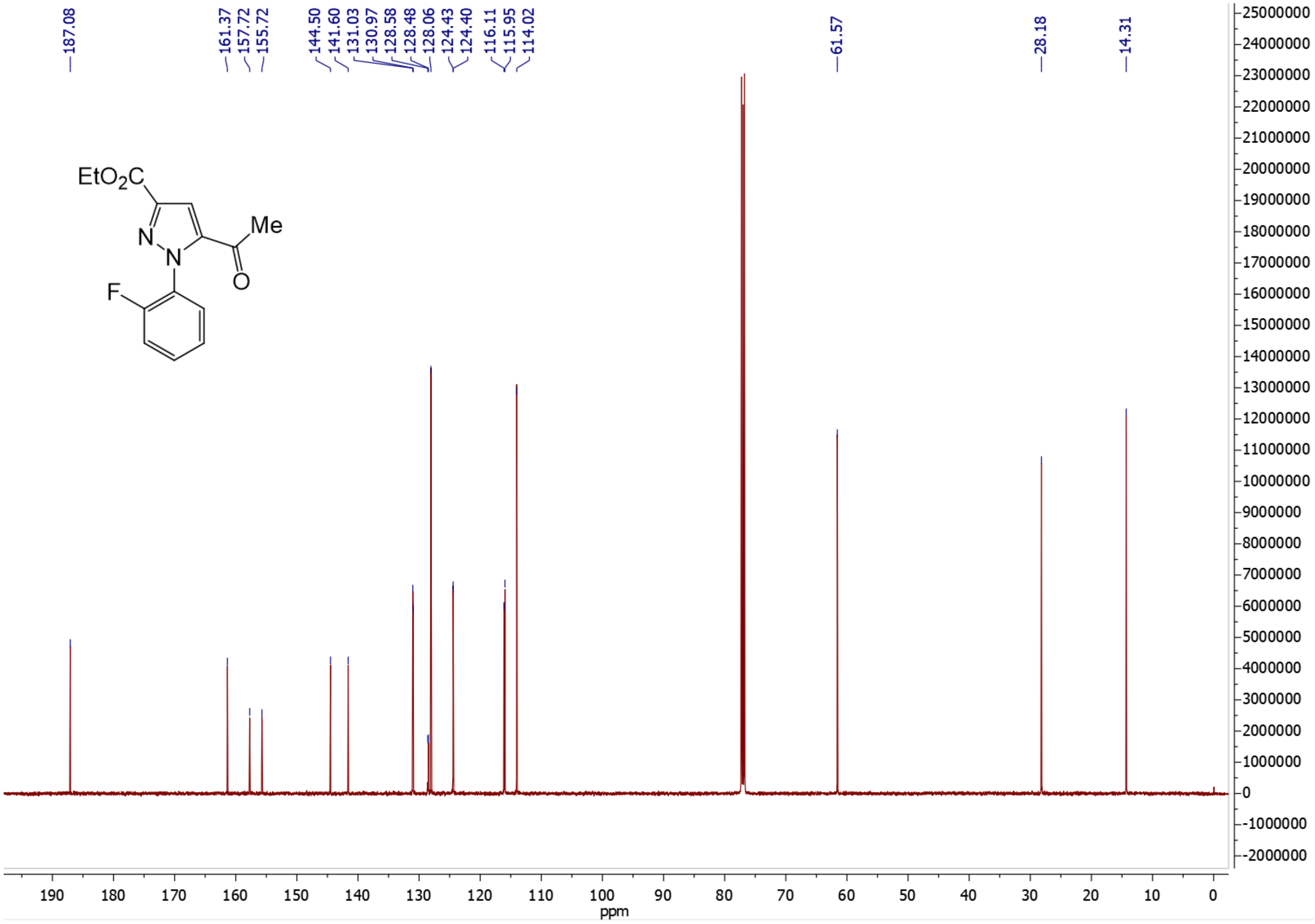

Figure S35. ^{19}F NMR spectrum of compound **7h**.

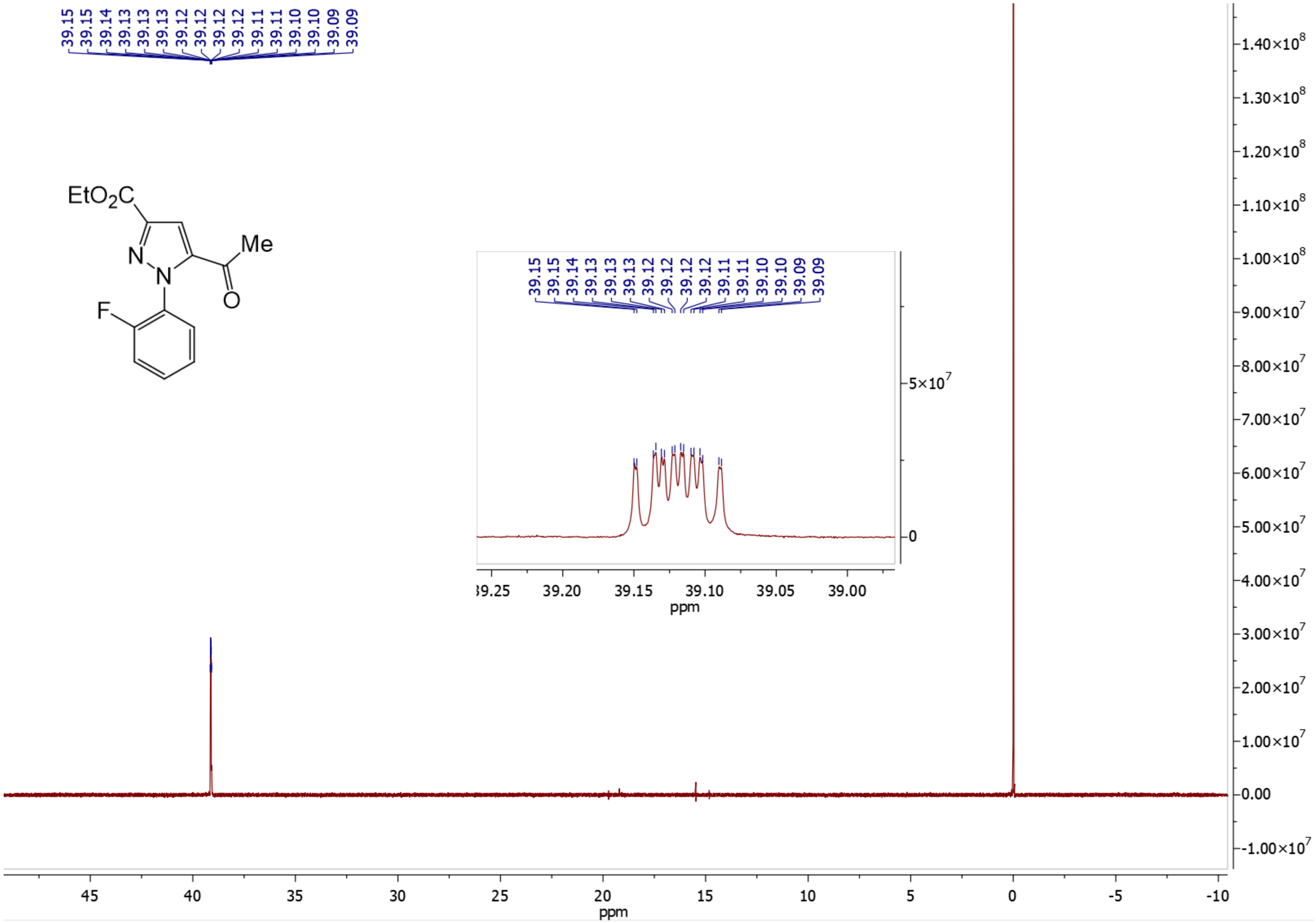

Figure S36. ^{1}H NMR spectrum of compound **7i**.

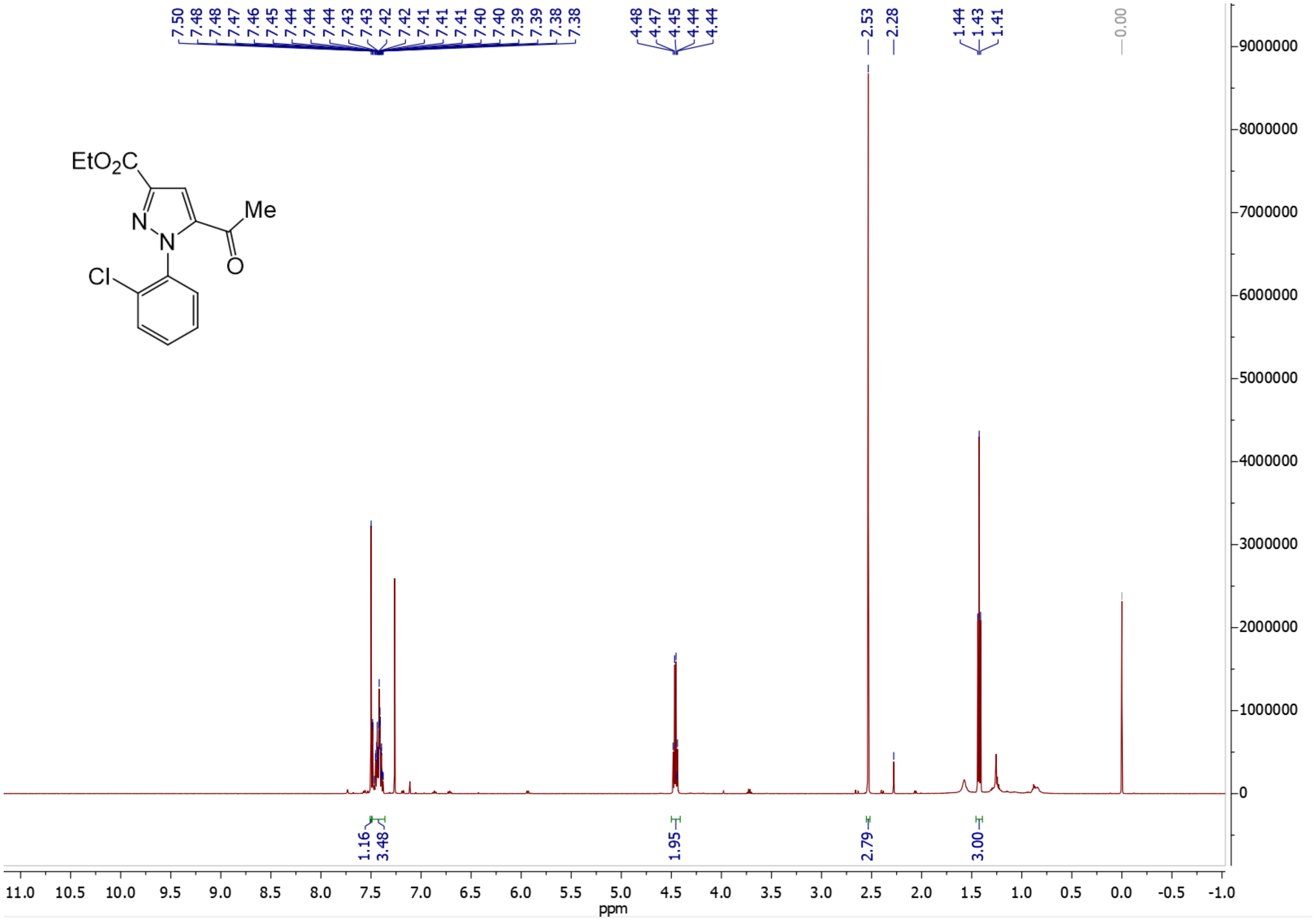

Figure S37. ^{13}C NMR spectrum of compound **7i**.

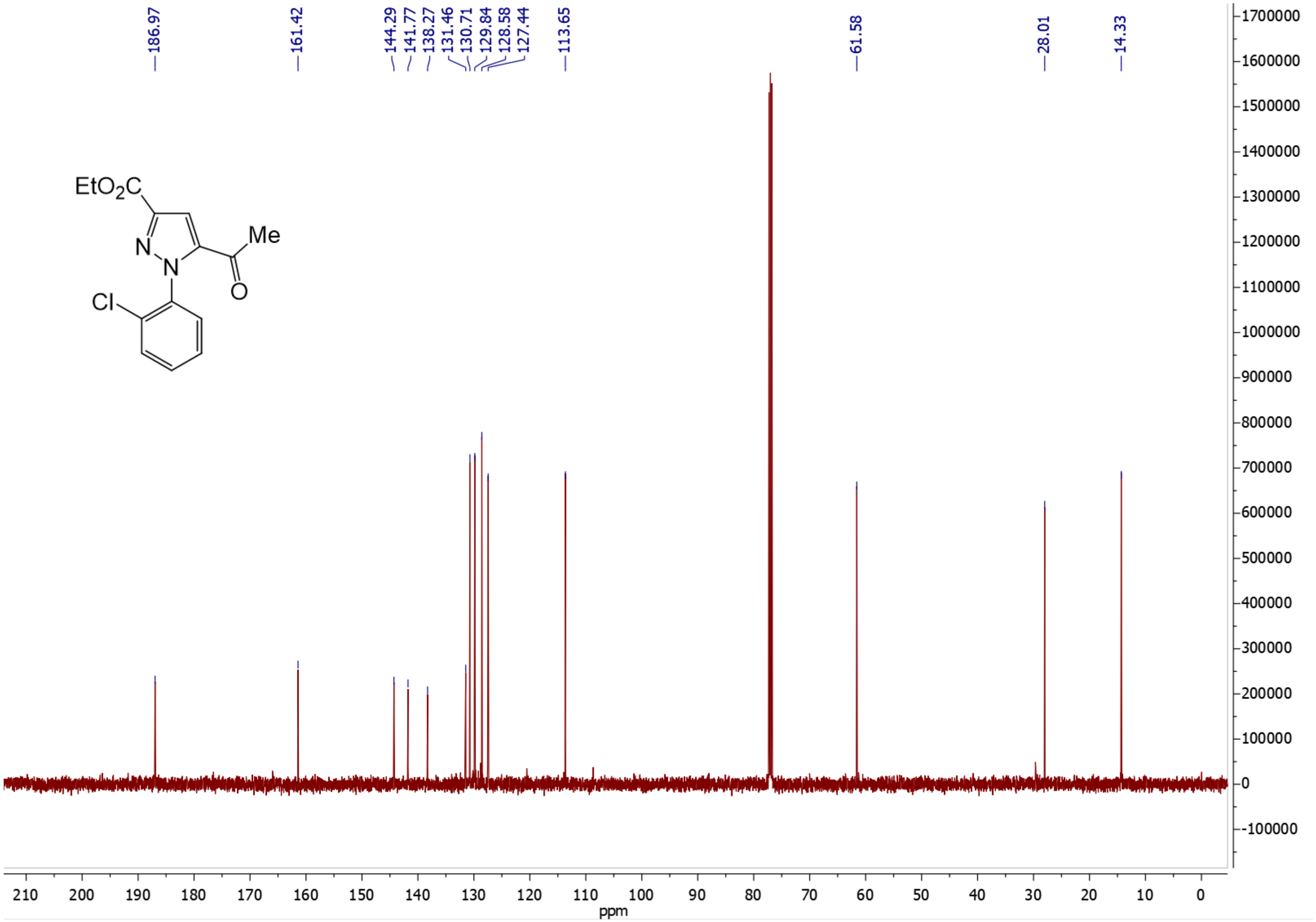

Figure S38. ^{1}H NMR spectrum of compound **7j**.

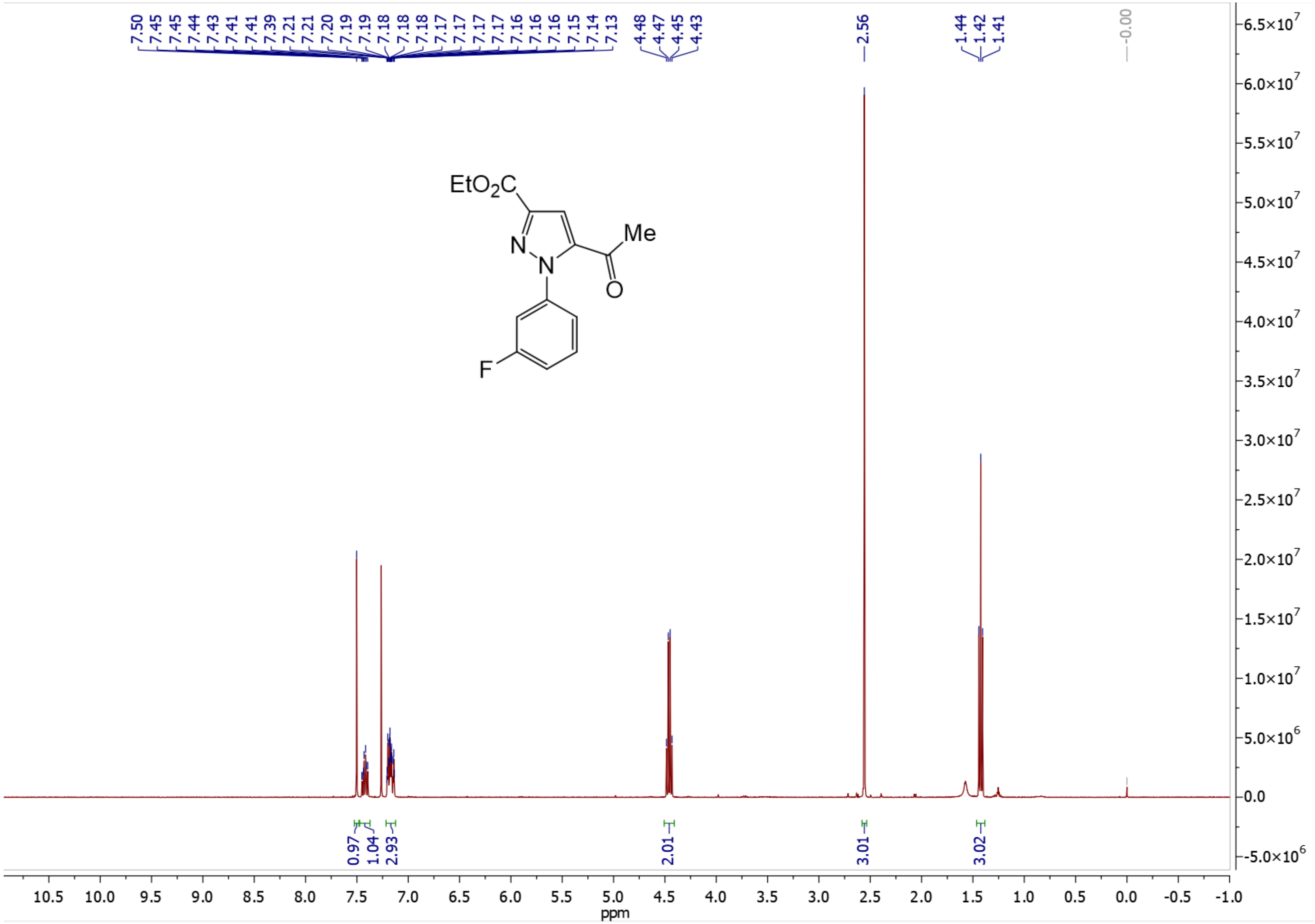

Figure S39. ^{13}C NMR spectrum of compound **7j**.

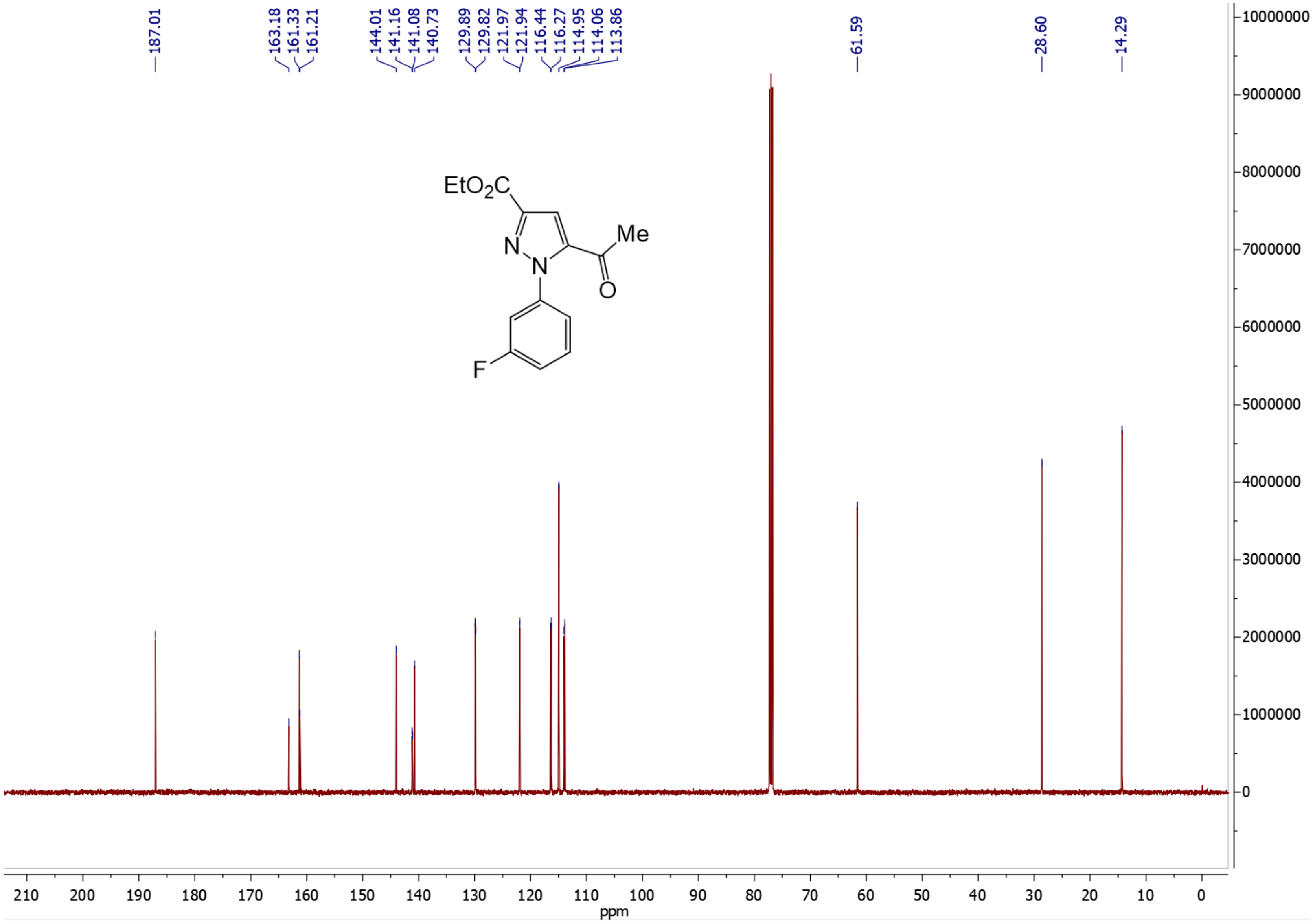

Figure S40. ^{19}F NMR spectrum of compound **7j.**

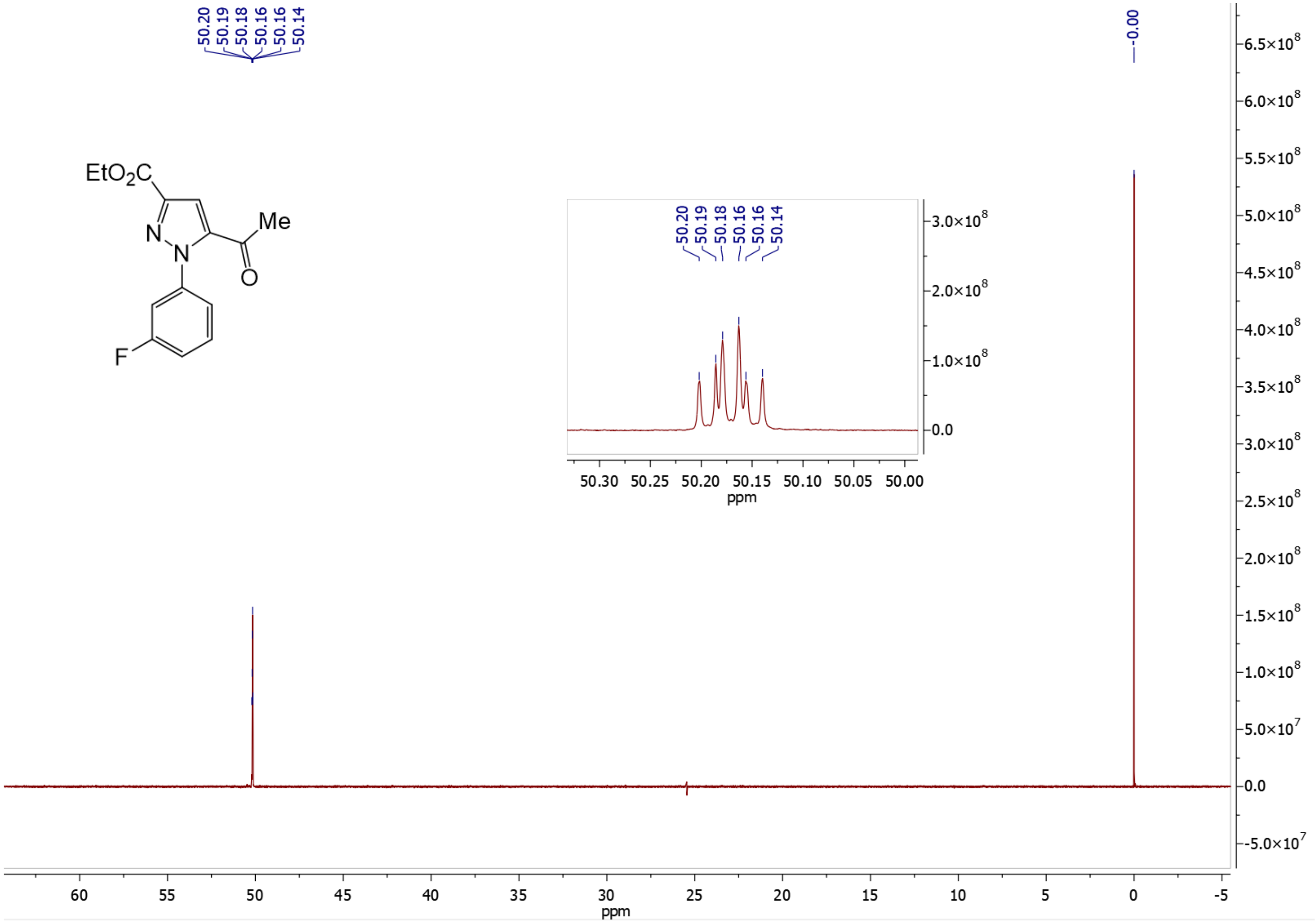

Figure S41. ^{1}H NMR spectrum of compound **7k**.

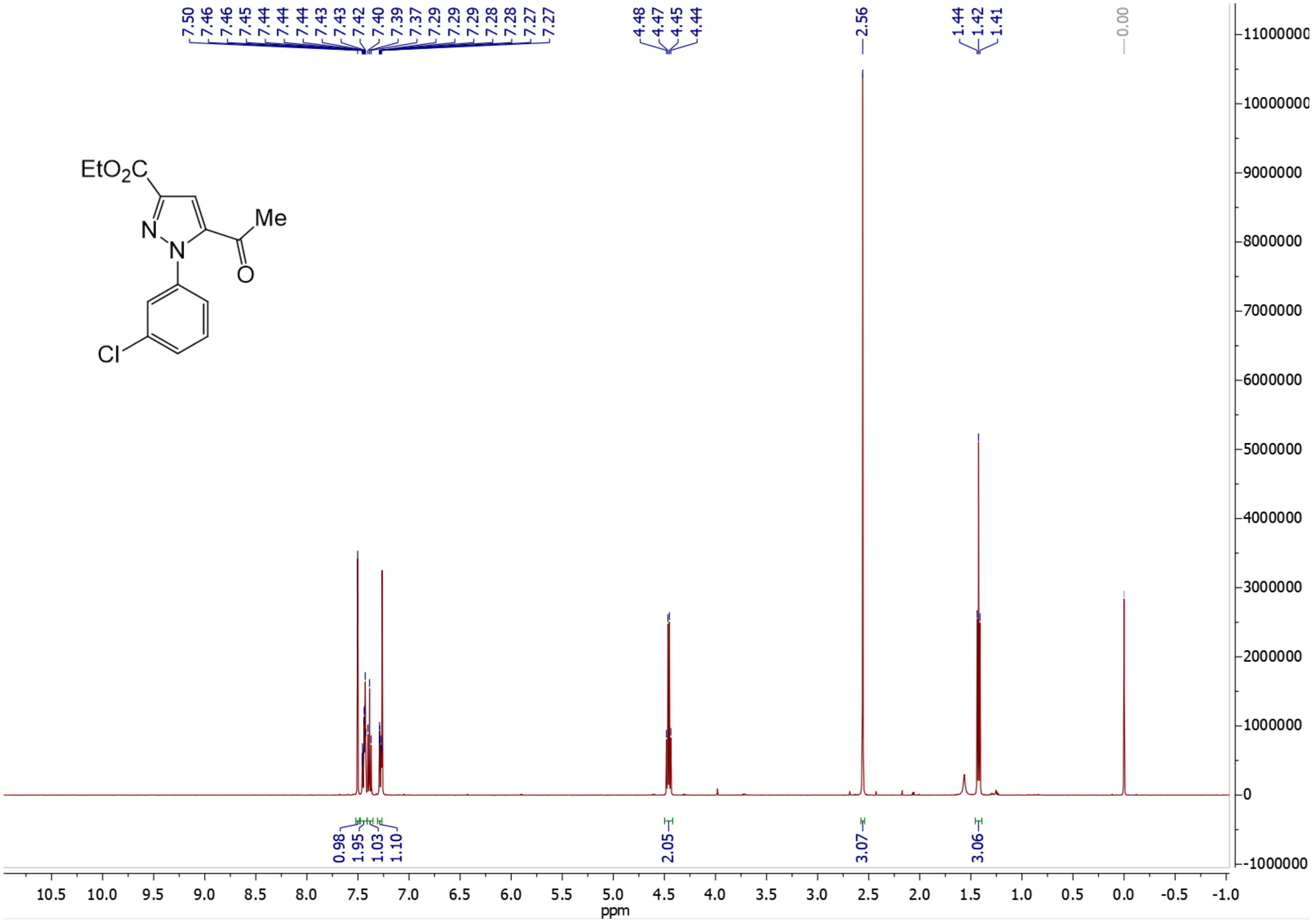

Figure S42. ^{13}C NMR spectrum of compound **7k**.

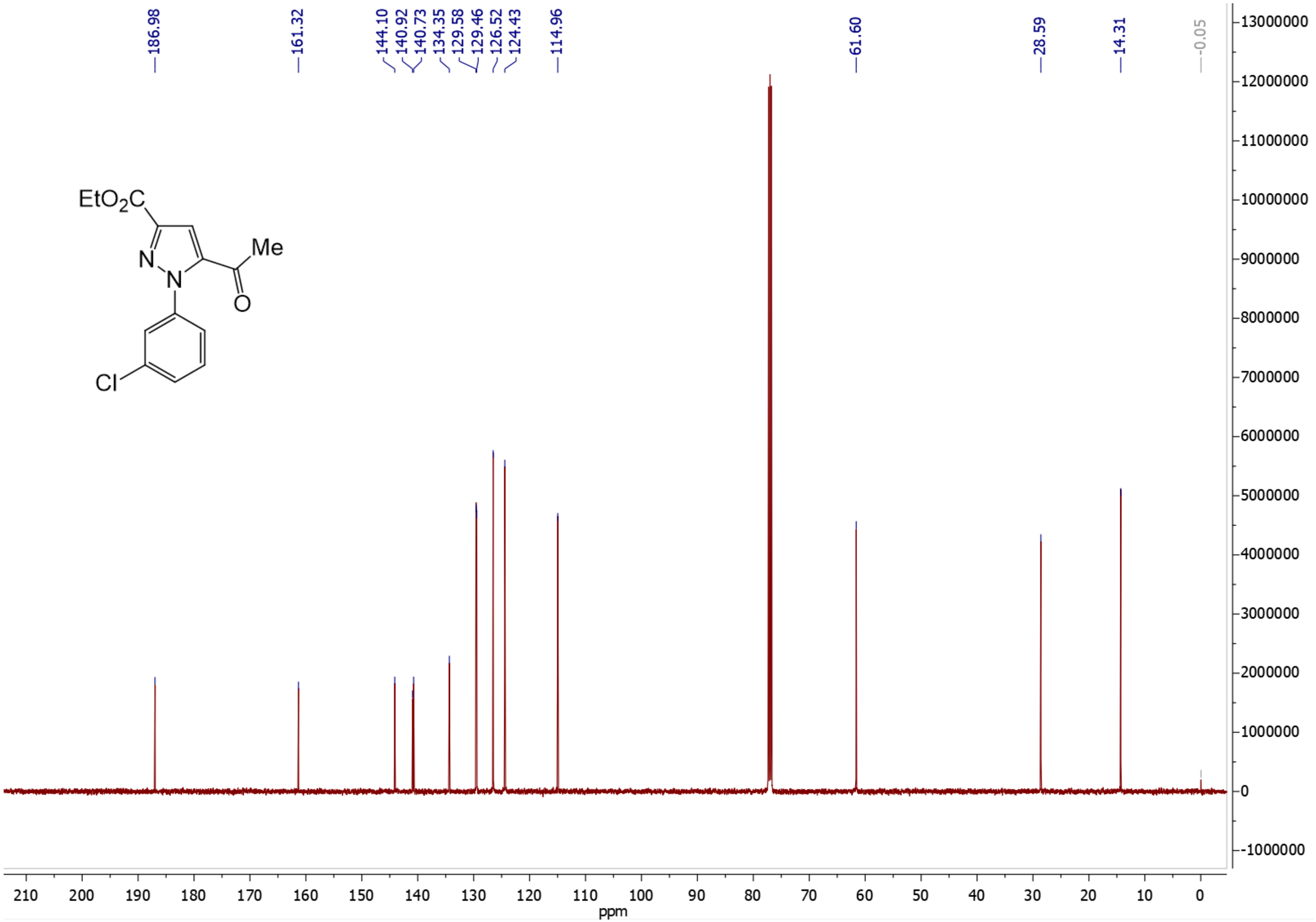

Figure S43. ^{1}H NMR spectrum of compound **7l**.

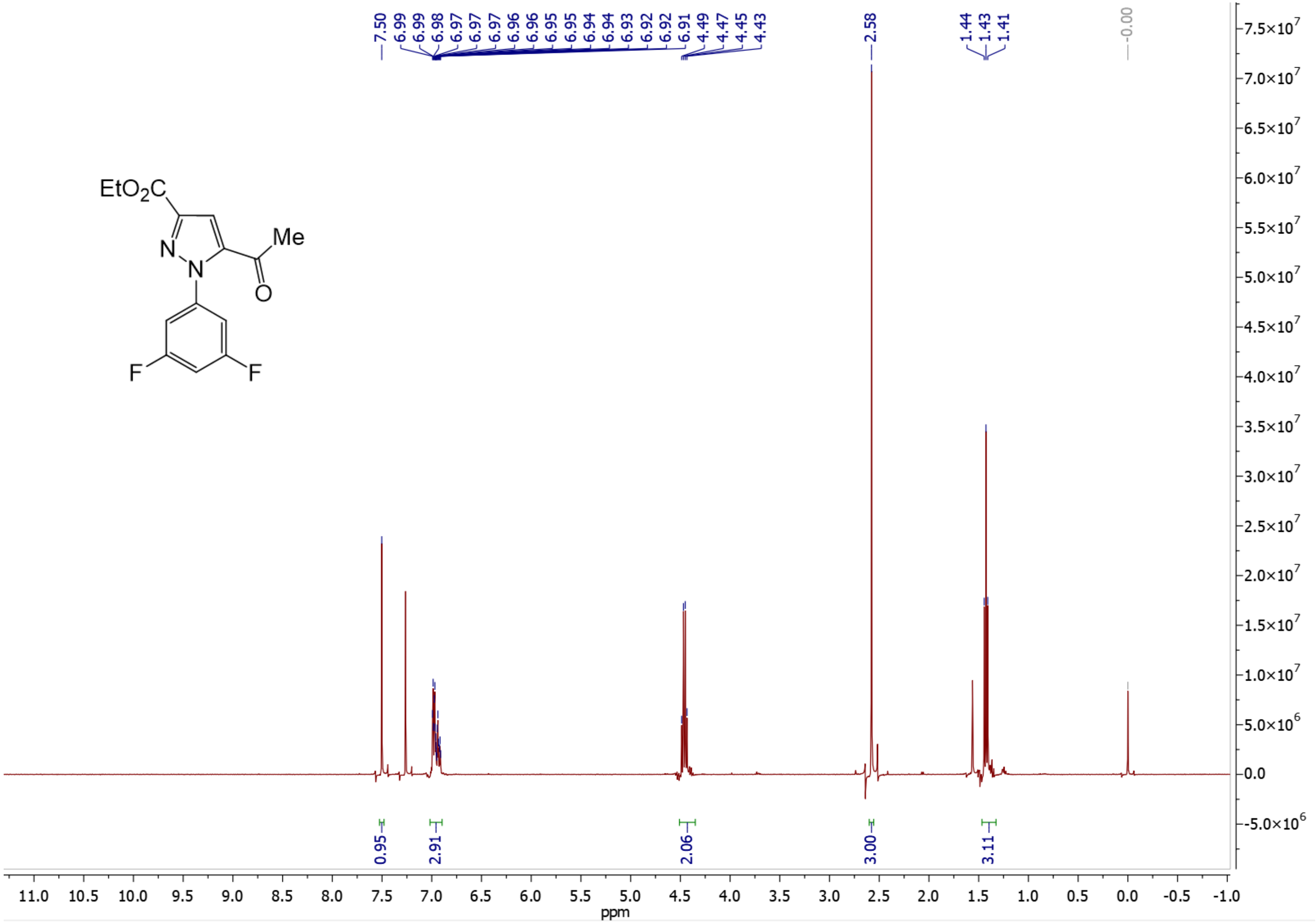

Figure S44. ^{13}C NMR spectrum of compound **7l**.

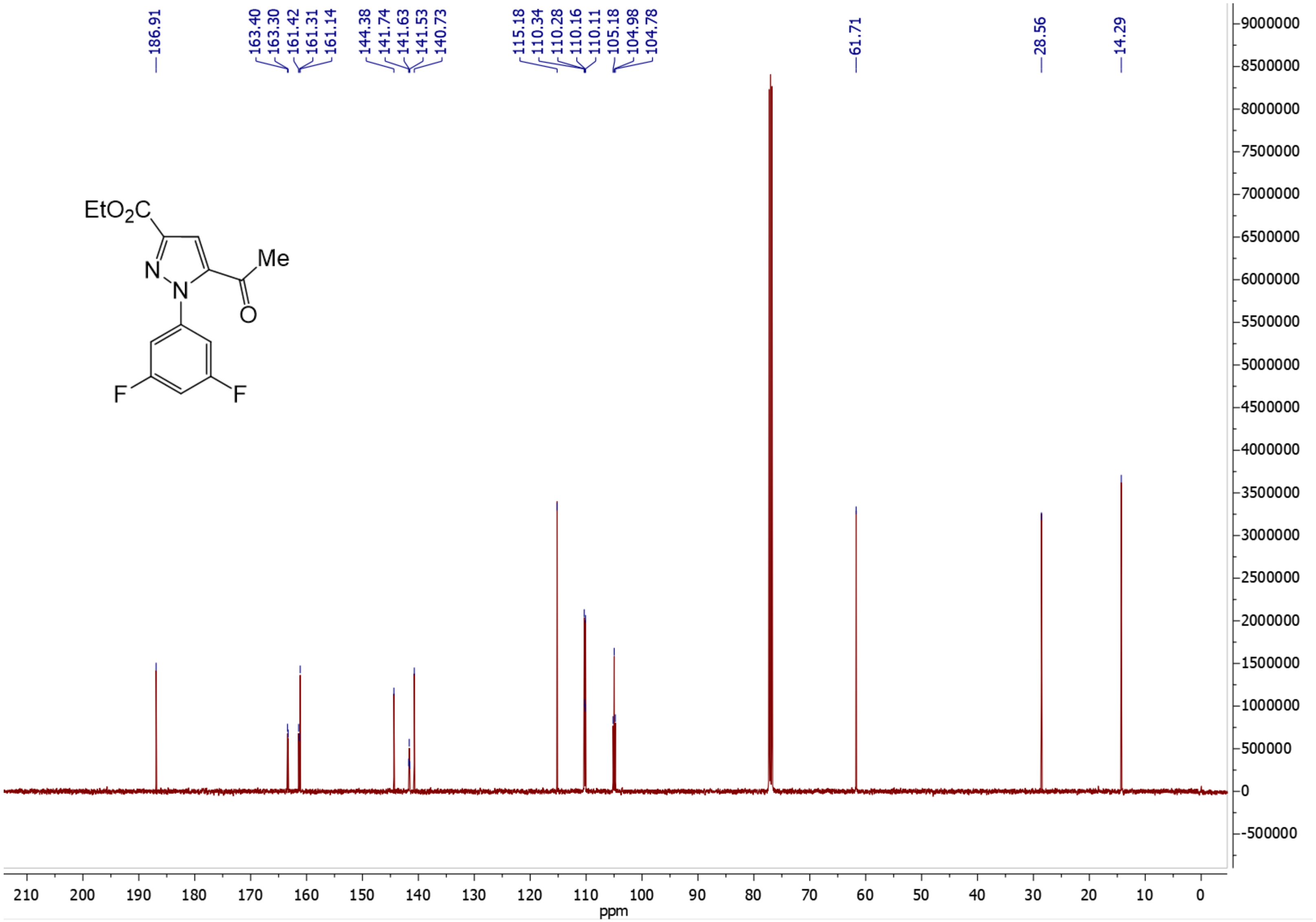

Figure S45. ^{19}F NMR spectrum of compound **7l**.

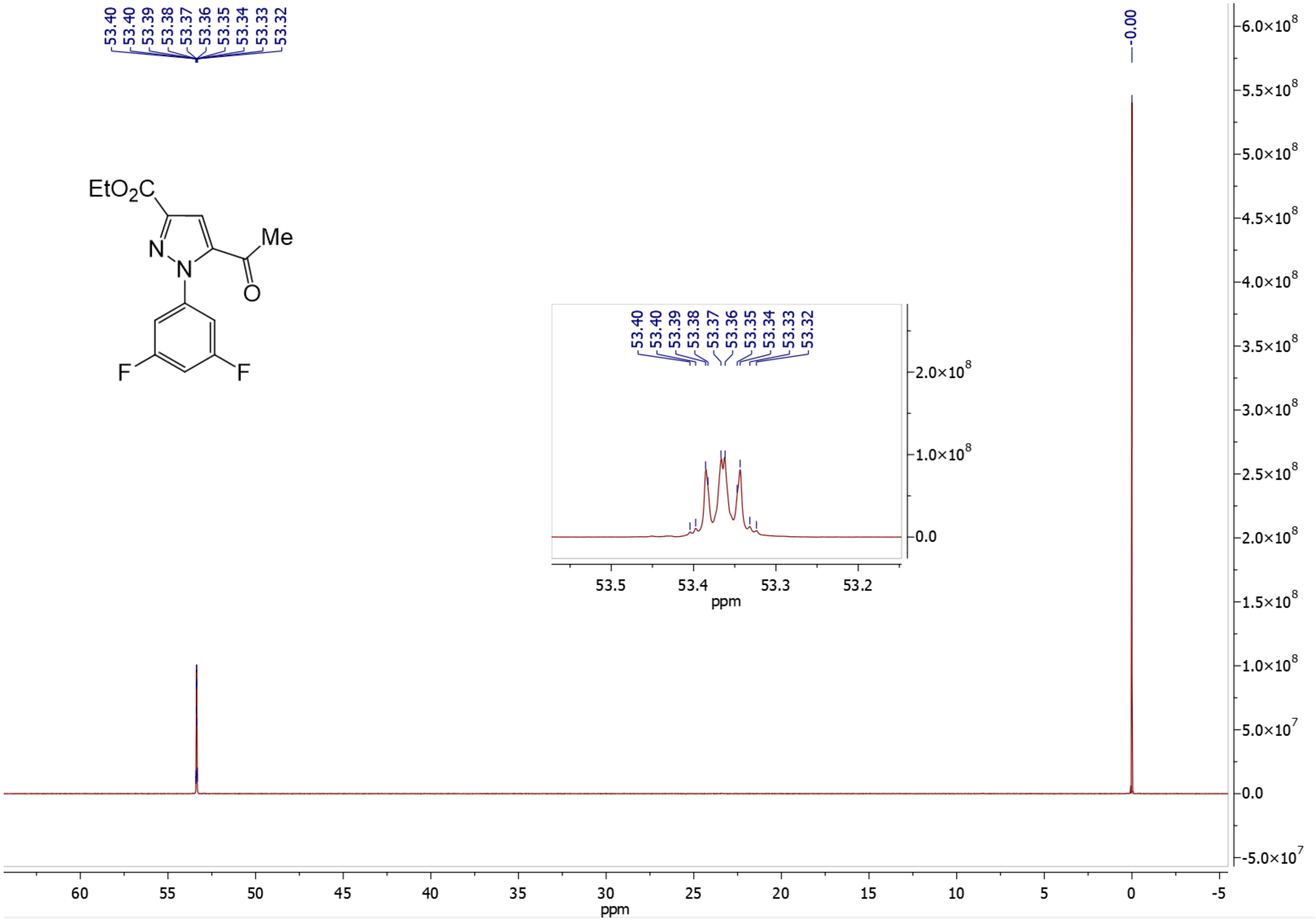

Figure S46. ^{1}H NMR spectrum of compound **8**.

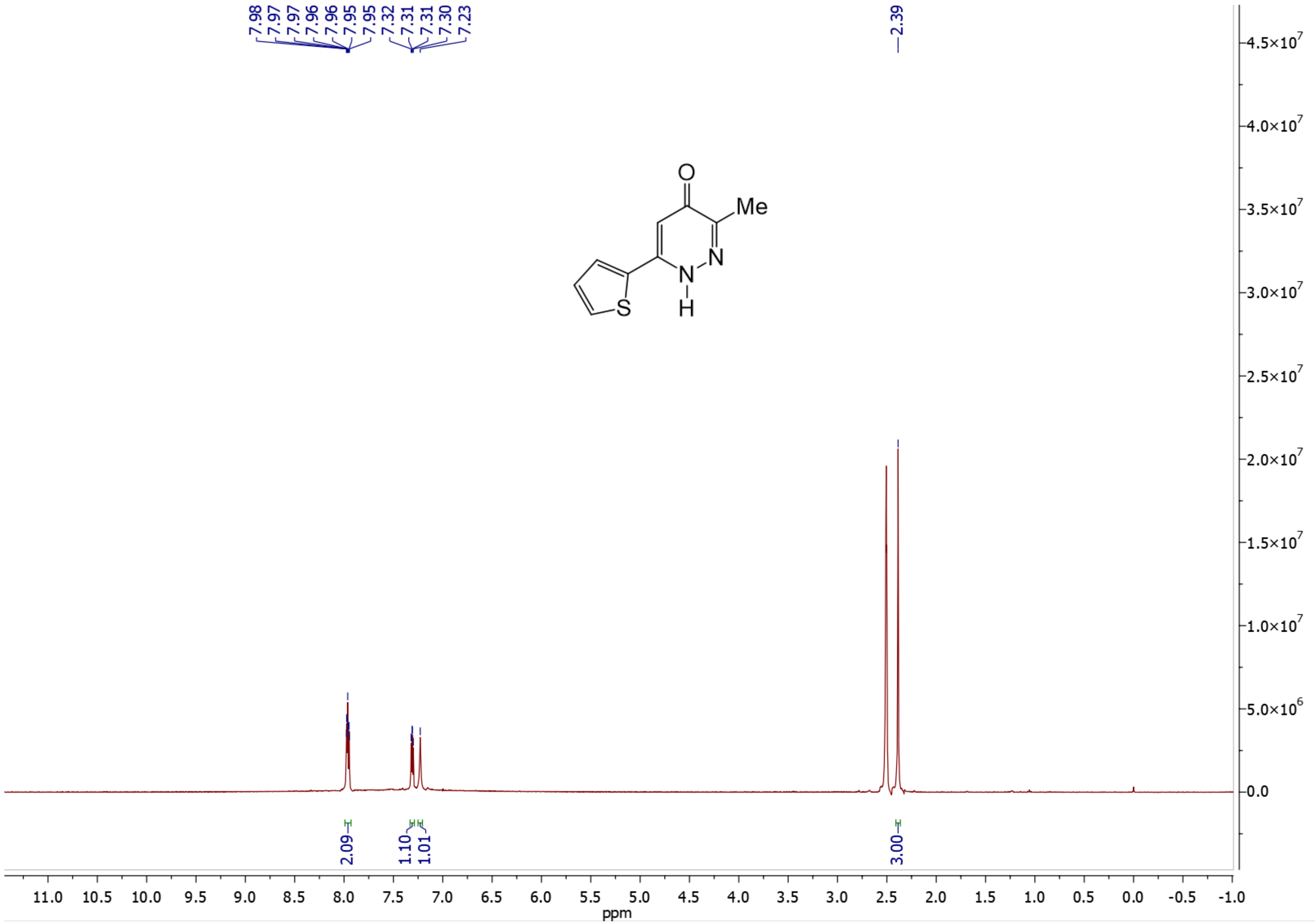

Figure S47. ^{13}C NMR spectrum of compound **8**.

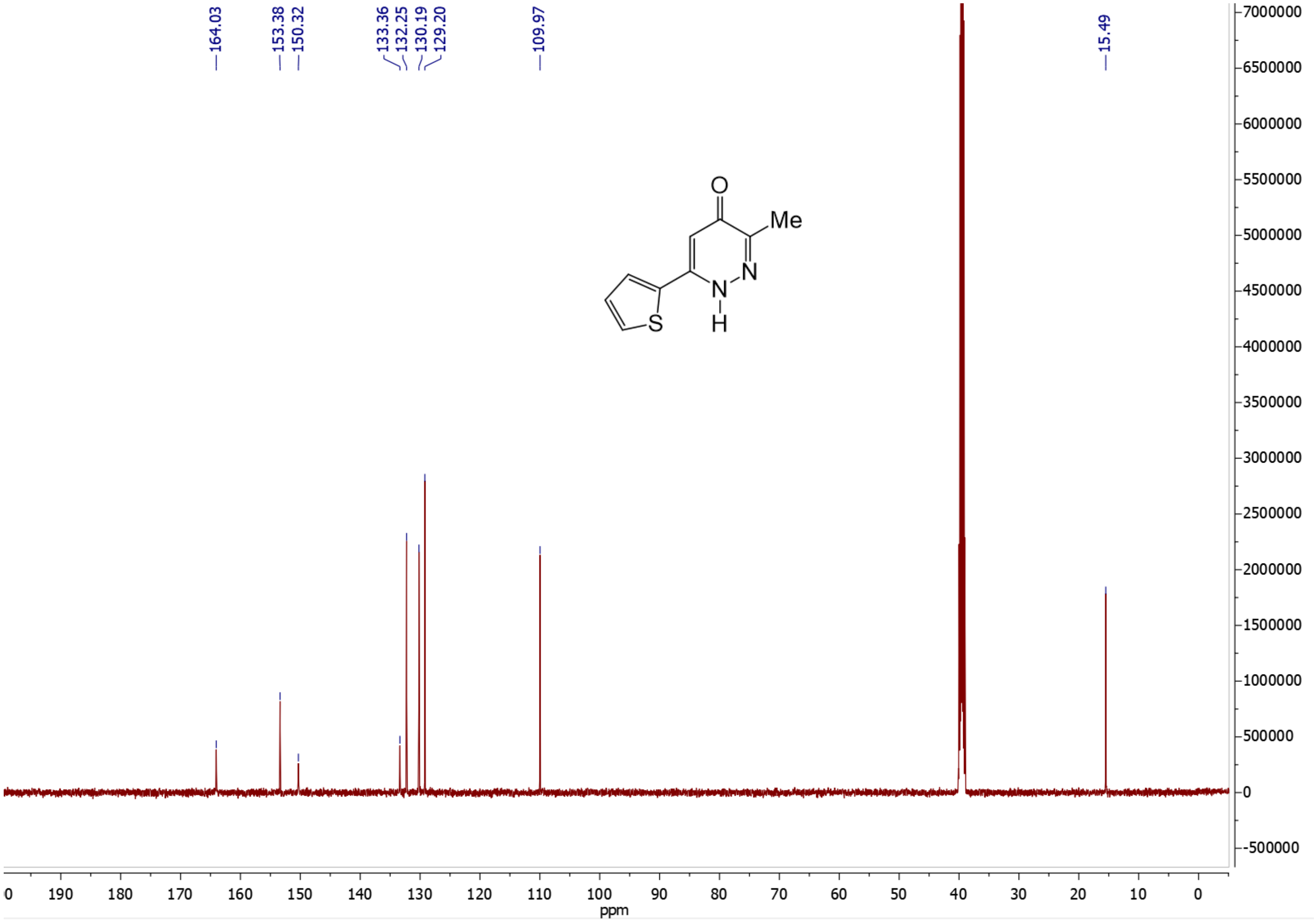

Figure S48. ^{1}H NMR spectrum of compound **9a**.

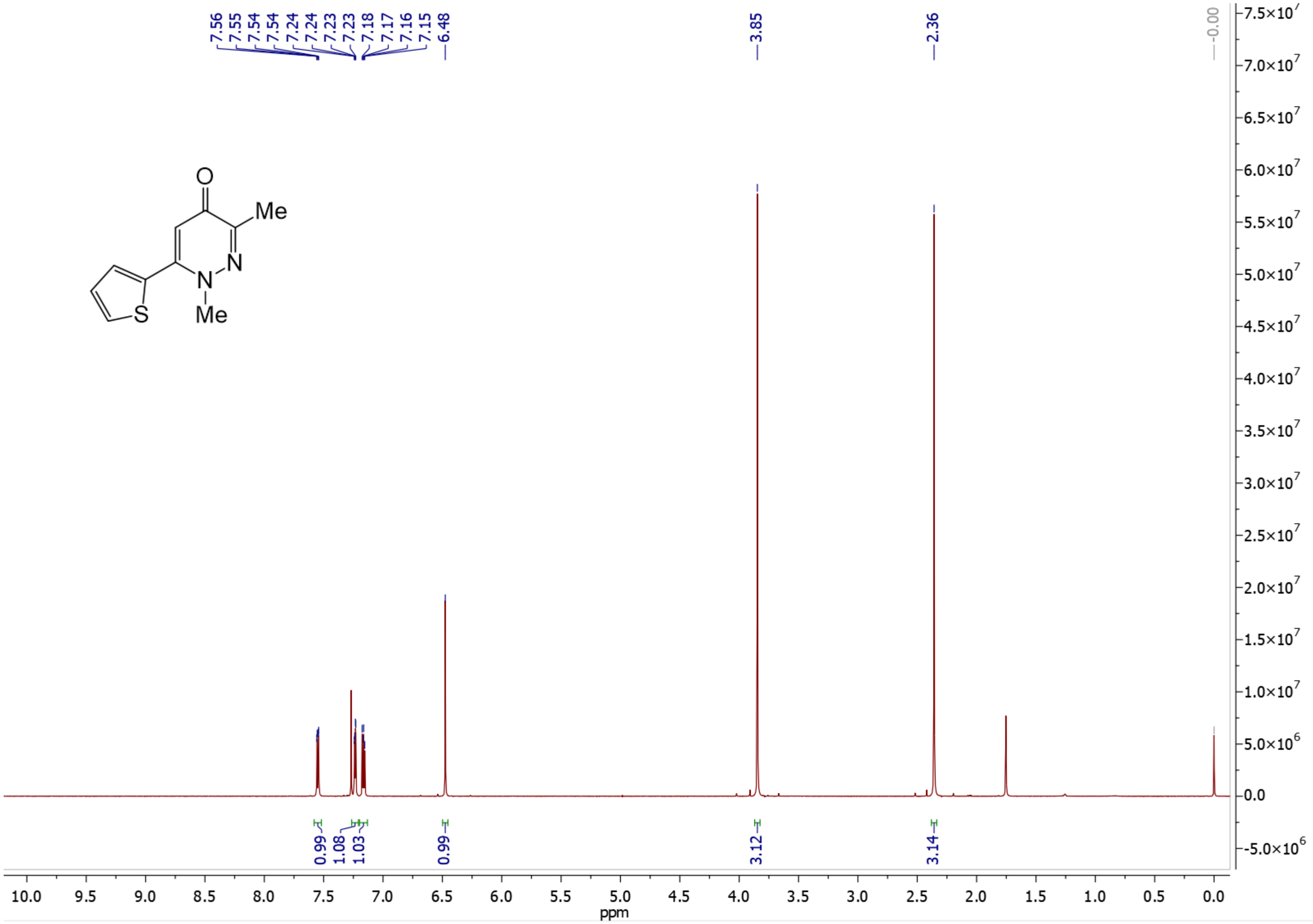

Figure S49. ^{13}C NMR spectrum of compound **9a.**

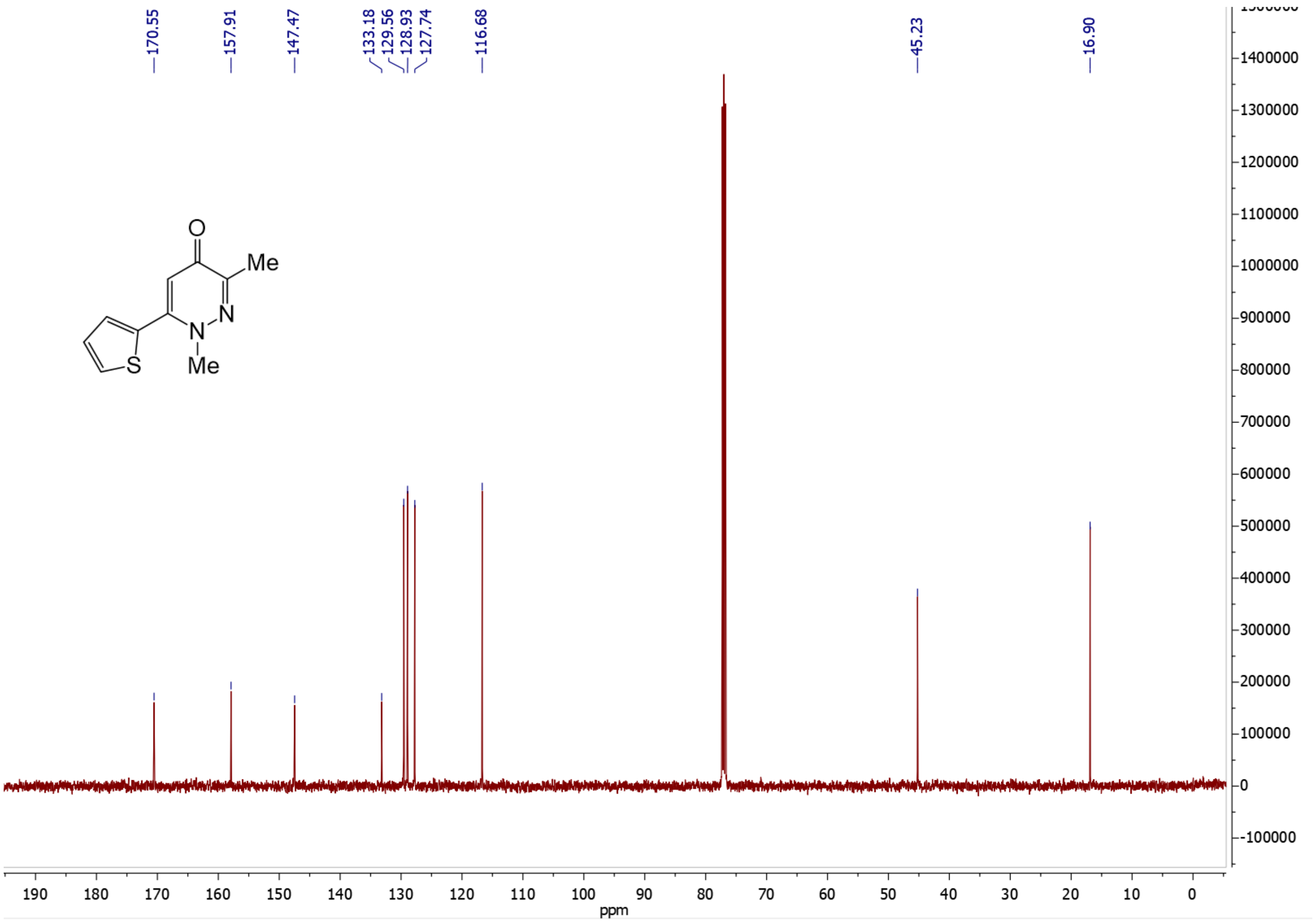

Figure S50. 1H NMR spectrum of compound **9b**.

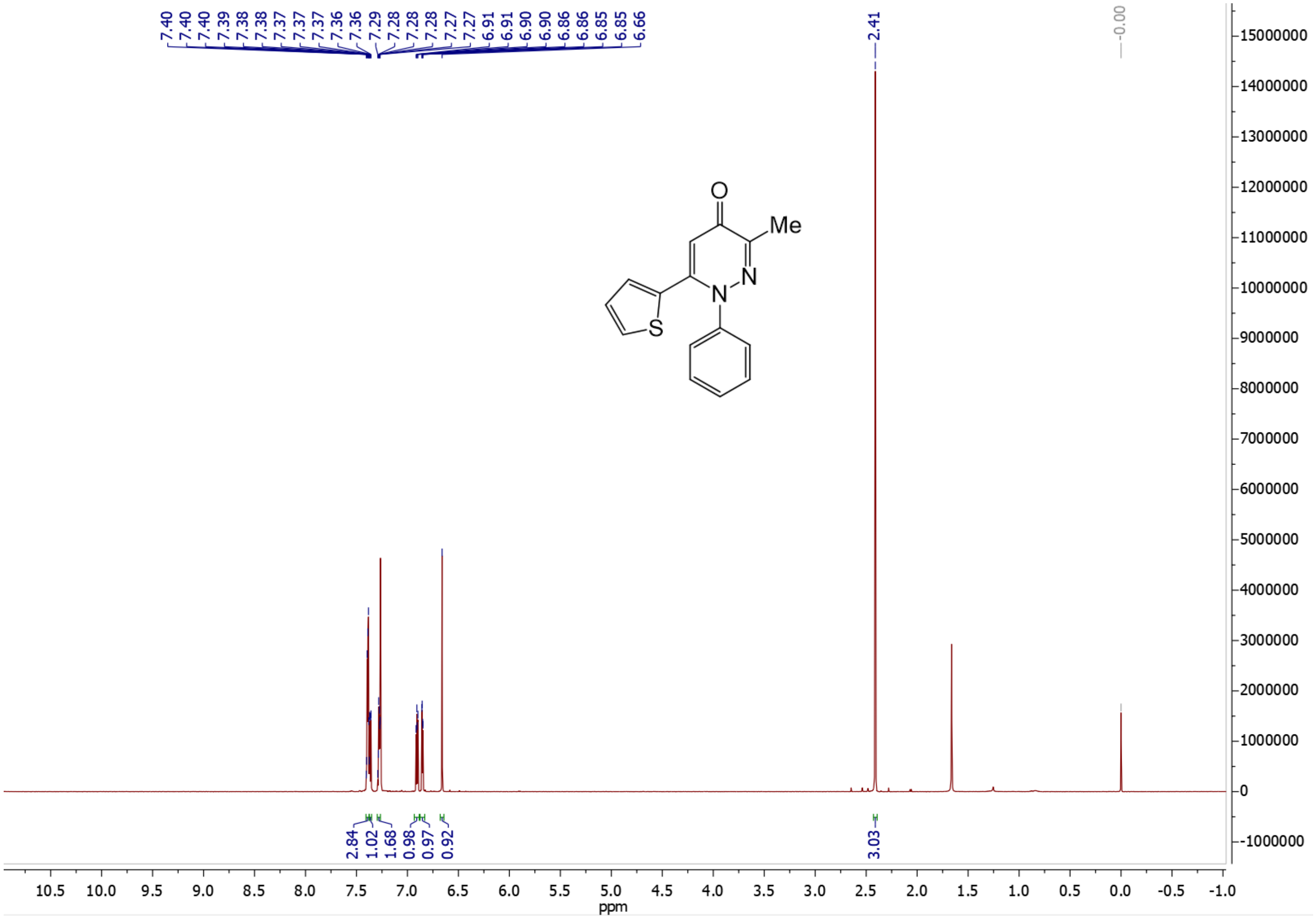

Figure S51. ^{13}C NMR spectrum of compound **9b**.

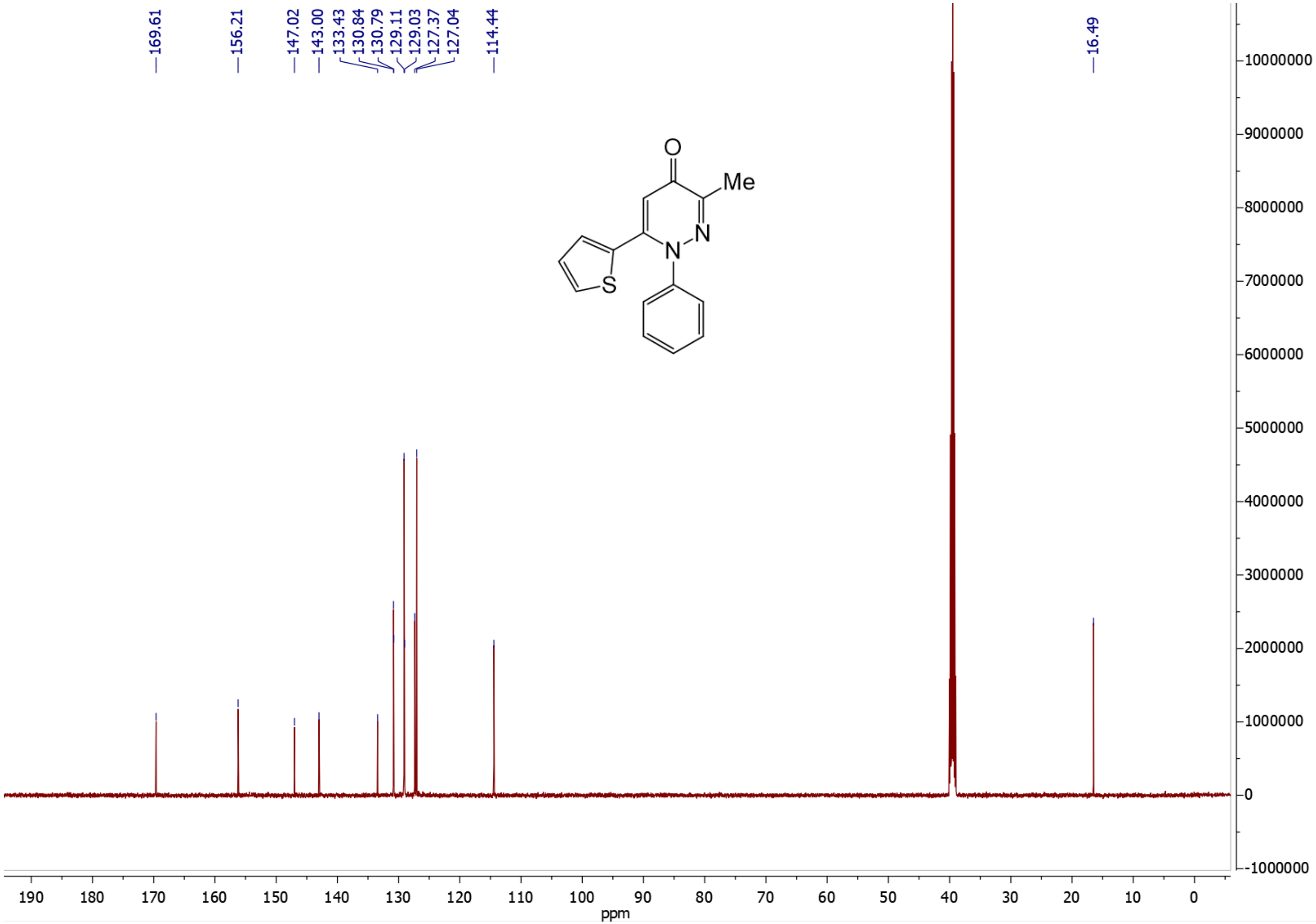

Figure S52. ^{1}H NMR spectrum of compound **10a**.

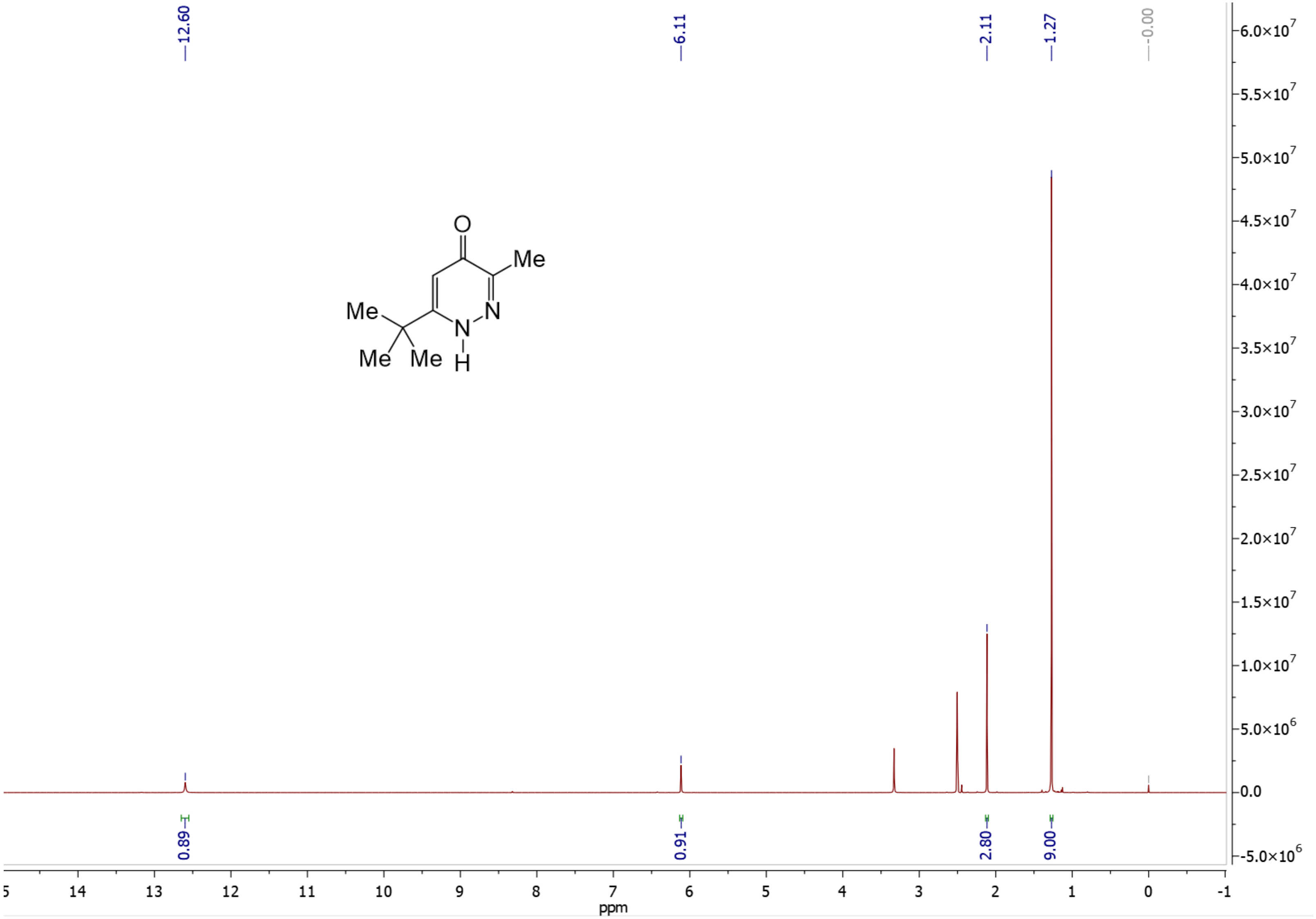

Figure S53. ^{13}C NMR spectrum of compound **10a**.

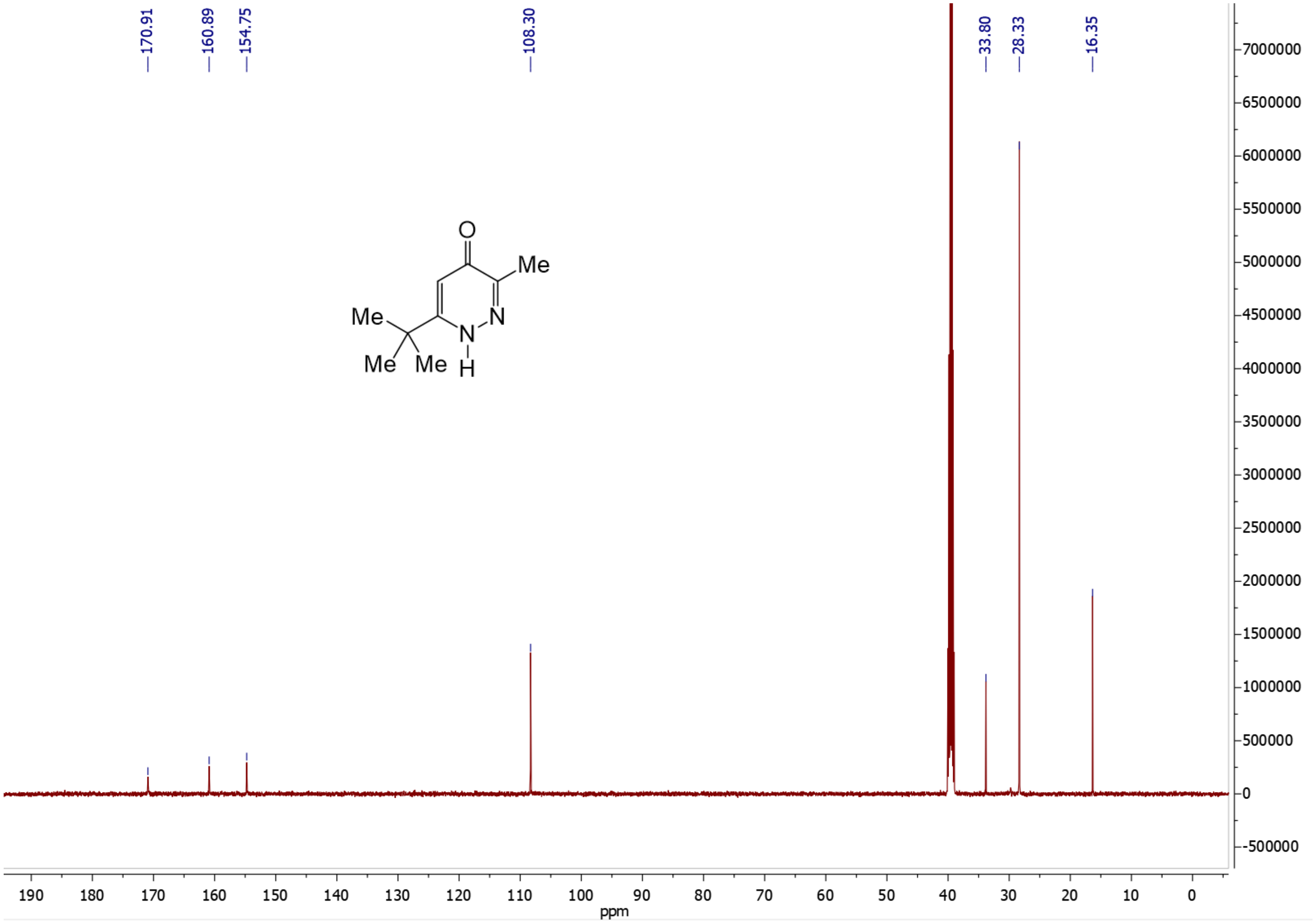

Figure S54. ^{1}H NMR spectrum of compound **10b**.

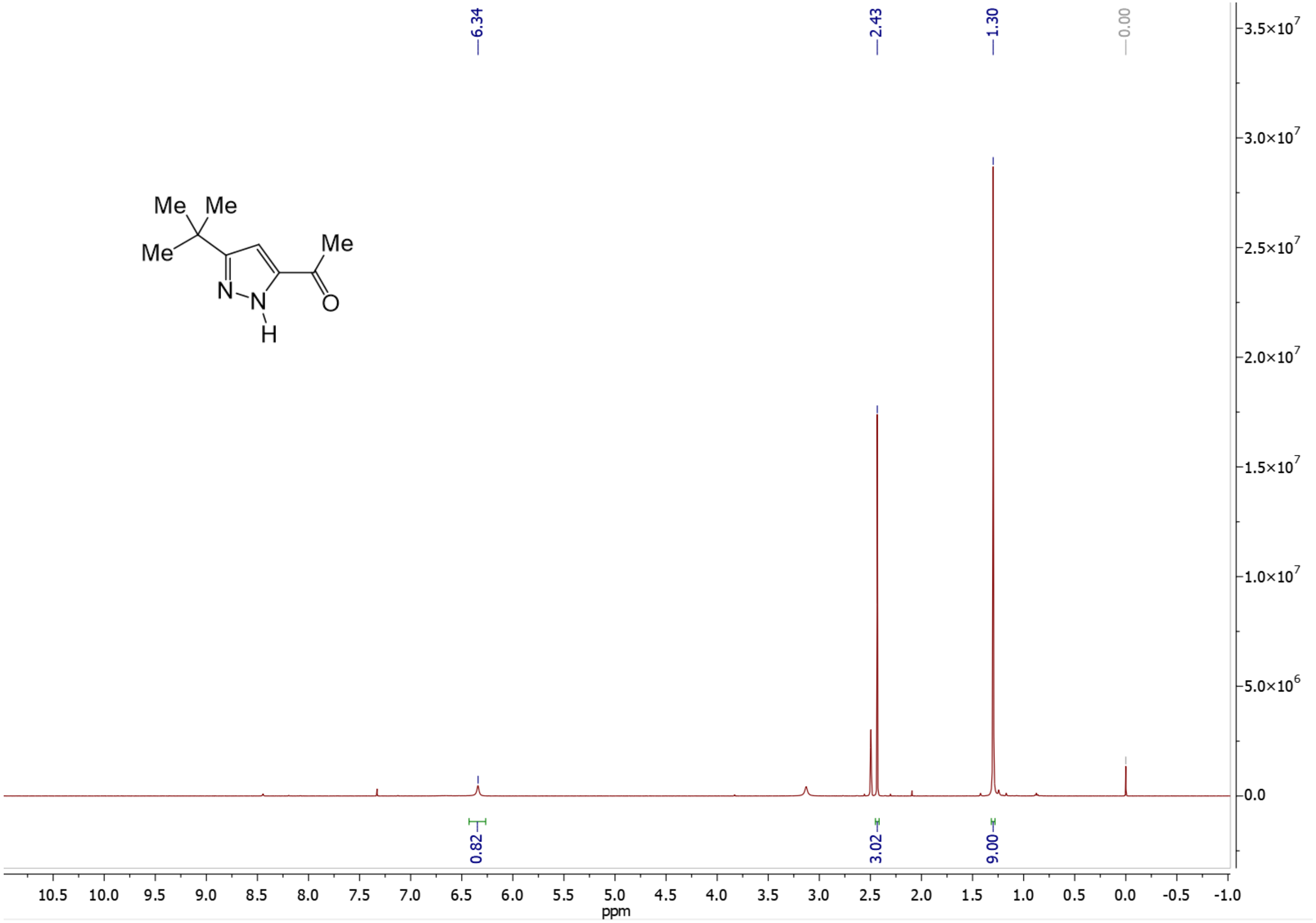

Figure S55. ^{13}C NMR spectrum of compound **10b**.

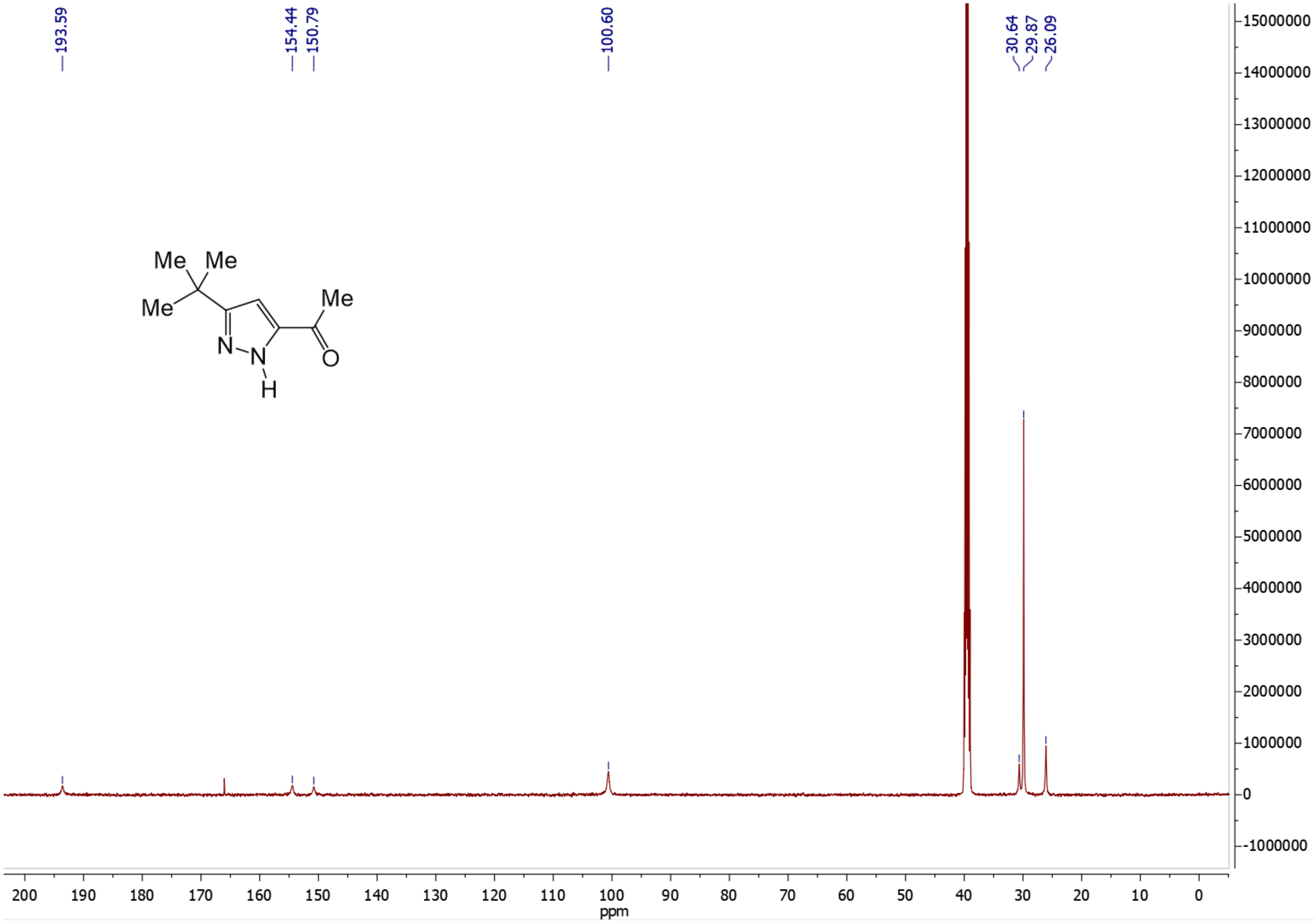

Figure S56. ^{1}H NMR spectrum of compound **11**.

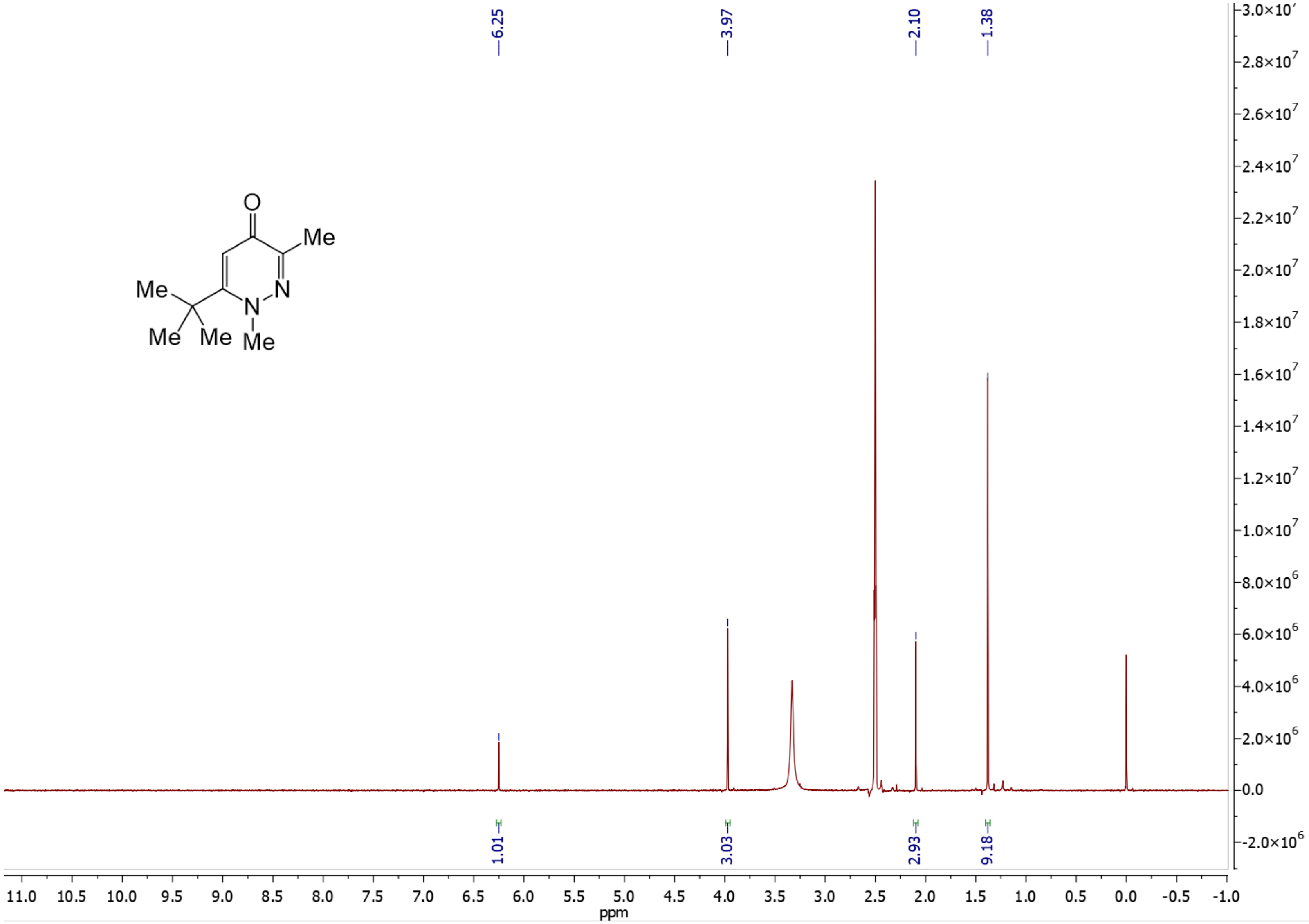

Figure S57. ^{13}C NMR spectrum of compound **11**.

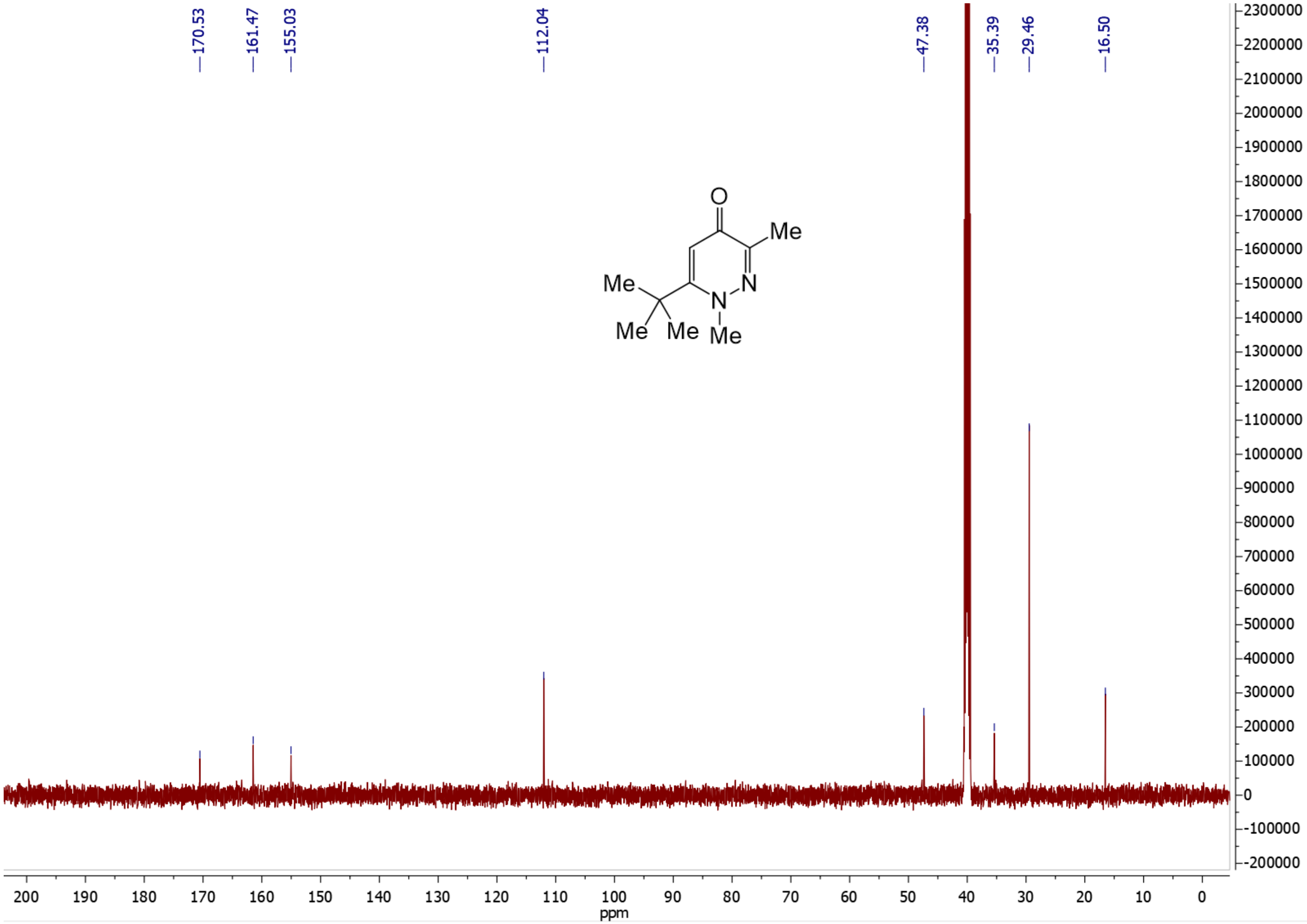

Figure S58. ^{1}H NMR spectrum of compound **12**.

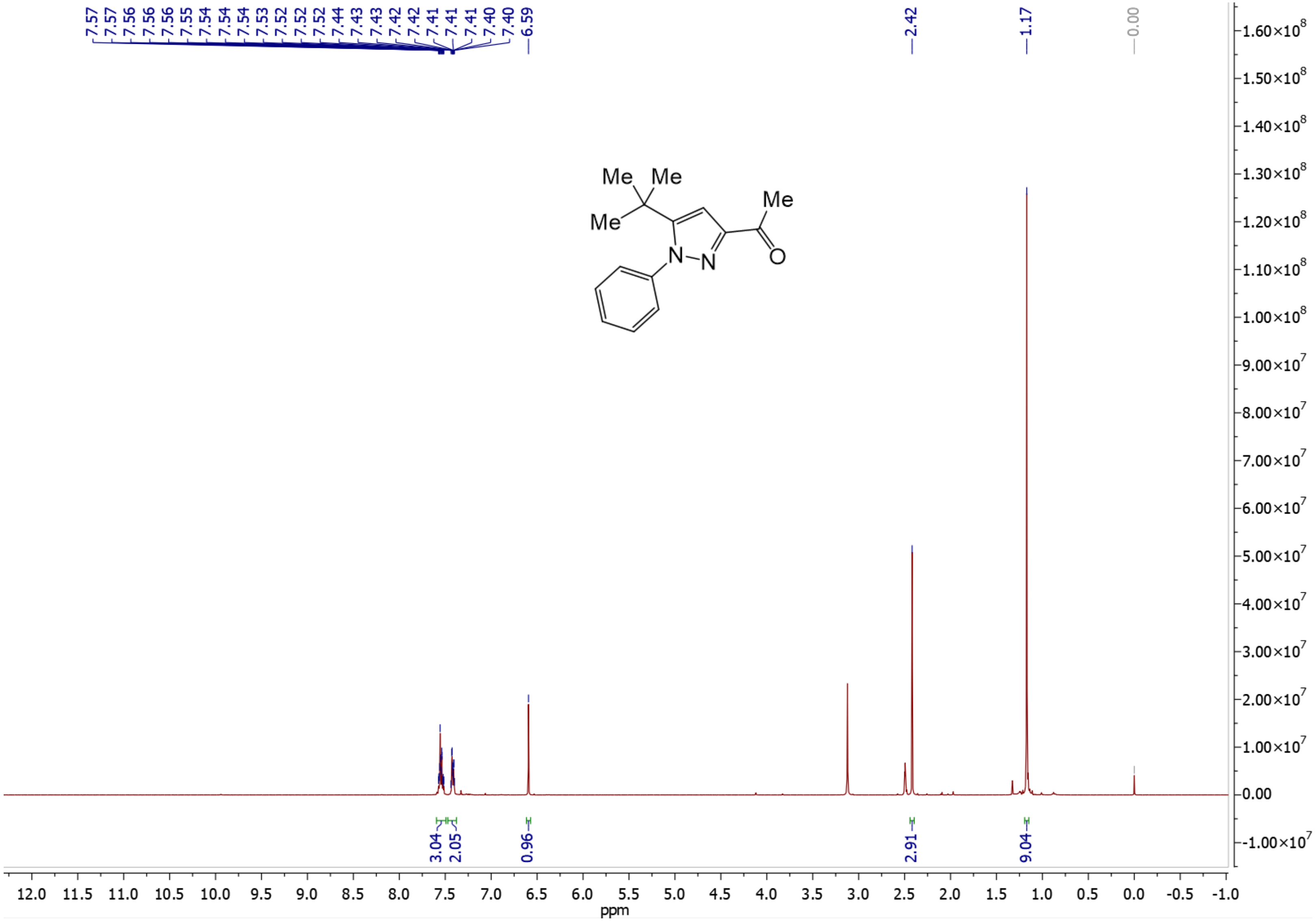

Figure S59. ^{13}C NMR spectrum of compound **12**.

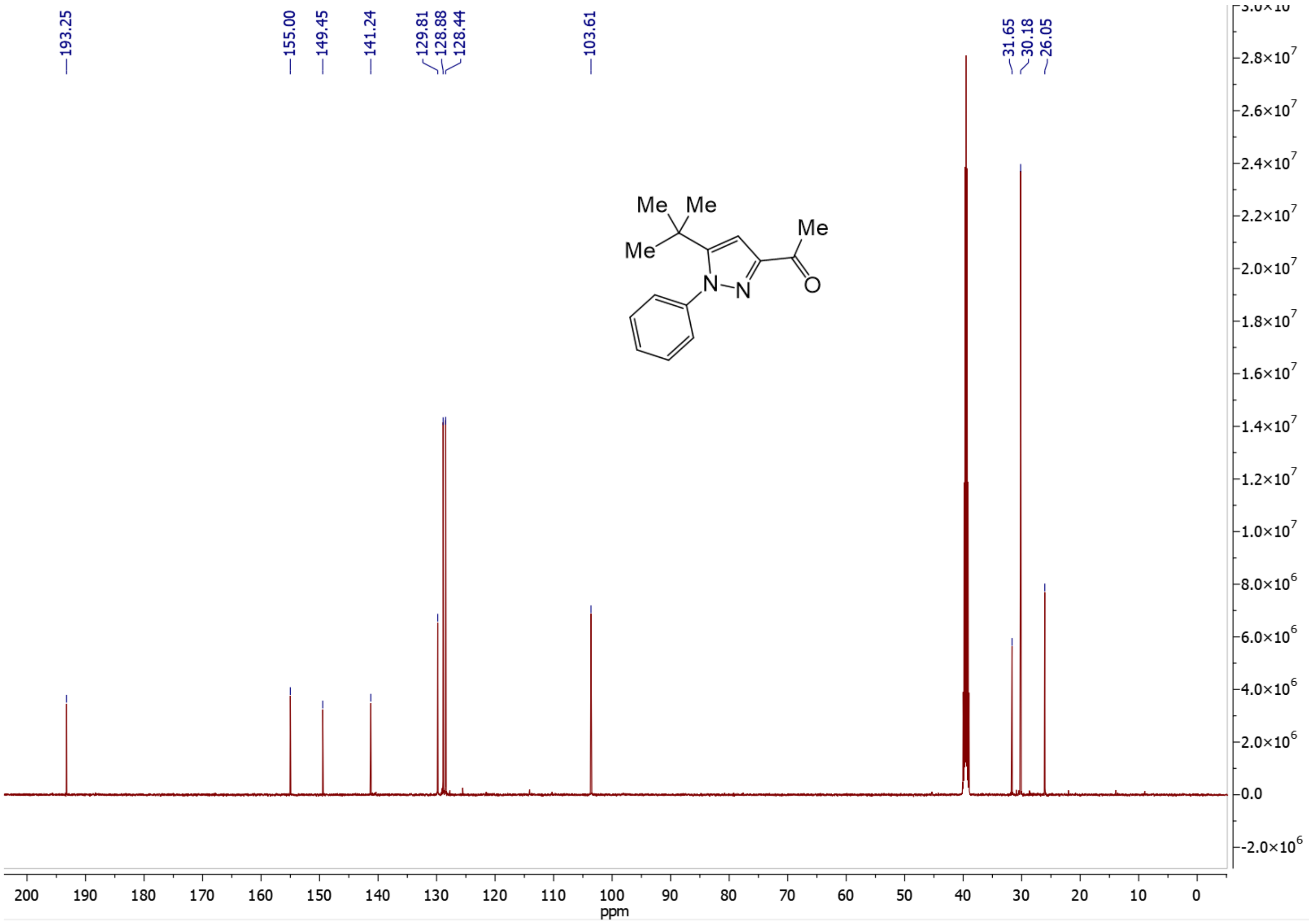

Figure S60. ^{1}H NMR spectrum of compound **13a**.

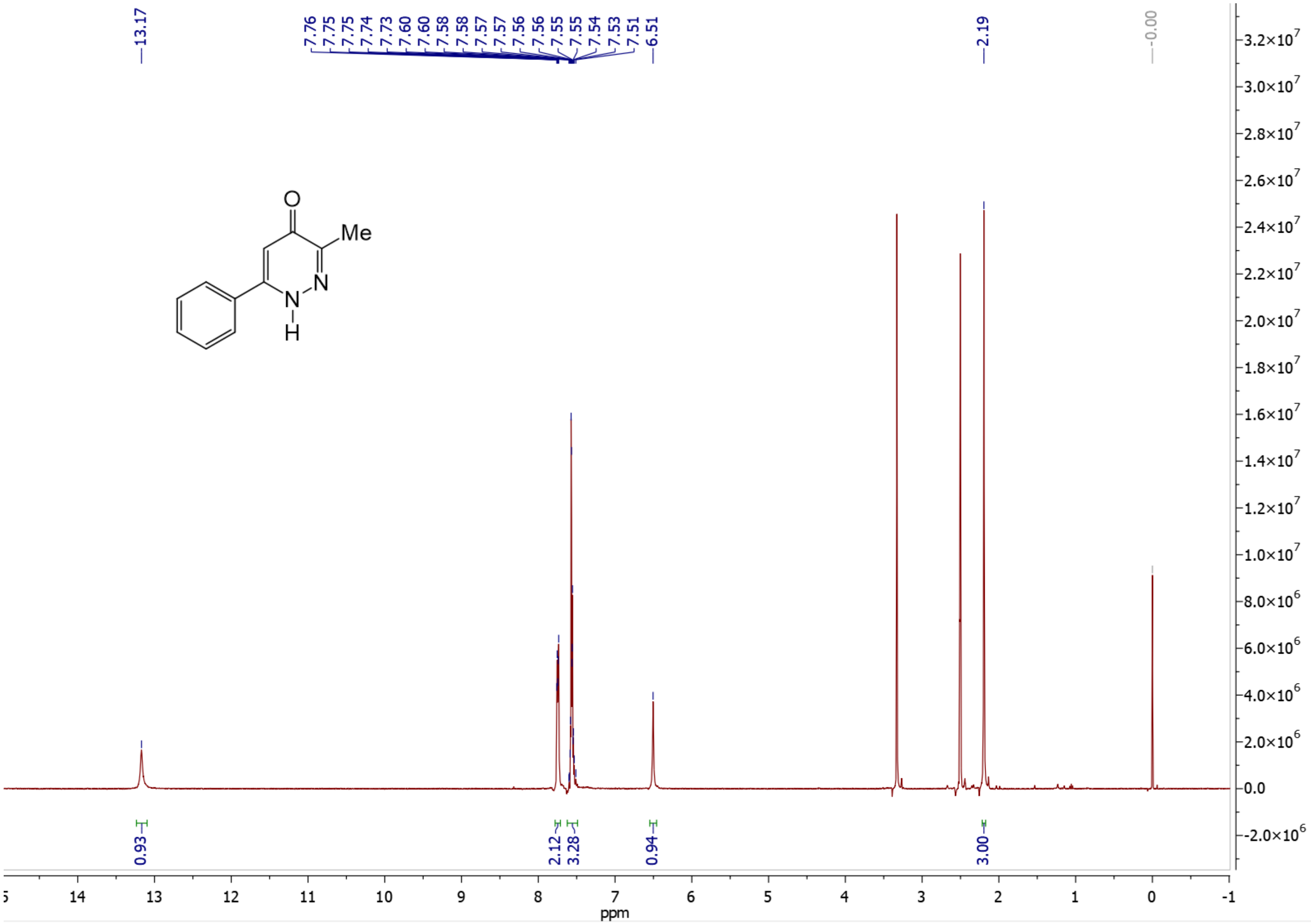

Figure S61. ^{13}C NMR spectrum of compound **13a**.

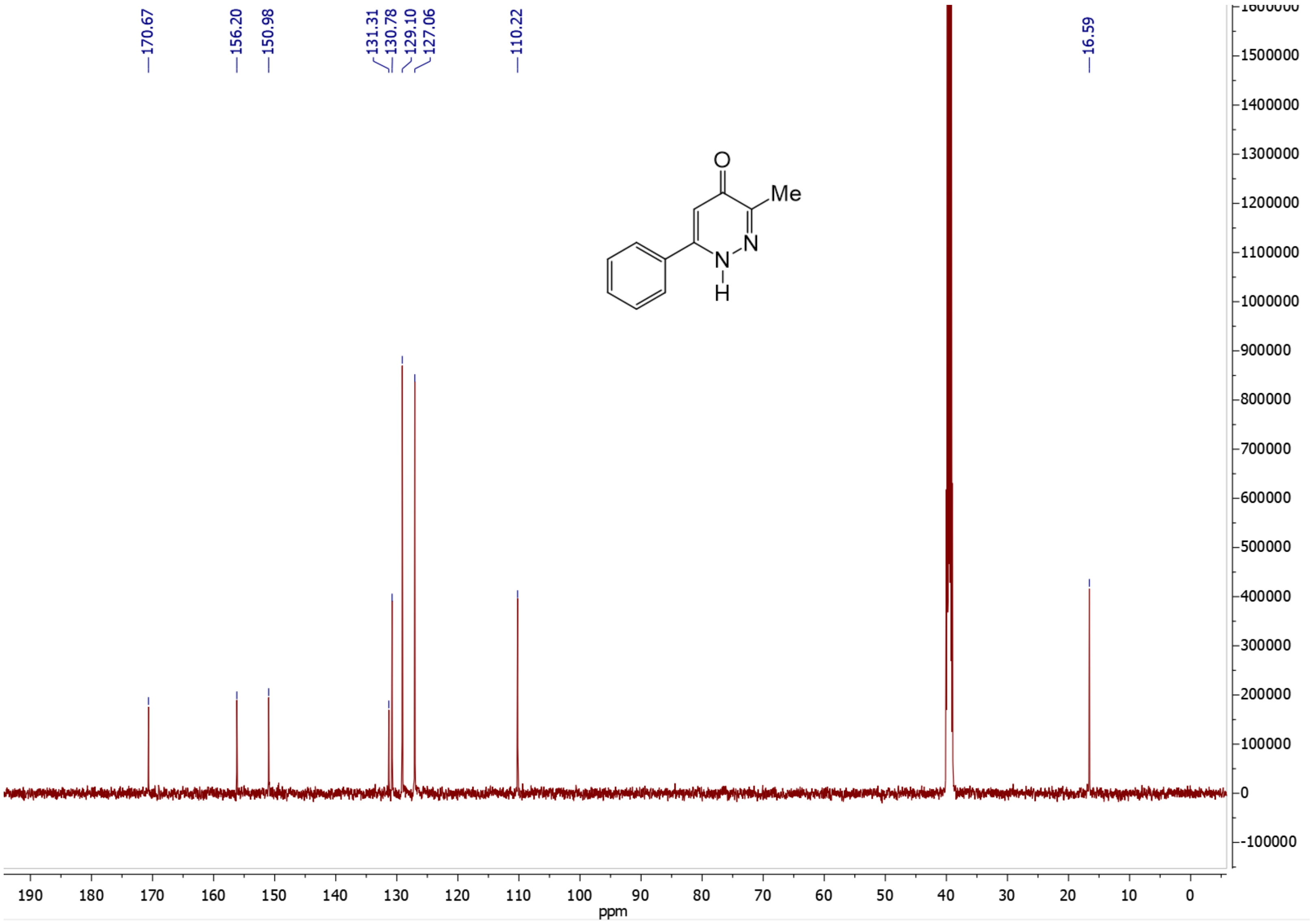

Figure S62. ^{1}H NMR spectrum of compound **13b**.

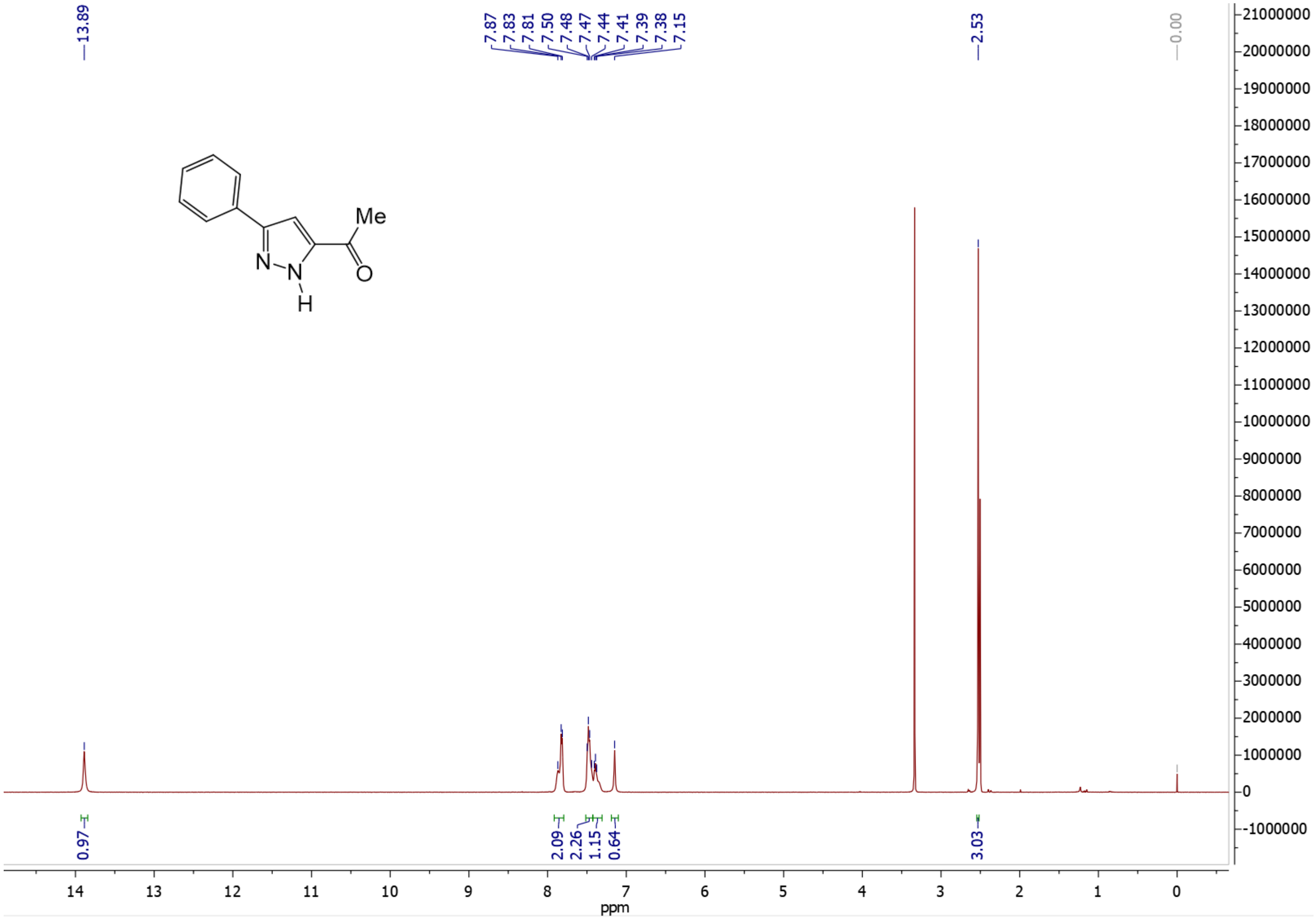

Figure S63. ^{13}C NMR spectrum of compound **13b**.

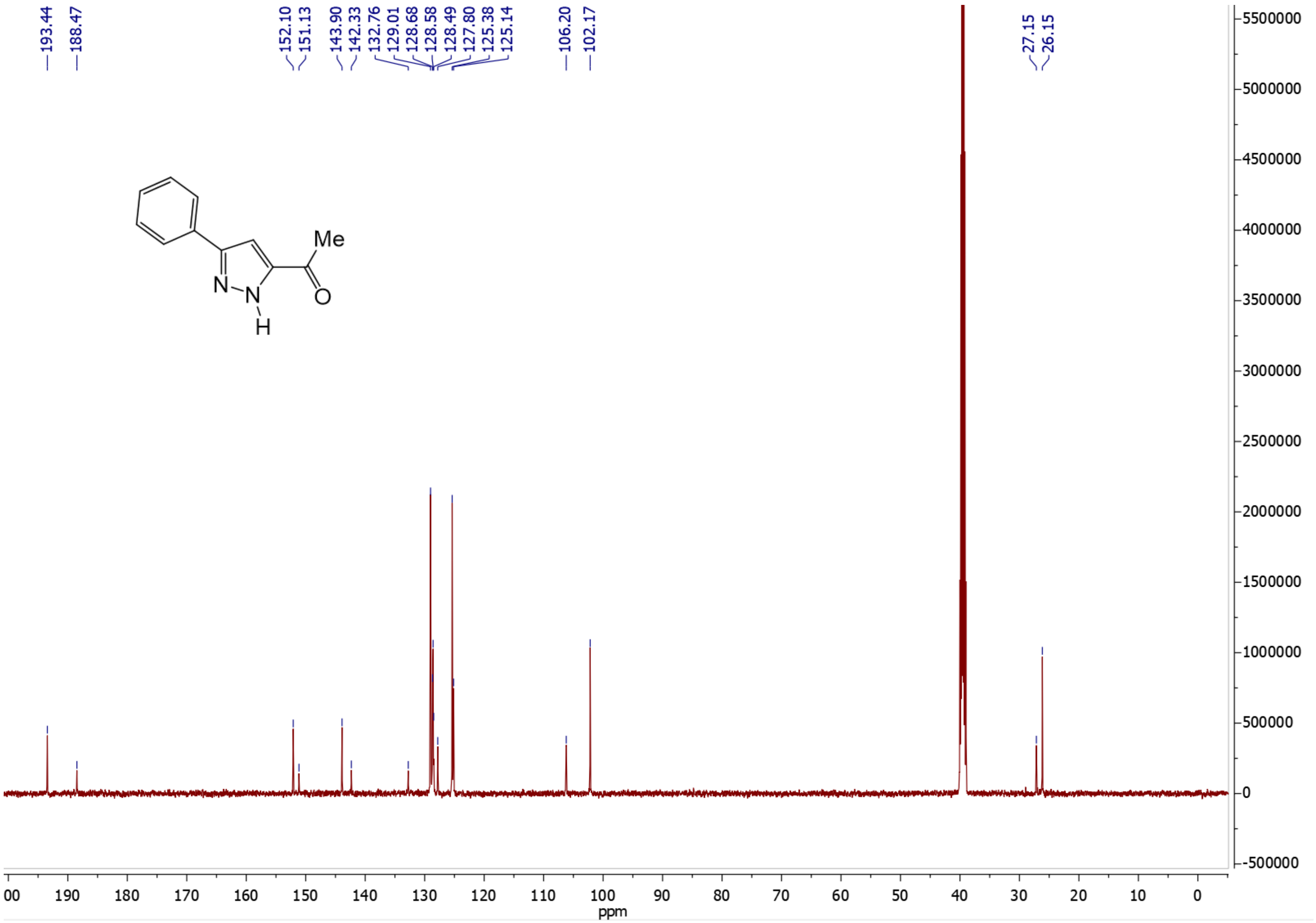

Figure S64. ^{1}H NMR spectrum of compound **14**.

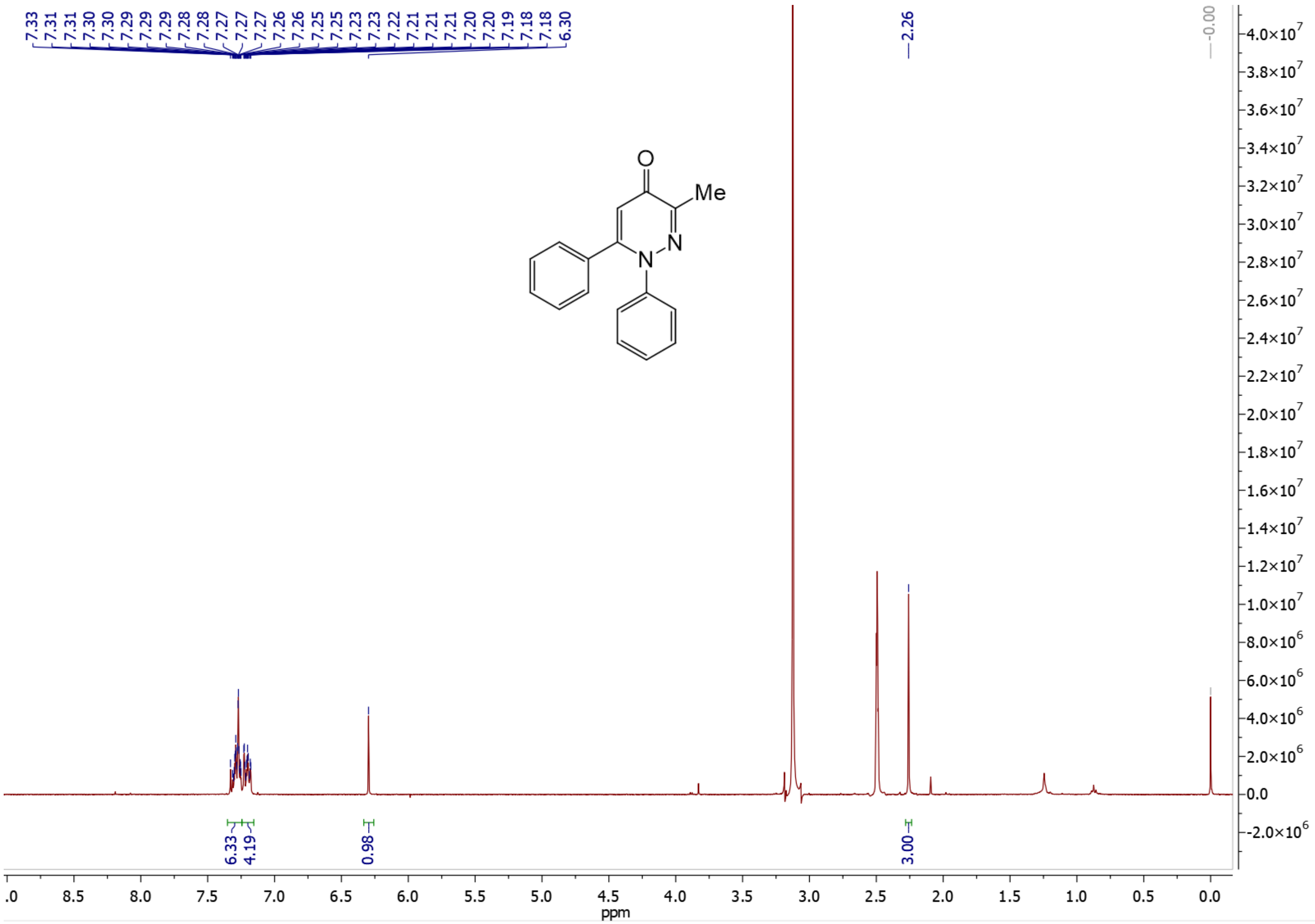

Figure S65. ^{13}C NMR spectrum of compound **14**.

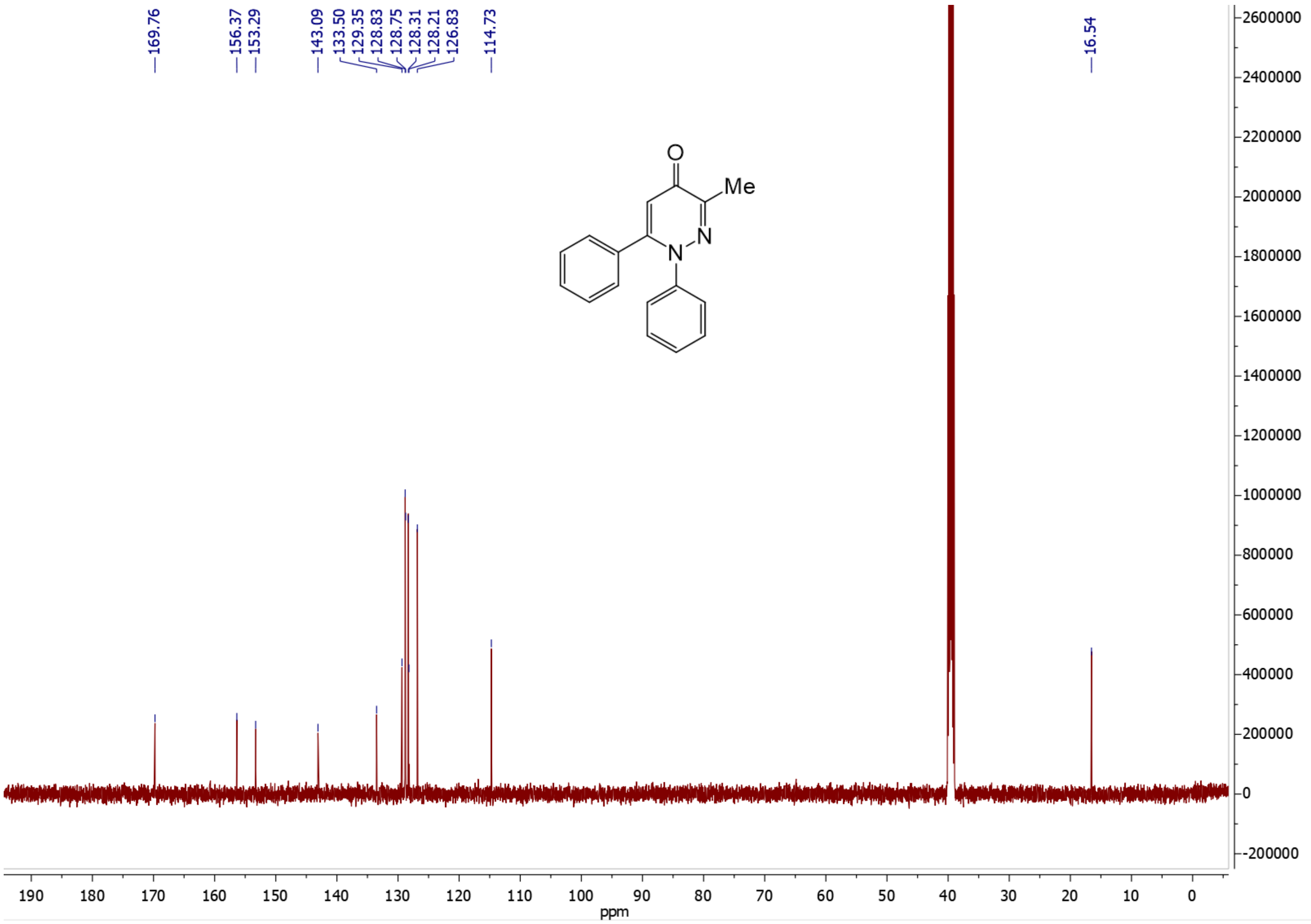

Figure S66. ^{1}H NMR spectrum of compound **15a**.

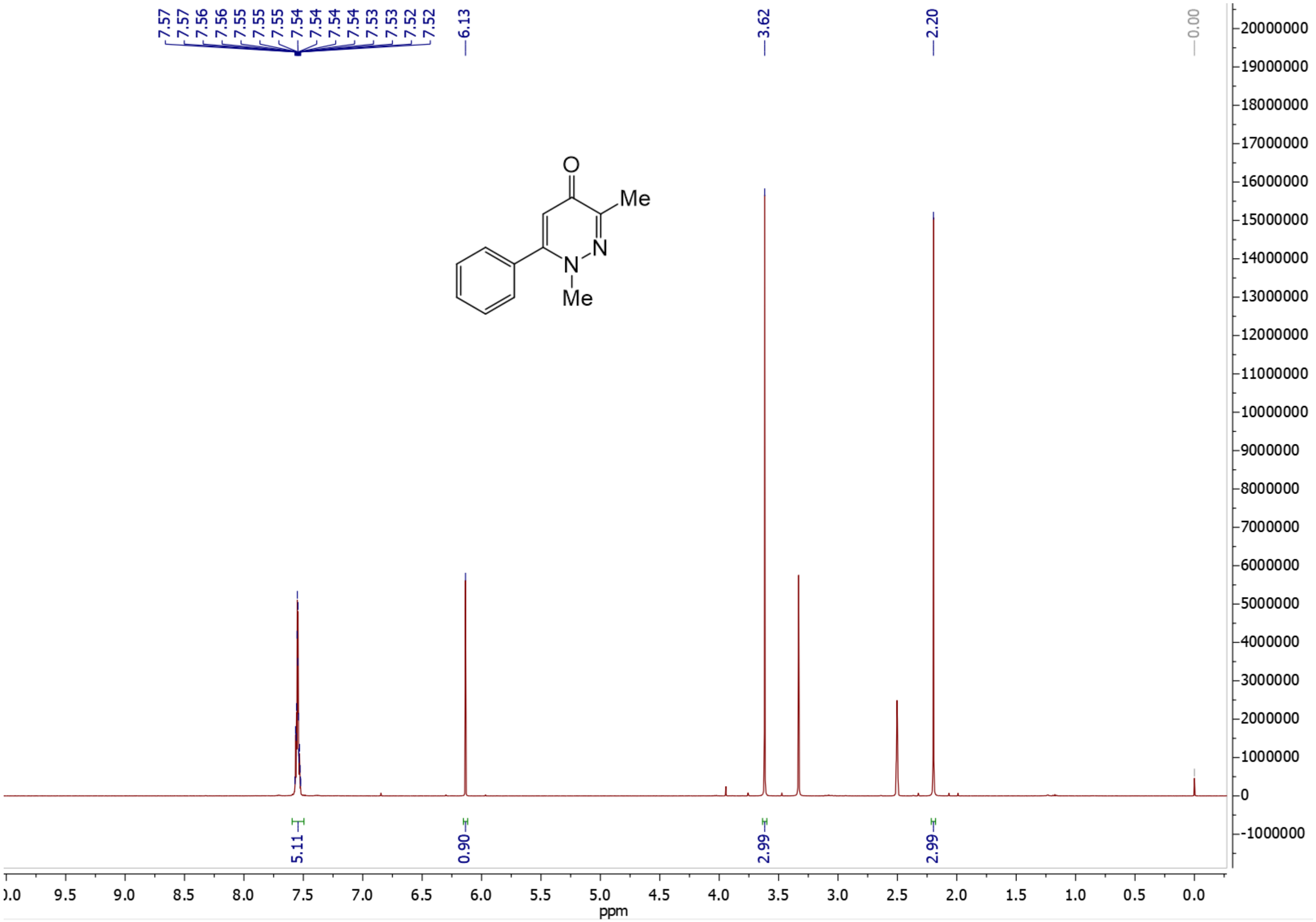

Figure S67. ^{13}C NMR spectrum of compound **15a**.

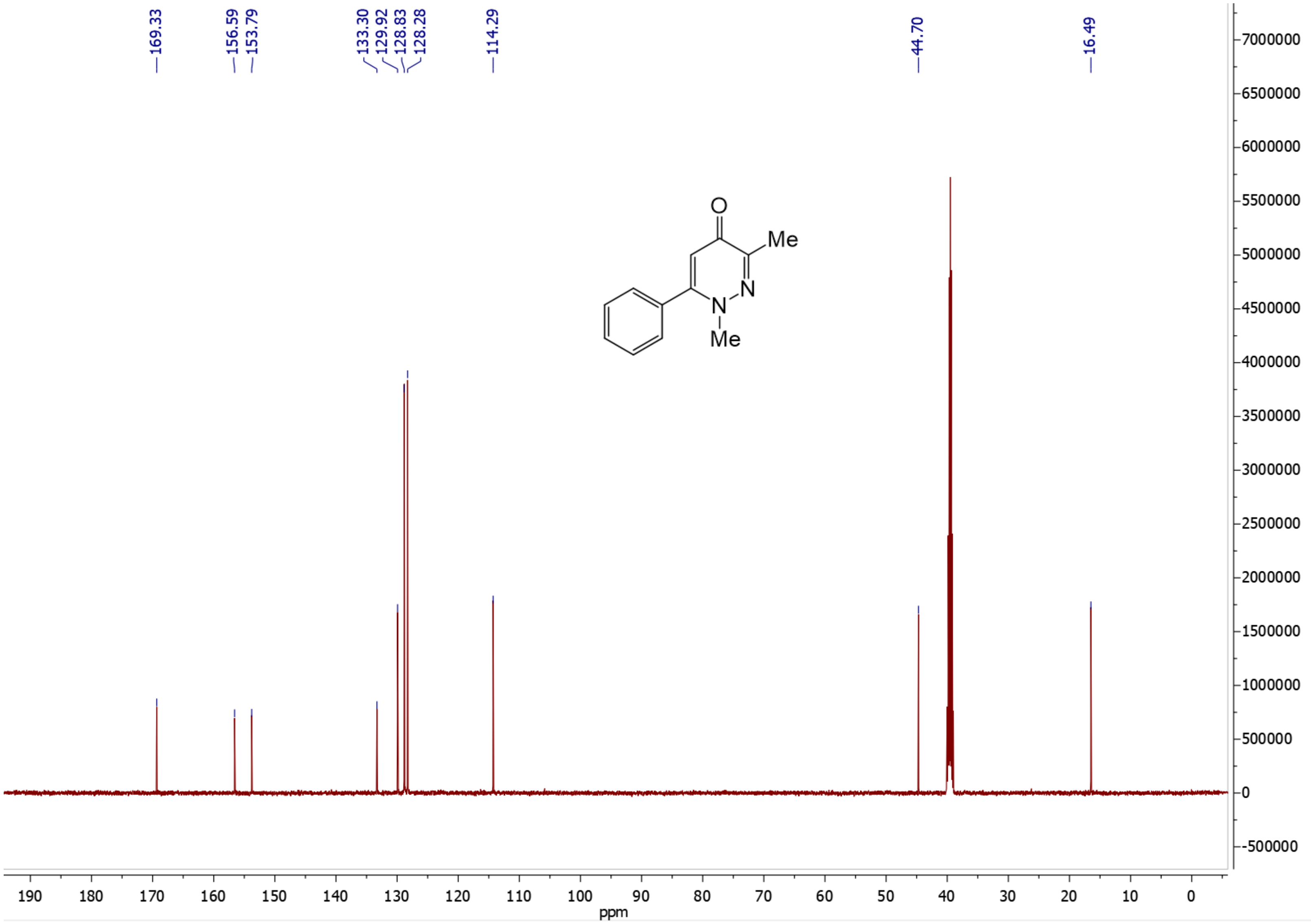

Figure S68. ¹H NMR spectrum of compound **15b**.

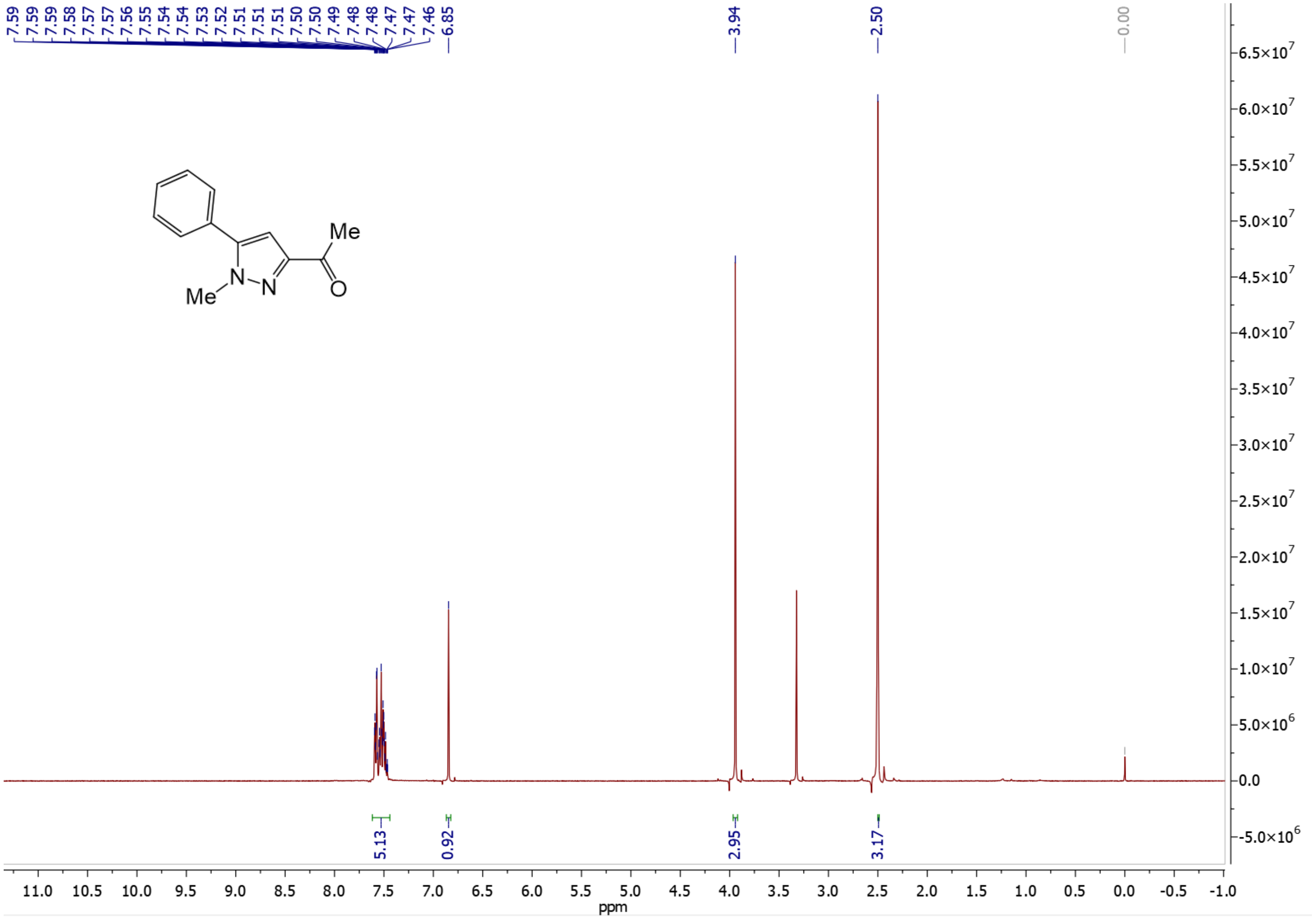

Figure S69. ^{13}C NMR spectrum of compound **15b**.

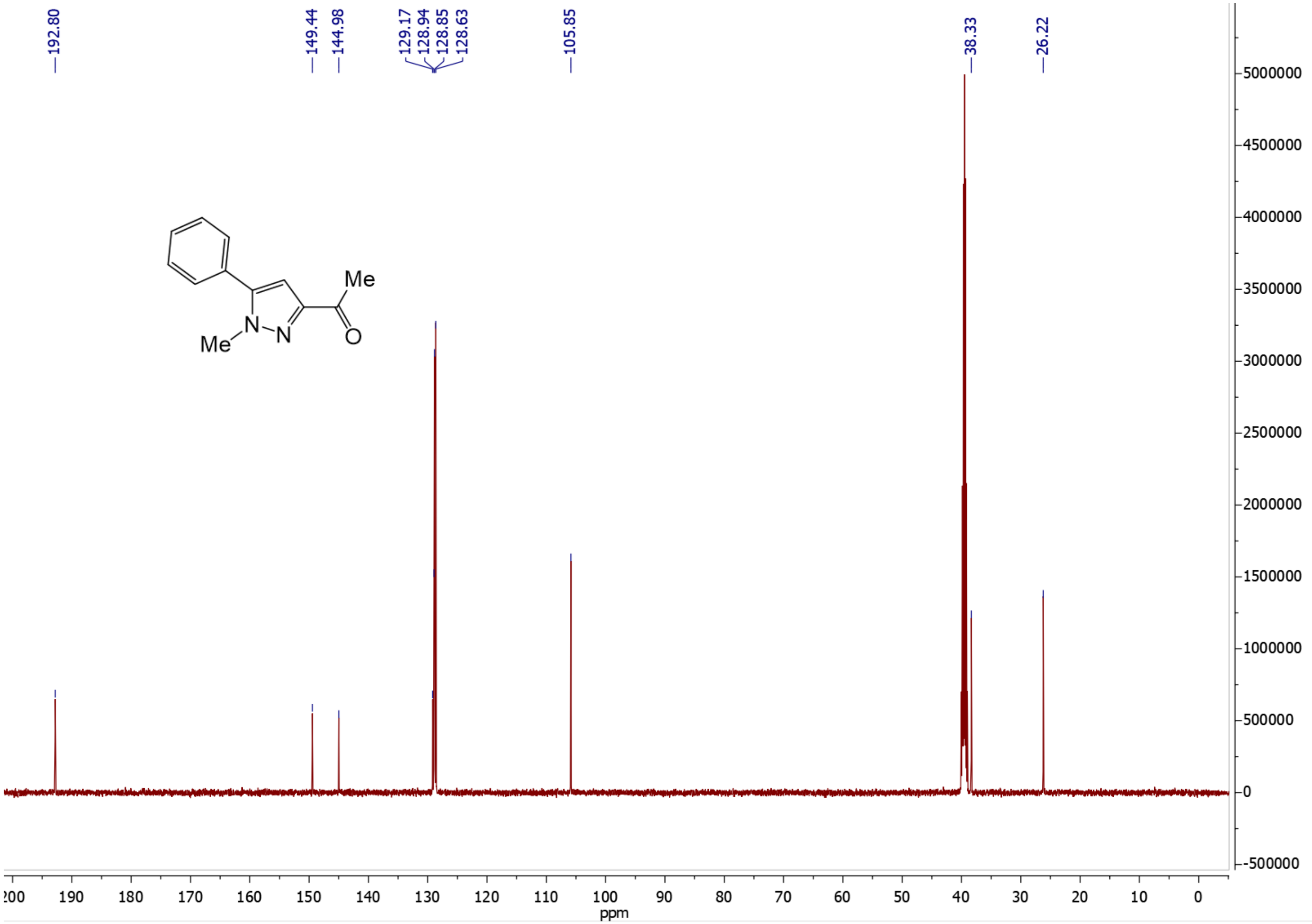

Figure S70. ¹H NMR spectrum of compound **16**.

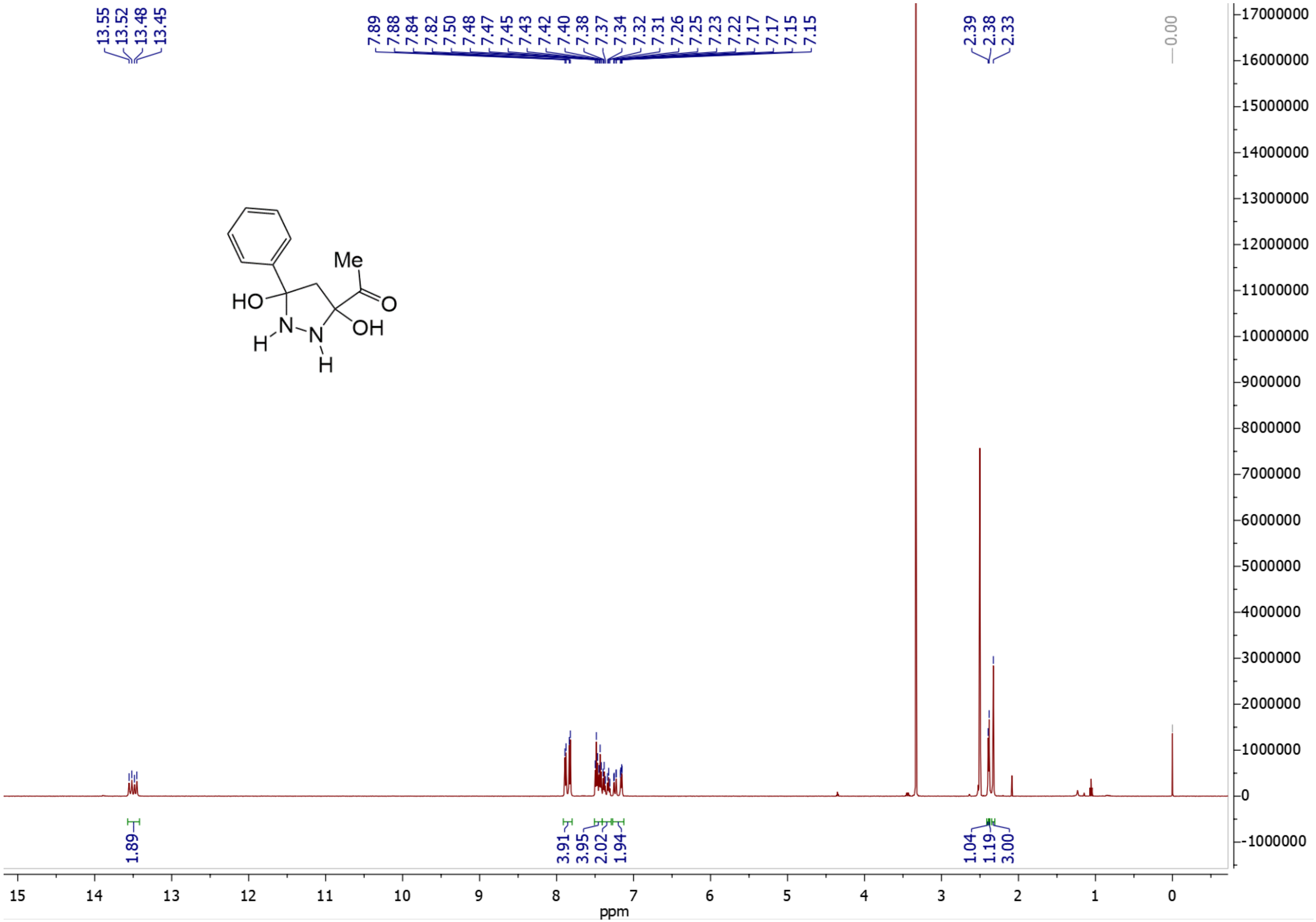

Figure S71. ¹H NMR spectrum of compound **17**.

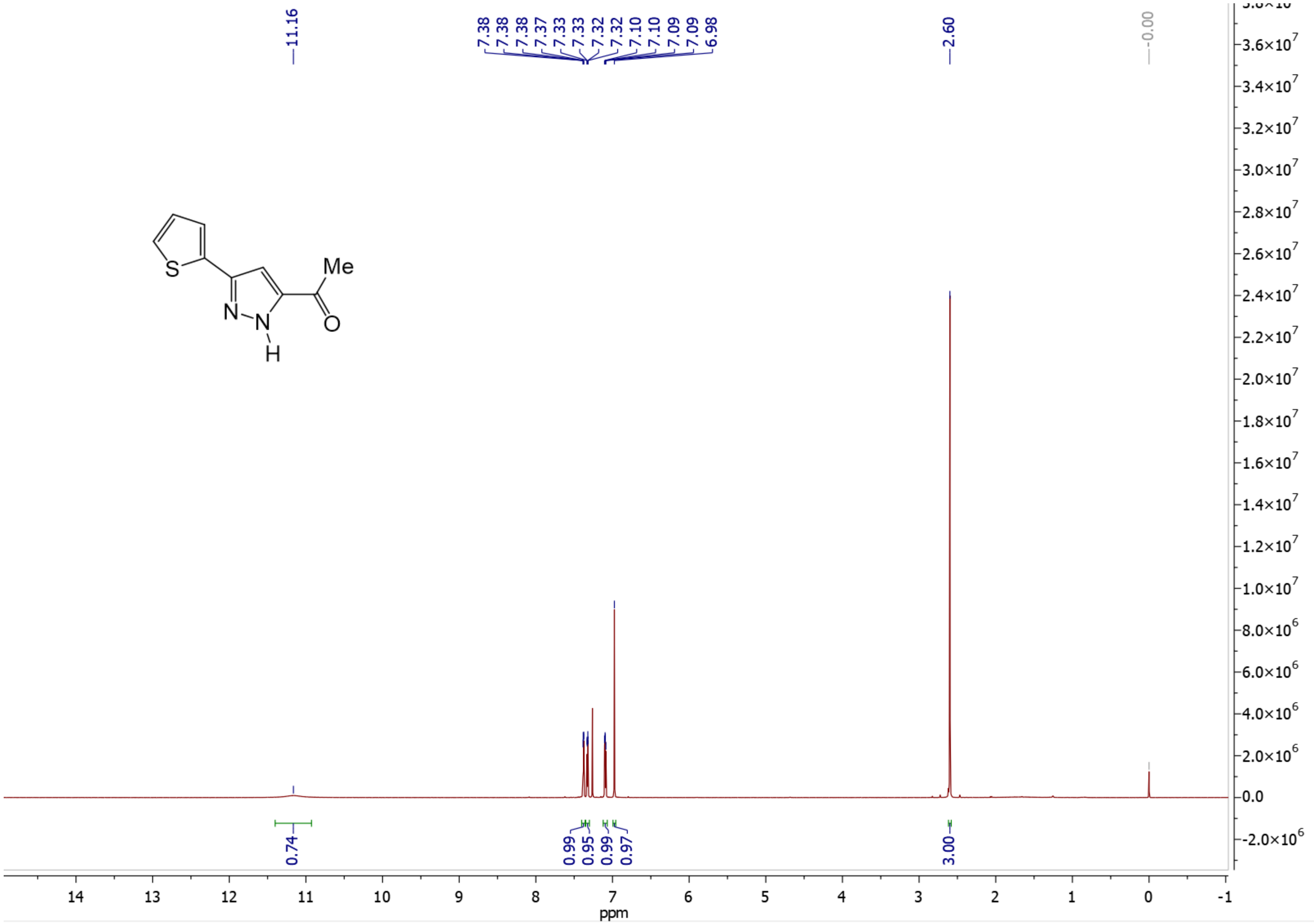

Figure S72. ^{13}C NMR spectrum of compound **17**.

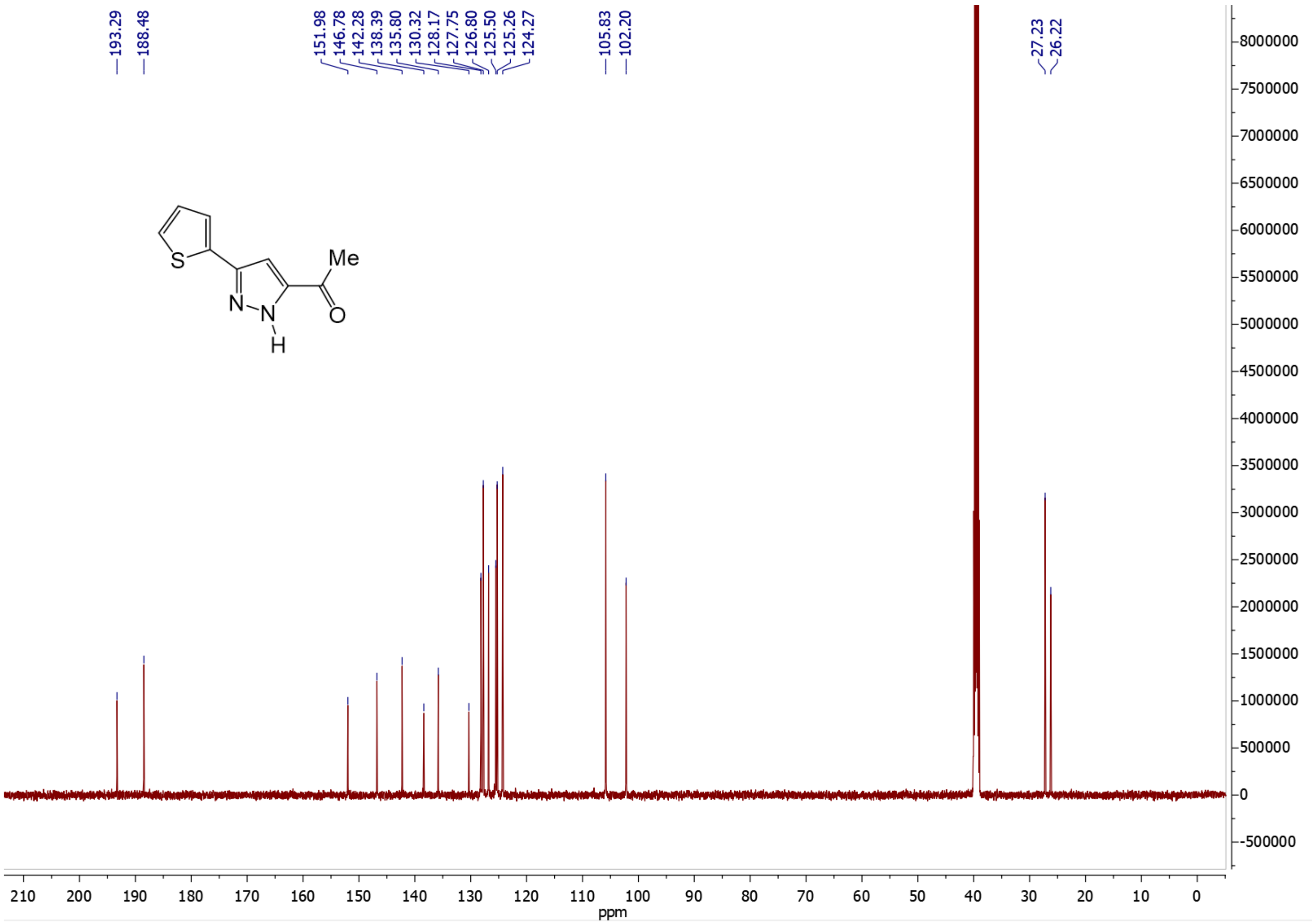

Figure S73. ^{1}H NMR spectrum of compound **18a**.

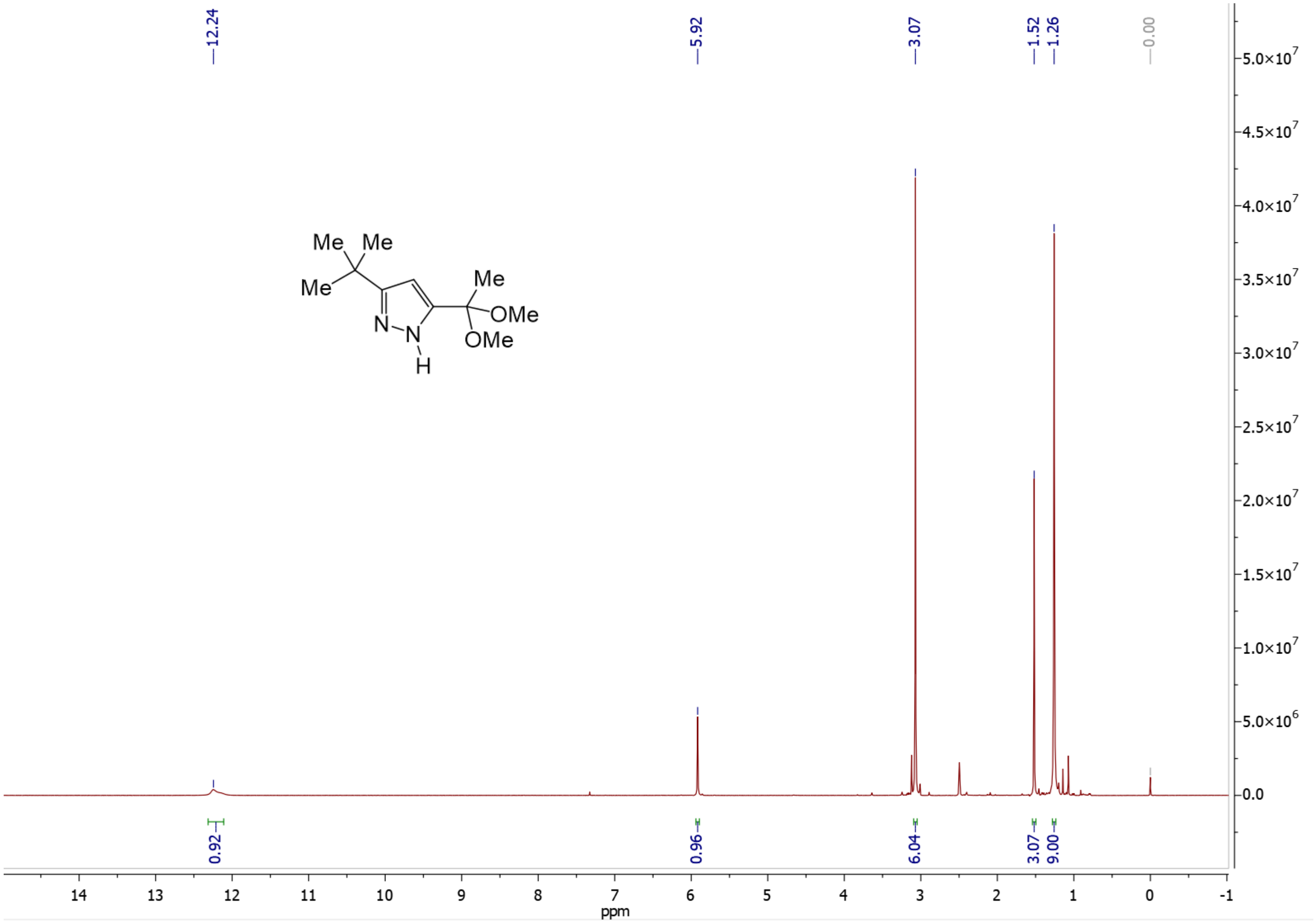

Figure S74. ^{13}C NMR spectrum of compound **18a**.

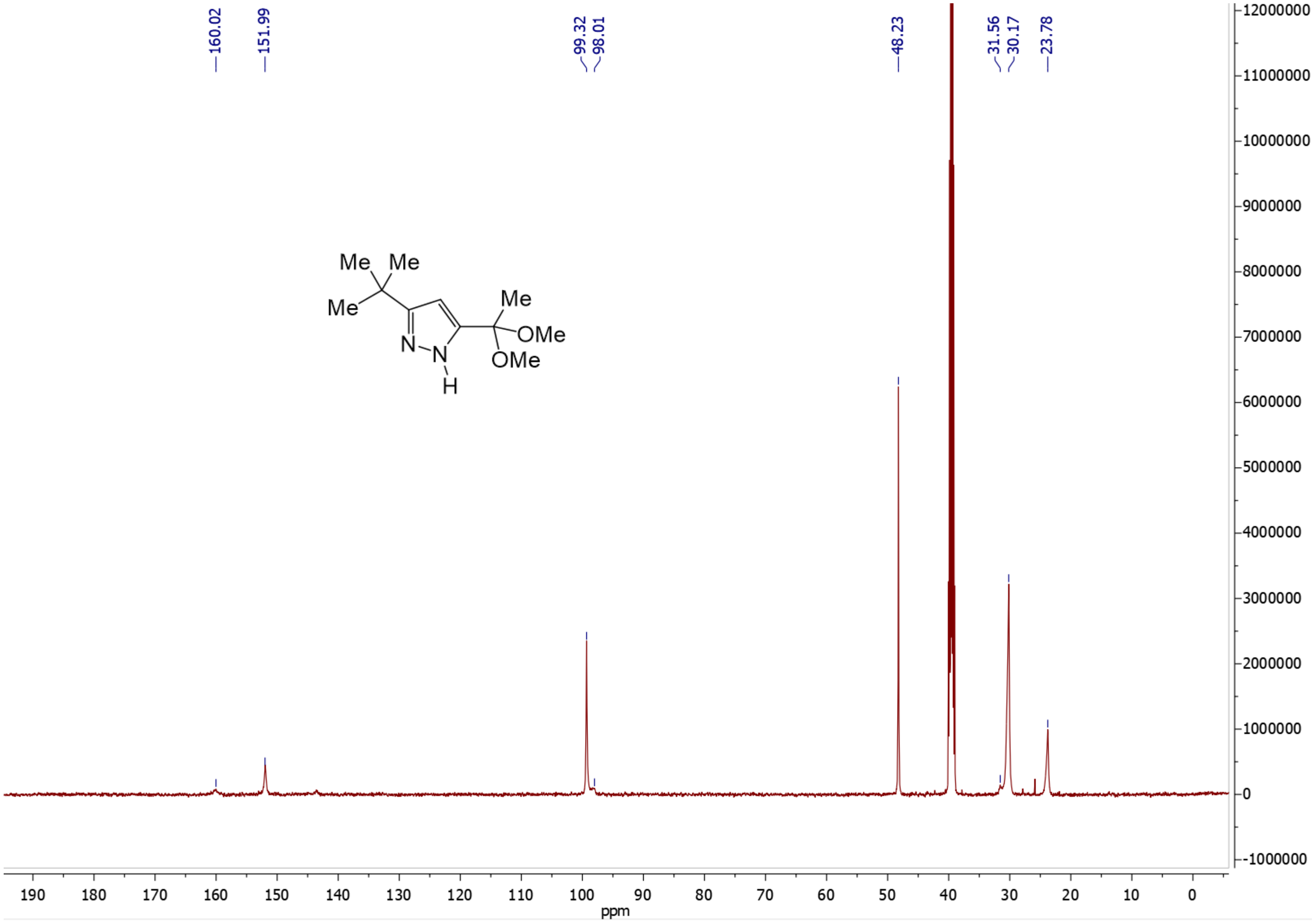

Figure S75. ^{1}H NMR spectrum of compound **18b**.

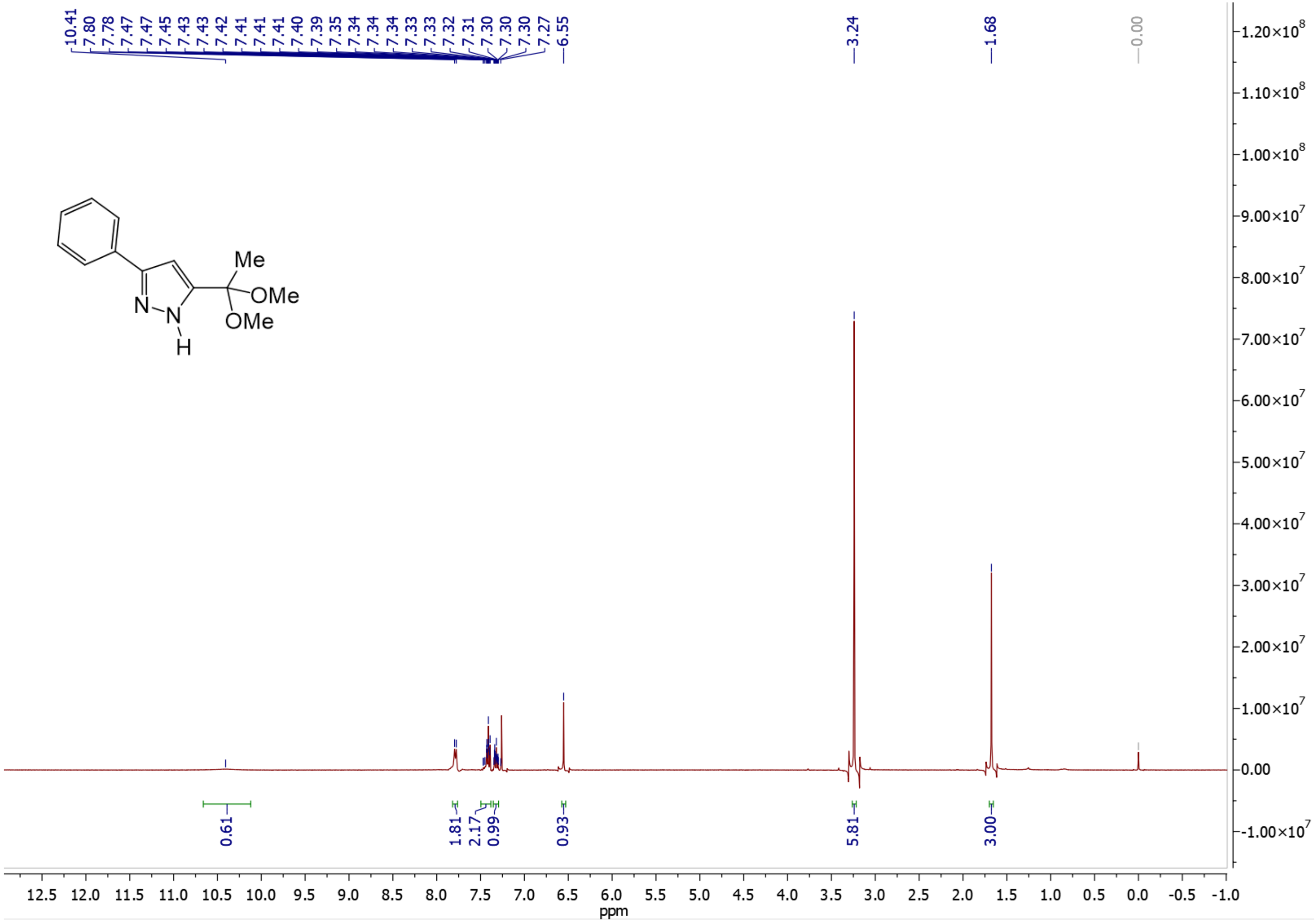

Figure S76. ^{13}C NMR spectrum of compound **18b**.

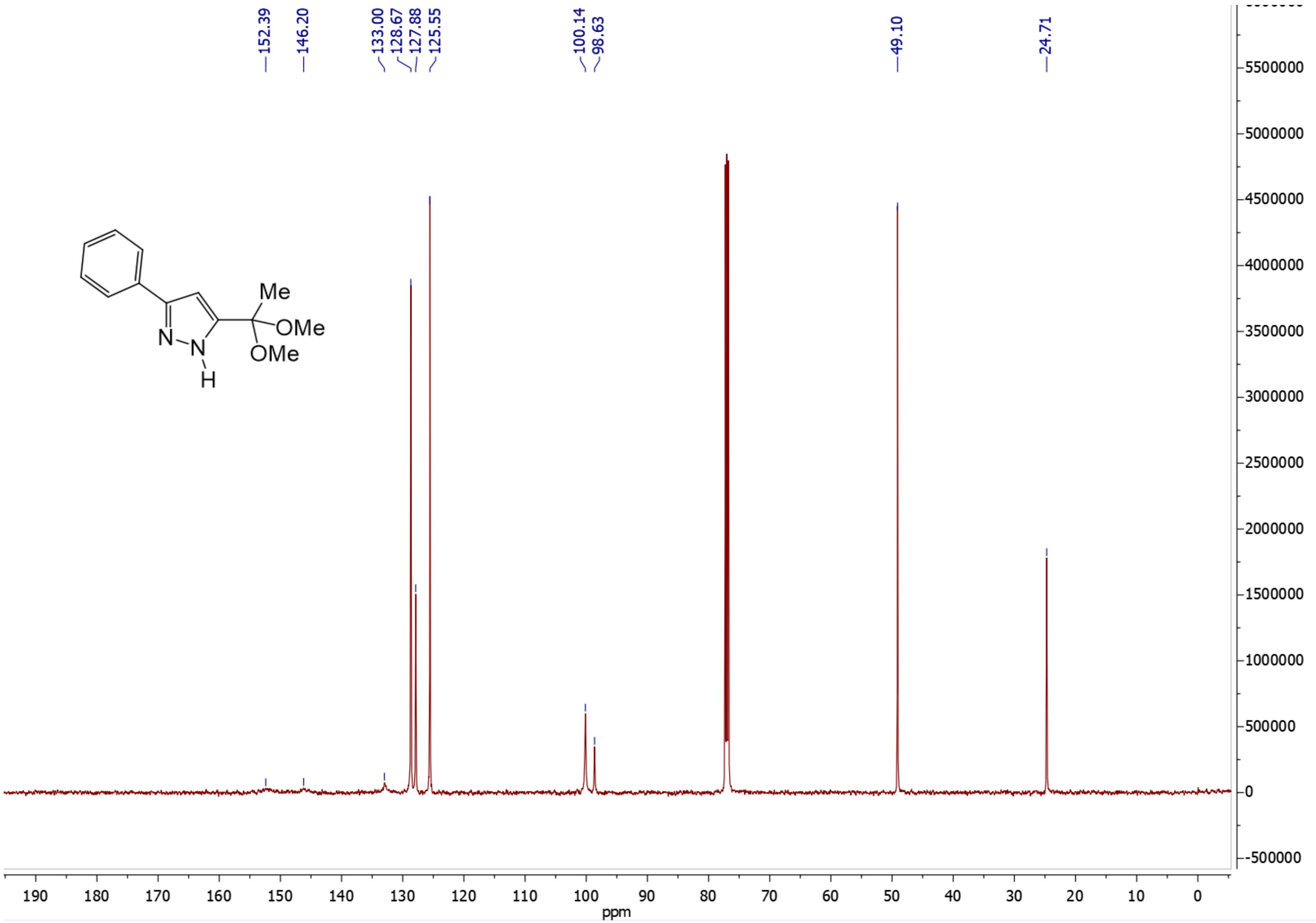

Figure S77. ^{1}H NMR spectrum of compound **18c**.

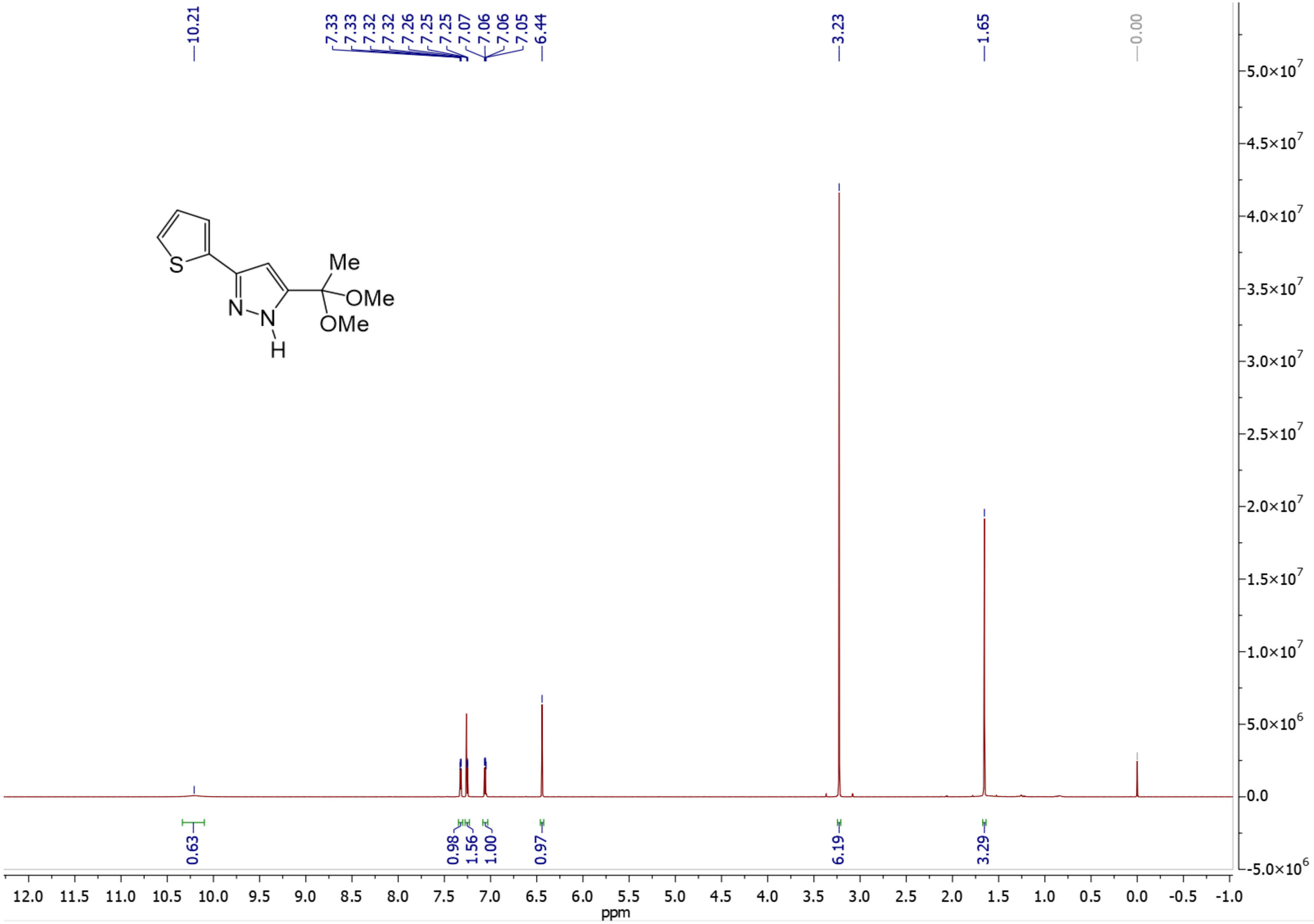

Figure S78. ^{13}C NMR spectrum of compound **18c**.

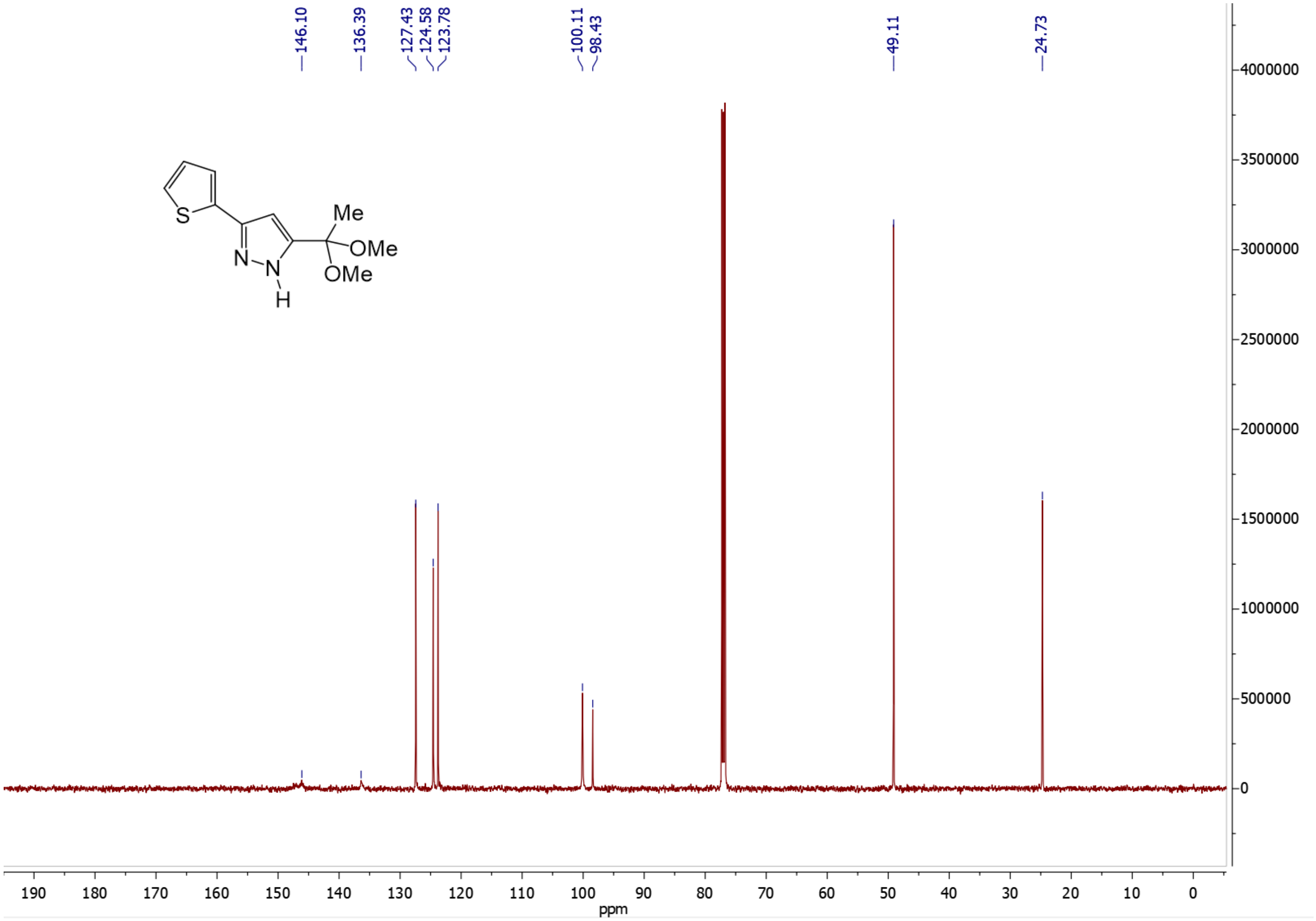

Figure S79. ^{1}H NMR spectrum of compound **19a**.

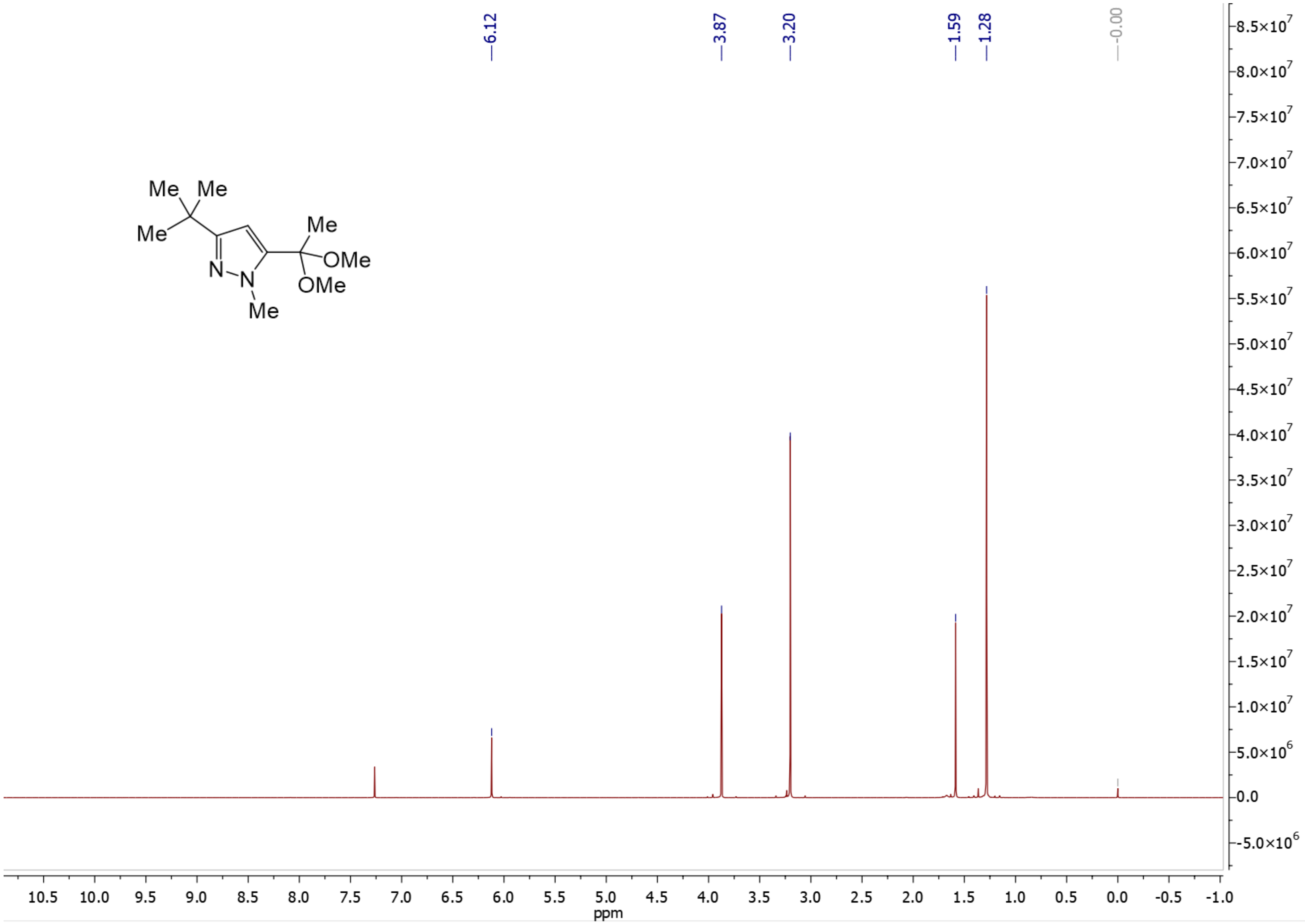

Figure S80. ^{13}C NMR spectrum of compound **19a**.

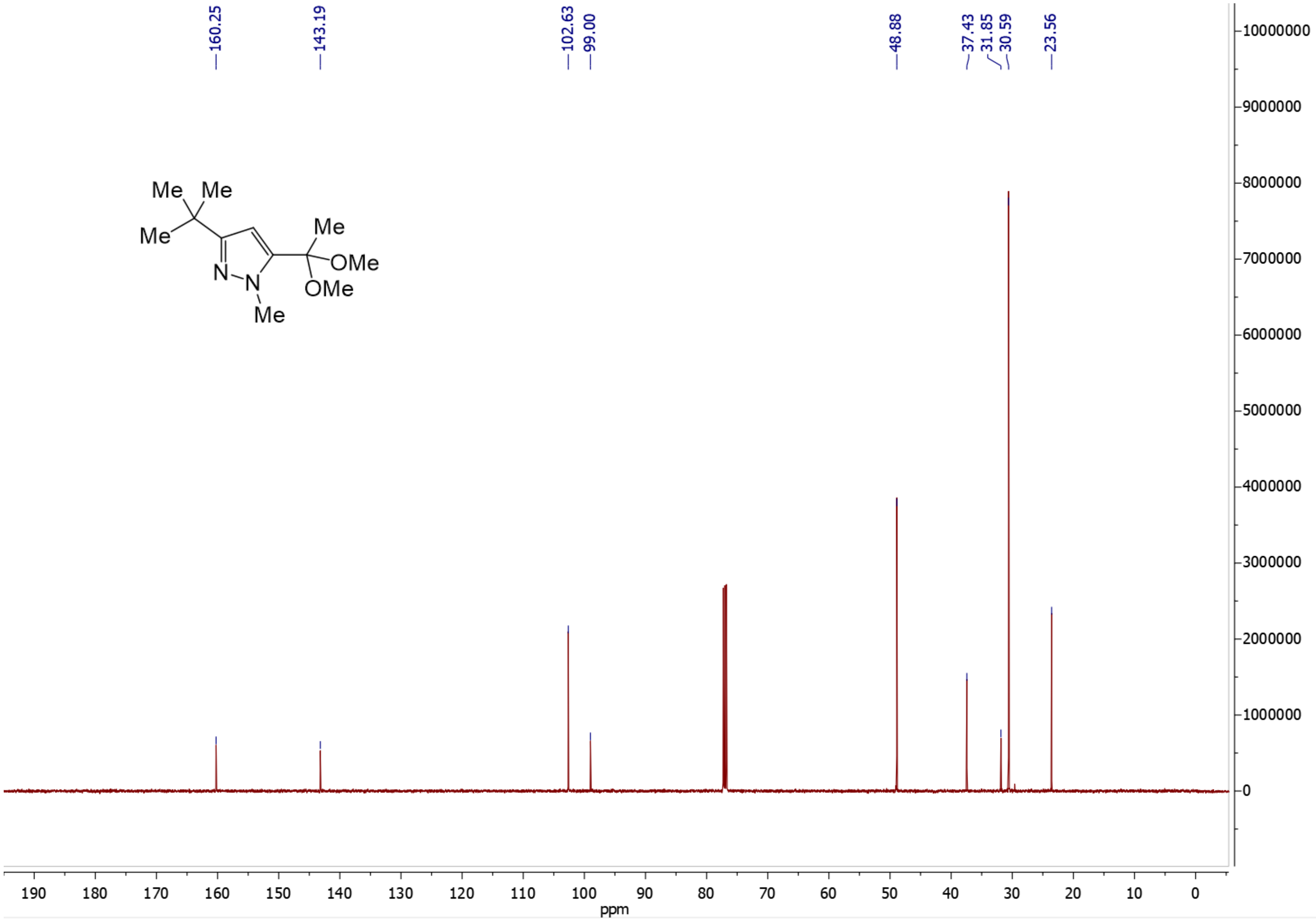

Figure S81. ^{1}H NMR spectrum of compound **19b**.

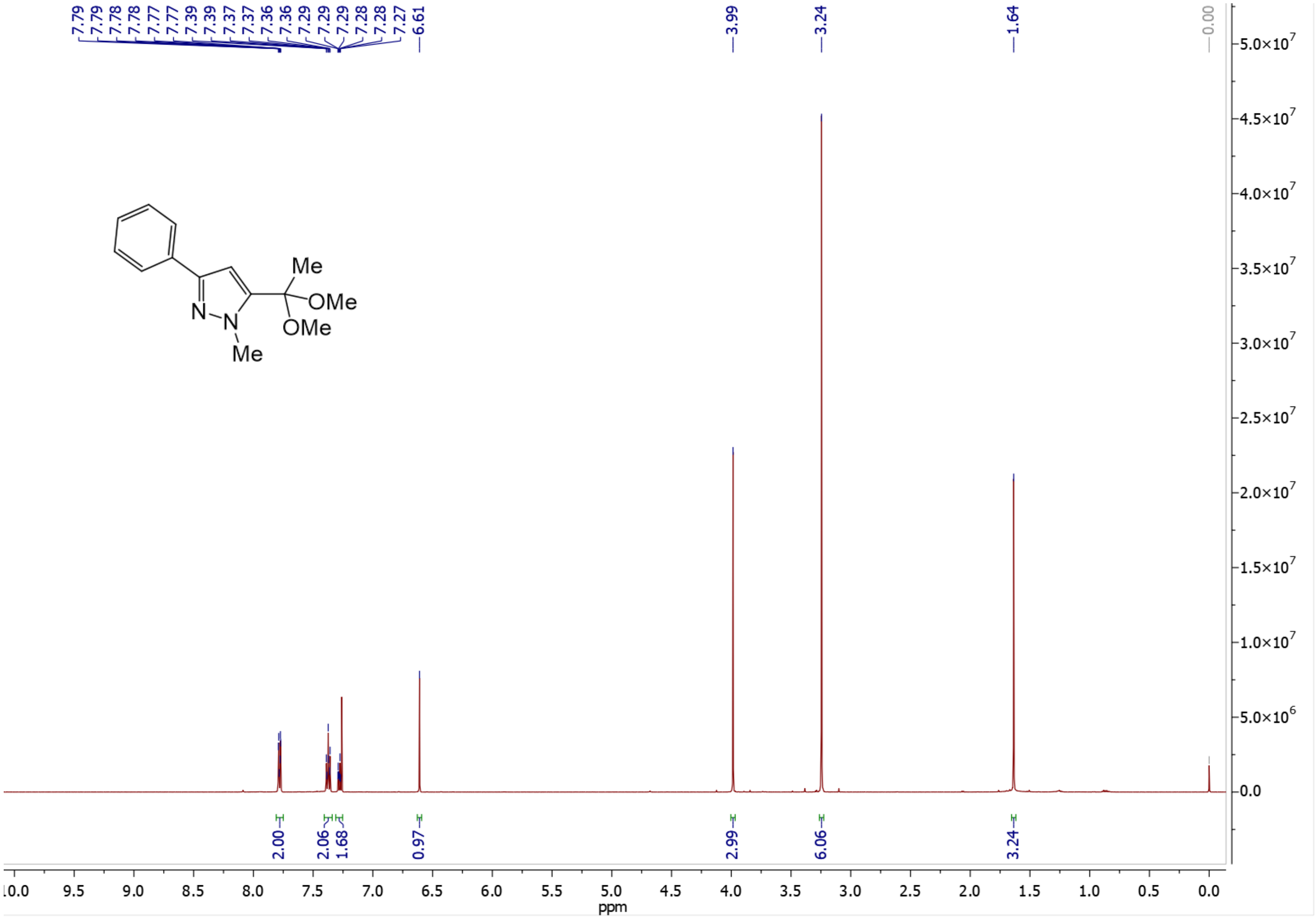

Figure S82. ^{13}C NMR spectrum of compound **19b**.

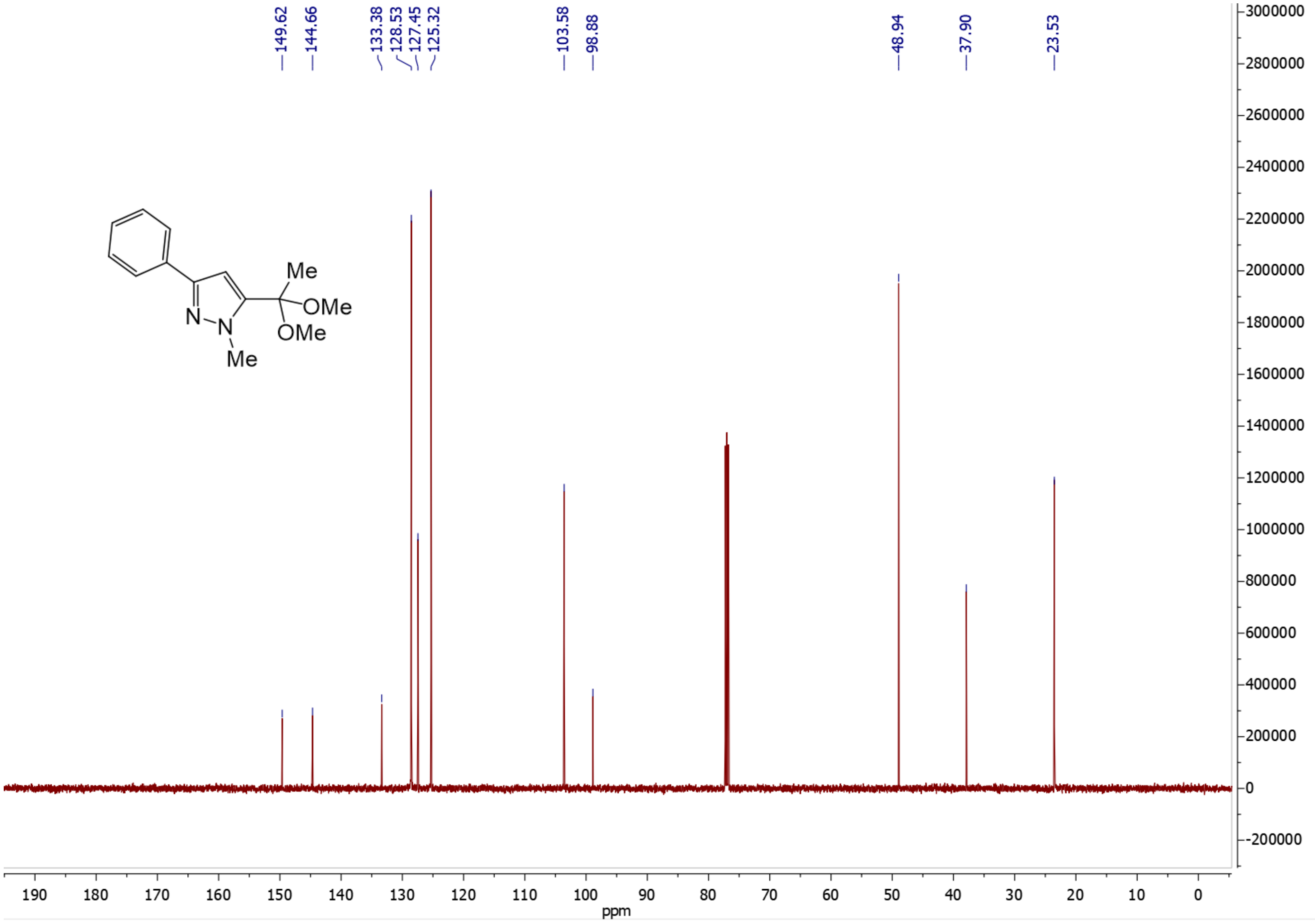

Figure S83. ^{1}H NMR spectrum of compound **19c**.

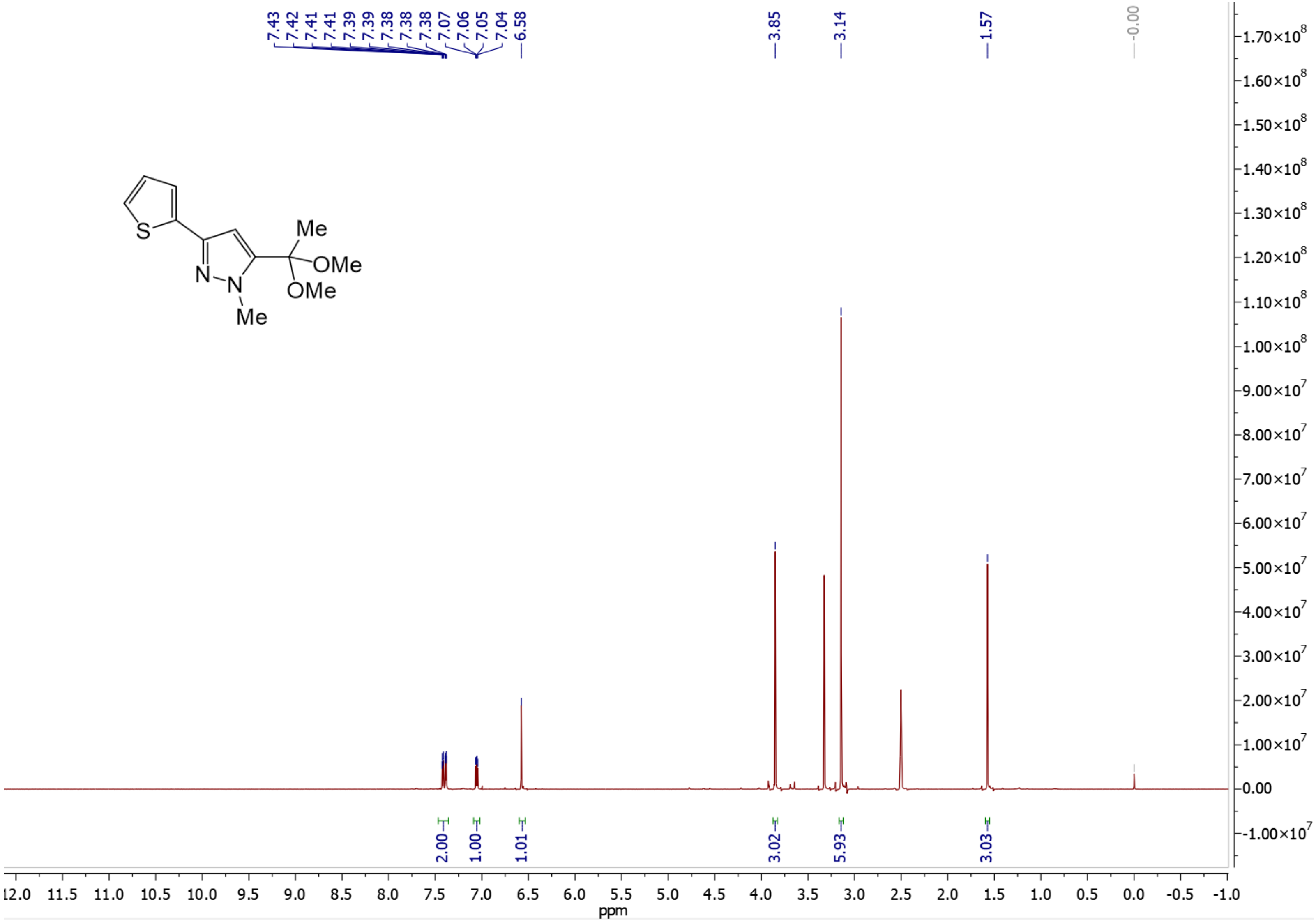

Figure S84. ^{13}C NMR spectrum of compound **19c**.

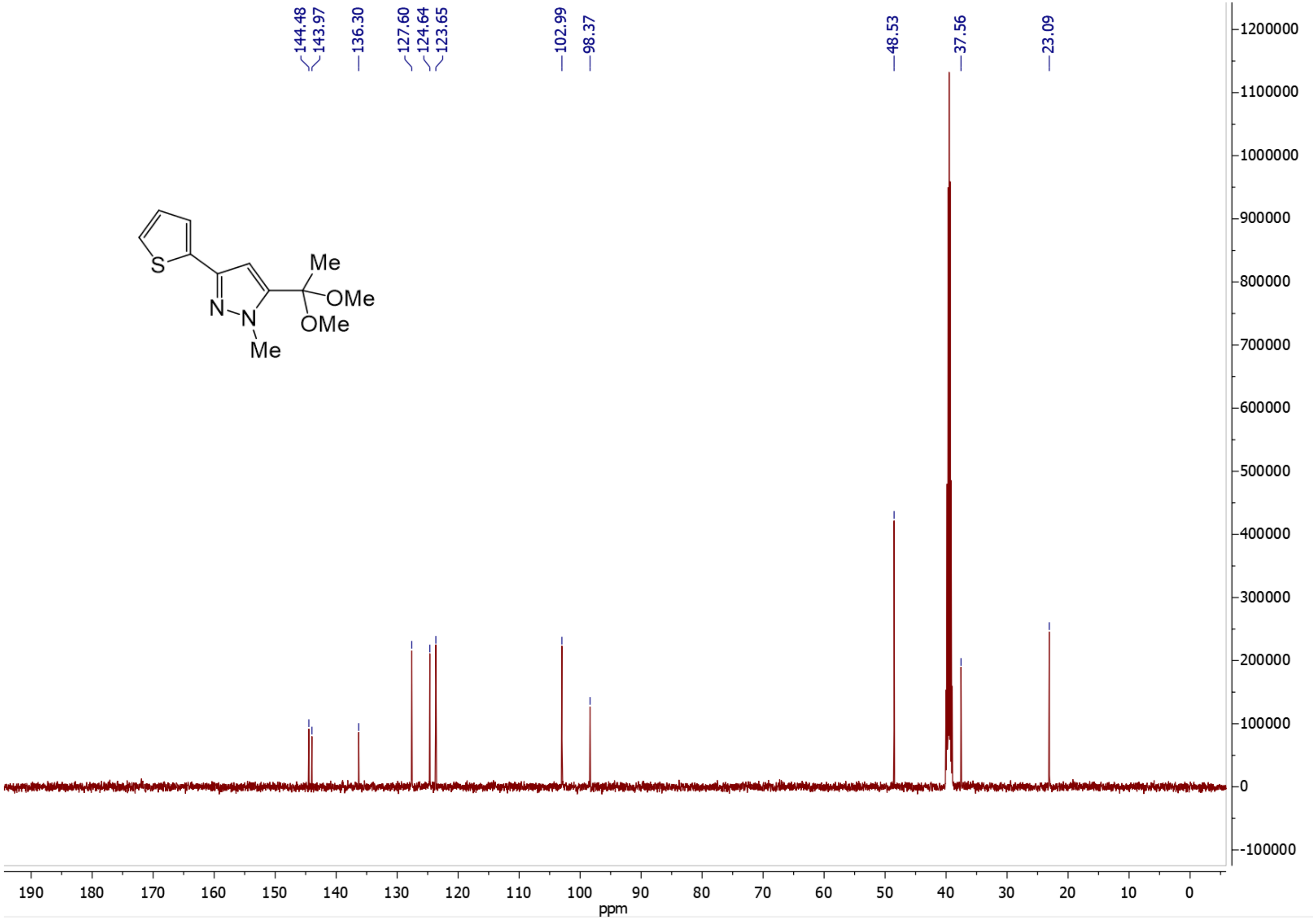

Figure S85. ^{1}H NMR spectrum of compound **20a**.

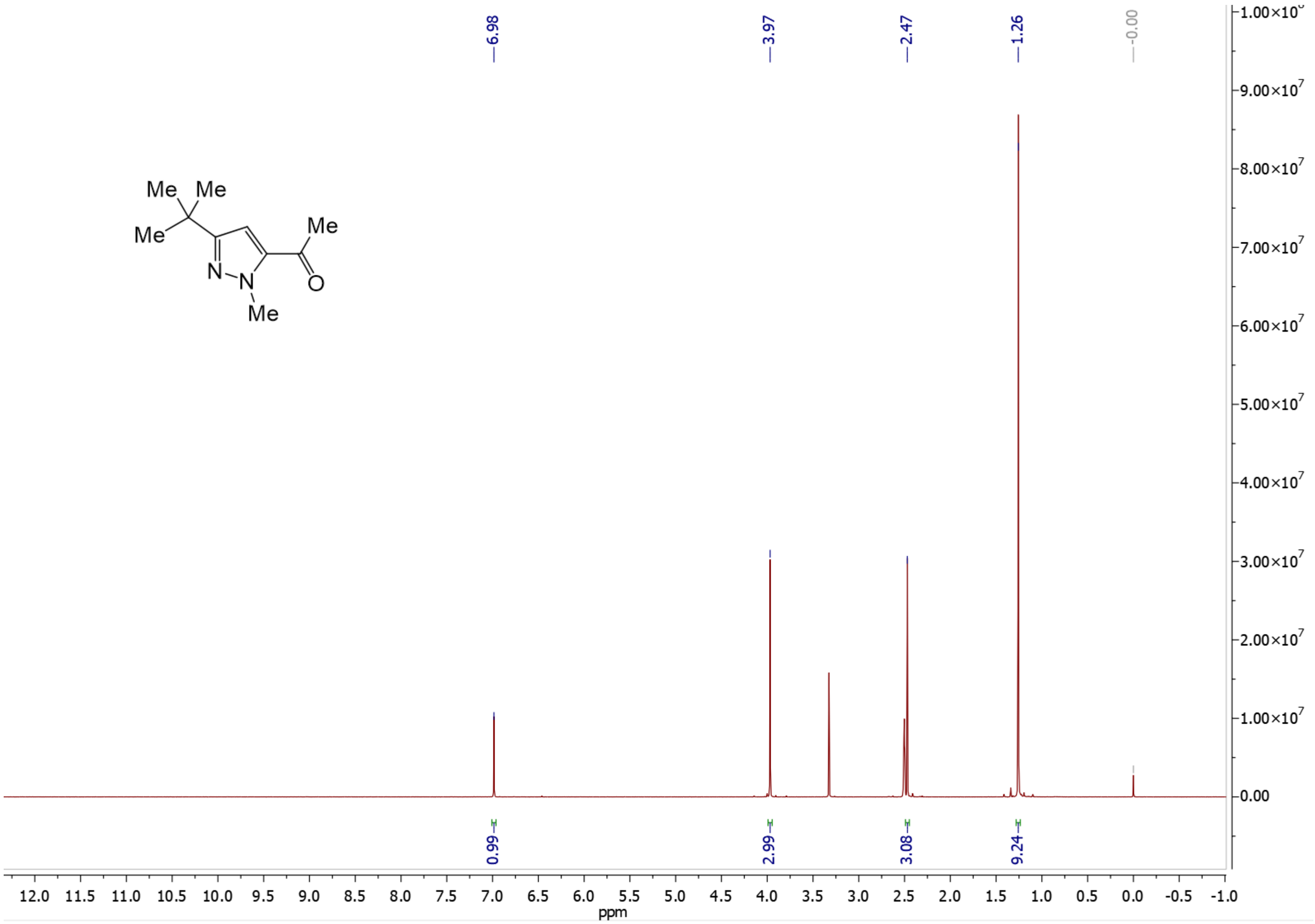

Figure S86 ^{13}C NMR spectrum of compound **20a**.

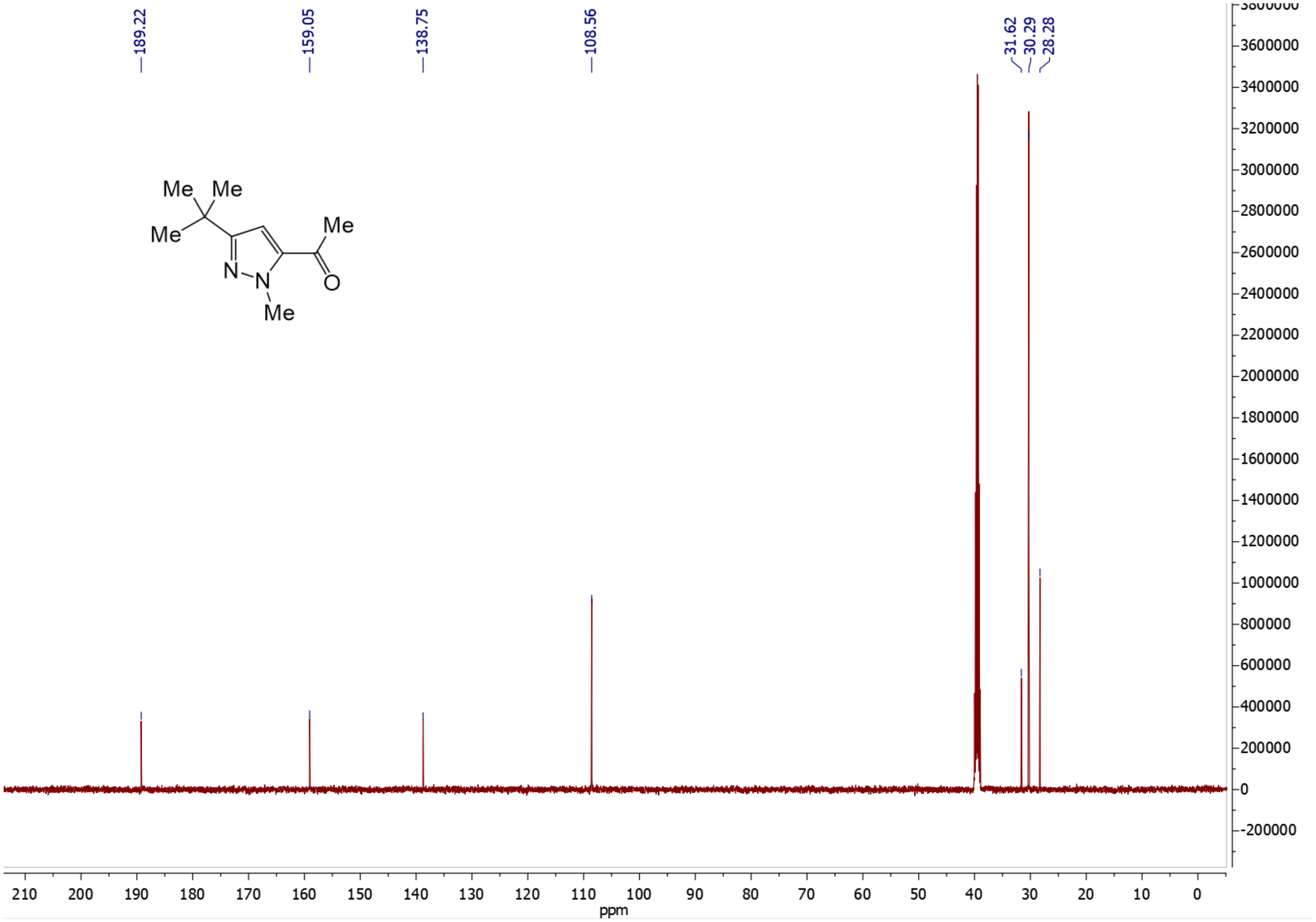

Figure S87. ^{1}H NMR spectrum of compound **20b**.

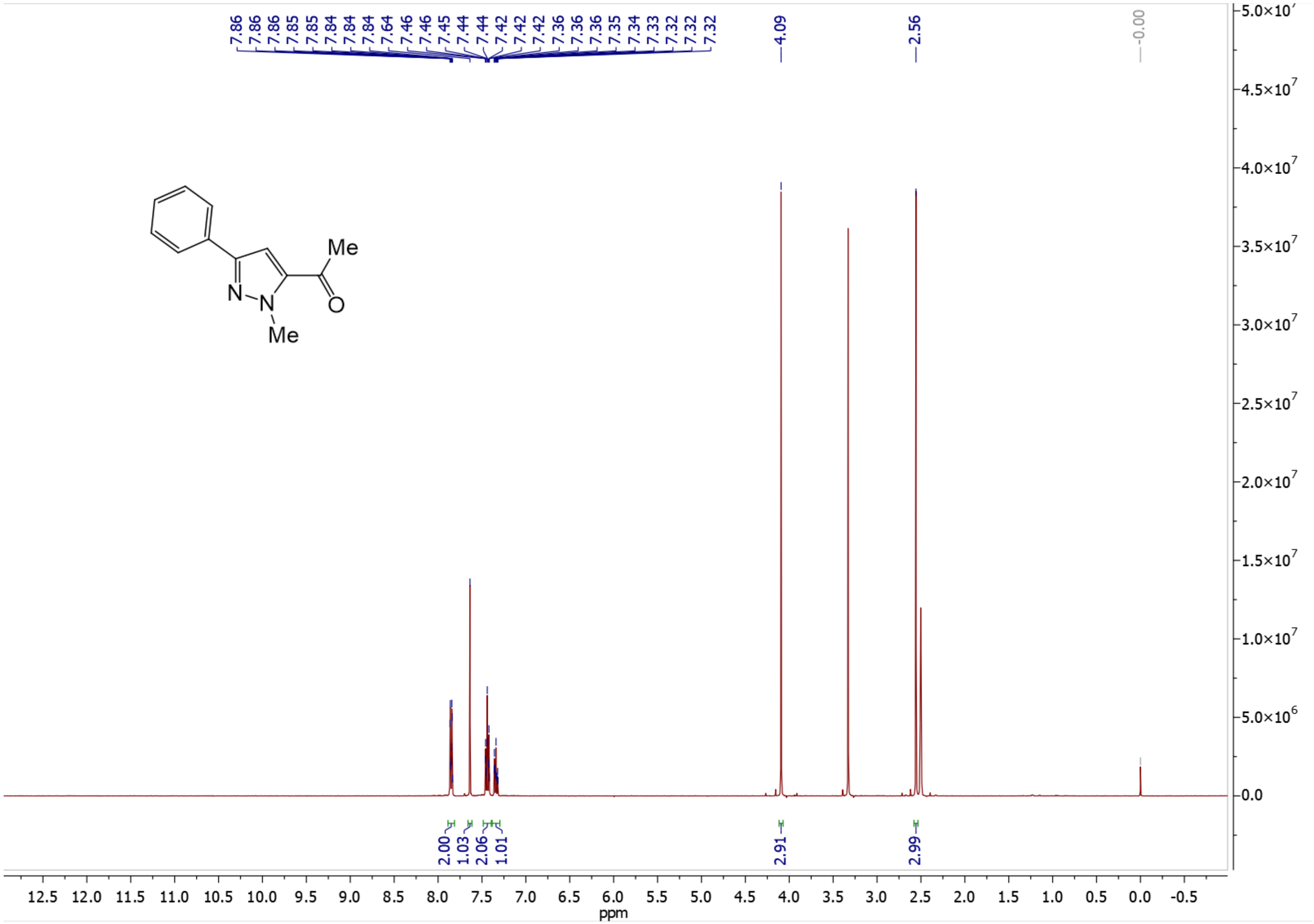

Figure S88. ^{13}C NMR spectrum of compound **20b**.

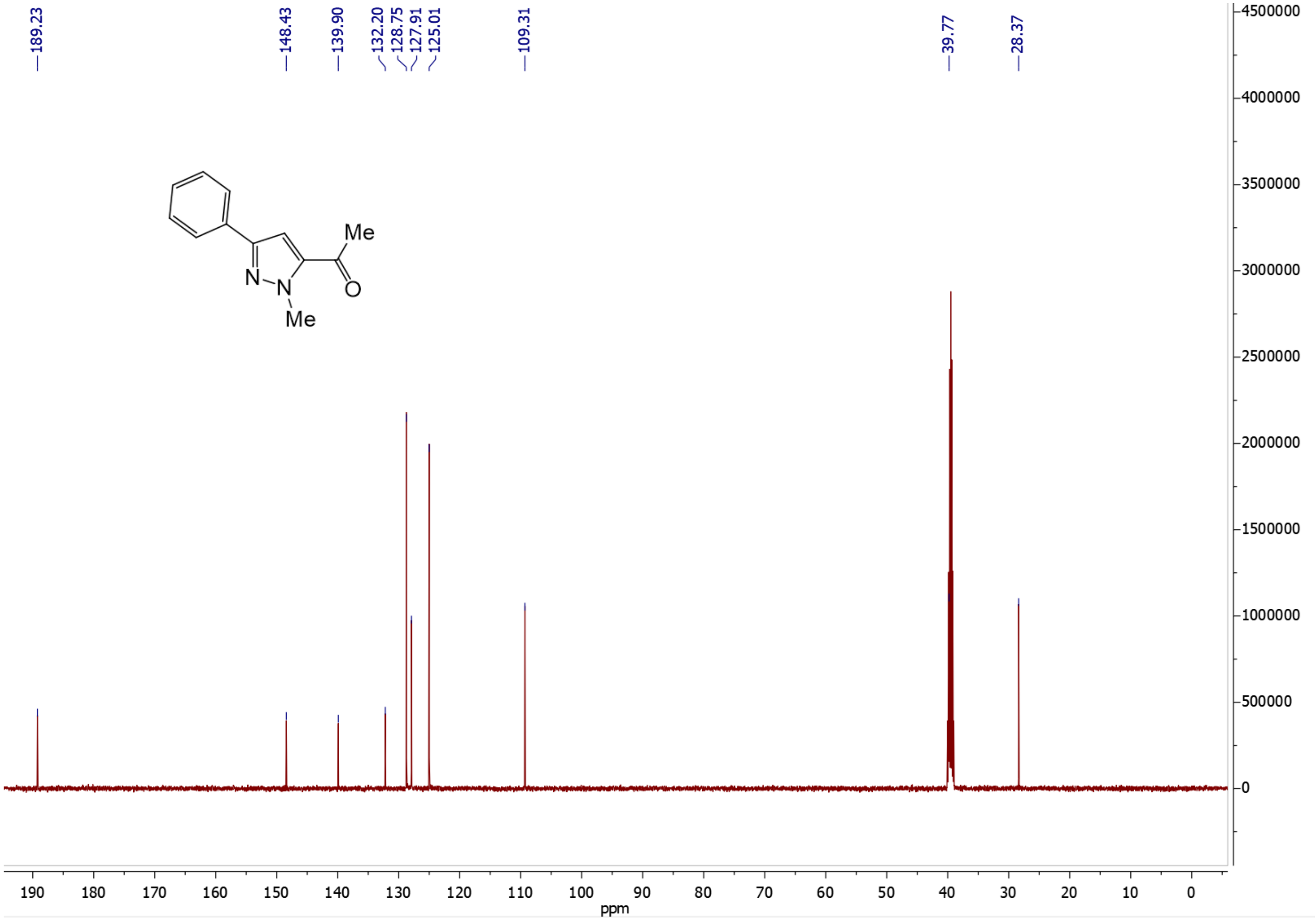

Figure S89. ^{1}H NMR spectrum of compound **20c**.

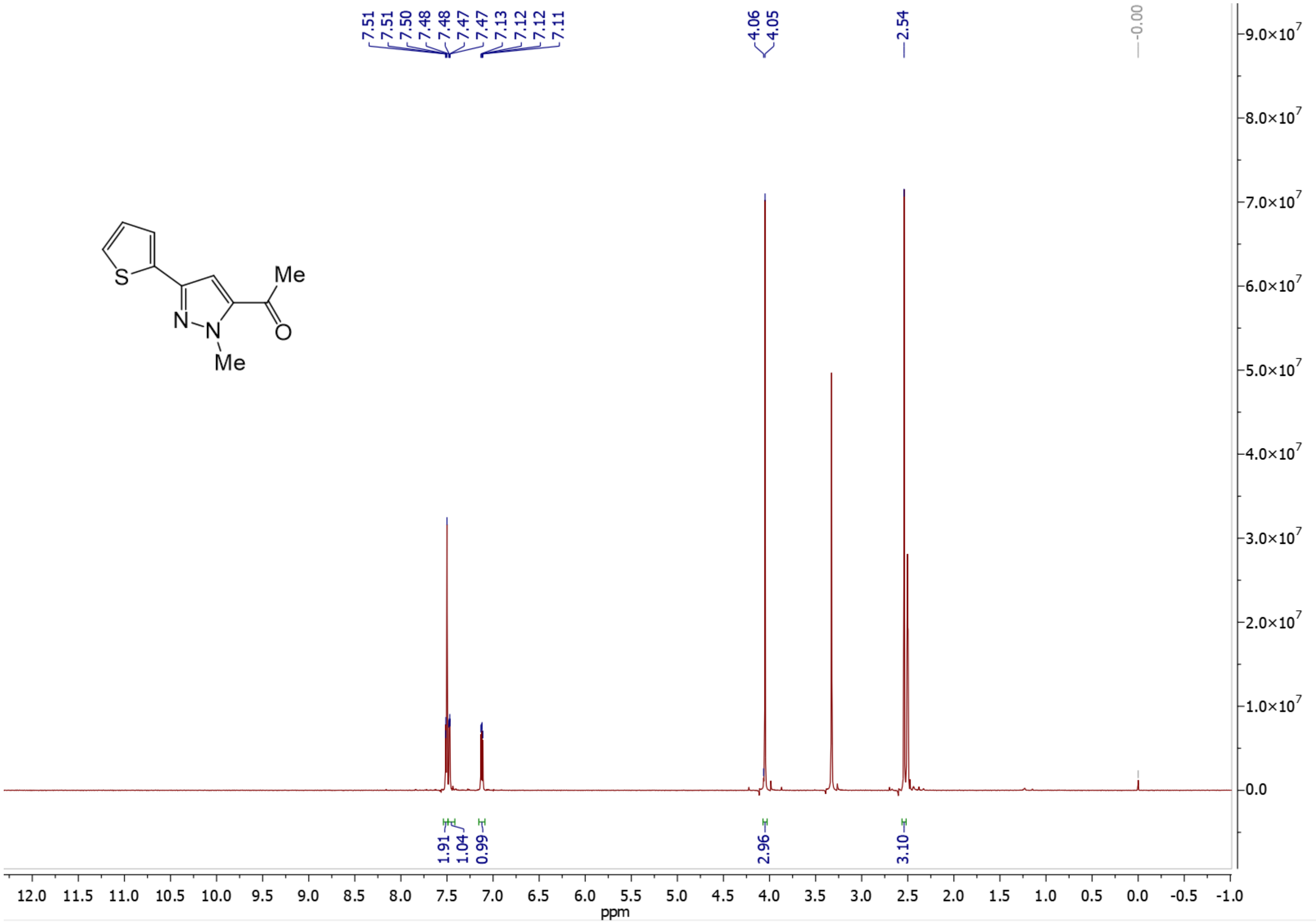

Figure S90. ^{13}C NMR spectrum of compound **20c**.

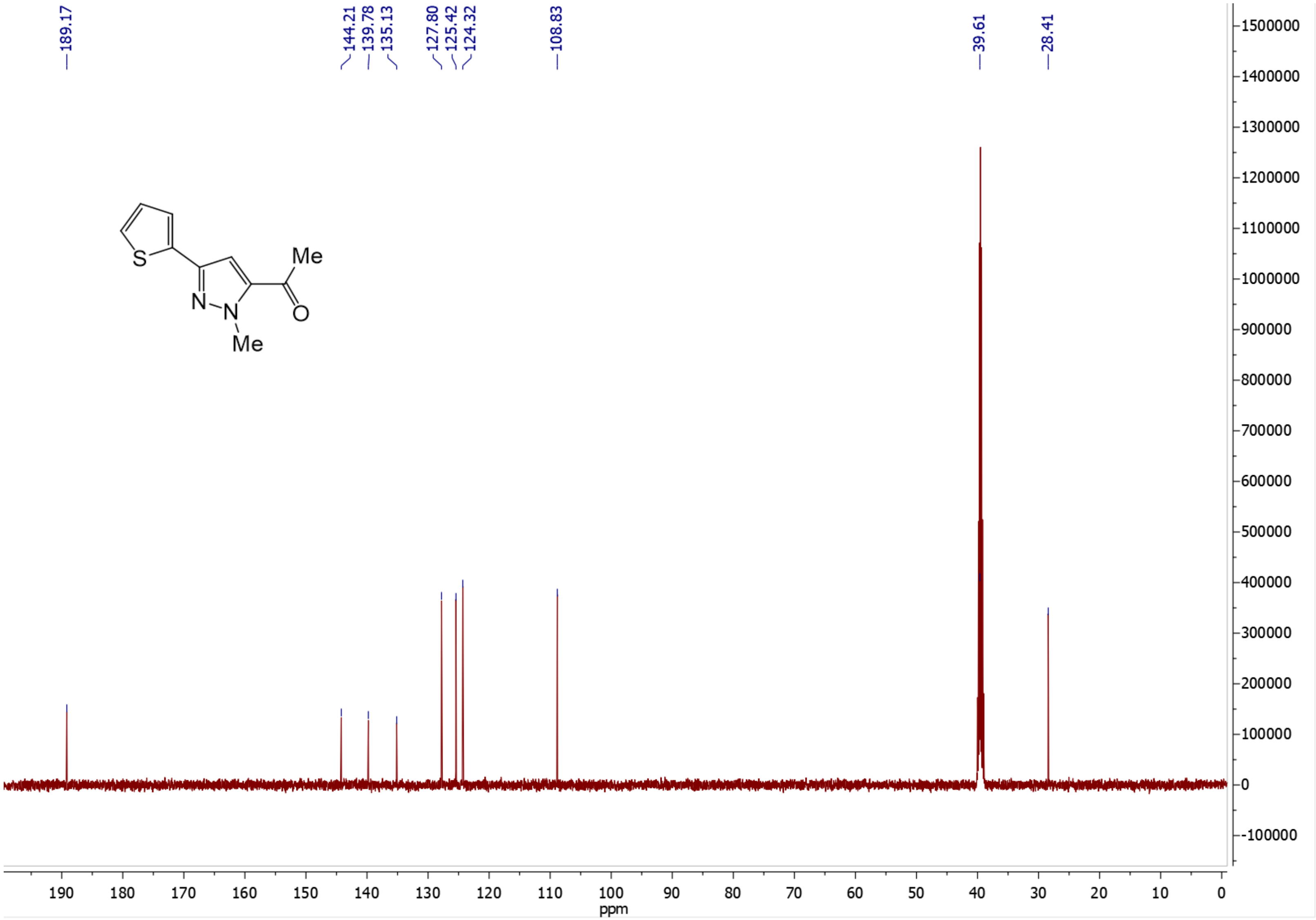

Figure S91. ^{1}H NMR spectrum of compound **21a**.

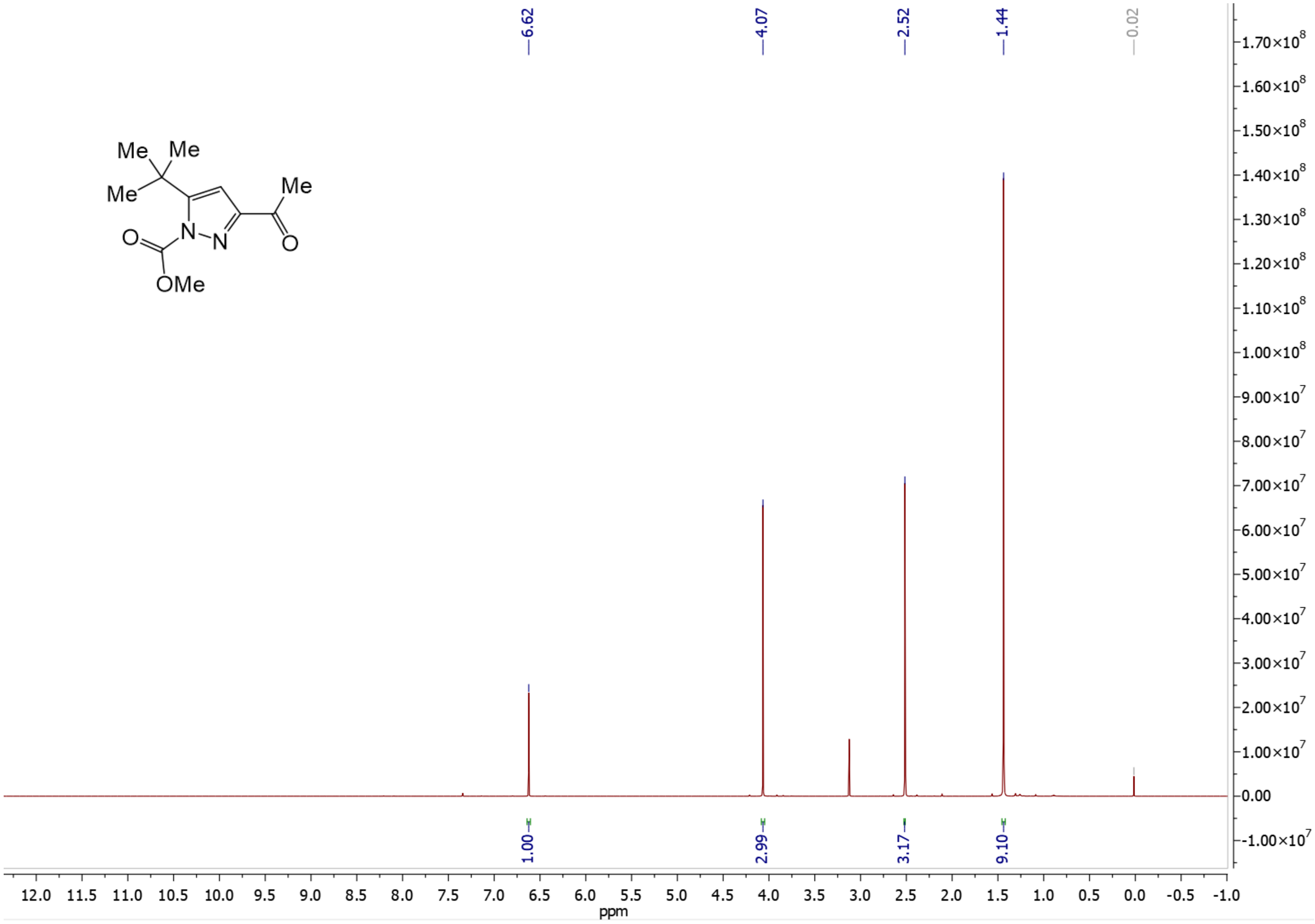

Figure S92. ^{13}C NMR spectrum of compound **21a**.

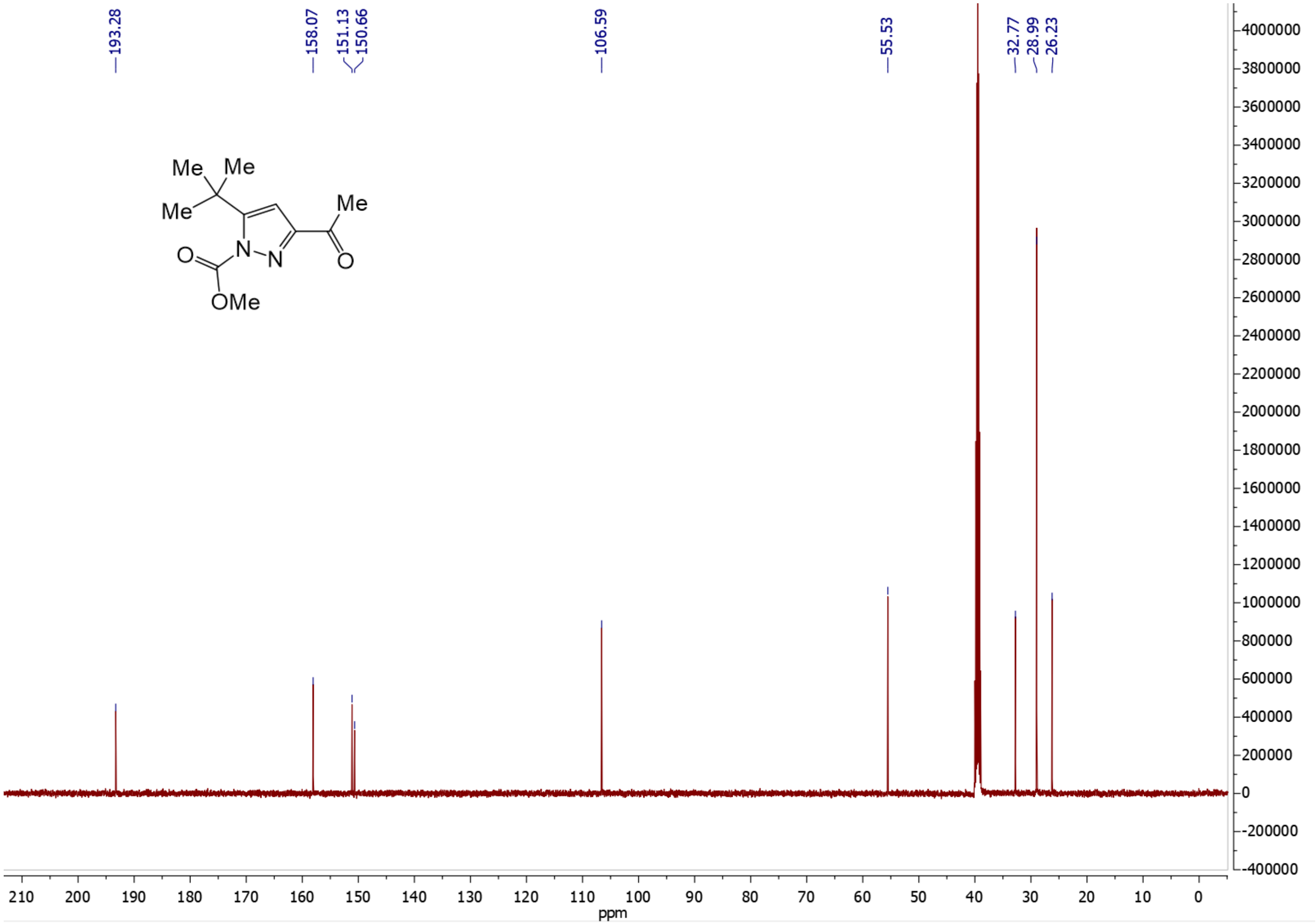

Figure S93. ^{1}H NMR spectrum of compound **21b**.

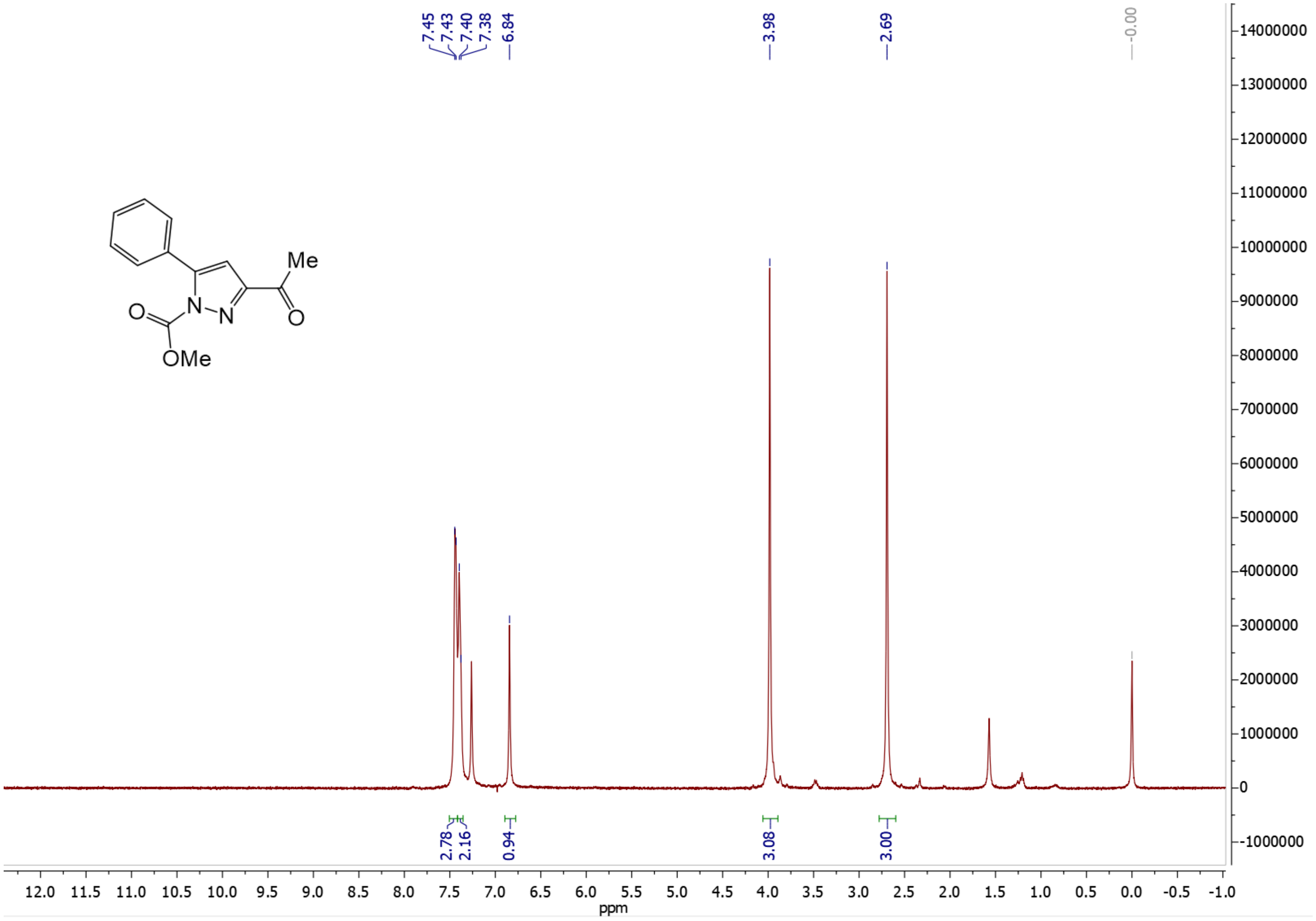

Figure S94. ^{13}C NMR spectrum of compound **21b**.

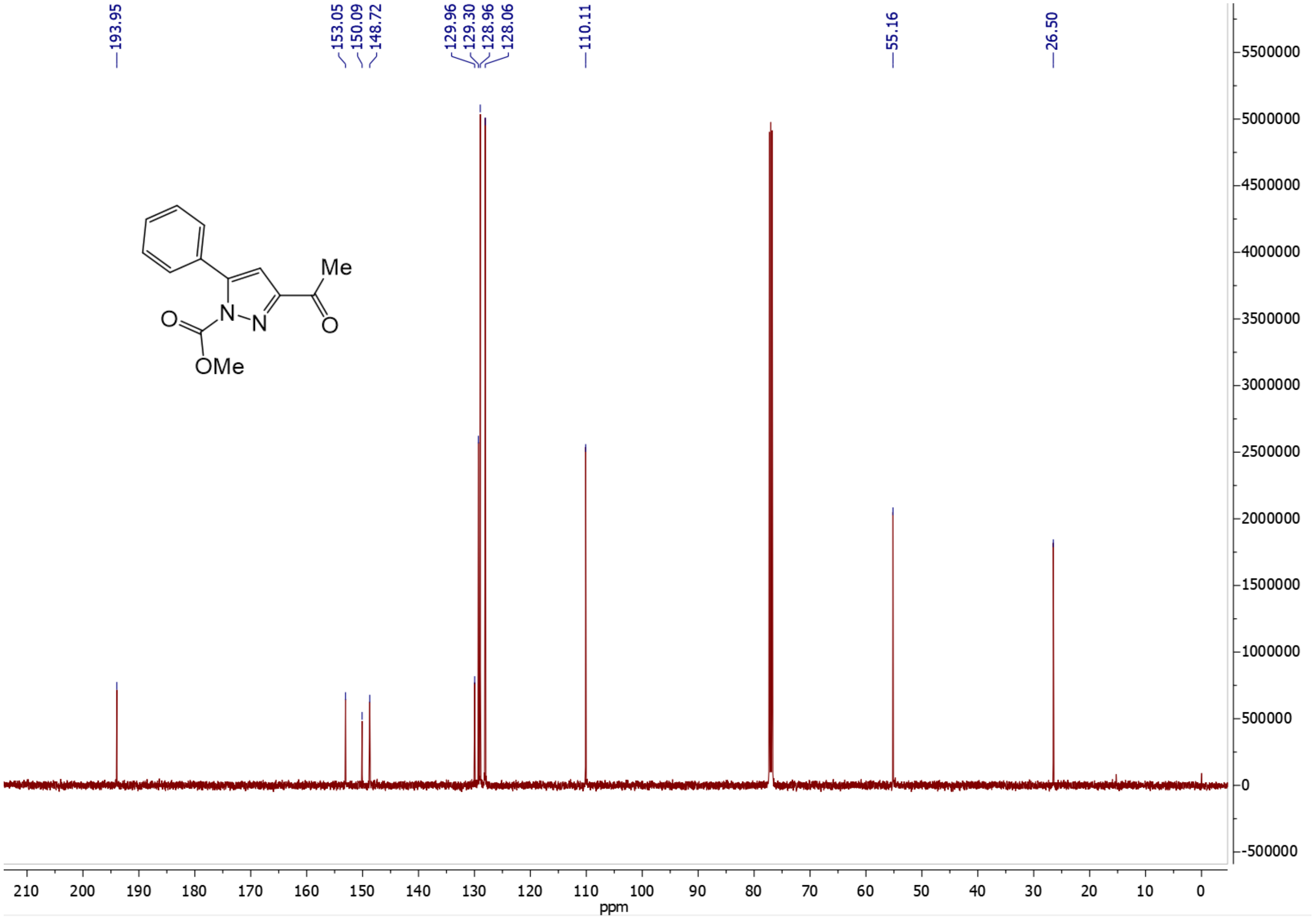

Figure S95. ^{1}H NMR spectrum of compound **21c**.

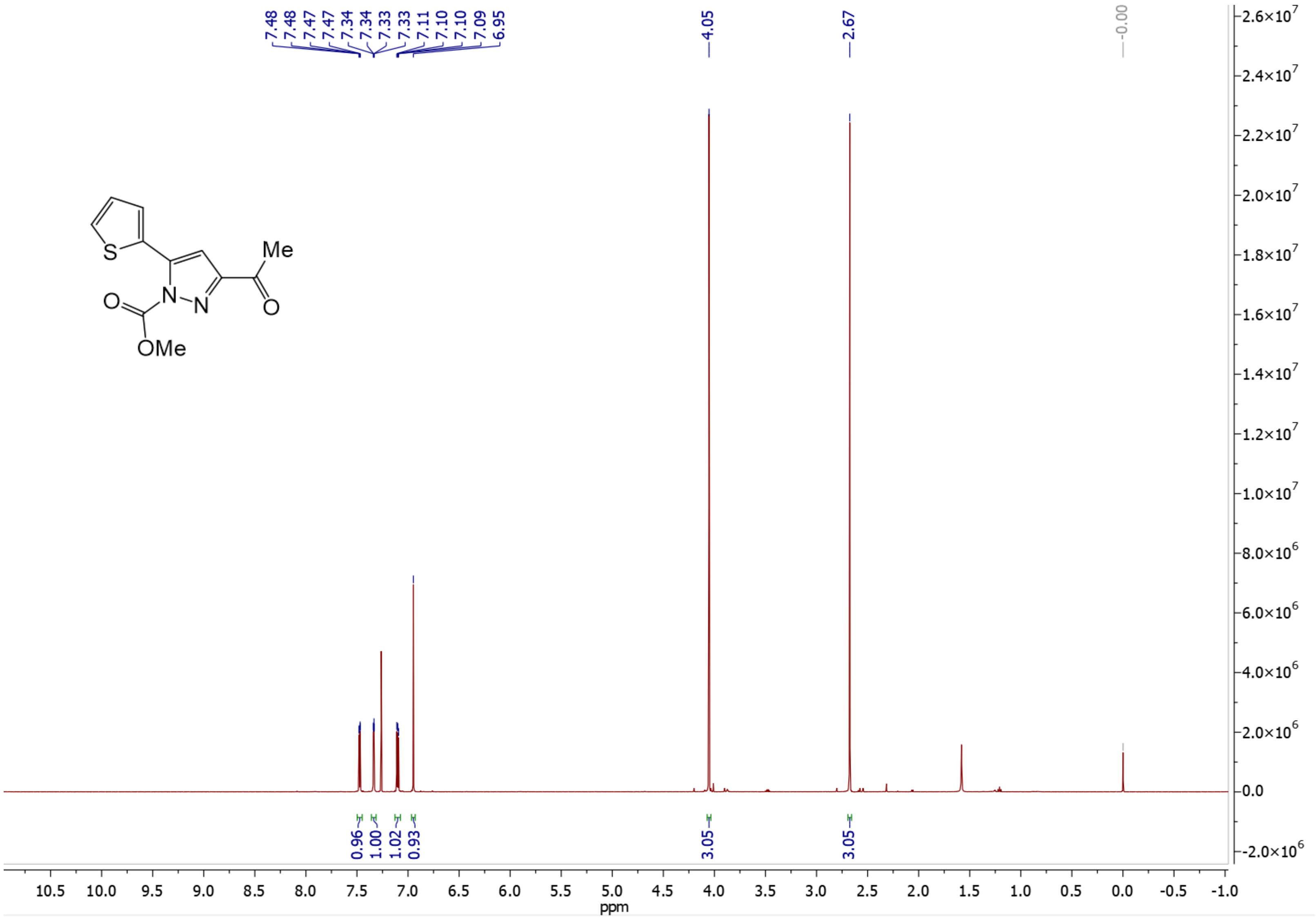

Figure S96. ^{13}C NMR spectrum of compound **21c**.

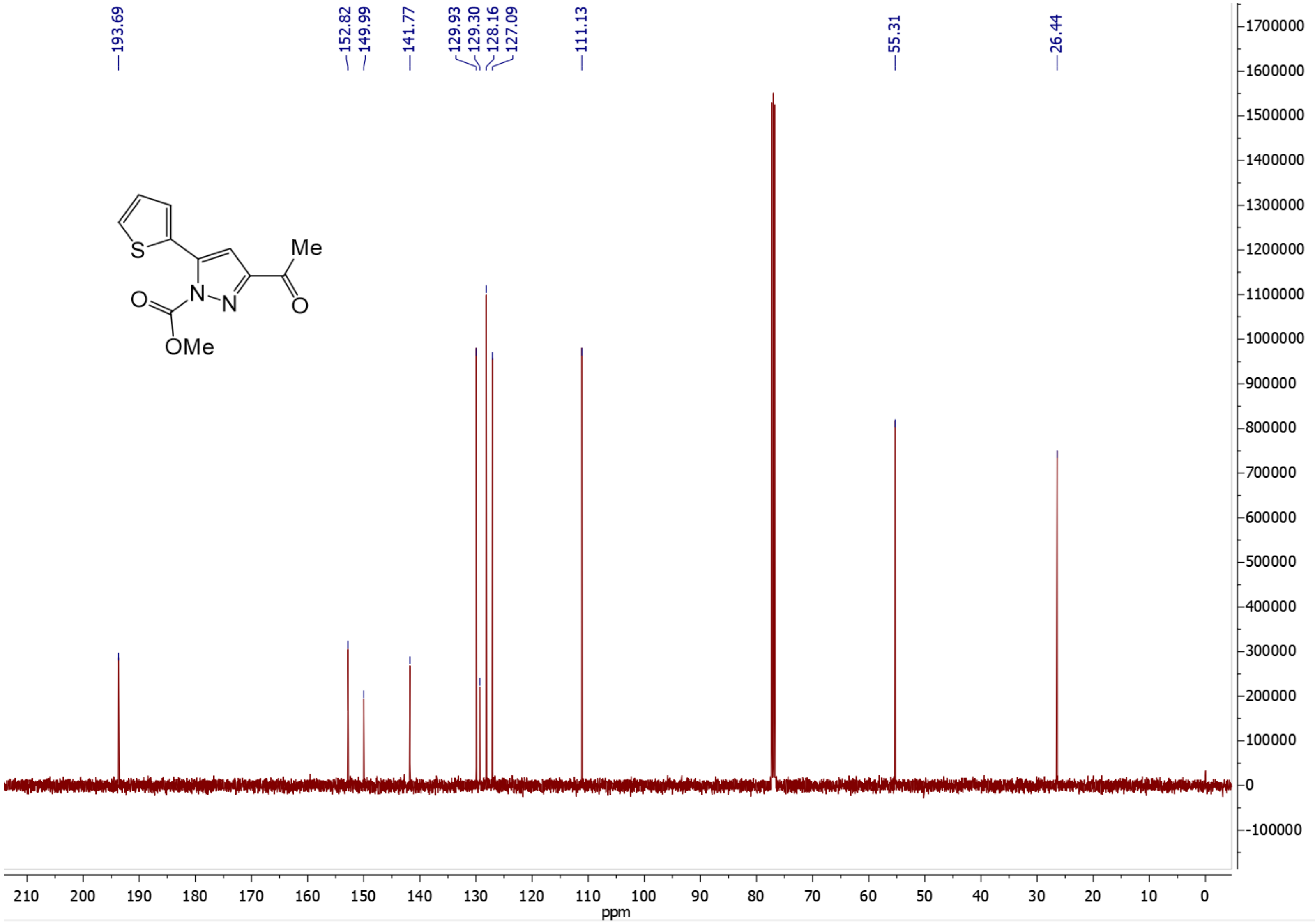

Figure S97. ^{1}H NMR spectrum of compound **22.**

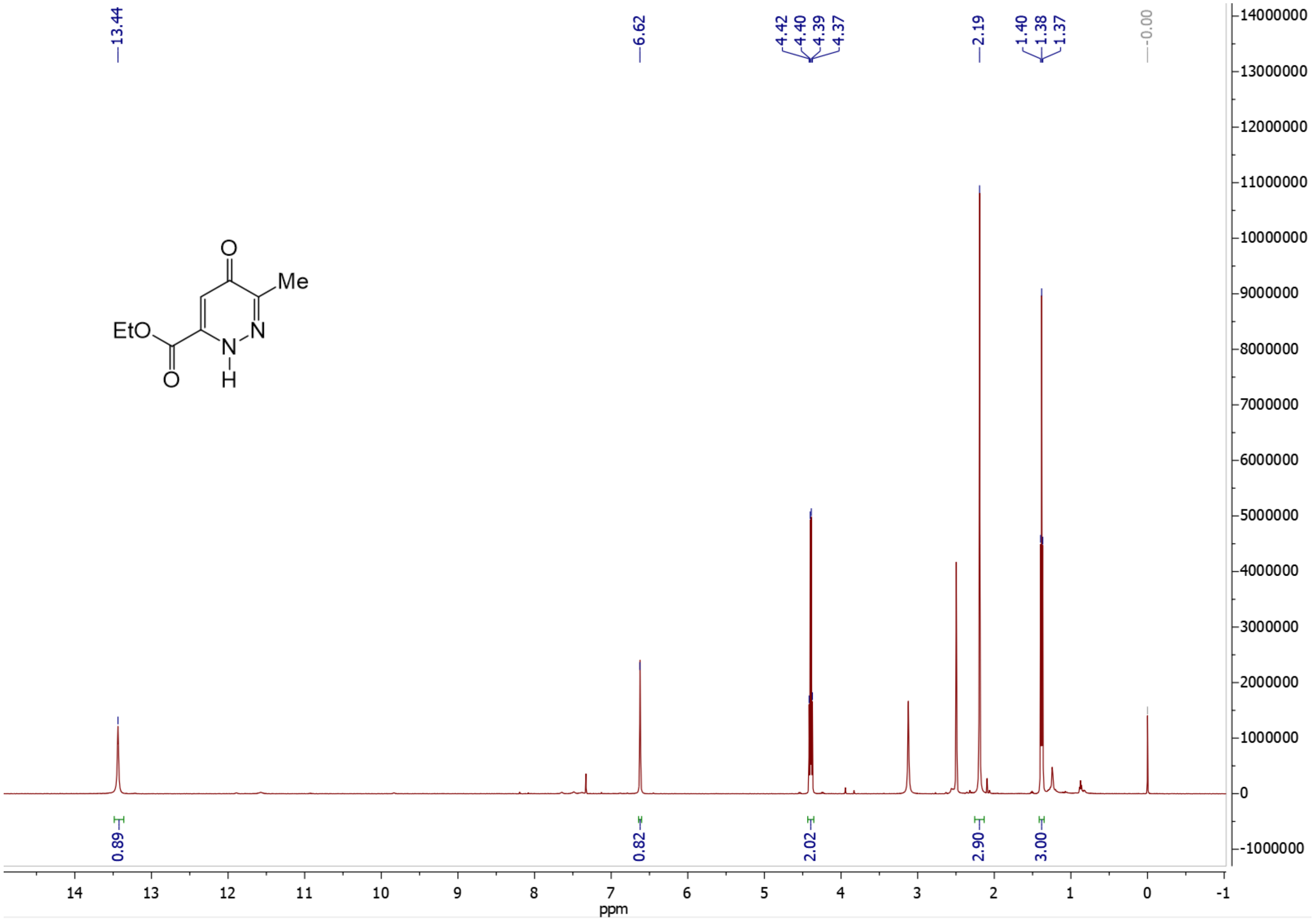

Figure S98. ^{13}C NMR spectrum of compound **22**.

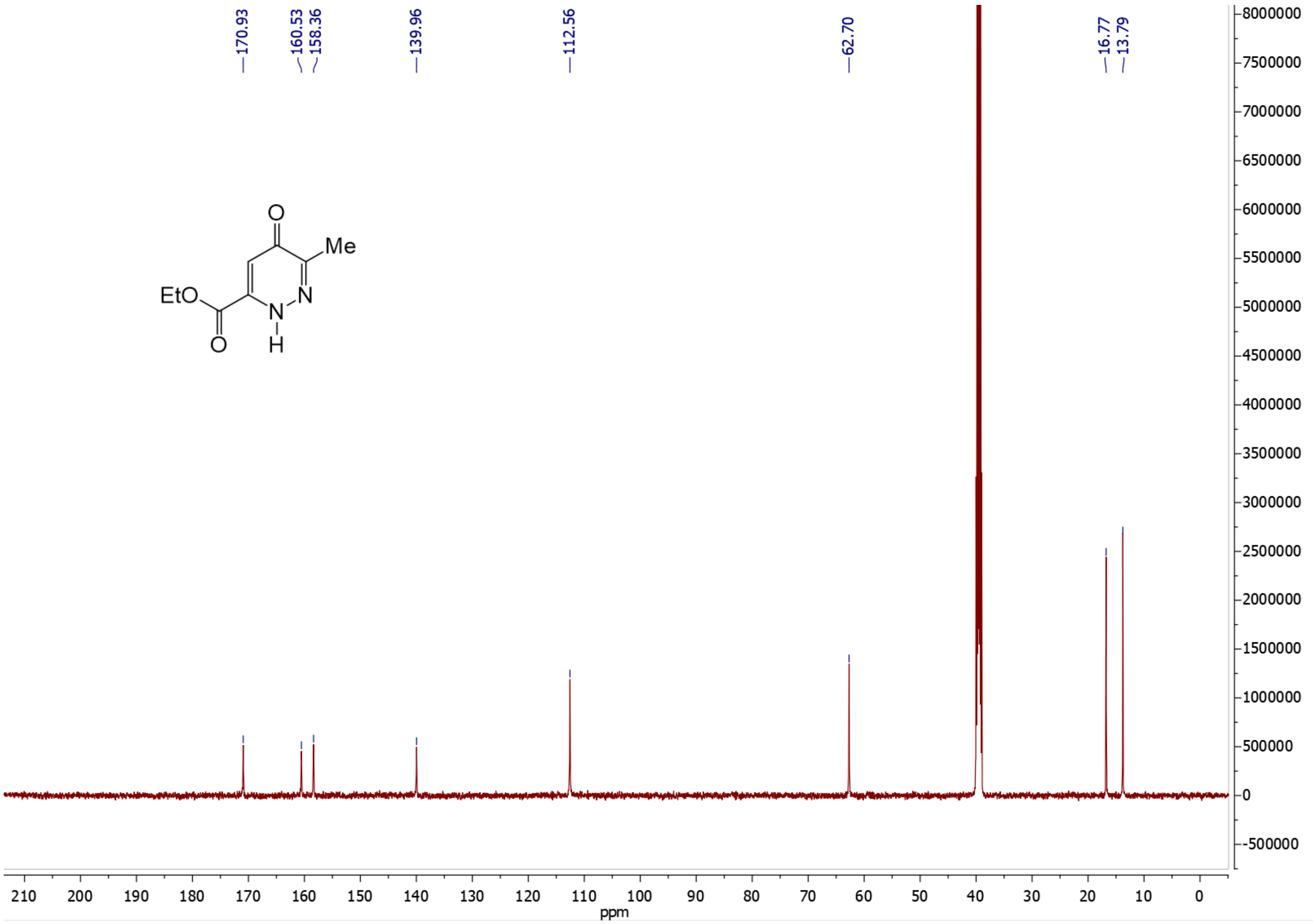

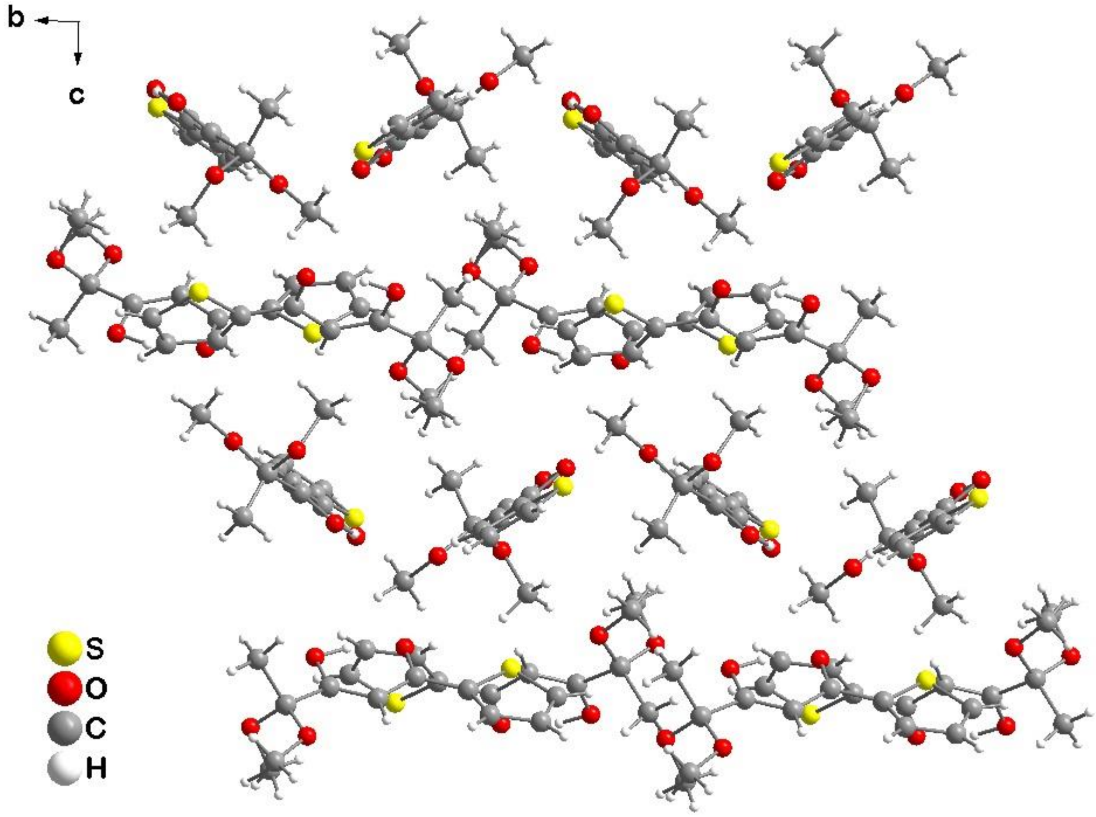

Figure S99. Crystal packing of diketone **2c** along *a* axis.

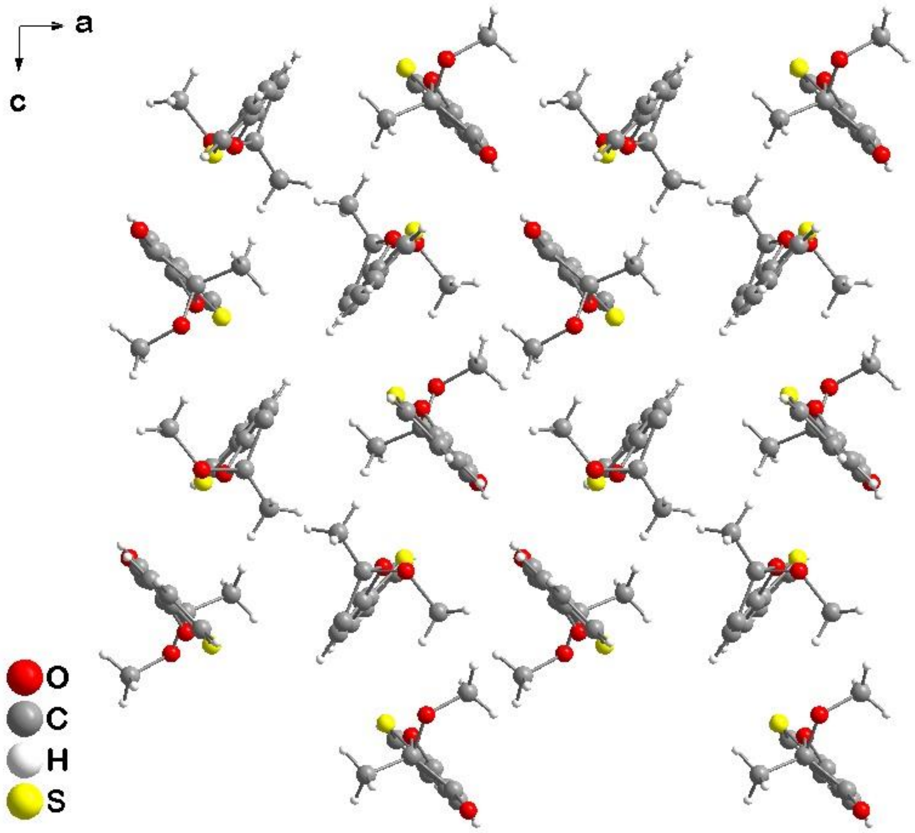

Figure S100. Crystal packing of furanone **3** along *b* axis.

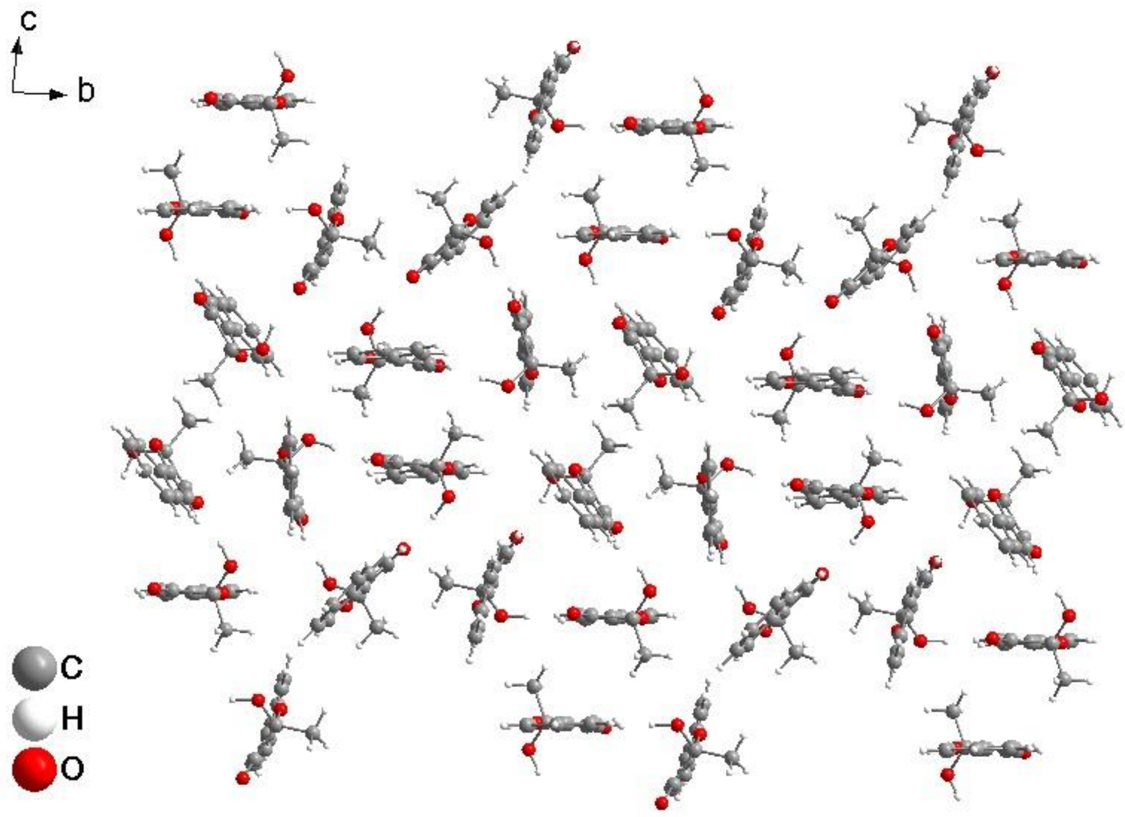

Figure S101. Crystal packing of furanone **5**.

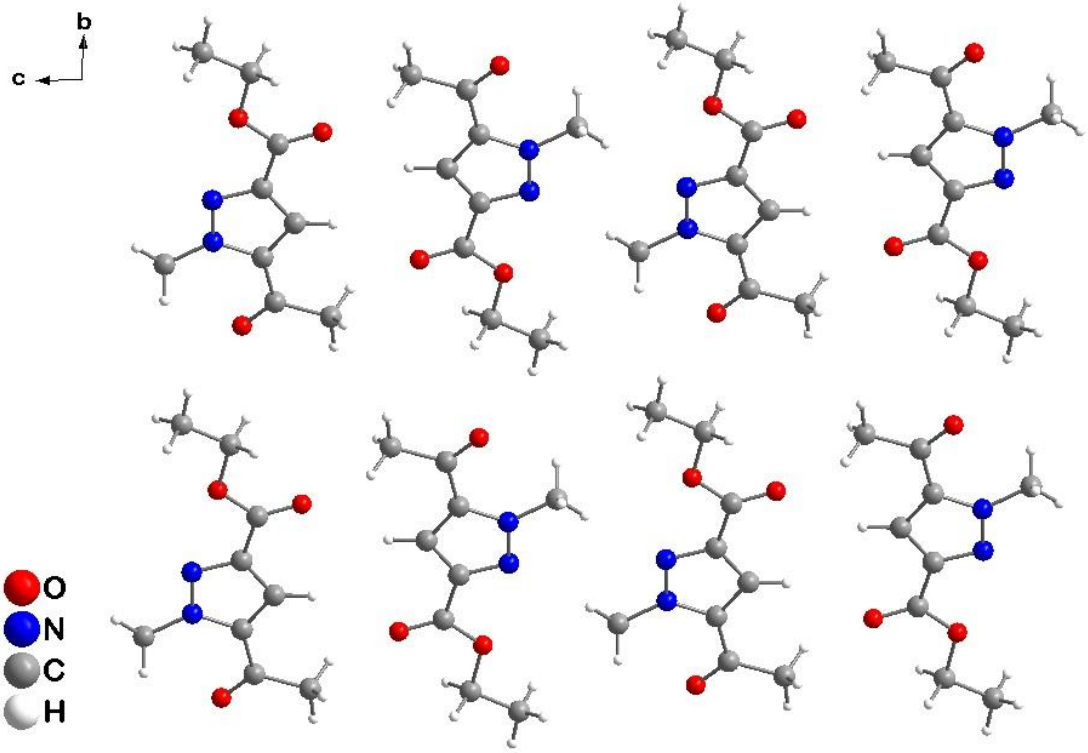

a

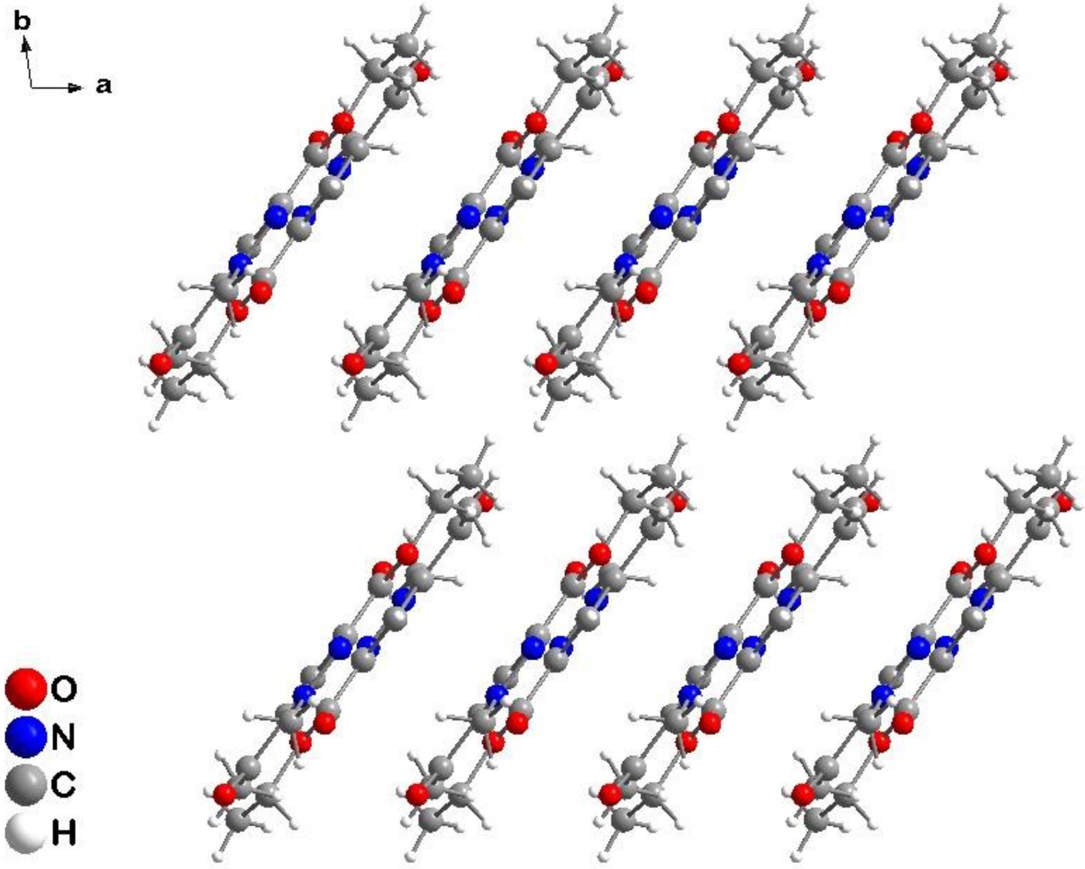

b

Figure S102. Crystal packing of pyrazole **7a** along *a* (**a**) and *c* (**b**) axis.

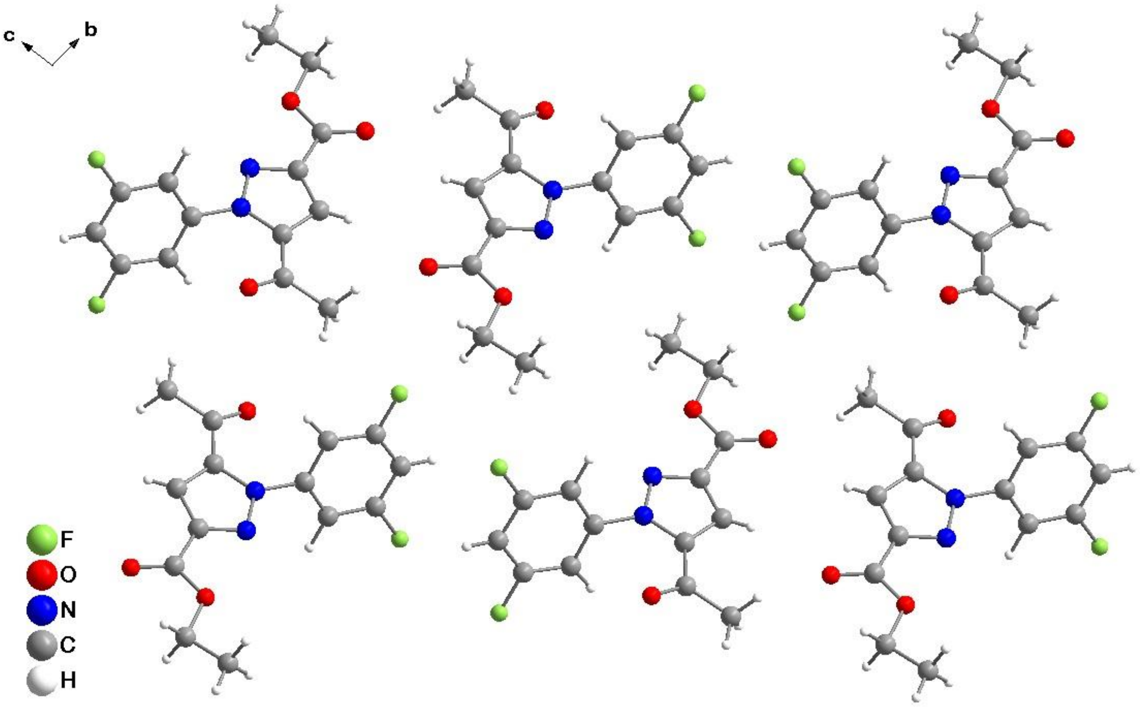

a

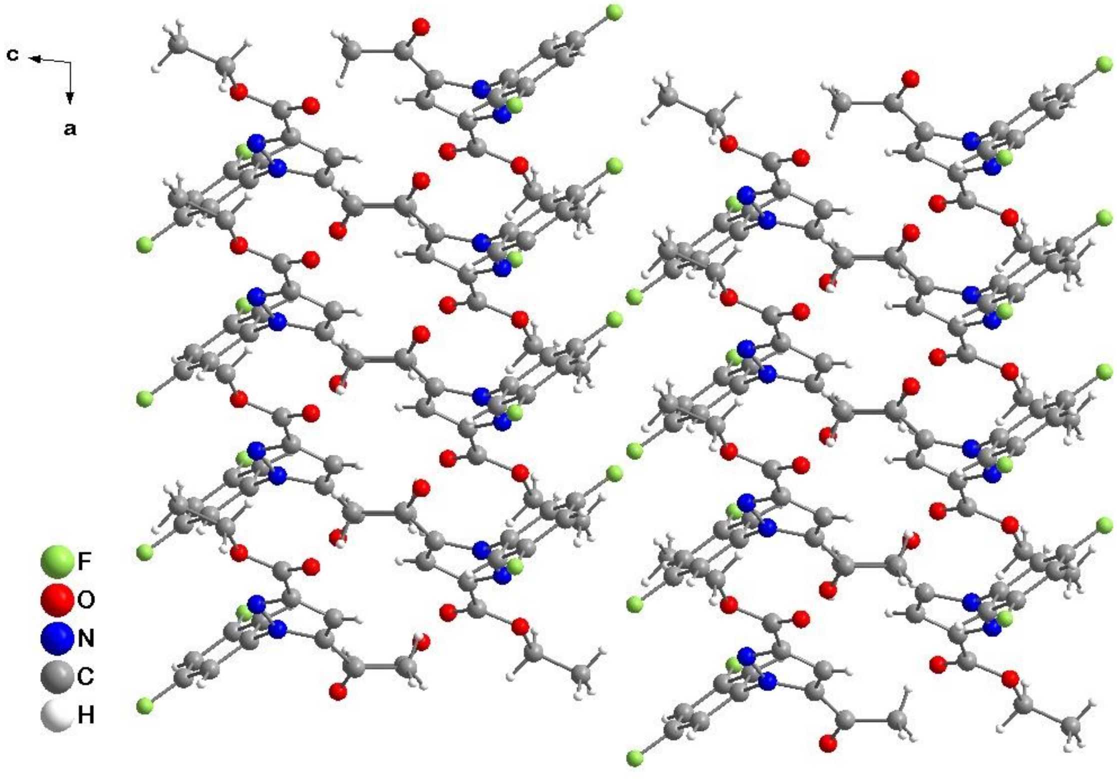

b

Figure S103. Crystal packing of pyrazole **7l** along *a* (**a**) and *b* (**b**) axis.

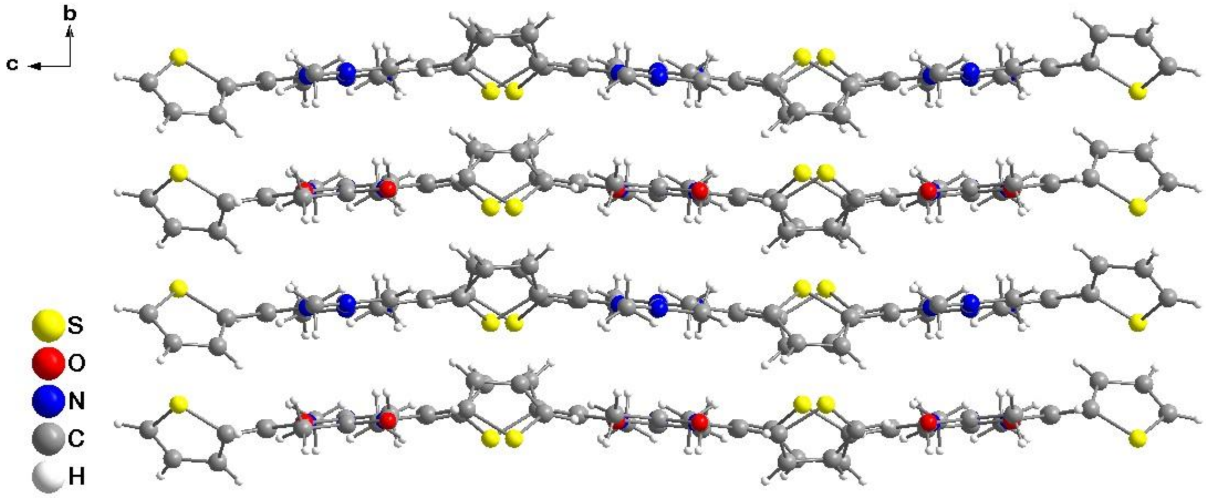

a

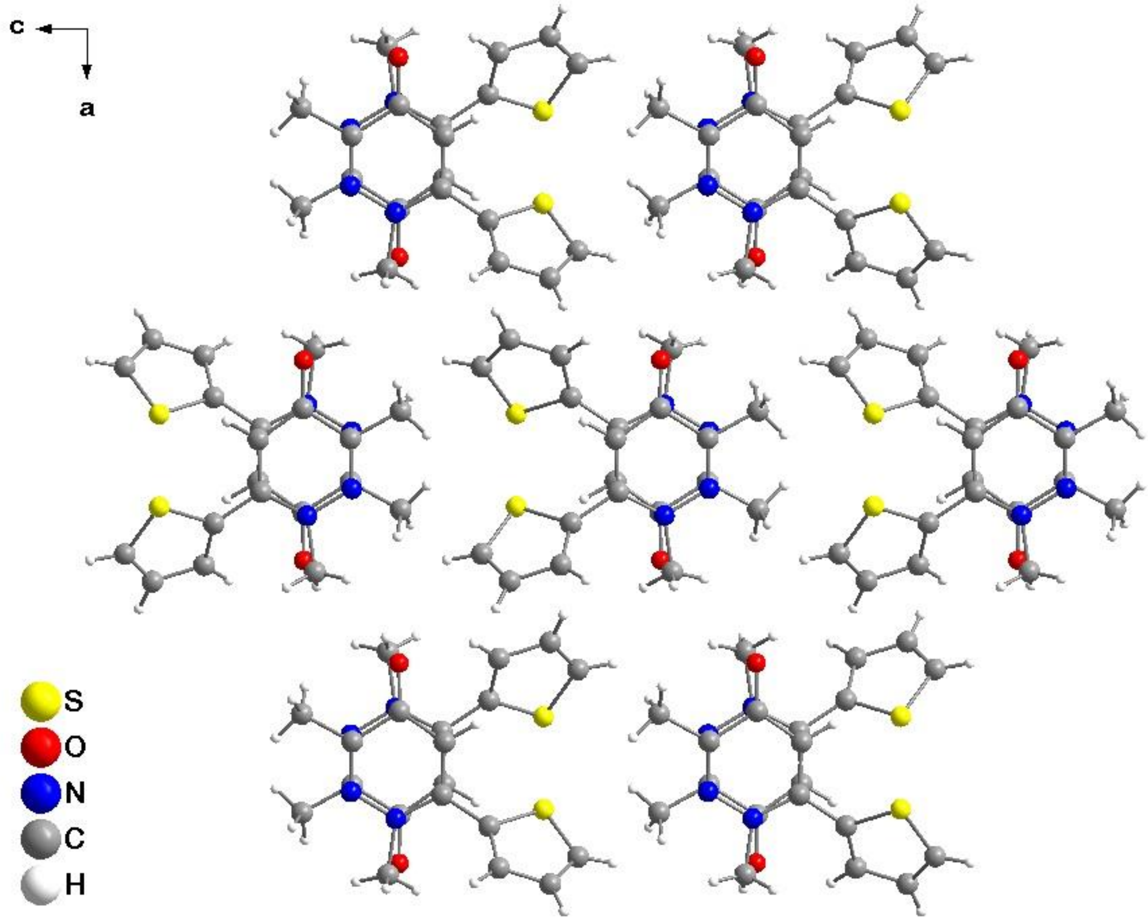

b

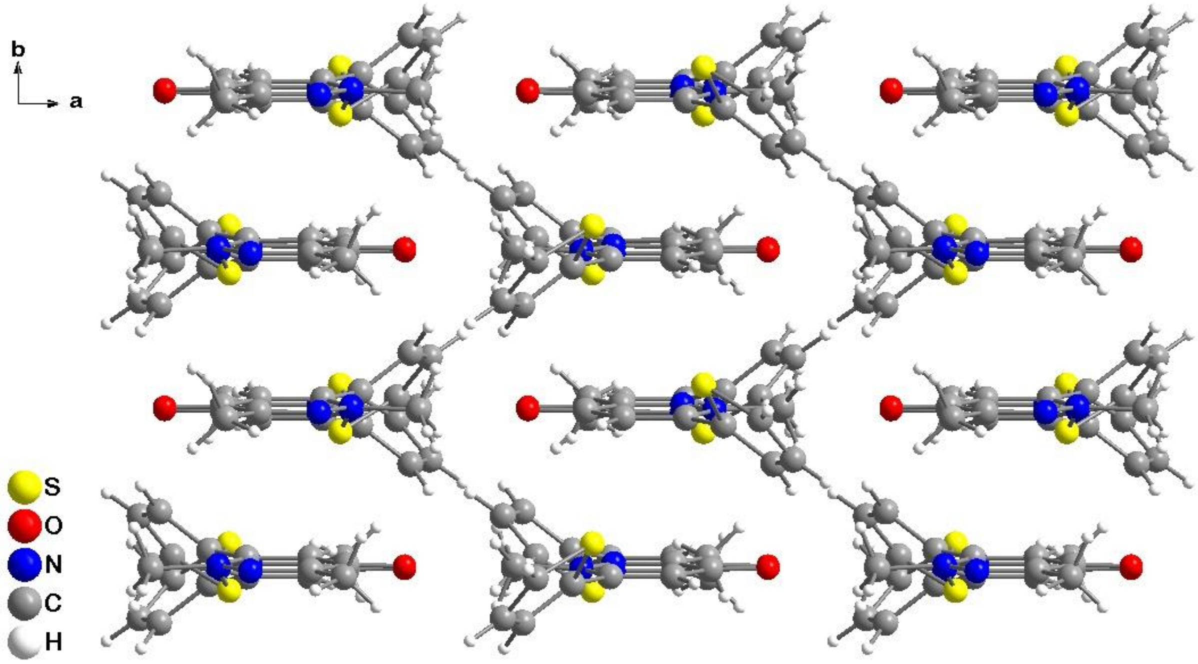

c

Figure S104**.** Crystal packing of pyridazinone **9a** along *a* (**a**), *b* (**b**) and *c* (**c**) axis.

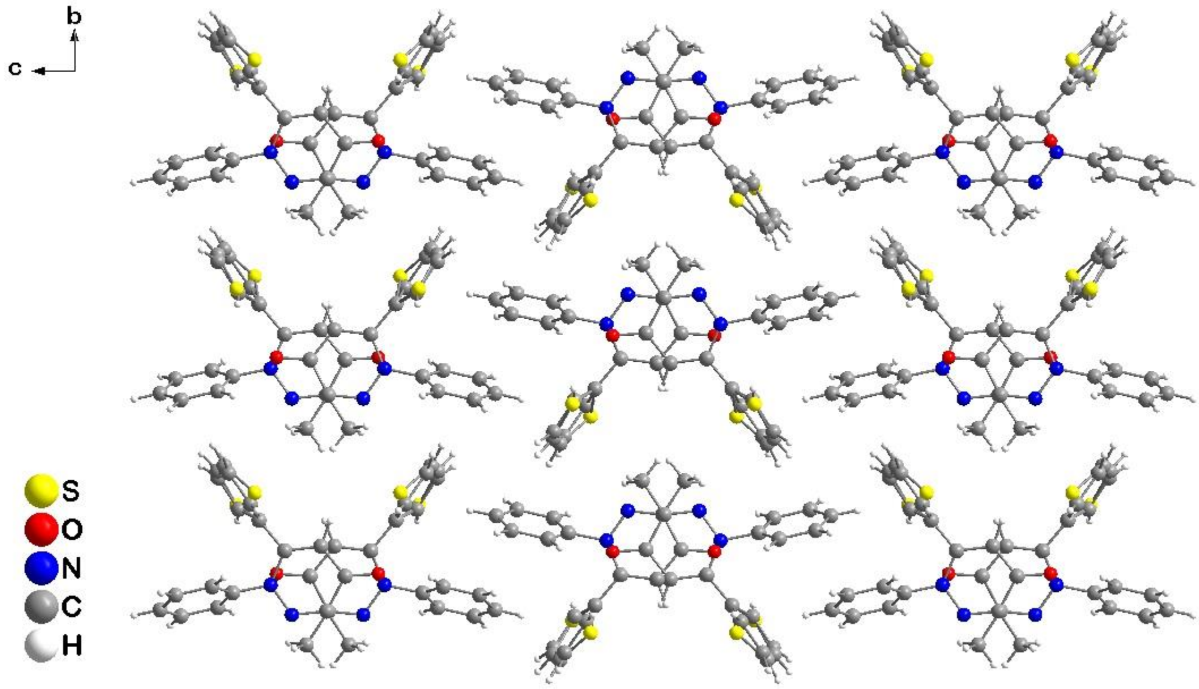

Figure S105. Crystal packing of pyridazinone **9b** along *a* axis.

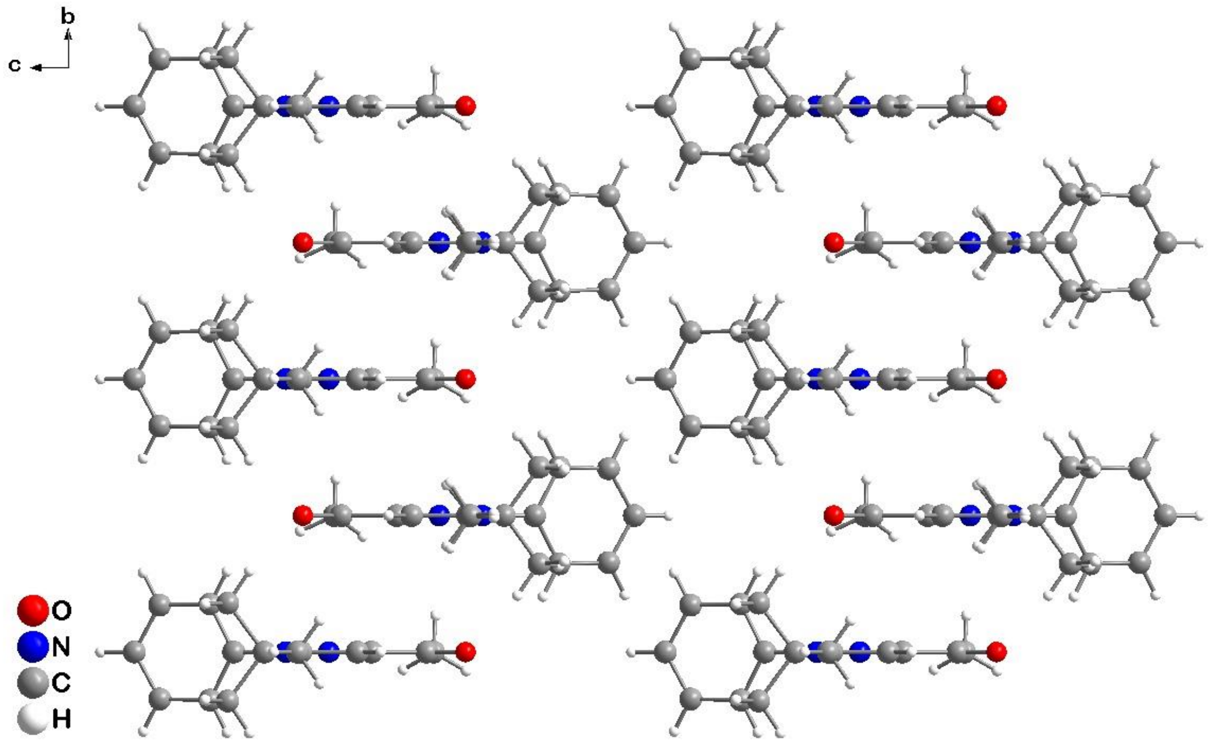

a

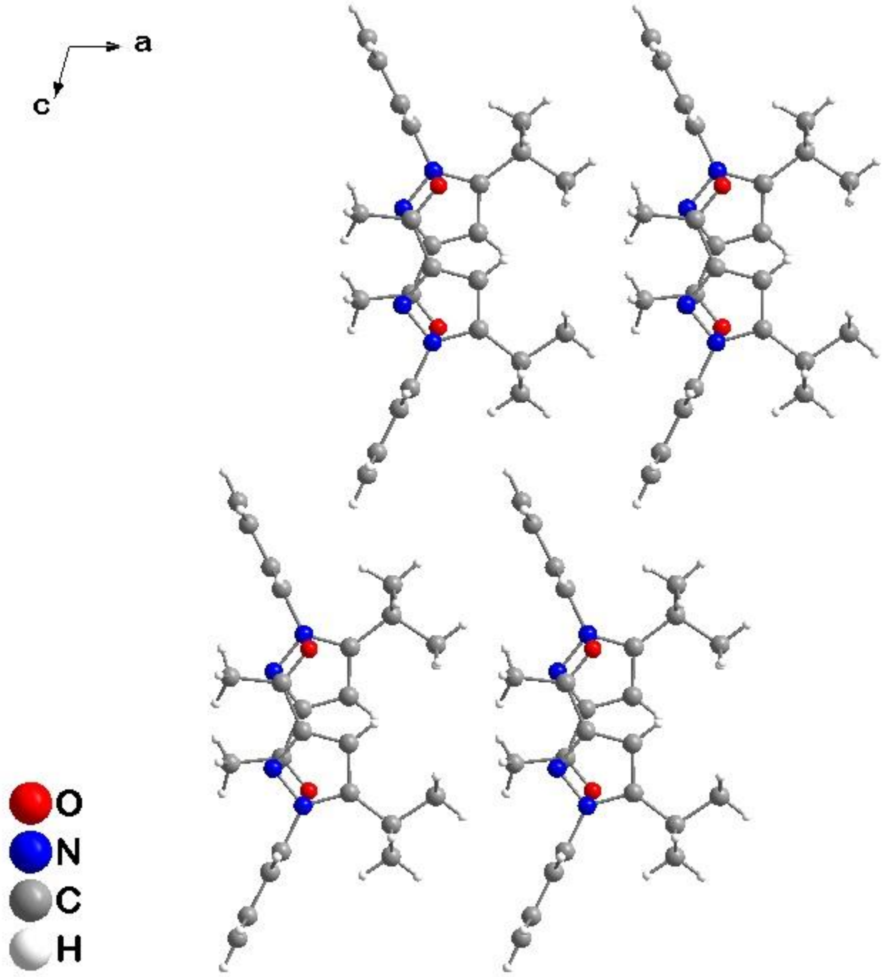

b

Figure S106. Crystal packing of pyrazole **12** along *a* (**a**) and *b* (**b**) axis.

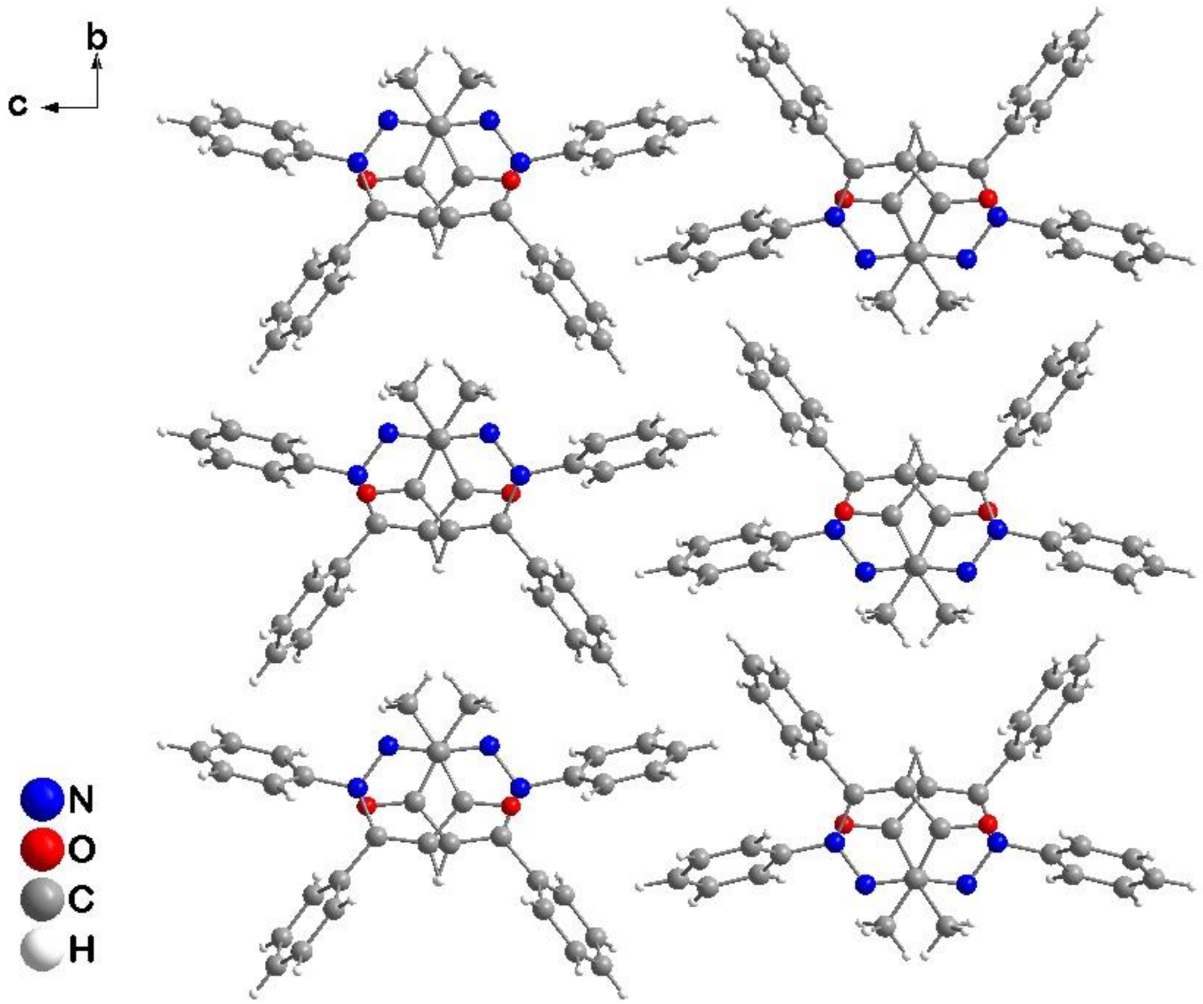

Figure S107. Crystal packing of pyridazinone **14** along *a* axis.

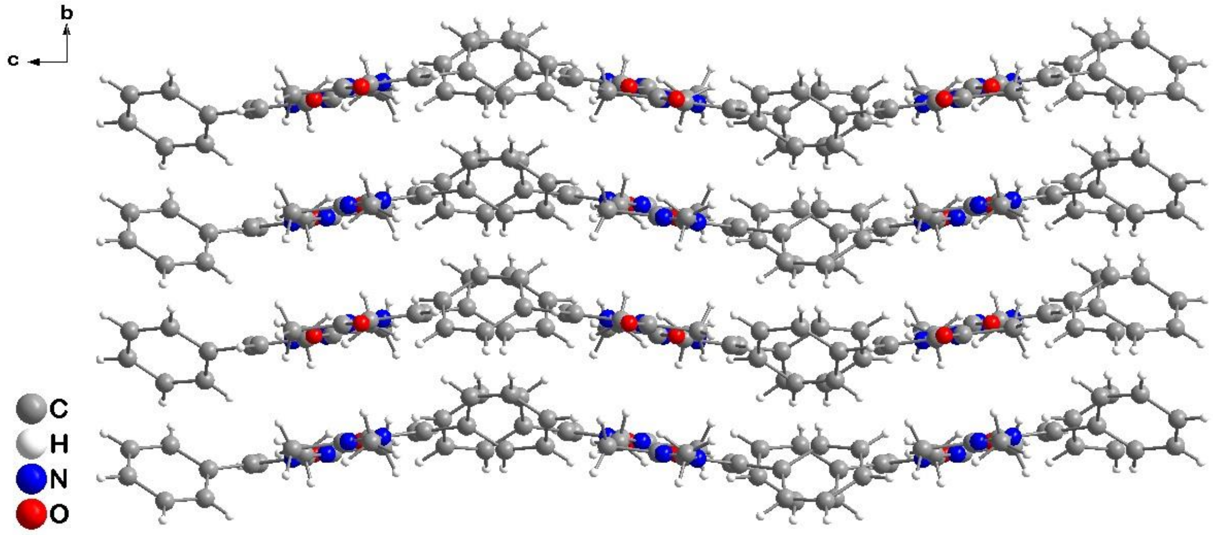

a

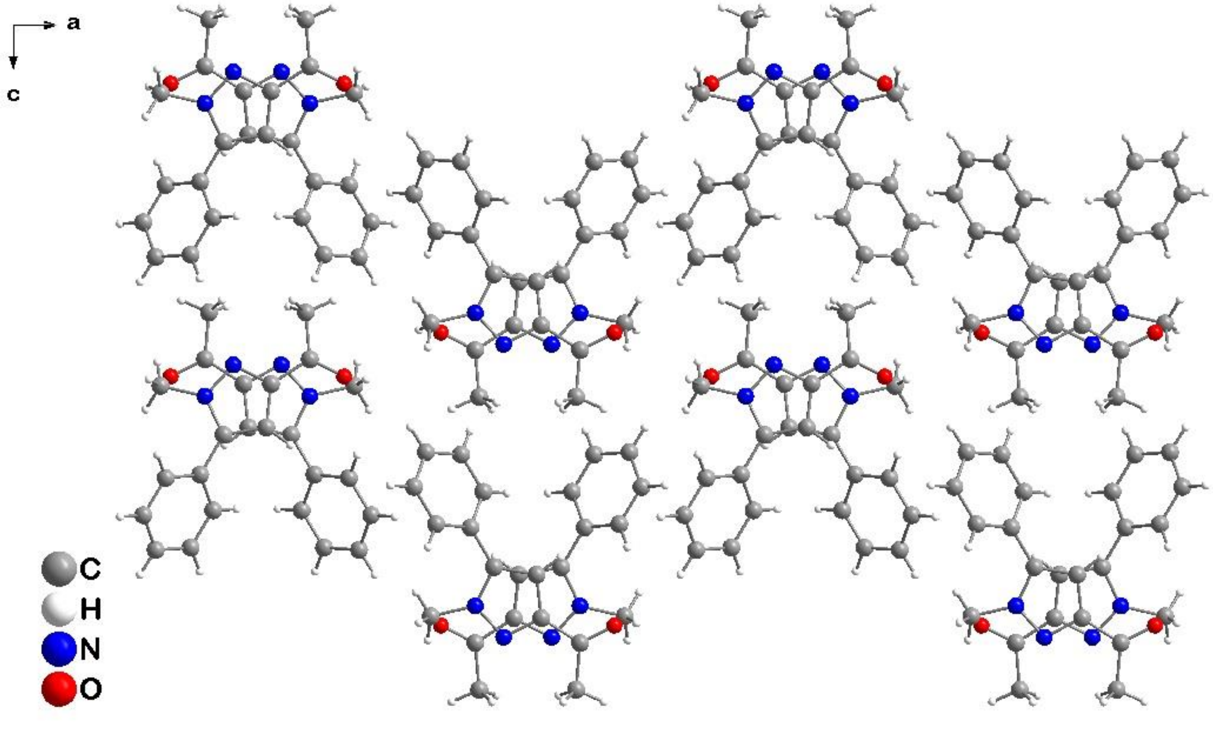

b

Figure S108. Crystal packing of pyrazole **15b** along *a* (**a**) and *b* (**b**) axis.

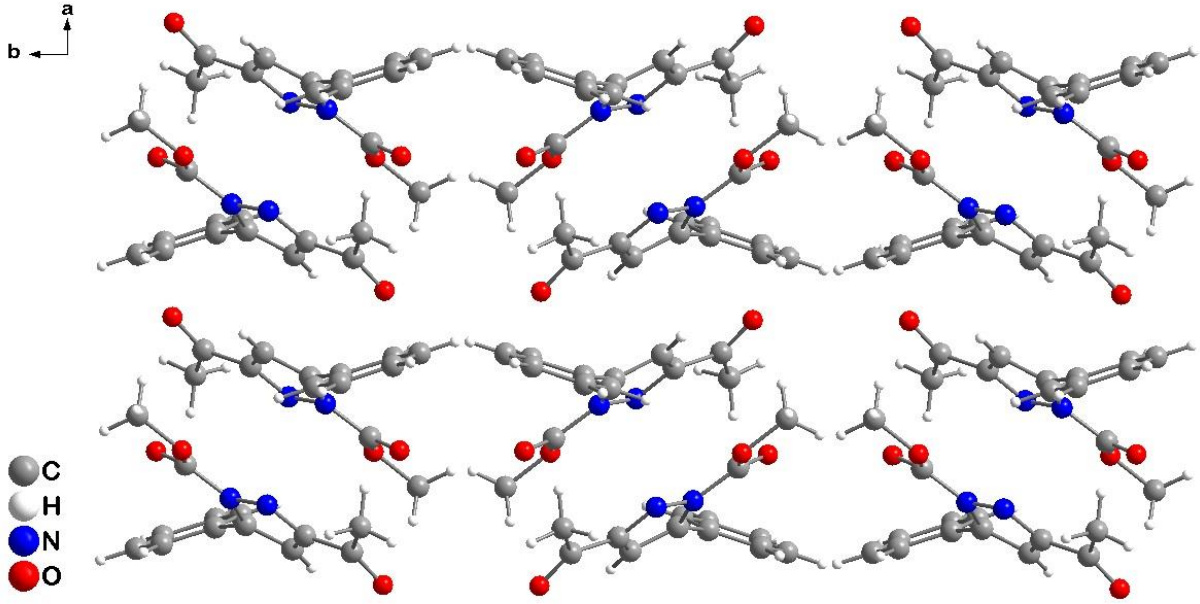

Figure S109. Crystal packing of pyrazole **21b** along *c* axis.

Table S1. Selected bond distances (Å) in diketone **2c**.

	**β-Diketone fragment**		
C–C	C–O	O–H	O–H...O
1.358(9)-1.435(9)	1.257(9)-1.302(11)	1.027(93)	1.57(10)
	**Acetal fragment**		
1.569(12)	1.371(10)-1.42.7(11)	–	–

Table S2. Selected bond distances (Å) in furanones **3** and **5**.

**Compound**	**Furanone fragment**			
	C=C	C–C	C–O	C–Me
**3**	1.344(4)	1.408(4)-1.541(8)	1.322(4)-1.522(9)	1.503(2)
**5**	1.354(4)	1.402(6)-1.531(4)	1.336(4)-1.470(4)	1.505(5)
		**Aromatic ring**		
**3**	1.353(6)-1.409(5)	–	–	–
**5**	1.349(8)-1.386(6)	–	–	–

Table S3. Dihedral angles between the azaheterocyclic and aryl planes in the crystals of compounds **7l**, **9a**, **9b**, **12**, **14**, **15b**, **21b**.

**Compound**	Aromatic substituent at heterocyclic atom N	Aromatic substituent at heterocyclic atom C	Dihedral angle between planes (°)
**7l**	difluorophenyl	–	63.19(23)
**9a**	–	thienyl	46.06(23)
**9b**	phenyl		66.32(7)
**–**		thienyl	65.84(25)
**12**	phenyl	–	89.71(44)
**14**	phenyl		64.21(17)
**–**		phenyl	68.20(24)
**15b**	–	phenyl	44.71(16)
**21b**	–	phenyl	53.05(13)

Table S4. Selected bond distances in pyrazoles **7a**, **7l**, **12**, **15b**, **21b**.

Parameter, Å	**7a**	**7l**	**12**	**15b**	**21b**
C–C (Ph)	–	1.361(6)-1.391(5)	1.363(9)-1.382(9)	1.365(6)-1.398(4)	1.378(5)-1.388(5)
C–C (pyr)	1.374(3)-1.392(3)	1.377(5)-1.387(5)	1.374(1)-1.391(1)	1.370(4)-1.399(5)	1.356(4)-1.399(4)
N–N	1.335(3)	1.346(4)	1.366(9)	1.342(3)	1.363(4)
C-N (pyr)	1.345(3)-1.371(3)	1.336(5)-1.372(4)	1.322(8)-1.361(9)	1.322(8)-1.361(9)	1.327(3)-1.340(4)
C-N	1.451(3)	1.435(4)	1.446(8)	1.452(4)	1.412(3)
C-O (acetyl)	1.210(3)	1.218(5)	1.220(9)	1.218(4)	1.218(4)
C-O (ester)	1.207(3)	1.205(5)	–	–	1.193(4)
	1.333(3)	1.317(5)			1.310(4)

Table S5. Selected bond distances in pyridazinones **9a**, **9b**, **14**.

Parameter, Å	**9a**	**9b**	**14**
C–C (Ph)	-	1.378(3)–1.389(3)	1.361(5)-1.395(4)
C–C (thienyl)	1.329(6)–1.400(5)	1.352(9)–1.440(2)	-
C–C (pyridazinone)	1.370(4)-1.461(4)	1.360(3)-1.469(3)	1.358(3)-1.472(4)
N-N	1.354(4)	1.356(2)	1.359(3)
C-N (pyridazinone)	1.303(4)-1.353(4)	1.304(3)-1.370(3)	1.297(3)-1.363(3)
C-N	1.468(4)	1.444(2)	1.439(3)
C-O	1.249(4)	1.240(3)	1.254(3)

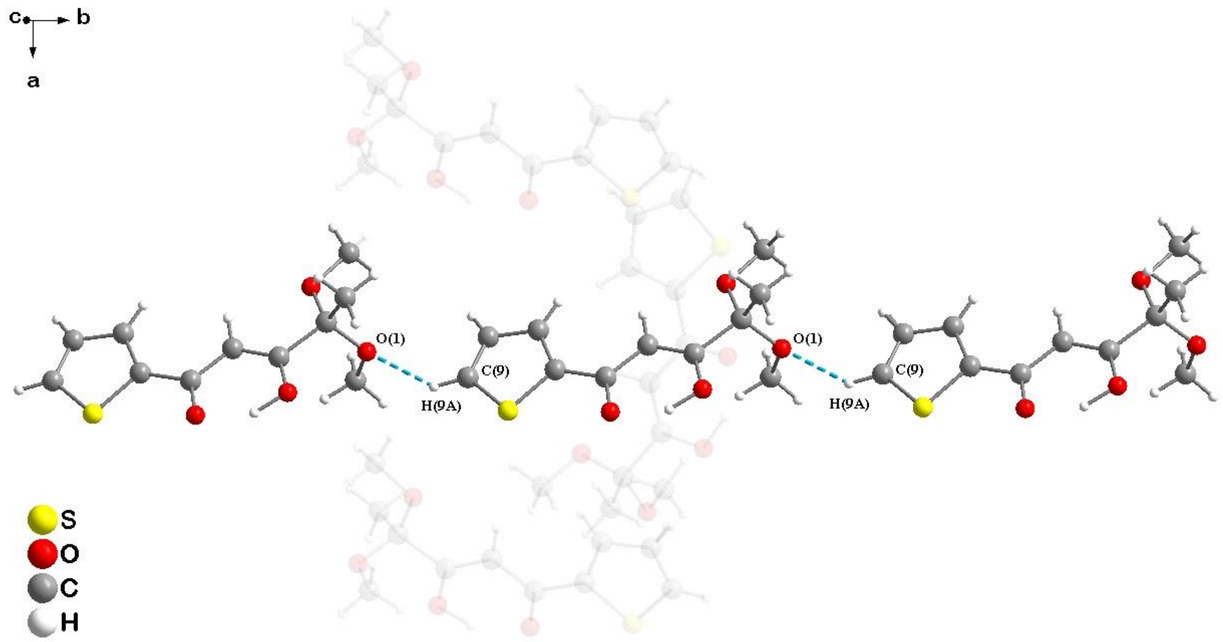

Figure S110. H-contacts in the crystal packing of **2c**.

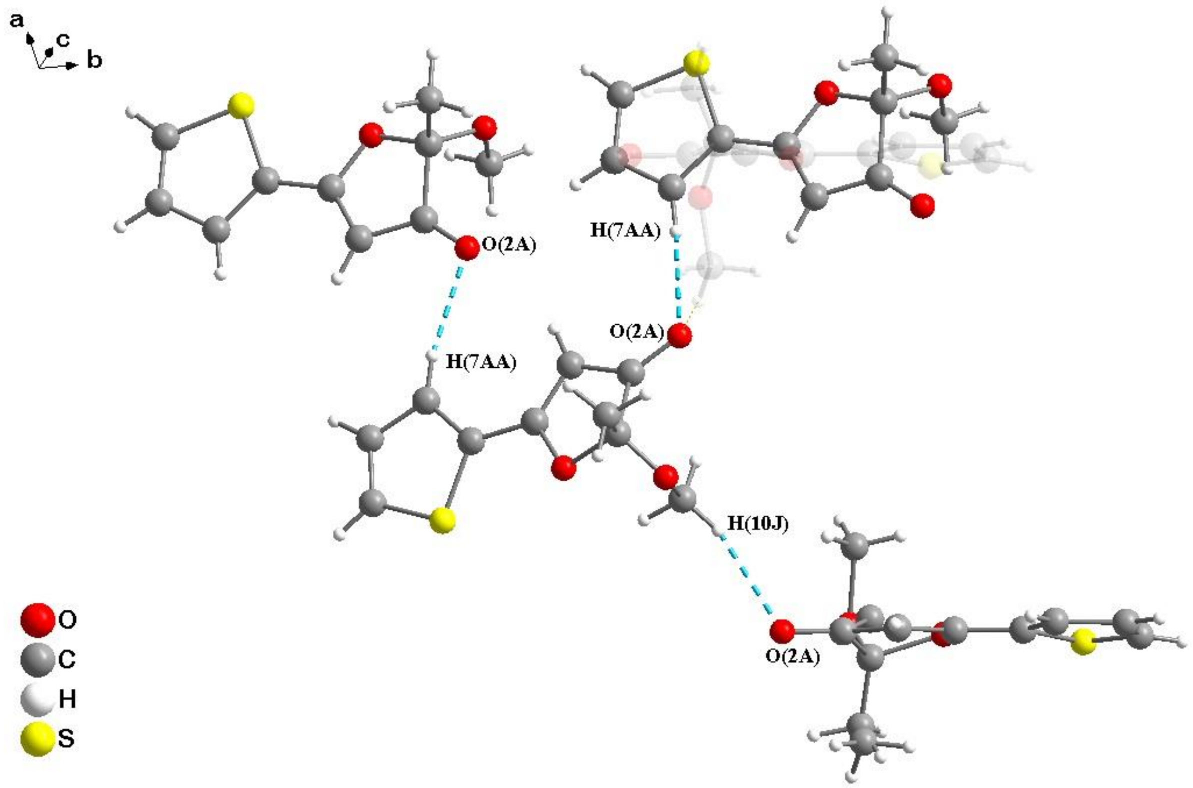

Figure S111. H-contacts in the crystal packing of **3**.

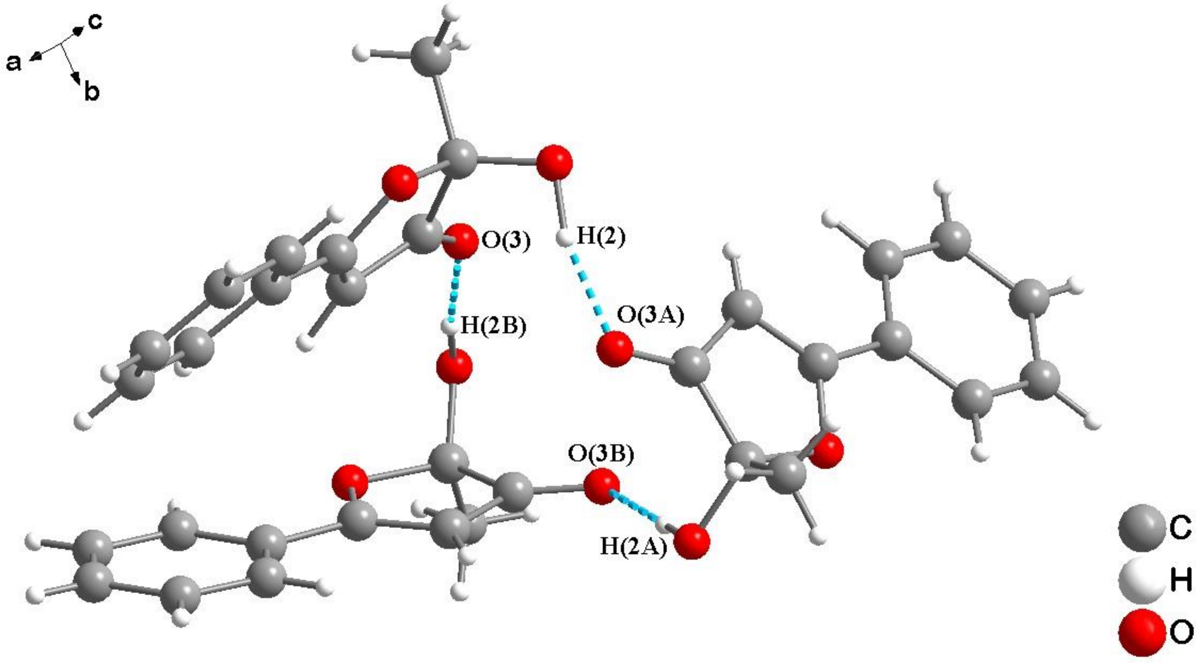

Figure S112. Intermolecular hydrogen bonds in the crystal packing of **5**.

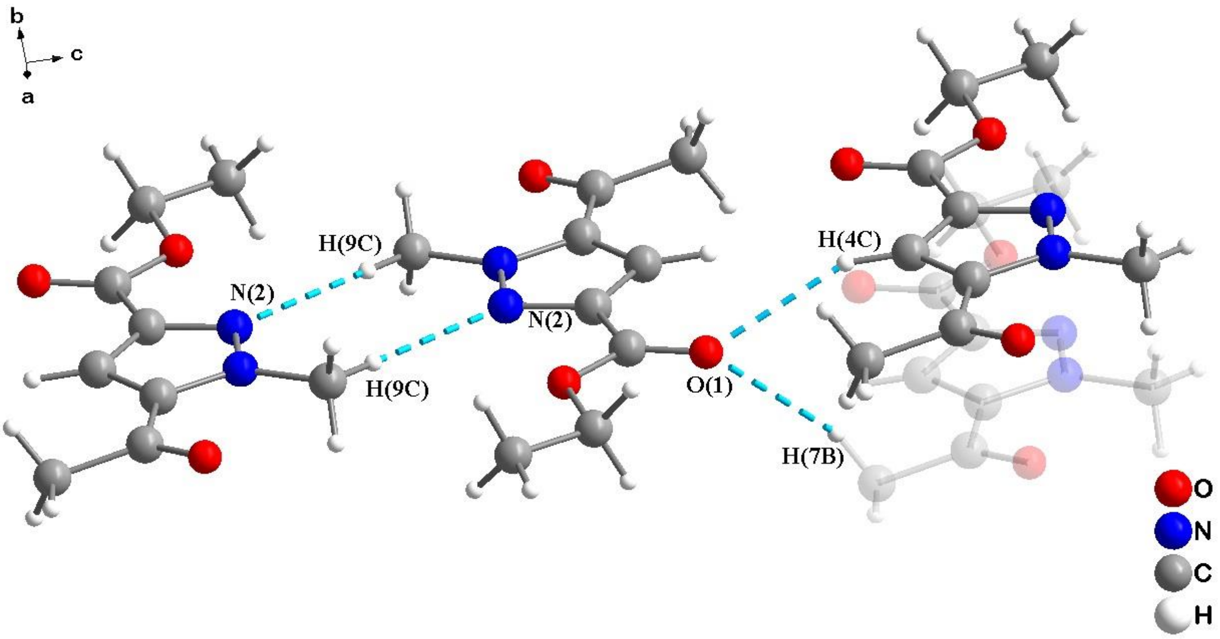

Figure S113. H-contacts in the crystal packing of **7a**.

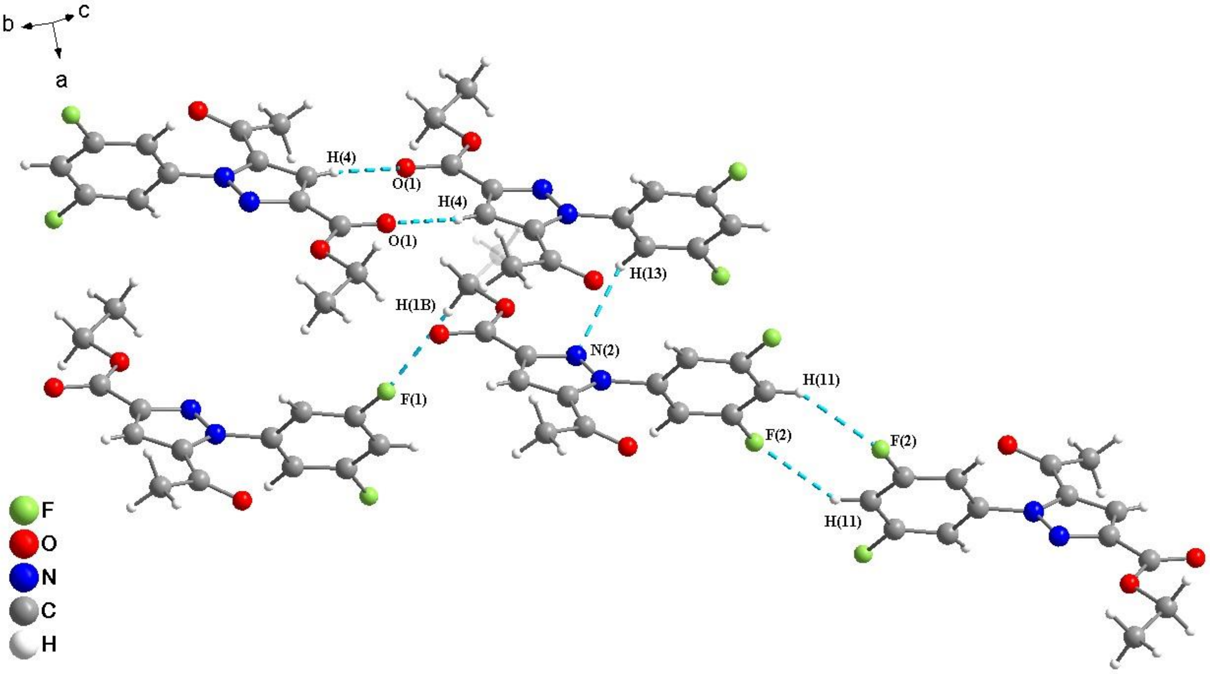

Figure S114. H-contacts in the crystal packing of **7l**.

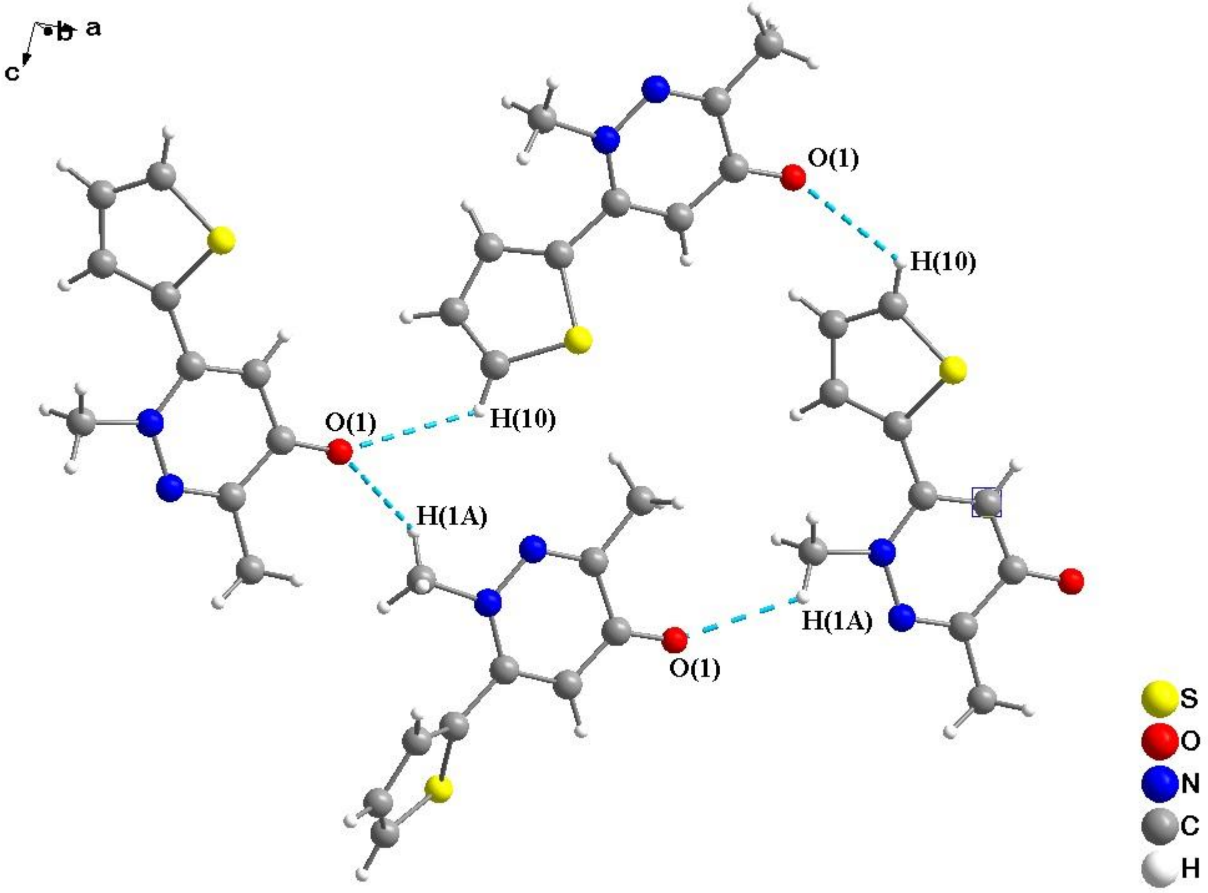

Figure S115. H-contacts in the crystal packing of **9a**.

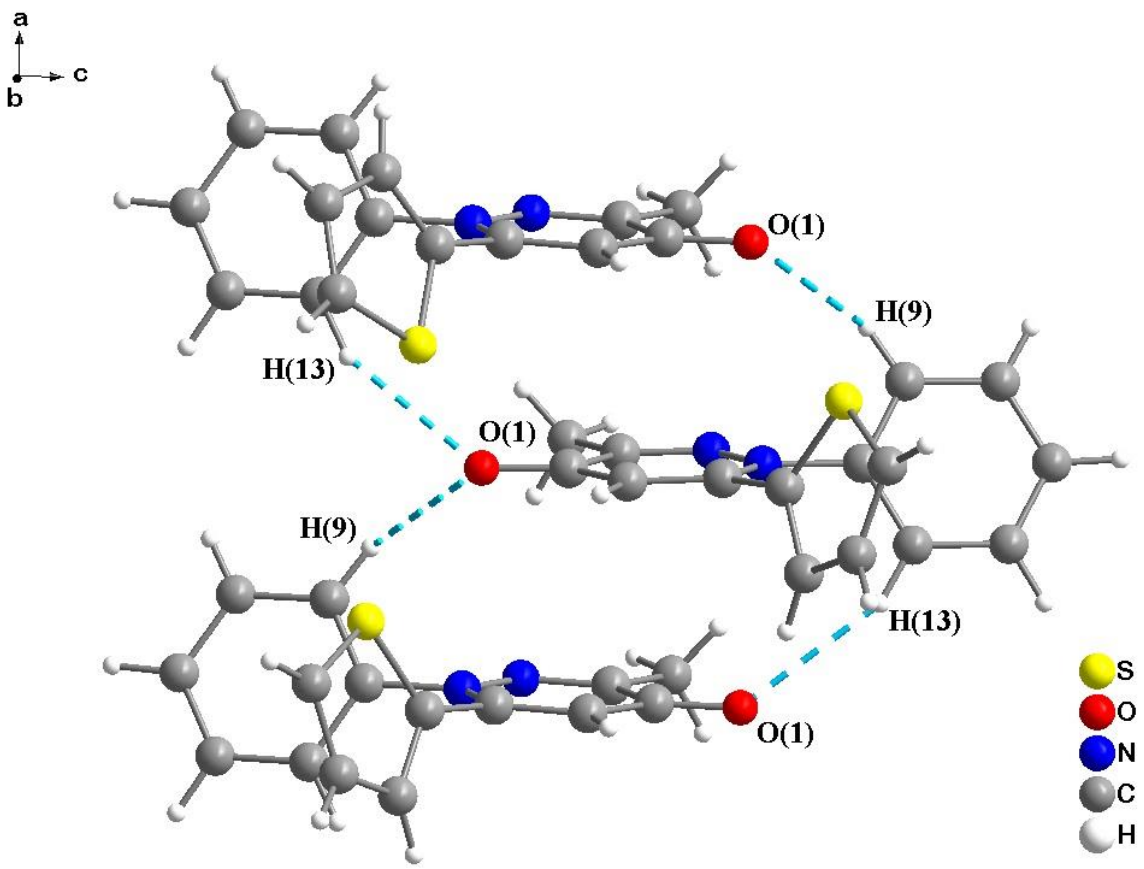

Figure S116. H-contacts in the crystal packing of **9b**.

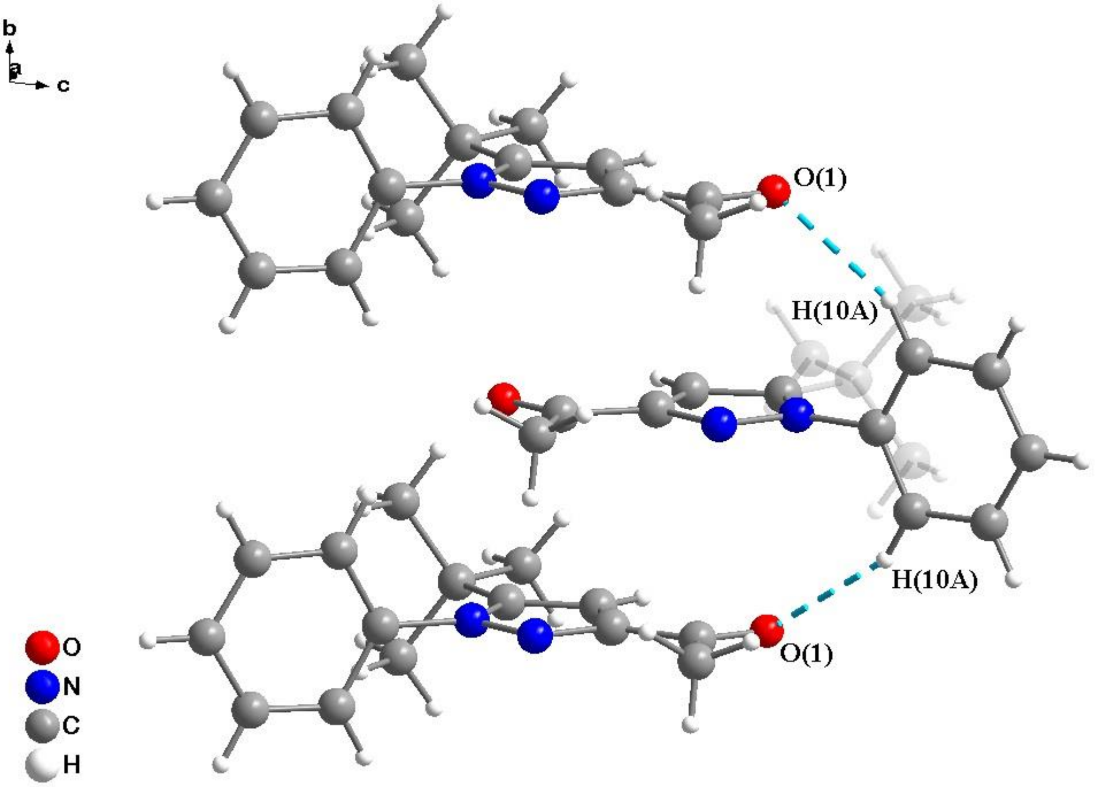

Figure S117. H-contacts in the crystal packing of **12**.

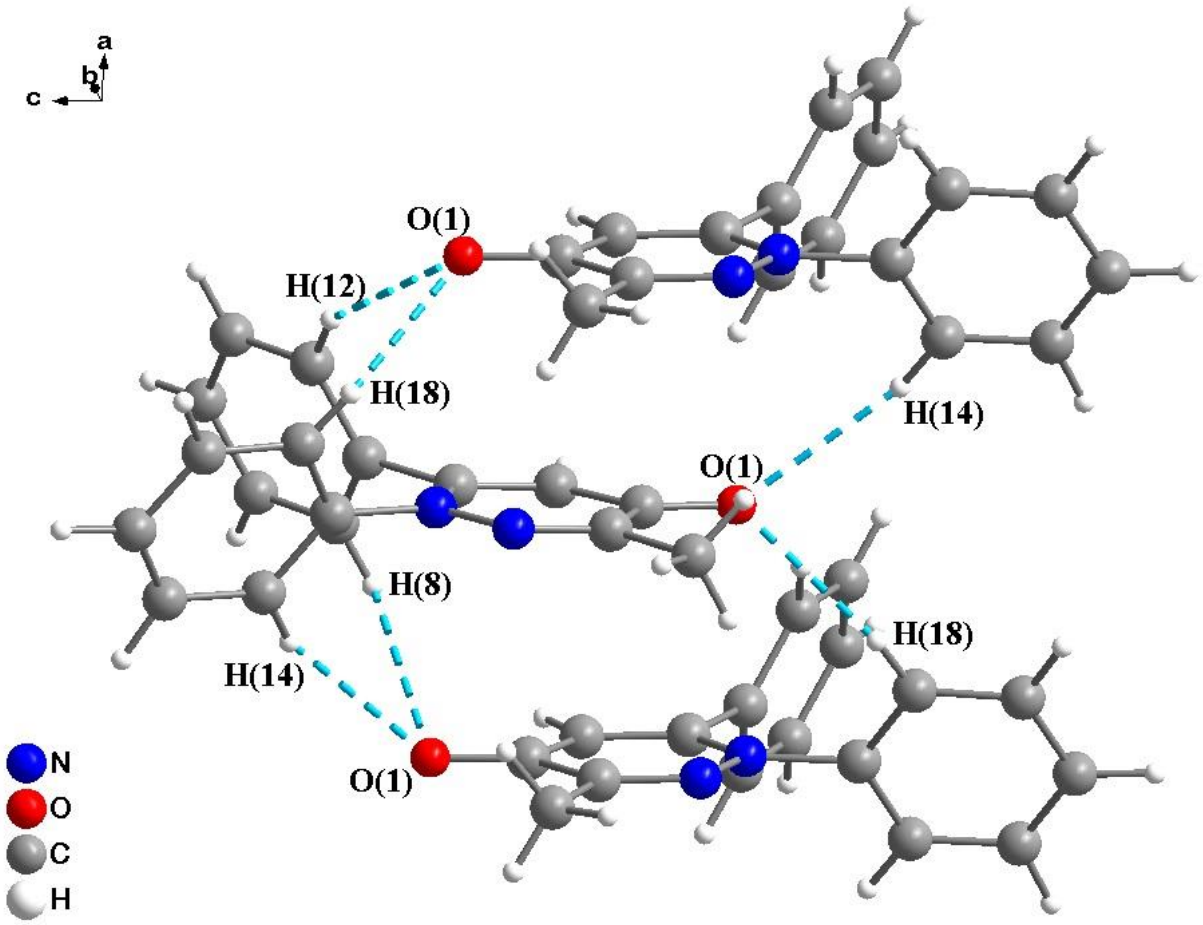

Figure S118. H-contacts in the crystal packing of **14**.

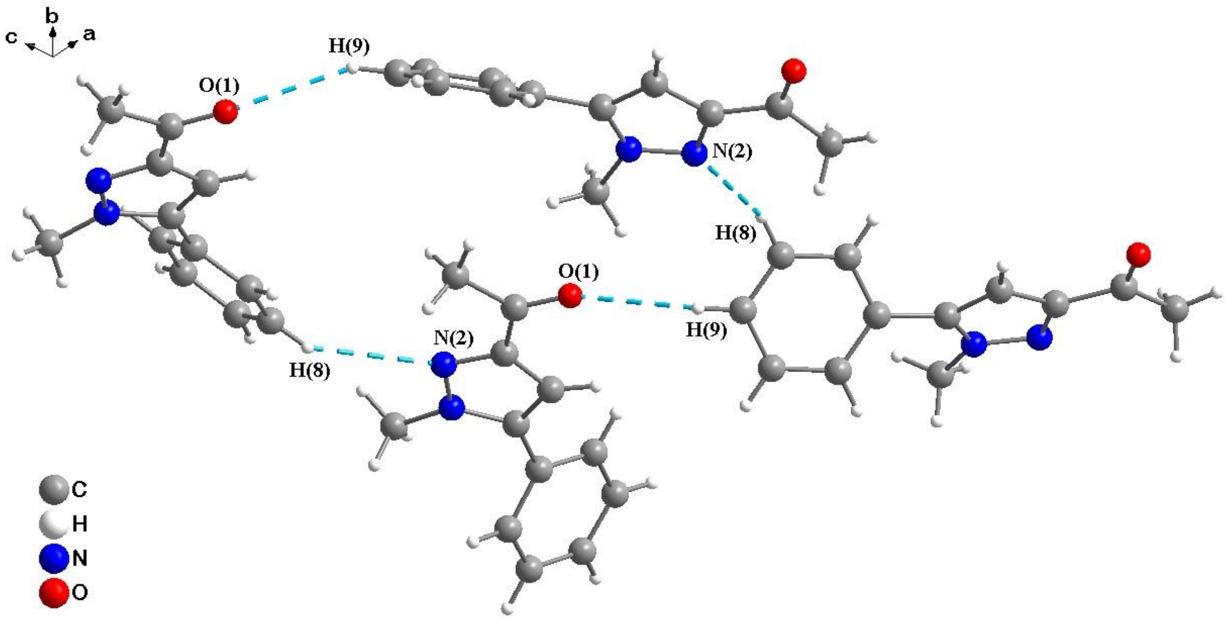

Figure S119. H-contacts in the crystal packing of **15b**.

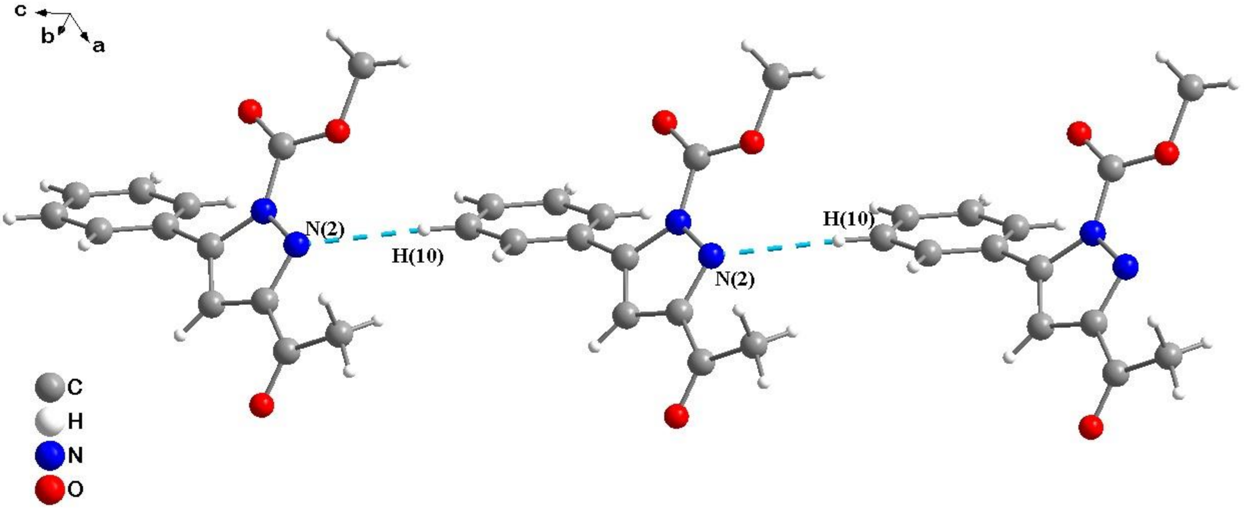

Figure S120. H-contacts in the crystal packing of **21b**.

Table S6. Intermolecular hydrogen bonds and hydrogen-contacts geometry (distances in Å, angles in degrees) in **2c, 3, 5, 7l, 9a, 9b, 12, 14, 15b, 21b**.

Compound	*D*–H···A	D–H	H···*A*	*D*···*A*	∠*D*–H···A	Symmetry
**2c**	C(9)–H(9A)···O(1)	0.930(8)	2.548(7)	3.232(9)	130	x,-1+y,z
**3**	C(7A)–H(7AA)···O(2A)	0.930(3)	2.475(2)	3.379(4)	164	1-x,1/2+y,1/2-z
	C(10B)–H(10J)···O(2A)	0.960(3)	2.411(2)	3.342(3)	163	1+x,3/2-y,1/2+z-
**5**	O(2B)–H(2B)···O(3)	0.929(51)	1.778(52)	2.699(4)	171	2-x,1-y,1-z
	O(2)–H(2)···O(3A)	1.052(66)	1.680(62)	2.695(4)	160	-1+x,y,z
	O(2A)–H(2B)···O(3B)	1.008(56)	1.731(55)	2.729(4)	169	–
**7a**	C(9)–H(9C)···N(2)	0.960(3)	2.610(2)	3.567(3)	174	1-x,1-y,-z
	C(4)–H(4C)···O(1)	0.987(2)	2.473(25)	3.401(3)	156	-x,1-y,1-z
	C(7)–H(7B)···O(1)	0.960(3)	2.680(2)	3.517(4)	146	2-x,1-y,1-z
**7l**	C(11)–H(11)···F(2)	0.907(37)	2.591(33)	3.381(5)	146	2-x,-y,1-z
	C(13)–H(13)···N(2)	1.049(44)	2.719(43)	3.295(5)	114	-1+x,y,z
	C(1)–H(1B)···F(1)	0.971(4)	2.620(3)	3.570(5)	163	x,1+y,z
	C(4)-H(4) ··O(1)	0.825(45)	2.574(43)	3.376(5)	165	1+x,y,1+z
**9a**	C(1)–H(1A)···O(1)	0.960(4)	2.604(2)	3.206(4)	121	-1/2+x,3/2-y,1-z
	C(10)–H(10)···O(1)	0.929(4)	2.945(2)	3.558(5)	125	-x,-1/2+y,z
**9b**	C(9)–H(9)···O(1)	0.943(32)	2.518(31)	3.244(3)	134	1/2+x,y,1/2-z
	C(1)–H(1A)···O(1)	0.987(26)	2.488(24)	3.388(3)	152	-1/2+x,y,1/2-z
**12**	C(10A)–H(10A)···O(1)	0.930(7)	2.510(5)	3.367(8)	153	x,1+y,1+z
**14**	C(14)–H(14)···O(1)	0.969(27)	2.598(26)	3.502(31)	156	-1/2+x,y,1/2-z
	C(8)–H(8)···O(1)	0.930(3)	2.850(22)	3.564(4)	134	-1/2+x,y,1/2-z
	C(18)–H(18)···O(1)	0.919(26)	2.535(25)	3.275(3)	138	-1/2+x,y,1/2-z
	C(12)–H(12)···O(1)	0.930(3)	2.709(2)	3.559(3)	152	1/2+x,y,1/2-z
**15b**	C(8)–H(8)···N(2)	0.930(4)	2.944(2)	3.744(5)	145	-1/2+x,1/2-y,1-z
	C(9)–H(9)···O(1)	0.930(3)	2.730(3)	3.585(4)	153	-1/2+x,-1+y,1/2-z
	C(10)-H(10) ··N(2)	0.930(4)	2.635(3)	3.540(5)	164	–
